# Automated Planar Tracking the Waving Bodies of Multiple Zebrafish Swimming in Shallow Water

**DOI:** 10.1371/journal.pone.0154714

**Published:** 2016-04-29

**Authors:** Shuo Hong Wang, Xi En Cheng, Zhi-Ming Qian, Ye Liu, Yan Qiu Chen

**Affiliations:** 1 School of Computer Science, Shanghai Key Laboratory of Intelligent Information Processing, Fudan University, Shanghai, P. R. China; 2 Jingdezhen Ceramic Institute, Jindezhen, Jiangxi, P.R. China; 3 Chuxiong Normal University, Chuxiong, Yunnan, P. R. China; 4 College of Automation, Nanjing University of Posts and Telecommunications, Nanjing, Jiangsu, P. R. China; Chinese Academy of Sciences, CHINA

## Abstract

Zebrafish (*Danio rerio*) is one of the most widely used model organisms in collective behavior research. Multi-object tracking with high speed camera is currently the most feasible way to accurately measure their motion states for quantitative study of their collective behavior. However, due to difficulties such as their similar appearance, complex body deformation and frequent occlusions, it is a big challenge for an automated system to be able to reliably track the body geometry of each individual fish. To accomplish this task, we propose a novel fish body model that uses a chain of rectangles to represent fish body. Then in detection stage, the point of maximum curvature along fish boundary is detected and set as fish nose point. Afterwards, in tracking stage, we firstly apply Kalman filter to track fish head, then use rectangle chain fitting to fit fish body, which at the same time further judge the head tracking results and remove the incorrect ones. At last, a tracklets relinking stage further solves trajectory fragmentation due to occlusion. Experiment results show that the proposed tracking system can track a group of zebrafish with their body geometry accurately even when occlusion occurs from time to time.

## Introduction

Collective motion of animal groups is one of the most common yet spectacular phenomenon in nature, which has attracted great attention of scientists from many disciplines. Various theoretical models have been developed to explain and simulate such collective motion including boids model, Vicsek model, etc. [1–6]. By studying such collective behavior, scientists are able to investigate neural cognitive mechanisms behind such behaviors and the research findings may also serve as source of inspiration for man-made systems. For example, simulated evolutionary algorithms were proposed to solve optimization problems [7, 8], collective behavior models were applied to help model complex traffic and transportation processes [9] and develop intelligent robots [10]. Multi-object tracking via video camera makes it possible to discover new principles underlying these collective behaviors because it can accurately acquire motion data of different organism groups without tedious manual work or pasting markers on the tracked objects and the trajectory data of them is essential for quantitatively analyzing their collective behavior [11–25].

Zebrafish (*Danio rerio*) is widely adopted as a model organism by biologists. By tracking a single zebrafish (which have been accomplished by existing computer softwares such as ANY-maze^®^ and EthoVision^®^) biologists can investigate individual behavior of zebrafish under various circumstances. In order to study their social behavior, multi-object tracking is an effective way. Fish swim in 3D space and 3D tracking is certainly most informative for investigating their behavior [19], but considering that the water is shallow in many experiments and the shape of fish group is more spread in horizontal plane than vertical plane, 2D tracking is accurate enough to describe their trajectories.

Most of the existing 2D multi-object tracking systems treat each tracked individual as a single point. Miller *et al.* developed a system to track a fish group by firstly clicking on the snout or body of each fish manually [15], which requires large amount of human effort when the fish group is large. EthoVision (2.3 and more recent versions) [11, 26, 27], a tracking system widely used by biologists, can detect and track the barycenter of different kinds of organisms. However, the number of objects is limited and when occlusion occurs, the identity of the objects is unable to be remained, what’s worse, the strict luminosity condition is necessary to guarantee the performance of tracking. Color detection based tracking systems (such as EthoVision Color-Pro^®^) use color tags to efficiently resolve the individual identification problem even when occlusion occurs [21]. Ylieff *et al.*[28] used color plastic pearls attached under the dorsal fin of fish to simultaneously track up to 3 fish per aquarium. Delcourt *et al.* [29] used visible implant elastomer (VIE) tags to simultaneously track 4 glass eels (*Anguilla anguilla*). To guarantee that the color differentiation is sufficient for the tracking system to differentiate the individuals, the number of simultaneously tracked individuals is limited. And those tags may potentially affect social behaviour of tracked individuals. Delcourt *et al.* proposed a multitracking system which can detect and track barycenter of up to 100 fish [13]. The correct identity of each individual can be recovered after occlusion events, but the system is not capable of long time tracking. Qian *et al.* proposed a novel fish head detector based on ellipse fitting and track a group of fish based on fish head detection [24]. But when severe occlusion occurs, using only image features and motion continuity of fish head is not enough to ensure the correct identity of each individual. Recently, ‘fingerprinting’ based tracking system such as idTracker proposed by Alfonso *et al.* [25] found another way to accomplish the task, that is, to use a set of traits to recognize each tracked object, thus, after occlusion the identity of each object will be remained. The limitation of it is when the number of objects is large, the error rate of identification will increase significantly due to similar appearance of the tracked objects.

However, in all the above mentioned point based tracking systems, fish body is approximated as a single point and its highly dynamic and complex body geometry which is valuable for research of fish swimming performance and hydrodynamics [30, 31] cannot be adequately described. Blob-contour based tracking systems such as [12] can track complex contour of animals, but they suffer from high time complexity resulting from large amount of samples. And the tracking performance is not robust when occlusion occurs. Body model based tracking is another strategy to achieve the geometry of fish body. Different mathematical models have been proposed to model fish body. The state vector in tracking system is thus composed by parameters of the fish model and variables related to the motion of the object. Mirat *et al.* separated fish body into two parts, namely head and tail part [20]. The head part is regarded as linear, the tail part may bend as a curve. The tail-angle was investigated and used to analyze the locomotion of fish. But this tail-angle definition only considers the start and end point of tail part which ignores specific shape of fish tail. Fontaine *et al.* also separated head and tail part and applied B-spline basis functions to describe the body wave [16]. This novel model combined with iterative Kalman filter for contour tracking achieved good results. However, the method is semi-automated which need manual initialization and only one zebrafish is tracked, when applied to track a fish group, it will suffer from relatively high dimensionality and the tracking procedure requires high frame rate (1500fps). What’s more, the parameters of these fish models do not have direct physical meanings.

In order to overcome these limitations, this paper proposes a chained rectangle fish body model. The whole fish body is discretized into several linked rectangles with adaptable sizes. The head of fish is tracked first and then the rectangle chain of the body part is fitted. In body fitting stage, the correctness of head tracking result will be verified and some of tracking errors can be eliminated. A novel fish head detector is proposed which can accurately detect the location and orientation of each fish head via curvature when the fish head is not occluded. In the tracking stage Kalman filter is applied to track fish head effectively and accurately. After the head of each fish is successfully tracked, the body rectangles of body part can be accurately fitted. And when severe occlusion occurs, the motion continuity of fish head and body can help to resolve the association ambiguity at most times.

## The Proposed Tracking System

**Fig 1:**
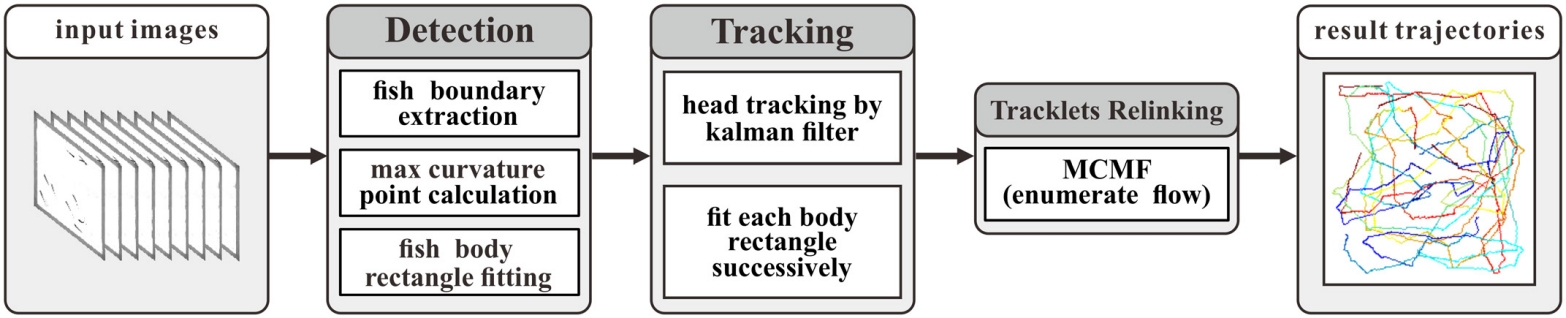
System workflow. The proposed tracking system has three main stages, namely fish detection, fish tracking and tracklets relinking. The first two stages are repeated until all the images have been processed. Tracklets relinking is a postprocessing stage which further solves trajectory fragmentation caused by occlusion and detection error.

The proposed tracking system is composed of three main stages, namely fish detection, fish tracking and tracklets relinking (as shown in Fig 1). The first two stages are repeated until all the images have been processed to produce preliminary tracklets. The tracklets are then relinked to create complete trajectory for each fish.

### 2.1 Ethics statement

All experimental procedures were in compliance with the Institutional Animal Care and Use Committee (IACUC) of Shanghai Research Center for Model Organisms (Shanghai, China) with approval ID 2010-0010, and all efforts were made to minimize suffering. This study was approved by the Institutional Animal Care and Use Committee (IACUC), and written informed consent was obtained.

### 2.2 Fish model

The simplest fish model used by most existing tracking systems is ‘point model’ [11, 13, 15, 24, 25], meaning that a fish is represented by a 2D point location. However, as fish propels itself by bending its body into a backward-moving propulsive wave that extends to its caudal fin or uses its median and pectoral fins, forming a curve like shape [32], this kind of deformation is highly non-rigid, so it is not sufficiently accurate to model it as a single point plus orientation.

**Fig 2:**
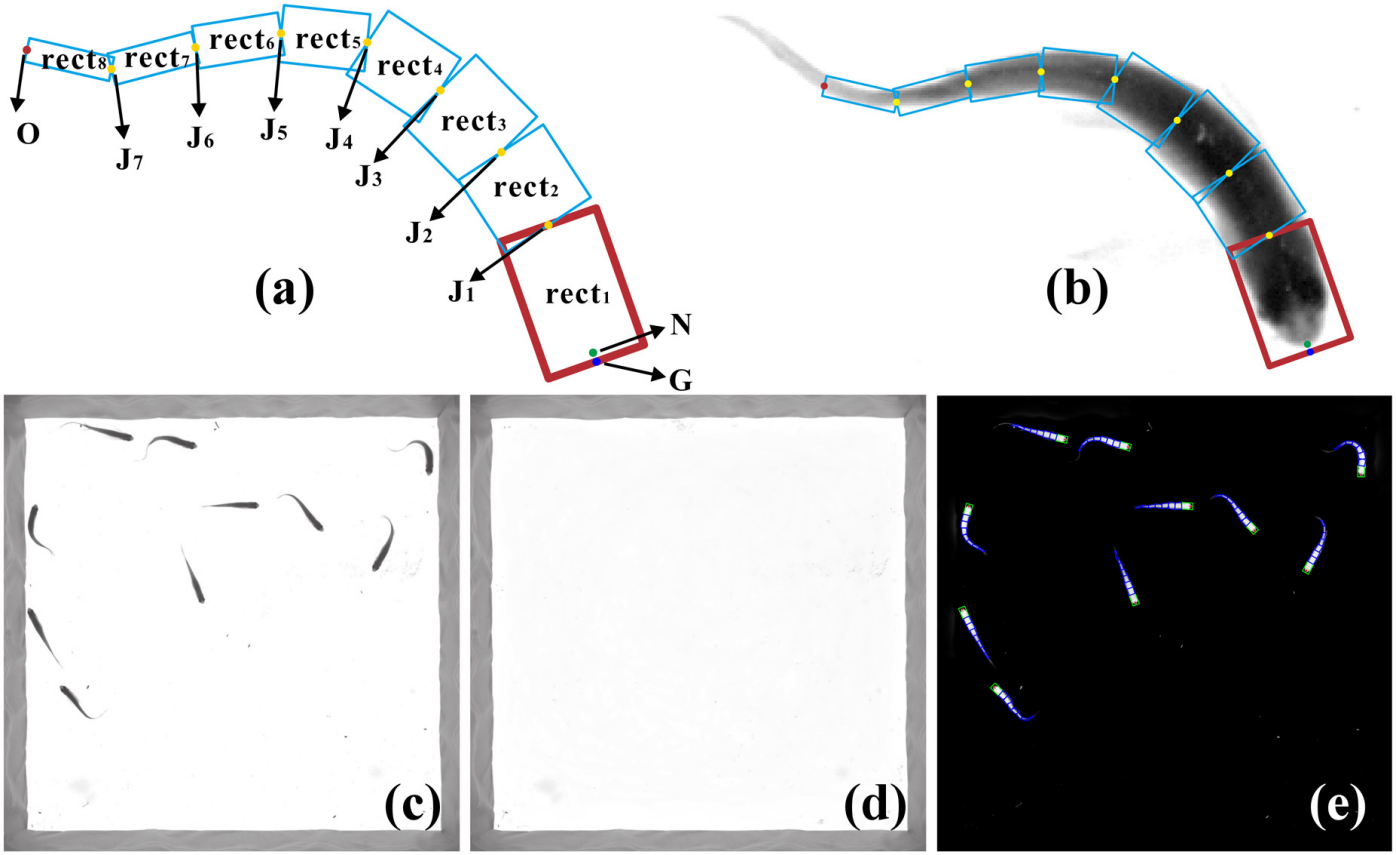
Fish detection. (a). Fish model. The whole fish body is discretized into 8 linked rectangles with adaptable sizes. Green point (point *N*) denotes fish nose point. Yellow points mark the joint points. Blue point (point *G*) and red point (point *O*) is the midpoint on front edge of *rec*_1_ and back edge of *rec*_8_ respectively; (b). Fish model mapped onto real image; (c). One sample image captured with a high speed camera; (d). Background image achieved by calculating average pixel value of a large number of images with fish in the tank; (e). Result of image background subtraction, fish head detection and body fitting by the proposed system.

From the top view, the motion of zebrafish group swimming in shallow water (about 10cm deep) is approximately restricted within a plane. The fish body consists of an almost rigid fish head [33] and a curve-like fish body part. And fish body has vertebras that act as joints to form tail geometry [34]. Inspired by this observation, we model the fish body as a chain of rectangles with adaptive sizes as shown in Fig 2a. The rectangles are denoted by *rec*_*i*_(*i* = 1, …, *n*_*r*_ from fish head to tail) where *n*_*r*_ is the total number of rectangles, the length and width of *rec*_*i*_ is defined as *len*_*i*_ and *wid*_*i*_ respectively. *len*_1_ is greater than *len*_*i*_,(*i* = 2, …, *n*_*r*_) to guarantee the nose of fish in the image is within the boundary of *rec*_1_ (This strategy is also helpful for head rectangle similarity measurement). The joint of two adjacent rectangles *rec*_*i*_ and *rec*_*i*+1_ is denoted as *J*_*i*_ = (*x*_*i*_, *y*_*i*_)(*i* = 1, …, *n*_*r*_−1). The midpoint on front edge of *rec*_1_ is denoted as *G* = (*x*, *y*).

In our implementation, *n*_*r*_ is set to 8 (meaning there are 8 rectangles in total including head rectangle), as we found that this 8-rectangle model achieves a good balance between model complexity and description accuracy of body geometry. The length of each body rectangle is set to 1/8 of average fish length. The length and width of *rec*_*i*_ are shown in Table 1.

**Table 1:** Length and width of each rectangle.

rectangle no.	1 (head rectangle)	2	3	4	5	6	7	8
length (pixel)	50	30	30	30	30	30	30	30
width (pixel)	35	35	35	26	20	15	11	8

Considering that the fish in our experiments are of almost the same size, the rectangle sizes for each fish are the same and predefined. Thus, the parameters of the fish model are the location and orientation of each rectangle. If the size of tracked fish vary significantly, the sizes of the body rectangles can also be adjusted and they can be determined in detection stage when a fish is initially detected.

### 2.3 Fish detection and pose estimation

One frame of the captured fish school is shown in Fig 2c. The background image Fig 2d is calculated by computing the mean image of 18000 successive frames. After background subtraction, the pixels within fish body area are significantly different from the background pixels, what’s more, the curvature of fish boundary at head/tail positions is significantly greater than the curvature at other positions. Taking these features into consideration, we propose a four-step method to detect fish head and estimate fish pose accurately.

**Fig 3:**
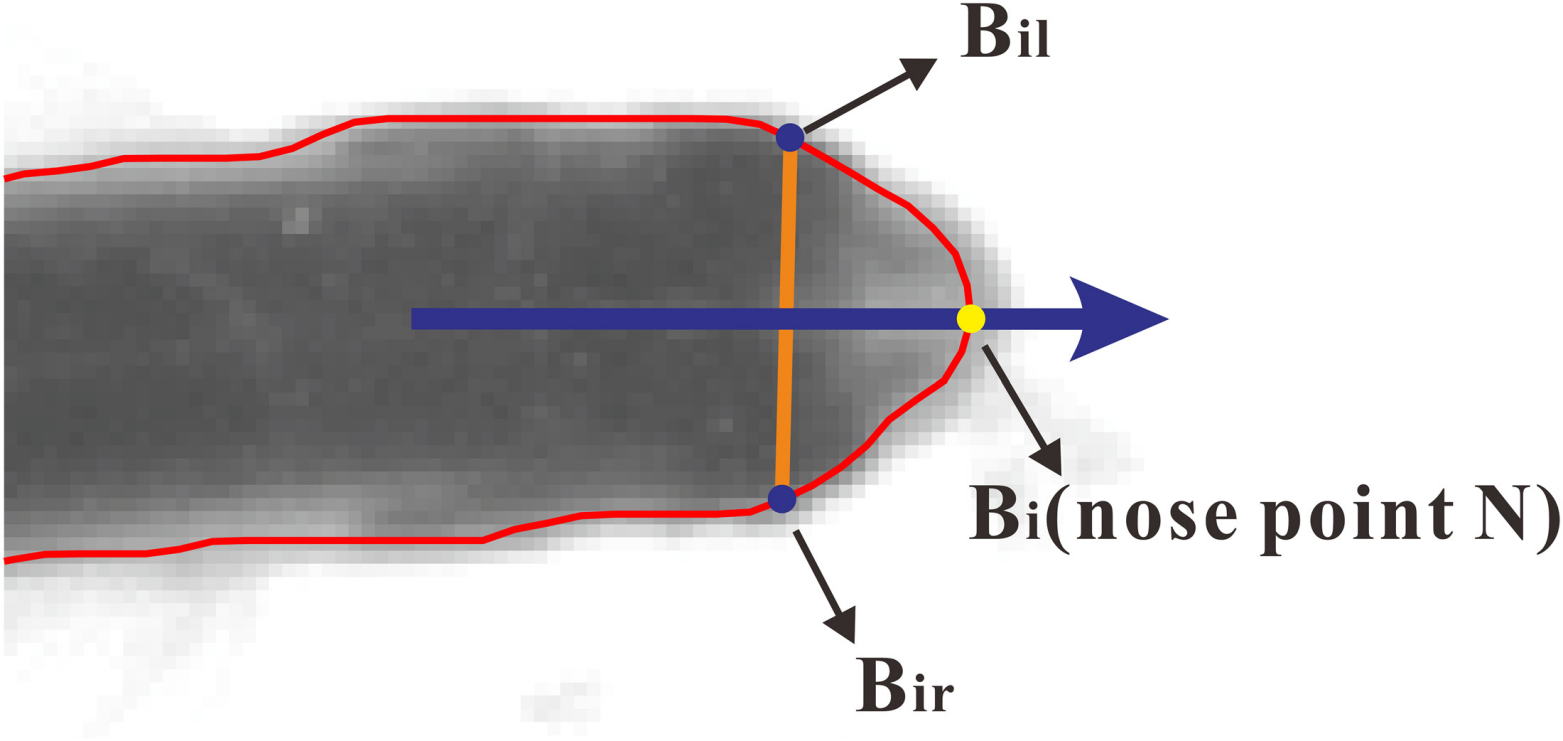
Head orientation computation. *N* (yellow point) is the detected fish nose point, *B*_*il*_ and *B*_*ir*_ (blue points) are points on the left and right side of *N* on the fish boundary 8 unit arc length away. Blue arrow indicates the orientation of head.

**Fig 4:**
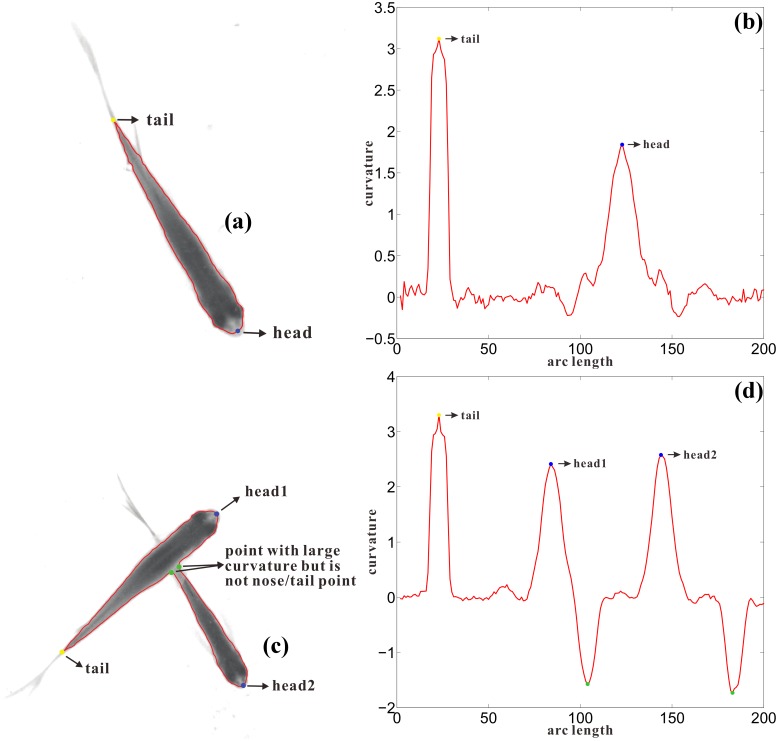
Fish head and tail detection. (a). Sample image of one single fish; (b). Boundary curvature curve of the fish in (a); (c). Sample image of two overlapping fish; (d). Boundary curvature curve of the two fish in (c). It can be seen that the points at intersection corner (such as the green points in (c)) won’t be misjudged as tail or nose points. The two green points in (c) are also plotted in the figure.

1. Fish boundary extraction
  The image after background subtraction is firstly transformed into a binary image using image thresholding method. Then the fish boundary can be obtained by ‘bwboundaries’ function in MATLAB [35]. The boundary points calculated by ‘bwboundaries’ form an ordered point set, then the points are resampled so that the distance between adjacent points is equal. The points after resampling are still ordered, called boundary point set, written as *B*_*i*_ = (*x*_*B*_*i*__, *y*_*B*_*i*__)(*i* = 1,.., *n*_*bw*_), *n*_*bw*_ is the number of points in the set.
2. Curvature computation and nose point detection
  The curvature *κ* at each point on the fish boundary curve is defined as the infinitesimal angle between tangents to that curve at the ends of an infinitesimal segment of the boundary curve to the length of that segment (written as *dφ*/*ds*, *φ* is the tangential angle, *s* is length of the segment). Assuming the boundary curve is parameterized by arc length, *φ* is defined as (cos*φ*, sin*φ*) = (*x*′, *y*′). Curvature *κ* is positive if the curve bends to the left and negative if the curve bends to the right. The boundary curve here is represented by discrete point set *B*_*i*_, so we use an approximation method to calculate curvature. As the resampled points are close enough to each other, the length of arc $\overset{⌢}{AB}$ (written as $l_{\overset{⌢}{AB}}$, *A*, *B* are two adjacent points in the boundary point set) can be estimated by the length of line segment *AB*. The unit tangent at point *B*_*i*_ (written as *φ*(*B*_*i*_)) can be estimated by coordinate of left and right adjacent point of *B*_*i*_. Thus the curvature at each point *B*_*i*_ on fish boundary is approximately calculated by:
  $$\begin{array}{c} \begin{array}{c} C\left(i\right)=\frac{\varphi \left(B_{il}\right)-\varphi \left(B_{ir}\right)}{l_{\overset{⌢}{B_{il}B_{ir}}}}\\ \varphi \left(B_{i}\right)=atan2\left(y_{B_{i+1}}-y_{B_{i-1}},x_{B_{i+1}}-x_{B_{i-1}}\right) \end{array} \end{array}$$ (1)
  in which *atan*2() refers to four-quadrant inverse tangent, *B*_*il*_ and *B*_*ir*_ are point on the left and right side of *B*_*i*_ respectively (as shown in Fig 3) and $l_{\overset{⌢}{B_{il}B_{i}}}$ equals to $l_{\overset{⌢}{B_{i}B_{ir}}}$. Considering that the computed fish boundary is not smooth enough for estimating curvature with two points too close to each other, we do not use the adjacent points of *B*_*i*_ but the left and right neighbors 8 unit arc length away from it, that is, $l_{\overset{⌢}{B_{il}B_{i}}}$ and $l_{\overset{⌢}{B_{i}B_{ir}}}$ equal to 8. The points at intersection corner (such as the green points in Fig 4c) which are not tail or nose points also have relatively larger absolute curvature value but is negative, so they won’t be misjudged as tail or nose points. The resulting curvature curve is shown in Fig 4.
  It can be seen from Fig 4b that the curvature curve has two obvious local maximum values. The lower one corresponds to the nose point (denoted as *N*) and the relative larger one corresponds to tail point (denoted as *O*). Thus by locating the two local maximum points, nose point can be detected.
  When occlusion occurs, the curvature curve may have more than two candidate maxima of nose and tail. In these circumstances, the threshold is still valid for nose detection if the fish head is not occluded. As shown in Fig 4c and 4d, tail of one fish is occluded, but two noses can still be detected.
3. Head orientation computation and head rectangle determination
  The left and right half of zebrafish head are laterally symmetric about their body axis [33]. Thus the head orientation can be determined by nose point *N* and its neighbor points *B*_*il*_ and *B*_*ir*_, as shown in Fig 3. Fish head orientation is defined as the direction of perpendicular bisector of segment *B*_*il*_ *B*_*ir*_. Now we have nose point and head orientation, thus the head rectangle *rec*_1_ of each fish can be uniquely determined.
4. Pose estimation based on rectangle chain fitting
  The final step is to estimate fish pose by fitting the body rectangle chain. As we have no prior information about the pose of each fish before detection, for each body rectangle, we have to generate angles randomly to search for all possible configurations. Firstly, *n*_*pb*_ random angles ranging between [0, 2*π*) are generated. Because the length and width of each body rectangle *rec*_*i*_(*i* = 2, …, *n*_*r*_) are predefined and joint *J*_1_ (as shown in Fig 2a) is determined after head rectangle *rec*_1_ has been fitted, one random angle corresponds to one rectangle. The rectangle that covers the largest area of fish body region in the image is chosen to be *rec*_2_. When the first body rectangle *rec*_2_ is determined, joint *J*_2_ is determined at the same time. Using similar strategy, the remaining rectangles of fish body can be determined in the same way as *rec*_2_. After all the head and body rectangles are determined, the total cover ratio of the 8 rectangles can be calculated. If cover ratio is less than 80% of the fish region, the detection result of this fish is considered to be problematic and it will be removed from the final detection result of this frame. The detection result of a whole image is shown in Fig 2e.

### 2.4 Fish tracking

It is observed as discussed in section 2.2 that the motion of head region in top-view image is almost rigid. It therefore makes sense to firstly track the head region and then track the deformable body part. This two-staged tracking shows good performance in the experiments. The two stages will be introduced respectively.

1. Head tracking
  In most of the time, the 8 rectangles of each fish can be accurately detected in detection stage. And the frame rate of the camera is relatively high (100fps), so fish displacement and body deformation between two consecutive frames are relatively small, and the state variation in several consecutive frames is nearly uniform. The motion of fish can be accurately predicted using simple linear Bayesian filter like Kalman filter [36]. In addition, Kalman filter is more efficient than other algorithms such as particle filter [37]. Hence, in our system, Kalman filter is applied to accomplish the tracking task.
  In our system, the state vector of fish head is composed of 6 variables, i.e., the coordinate of *J*_1_ (as shown in Fig 2a) and orientation of head rectangle at current and previous frame (it will be explained later in section 3.3 why *J*_1_ is used instead of nose point *N* or midpoint *G* on front edge of *rec*_1_). The coordinates of *J*_1_ at frame *t* is denoted as $\left(x_{1}^{t},y_{1}^{t}\right)$, and head orientation is $\theta_{1}^{t}$. So the state vector *X*^*t*^ is defined as [*x*^*t*^, *y*^*t*^, *θ*^*t*^, *x*^*t*−1^, *y*^*t*−1^, *θ*^*t*−1^]^*T*^. The observation vector *Z*^*t*^ is defined as [*x*^*t*^, *y*^*t*^, *θ*^*t*^]^*T*^ (we drop the subscript of *X*^*t*^ and *Z*^*t*^ for ease of notion).
  The state and observation equation in Kalman filter can be described as
  $$\begin{array}{c} \begin{array}{c} X^{t}=FX^{t-1}+\omega^{t}\\ Z^{t}=HX^{t}+\nu^{t} \end{array} \end{array}$$ (2)
  where *F* and *H* are the state transition and observation matrix of the target at time *t* respectively, *ω*^*t*^ and *ν*^*t*^ are the noise of state and observation, both of them are zero-mean Gaussian noise.
  The first step of Kalman filter is to predict the state vector at time *t*. In our case, we assume that in most circumstances, the velocity of fish head is constant, thus the prior estimation of state vector ${\hat{X}}^{t}$ and its error covariance ${\hat{P}}^{t}$ at time *t* can be predicted by:
  $$\begin{array}{c} \begin{array}{c} {\hat{X}}^{t}=FX^{t-1}\\ {\hat{P}}^{t}=FP^{t-1}F^{T}+Q^{t}\\ F=\left[\begin{array}{cccccc} 2 & 0 & 0 & -1 & 0 & 0\\ 0 & 2 & 0 & 0 & -1 & 0\\ 0 & 0 & 2 & 0 & 0 & -1\\ 1 & 0 & 0 & 0 & 0 & 0\\ 0 & 1 & 0 & 0 & 0 & 0\\ 0 & 0 & 1 & 0 & 0 & 0 \end{array}\right] \end{array} \end{array}$$ (3)
  where *Q*^*t*^ is the covariance matrix of state noise *ω*^*t*^.
  The second step is data association aiming at associating each tracker with each measurement at current frame. Data association should follow the one to one criterion, which means that one tracker should be associated with at most one measurement and each measurement should be associated with at most one tracker. In our system, we formulate the data association task as a global optimization problem and employ Kuhn-Munkres algorithm to calculate a global optimum solution [38]. The cost matrix *C*(*i*, *j*) represents the cost of each tracker *i* being associated with each measurement *j*, defined as:
  $$\begin{array}{c} C(i,j)=\exp(-(NCC(i,j)+V(i,j)))\;\;\;(i=1,...,n,\;j=1,...,m) \end{array}$$ (4)
  the objective function is:
  $$\begin{array}{c} \tau =\arg\min_{A}\sum_{i=1}^{n}\sum_{j=1}^{m}C\left(i,j\right)*A\left(i,j\right) \end{array}$$ (5)
  subject to:
  $$\begin{array}{c} \sum_{i=1}^{n}A\left(i,j\right)=1\;\;\;and\;\;\;\sum_{j=1}^{m}A\left(i,j\right)=1 \end{array}$$ (6)
  where *NCC*(*i*, *j*) is normalized-cross-correlation (NCC) between the head rectangle image patches of predicted tracker *i* and measurement *j* [39]. We choose NCC to measure image similarity between predicted tracker *i* and measurement *j* because NCC is robust under illustration change and slight partial occlusion, and it is widely used in existing tracking systems [18]. NCC of head rectangle image patches of predicted tracker *i* (denoted as *I*) and measurement *j* (denoted as *I*′) after rotating to horizontal position is calculated as:
  $$\begin{array}{c} NCC\left(i,j\right)=\frac{\sum_{dy=0}^{h-1}\sum_{dx=0}^{w-1}I\left(dx,dy\right)I^{\prime }\left(dx,dy\right)}{\sqrt{{\left(\sum_{dy=0}^{h-1}\sum_{dx=0}^{w-1}I\left(dx,dy\right)\right)}^{2}{\left(\sum_{dy=0}^{h-1}\sum_{dx=0}^{w-1}I^{\prime }\left(dx,dy\right)\right)}^{2}}} \end{array}$$ (7)
  *h*, *w* is height and width of head rectangle image patch respectively. *V*(*i*, *j*) in Eq 4 measures the orientation difference between predicted head rectangle and measurement’s, which is measured by von Mises distribution [40] and is calculated as:
  $$\begin{array}{c} V\left(i,j\right)=\frac{\exp(k\cos\left(y-\mu \right))}{2\pi I_{0}\left(k\right)} \end{array}$$ (8)
  where *I*_0_(*k*) is modified Bessel function of order 0. In our experiment, *k* is set to 4, *μ* is set to the head orientation of measurement *j* at frame *t*. If *NCC*(*i*, *j*) is smaller than threshold *thr*_*ncc*_ or *V*(*i*, *j*) is smaller than threshold *thr*_*v*_, then *C*(*i*, *j*) will be set to *Inf*, meaning that tracker *i* is impossible to be associated with measurement *j*. If *n* ≠ *m*, then dummy nodes are added and the cost will be set to *Inf* to guarantee that no node will be associated with them.
  After the above procedures, the head rectangle *rec*_1_ of each fish at current frame is detected and tracked. Three situations may occur:
  - Each tracker is associated with exactly one measurement.
  - Some trackers are not associated with any measurements. In this case the tracker is considered to be losing its target, then the state vector is updated using state of the previous two consecutive frames as:
    $$\begin{array}{c} \left\{\begin{array}{c} x^{t}=2x^{t-1}-x^{t-2}\\ y^{t}=2y^{t-1}-y^{t-2}\\ \theta^{t}=2\theta^{t-1}-\theta^{t-2} \end{array}\right. \end{array}$$ (9)
    If the length of the trajectory up to current frame is less than 2, then the target will be regarded as a tracking error, the tracker will be terminated and removed from the final tracking results. If one tracker has been losing its target for longer than 5 frames, then the tracker will be terminated.
  - Some measurements are not associated with any trackers. This happens when occlusion ends, some fish are successfully detected again, but the corresponding trackers before occlusion have been terminated. We regard the unassociated measurements as newly emerging objects and initialize a new tracker for each of them. The interrupted trajectories will be relinked in tracklets relinking stage.
  When data association finishes, the state vector and error covariance matrix are updated by:
  $$\begin{array}{c} \begin{array}{c} X^{t}={\hat{X}}^{t}+K^{t}\left({\hat{Z}}^{t}-H{\hat{X}}^{t}\right)\\ P^{t}=\left(I-K^{t}H\right){\hat{P}}^{t}\\ H=\left[\begin{array}{cccccc} 1 & 0 & 0 & 0 & 0 & 0\\ 0 & 1 & 0 & 0 & 0 & 0\\ 0 & 0 & 1 & 0 & 0 & 0 \end{array}\right] \end{array} \end{array}$$ (10)
  in which *K*^*t*^ is Kalman gain at time *t*, calculated as:
  $$\begin{array}{c} K^{t}={\hat{P}}^{t}H^{T}{\left(H{\hat{P}}^{t}H^{T}+R\right)}^{-1} \end{array}$$ (11)
  *R* is the covariance matrix of observation noise *ν*^*t*^.
2. Body pose tracking
  When the head tracking stage is finished, the head location and orientation of most fish can be successfully estimated. Body rectangle chain fitting is thus relatively easy because the joint *J*_1_ has been determined. Body tracking stage can on the other hand help verify whether the result of head tracking stage is correct.
  Assuming the coordinate of *J*_1_ obtained in head tracking stage is (*x*^*t*^, *y*^*t*^), then, *n*_*pb*_ random angles are generated by von Mises distribution (the mean value *μ* is set as the orientation of *rec*_2_ in the last frame). For each random angle, a rectangle is reconstructed. The one which covers the largest fish body area is chosen, the corresponding orientation is orientation of *rec*_2_ at current frame. After orientation of *rec*_2_ is determined, the remaining rectangles *rec*_3_-*rec*_8_ can be determined as *rec*_2_.
  After all the body rectangles are determined, the total cover ratio of the 8 rectangles (including head rectangle) is calculated as in detection stage. If cover ratio is less than 80% of the fish region, the tracking result of this fish at this frame is considered to be problematic and the tracker will be terminated.
  By applying this strategy, some tracking errors can be eliminated. Unfortunately, some trajectories may be split, so we propose a trajectory relinking stage to reconnect the interrupted trajectories.

### 2.5 Tracklets relinking

After the above stages, we have obtained 2D trajectory of each fish in the fish school. However, due to occlusion and detection error, the 2D trajectories of some fish may be fragmented into several tracklets. So a trajectory relinking stage is required to obtain complete trajectories for the fish.

Tracklets relinking can be formulated as a linear assignment problem. Several existing 2D tracking systems employed Kuhn-Munkres algorithm to relink the trajectories [38, 41], which is a combinatorial optimization algorithm that solves the assignment problem in polynomial time and can guarantee that the resultant trajectories are globally optimal according to a given objective function. However, the number of resulted trajectory which is an important prior cannot be specified and taken advantage of in their systems. So in our tracking system, we formulate the relinking problem as a minimum cost maximum flow (MCMF) problem, in which the total flow can be specified so that the number of relinked trajectories can be controlled. In this way, relinking errors can be reduced.

**Fig 5:**
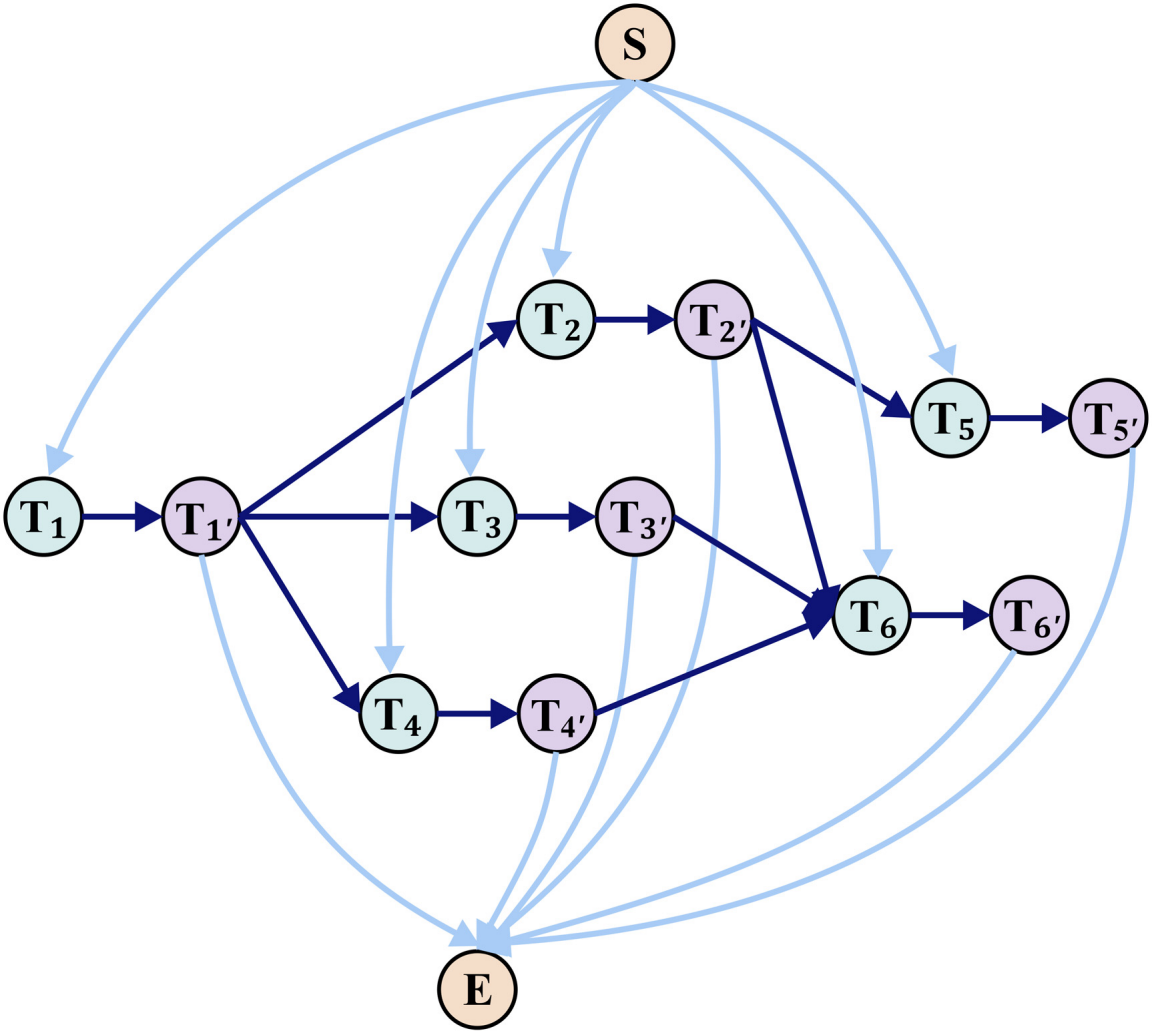
MCMF graph. Node *S* and *E* are source and sink node respectively. For each tracklet Γ_*i*_, two nodes *T*_*i*_ and *T*_*i*′_ are added in the graph. There are directed edges from *S* to each *T*_*i*_, from each *T*_*i*′_ to *E*, and from each *T*_*i*_ to each *T*_*i*′_. For tracklets Γ_*i*_ and Γ_*j*_ that may be trajectory of identical fish, one directed edge form *T*_*i*_ to *T*_*j*_ is added.

In an MCMF problem, the objective is to minimize the total cost of a directed weighted network, while the total flow is maximized or equals to a predefined value. In the trajectory relinking case, the flow should be binarized, which means that the capacity of each edge in the graph should be restricted to either 0 or 1, Fig 5 shows one sample of the MCMF graph.

For each tracklet (marked as Γ_*i*_), we set two nodes in the graph (namely *T*_*i*_ and *T*_*i*′_ in Fig 5) and there is one edge starting from the source *S* to node *T*_*i*_, and another edge starting from *T*_*i*′_ to the sink *E*. Let *cst*(*i*, *j*) and *cap*(*i*, *j*) denote the cost value and flow capacity of each edge in the graph respectively, which are defined as:

$$\begin{array}{c} \begin{array}{c} cap(S,i)=1\\ cap(i^{\prime },E)=1\\ cap(i,i^{\prime })=1\\ cap(i^{\prime },j)=1,\;\;\;if\;\;trajectory\;\;i\;\;and\;\;j\;\;satisfy\;\;Eq13 \end{array} \end{array}$$ (12)

$$\begin{array}{c} \begin{array}{c} st_{j}-ed_{i}>0\\ st_{j}-ed_{i}<max_{interf}\\ D\left(i,j\right)<max_{interd} \end{array} \end{array}$$ (13)

where *st*_*i*_ and *ed*_*i*_ are the start frame and end frame of tracker *i* respectively, *D*(*i*, *j*) is the Euclid distance between point *J*_1_ in the last frame of tracker *i* and the first frame of tracker *j*. In our experiment, *max*_*interf*_ is set to 6 and *max*_*interd*_ is set to 80.

$$\begin{array}{c} \begin{array}{c} cst(S,i)=0\\ cst(i^{\prime },E)=0\\ cst(i,i^{\prime })=0\\ cst\left(i^{\prime },j\right)=\exp\left(D\left(i,j\right)\right)*\exp\left(-V\left(i,j\right)\right),\;\;\;if\;\;cap\left(i^{\prime },j\right)=1 \end{array} \end{array}$$ (14)

where *V*(*i*, *j*) is the same as Eq 8 which measures the similarity of two angles. *k* is still set to 4 and here *μ* is defined as the head orientation at the point of tracklet *i*. In our system, the information of body rectangles is not used in tracklets relinking, the reason is that after a few frames, the body geometry may greatly change, which is not robust enough for tracklets relinking.

After building the directed weighted graph, we enumerate the possible total flow of the graph and generalized Ford-Fulkerson algorithm [42] is applied to solve the MCMF problem, the time complexity of the algorithm is *O*(*kfnm*), *n*, *m* is the number of nodes and edges in the graph respectively, *f* is the value of flow, *k* is the number of enumerated flow.

## Experiments and Discussions

### 3.1 Materials and setup

**Fig 6:**
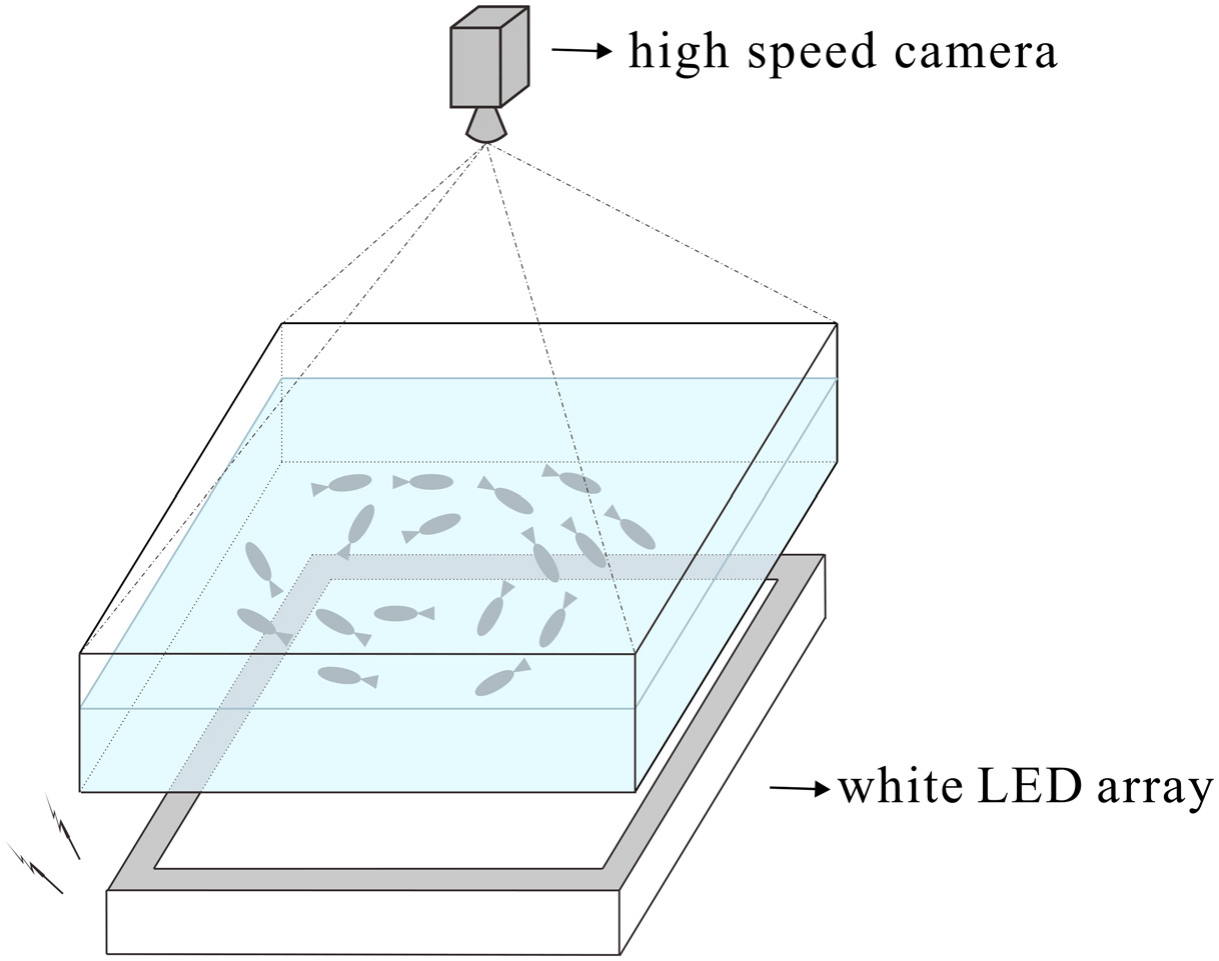
Experiment setup. The zebrafish school swim in a transparent acrylic tank horizontally placed above a white LED array covered by a diffusion panel. One high speed camera is mounted above the tank.

In order to evaluate the performance of the proposed tracking system, we captured two videos of zebrafish (*Danio rerio*) school with different group sizes (10 and 20 fish respectively). The zebrafish swim in a 20*cm* × 20*cm* × 20*cm* transparent acrylic tank. The four walls of the tank are pasted with white paper to prevent mirror effect that may affect fish behavior and tracking system. The tank was horizontally placed above a planar light source made up of a white LED array covered by a diffusion panel. The light source is placed at bottom of the water tank because in this way the camera captures backlit images, thus the boundary of the objects (fish) is clearer, and the object body is darker, without too much texture features, which facilitates the tracking task. One high speed camera (IO Industries Canada, Flare 4M 180-CL, 2048v×2040h pixels at 100fps) is mounted about 40*cm* above the tank, the imaging plane is almost parallel to the water surface. The experiment setup is shown in Fig 6. The captured videos are firstly stored in DVR Express (IO Industries Canada, DVR Express^®^ Core Camera Link Full, monochrome, 10×8 bit, Full, 1TB) when the experiment is in process and are then exported as bmp format images to a PC after the experiment is finished.

### 3.2 Evaluation of the proposed system

**Fig 7:**
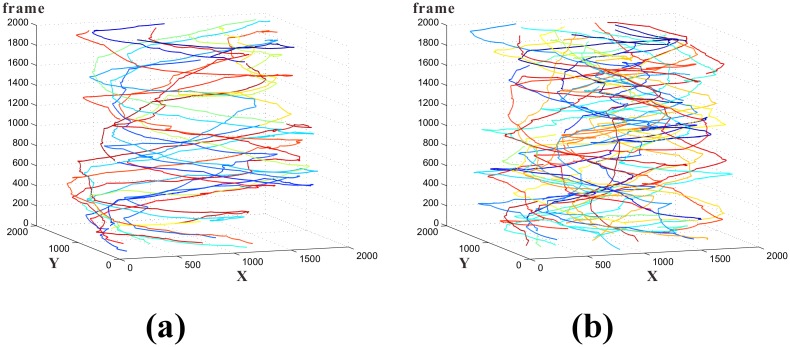
Tracking results. Z-axis represents the frame number, X-axis and Y-axis are coordinates of the image plane. Different colors indicate different individuals. (a). Tracking results of 10 fish for 2000 frames; (b). Tracking results of 20 fish for 2000 frames.

In this subsection we present the tracking results on two data sets (written as D1 and D2), each data set is a video clip that contains 2000 frames in total. The size of fish school in the two data sets are 10 and 20 respectively. Fig 7a shows the tracking result of the 10-fish group. Fig 7b shows the tracking result of the 20-fish group.

We have quantitatively evaluated the detection and tracking performance of the proposed system.

**Fig 8:**
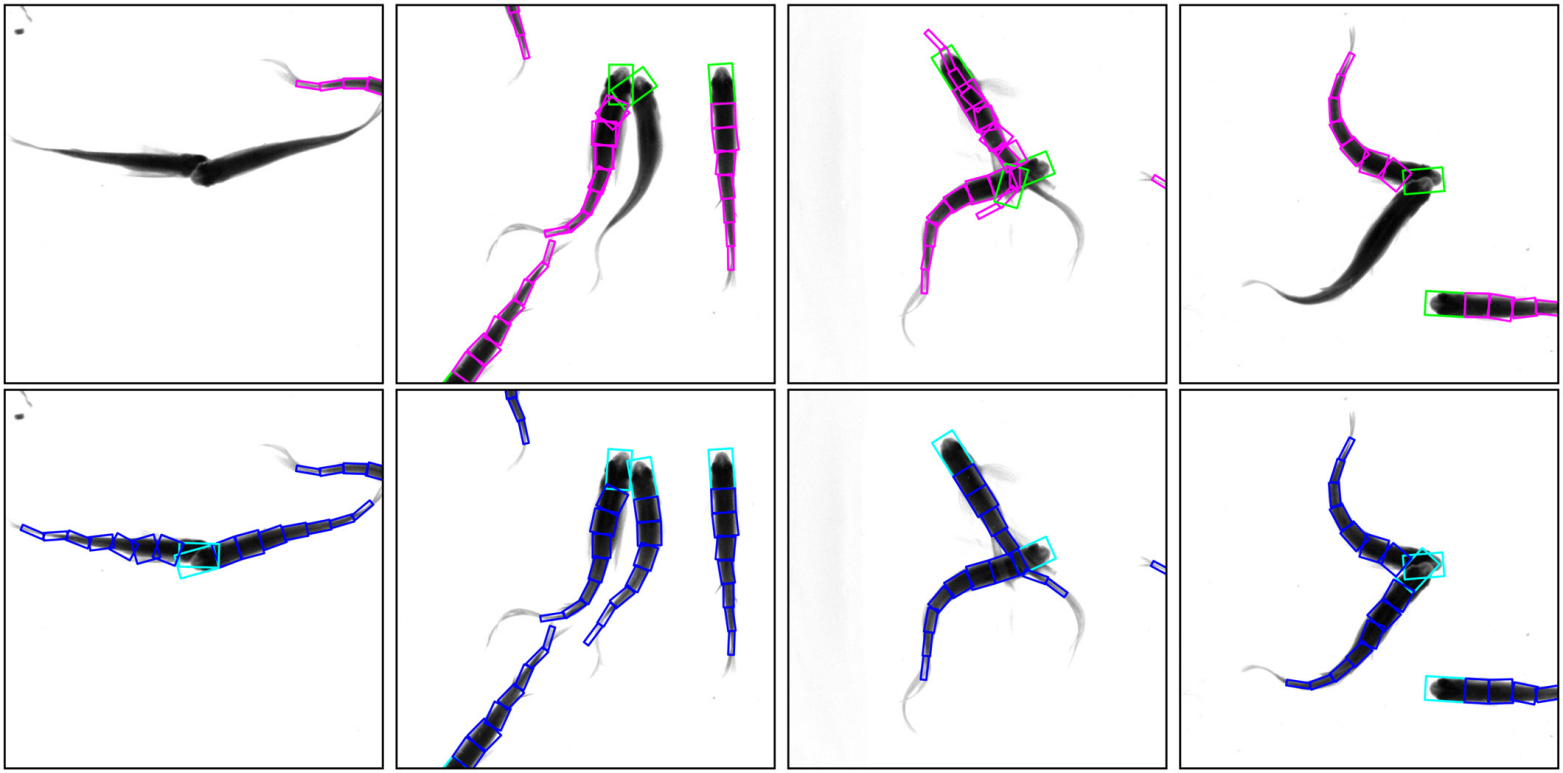
Examples of successful correction of detection failure. Images in the first row present four examples of detection failures including detection error, detection missing and duplication detection. The failures are successfully corrected in tracking stage, see images in the second row.

1. Performance of detection
  We selected 300 frames (frame No.1–300) from each of the two original videos (named DS1 and DS2 respectively) and manually annotated the nose point and correct identity of each fish (when occlusion occurs but the nose point can be recognized, it will also be annotated). The body fitting performance is judged by human eyes, because we have removed the fitting results whose cover ratio is lower than 80%, the possible fitting failure is mostly caused by occlusion, body rectangles being wrongly fitted onto another fish, which can be easily judged by human eye. The performance evaluation of detection is all based on the 300 annotated frames.
  Miss ratio and error ratio are applied to evaluate the performance of detection stage, which are calculated as:
  $$\begin{array}{c} Missratio=\frac{total\;number\;of\;undetected\;fish\;in\;all\;frames}{number\;of\;fish\;*\;number\;of\;frames}\\ Error\;ratio=\frac{total\;number\;of\;wrongly\;detected\;fish\;in\;all\;frames}{number\;of\;fish\;*\;number\;of\;frames} \end{array}$$ (15)
  The correct detection of a fish is defined as: (1) the 8 rectangles cover over 80% of the fish body area in the image after background subtraction (this has been checked in detection stage). (2) the distance between the detected fish nose and annotated groundtruth is less than 10 pixels (the width of fish head is about 35 pixels). (3) the fish body is correctly fitted (checked manually).
  To quantitatively investigate the influence of occlusion on detection performance, we counted the number of occlusions in video clips DS1 and DS2. When the fish bodies of two or more fish overlap, then we say one occlusion is detected, which also means that in one frame, there may be more than one occlusion. We calculated the proportion of miss caused by occlusion and proportion of error caused by occlusion respectively. The evaluation results are listed in Table 2.
  We can conclude from the result that when the density of fish group doubles, the number of occlusions increases dramatically, which is consistent with the content in [25]. Nearly two occlusions occur in each frame on average. Both miss ratio and error ratio increase a lot, which illustrates that density of fish group significantly affects the performance of detection stage. Accordingly, it is essential to consider about occlusion in tracking stage such as implementing tracklets relinking in our tracking system.
  To test the performance of fish body fitting, we calculated the proportion of detection errors due to incorrect fitting and proportion of incorrect fitting due to occlusion. The results are shown in Table 3.
  According to the results, we may conclude that when the occlusion frequency increases, greater percentage of detection errors are due to incorrect fitting. In those cases that fish head is not occluded but fish body is, the heads can still be correctly detected while the body rectangles may be fitted onto another fish. Moreover, nearly all fitting failures are caused by occlusion (in our experiment the ratio is nearly 100%). That is to say, if the fish body is not occluded, the body fitting accuracy of the proposed body fitting method is almost 100%. These fitting failures can be solved later in tracking stage.
2. Performance of tracking
  The tracking performance of the proposed system is compared with other two methods. One is the proposed system without body fitting. That means, only use the head detector and head tracking of the proposed system. The aim of comparing with this method is to verify the effectiveness of body fitting in judging the head tracking results and removing the incorrect ones. The other one is a recently proposed open source 2D tracking system: idTracker [25]. idTracker is one of the ‘fingerprinting’ based tracking system [22] which uses a set of traits to recognize each tracked individual, the advantage of it is that it can identify each individual even after severe occlusion and it can be applied to track different objects, but when the density of objects is higher, identification errors may occur. For animal behavior research, correctness of the identification is very important [25]. Considering this, the aim of our tracking system is firstly to ensure the correctness of the tracking results, integrity of the trajectories is in second place. In the tracklets relinking stage, we chose more stringent threshold so that only those trajectories without ambiguities are finally relinked. In evaluation of tracking performance, Correct Tracking Ratio (CTR) is analyzed based on the groundtruth image annotated manually (DS1 and DS2), Running time, Average Interruption Times (AIT) and Correct Identification Ratio (CIR) are analyzed based on the whole data set D1 and D2.
  Firstly, we tested the running time of the proposed system and idTracker on the two whole data sets D1 and D2. Both the proposed system and idtracker are implemented with MATLAB^™^. Each of the two video clips contains 2000 frames with resolution equals to 2048 × 2040, frame rate equals to 100fps. The computer hardware includes a quad-core Intel Core i5-2500, 3.30GHz CPU, 8GB RAM. To run idTracker, we compressed the two video clips to 2% of the original file size. For the proposed method, we used the original video to guarantee the high accuracy of fish body fitting. The results are shown in Table 4.
  Accoding to the results, the proposed system without body fitting requires less running time than idTracker, and much more time (more than 95%) is spent on fish body fitting.
  To evaluate the performance of the tracking stage, we use the following three indices to measure the tracking performance.
  - Correct Tracking Ratio (CTR)
    CTR describes the percentage of correctly tracked frames of a single fish that calaulated as:
    $$\begin{array}{c} CTR=\frac{\Sigma (number\;of\;correct\;frames\;of\;a\;single\;fish)}{number\;of\;fish\;*\;number\;of\;frames} \end{array}$$ (16)
    For the proposed system, we calculated CTR before and after tracklets relinking (trajectories after tracklets relinking are also called final result). The correct tracking of a fish is defined similar to correct detection: (1) the 8 rectangles cover over 80% of the fish body area in the image after background subtraction (this has been checked in tracking stage); (2) the distance between the tracked fish nose and annotated groundtruth is less than 10 pixels. To further evaluate the performance of fish body fitting, we tested the accuracy of body fitting after tracklets relinking, which is checked manually. For the compared idTracker, CTR before and after tracklets relinking refers to the raw output trajectories and trajectories that contain estimated positions of the individuals during occlusions (which contains fewer gaps) respectively. The correct tracking of idTracker is defined as: the tracking result (fish positions) correctly falls on the fish body in the image. The detailed comparison of the three methods is shown in Table 5.
    It can be seen from the results that CTR of the proposed system outperformed idTracker when fish density is higher, mainly because detection in idTracker is based on blob detection, when occlusion occurs frequently, the detector will fail and sometimes relinking is difficult. Our system is based on a fish head detector, when occlusion occurs, fish head can still be detected if the head part is not occluded. When the proposed system is used without body fitting, CTR would drop a little, because without verifying head tracking results in body fitting stage, some tracking errors in head tracking remain. It can also be seen that tracklets relinking further improved the tracking performance and the accuracy of body shape is higher than 99%.
  - Average Interruption Times (AIT)
    AIT measures how many times that the trajectory of a single fish interrupt on average per 100 frames, calculated as:
    $$\begin{array}{c} AIT=\frac{\Sigma (trajectory\;interruption\;times\;of\;each\;fish)}{number\;of\;fish\;*\;number\;of\;frames\;/\;100} \end{array}$$ (17)
    The AIT as well as the proportion of trajectory interruption caused by occlusion of the three methods are shown in Table 6. Note that the proposed system is not identification remaining, it means that after trajectory interruption, the trajectory in the next frames will be labeled as a new object, which is different from ‘fingerprinting’ based tracking systems such as idTracker.
    From the result it can be seen that tracklets relinking stage effectively improves the integrity of the trajectories. After tracklets relinking, AIT reduces nearly 90%, the trajectory continuity of the proposed system is better than that of the proposed system without body fitting. It also outperforms idTracker when the fish group density is doubled. For the proposed system, when the frequency of occlusion increases, a higher percentage of trajectory interruption is caused by occlusion.
  - Correct Identification Ratio (CIR)
    CIR represents the probability of correct identification of all fish after an occlusion event, calculated as:
    $$\begin{array}{c} CIR=\frac{times\;that\;all\;fish\;get\;correct\;identity\;after\;occlusion}{number\;of\;occlusion\;events} \end{array}$$ (18)
    the comparison of CIR between the proposed system and the two compared methods is shown in Table 7.
    The result shows that idTracker is capable of correctly recognizing the identification of a small number of objects (10 fish), even when trajectory interruption occurs, the identity of each individual can be preserved after interruption, which outperforms the other two systems. However, when the group density is higher (for example, 20 fish), identification errors may occur, which is a limitation of ‘fingerprinting’ based tracking systems at present. It also shows that body fitting does little help to increase CIR because the information of body rectangles are not used in tracklets relinking in current system.
3. The dependence of tracking performance on detection result
  The proposed tracking system applies Kalman filter to accomplish the tracking task which requires detection before tracking. To investigate to what extent the tracking results depend on the detection performance, we calculated the probability of correct tracking when detection is wrong or missing. The evaluation is based on the manually annotated data set DS1 and DS2. The detailed results are shown in Table 8. Four examples of successful correction of detection failures in tracking stage are shown in Fig 8.
  It can be concluded that no matter whether body fitting is performed, the correctness of tracking result is more dependent on performance of detection when the group density of fish is higher. When the density of fish is low, most incorrect detection can be corrected in tracking stage, but when the density of fish group increases (frequency of occlusion increases), only a small part of detection failures can be corrected. The reason why the correction performance of the proposed system without body fitting is even a little better than the system with body fitting is that: for system with body fitting, when calculating correctness of detection and tracking, correctness of body fitting is also taken into consideration, which to some extent affects the results.

**Table 2:** Evaluation of detection performance.

Data Set	# of occlusions per image	Miss ratio (%)	Miss due to occlusion (%)	Error ratio (%)	Error due to occlusion (%)
DS1	0.13	2.53	45.95	0.93	50.00
DS2	1.93	4.98	94.79	1.13	84.62

**Table 3:** Evaluation of fish body fitting.

Data Set	Proportion of detection errors due to incorrect fitting(%)	Proportion of incorrect fitting due to occlusion(%)
DS1	46.67	100.00
DS2	88.14	100.00

**Table 4:** Running time of the proposed system and idTracker.

Method		Data Set	Running time (/s)
The proposed system	without body fitting	D1	853.1
		D2	1463.4
	with body fitting	D1	19092.5
		D2	34482.5
idTracker		D1	1294.9
		D2	1521.7

**Table 5:** CTR of the proposed system and idTracker.

Method		Data Set	CTR (before relinking)	CTR (final result)	accuracy of body fitting(%)
The proposed system	without body fitting	DS1	96.67	98.97	—
		DS2	95.23	96.45	—
	with body fitting	DS1	98.93	99.17	99.81
		DS2	96.30	97.18	99.12
idTracker		DS1	96.75	99.87	—
		DS2	82.46	90.11	—

**Table 6:** AIT of the proposed system and idTracker.

Method		Data Set	AIT (before relinking)	AIT (final result)	interruption due to occlusion(%)
The proposed system	without body fitting	D1	1.43	0.33	12.96
		D2	1.37	0.48	96.43
	with body fitting	D1	1.42	0.16	66.67
		D2	1.36	0.17	98.36
idTracker		D1	0.22	0.02	100.00
		D2	0.46	0.21	93.10

**Table 7:** CIR of the proposed system and idTracker.

Method		Data Set	CIR(%)
The proposed system	without body fitting	D1	88.89
		D2	84.62
	with body fitting	D1	93.33
		D2	87.23
idTracker		D1	100.00
		D2	87.10

**Table 8:** Proportion of correct tracking with incorrect detection.

Method		Data Set	Proportion of correct tracking with incorrect detection(%)
The proposed system	without body fitting	DS1	77.78
		DS2	28.26
	with body fitting	DS1	70.97
		DS2	28.14

### 3.3 Discussions

According to our experiment, we found that zebrafish may change their body shape from straight to bent and then back to straight again in less than 0.2s, we set the frame rate to 100 fps so that we can track the changing process of fish body shape. Much higher frame rate is not necessary for our study, moreover, higher frame rate such as 3000fps will lead to poor efficiency for long time tracking.

From the detection results we find that when occlusion occurs, as long as the fish head is not occluded, it can still be successfully detected by fish head detector of the proposed tracking system, which outperforms the tracking systems based on blob detector. When there is substantial increase of occlusion frequency, more detection failures of head detection and fish body fitting occur due to occlusion.

There is practically no possibility that a tracker would be assigned to a non-fish object. The reason is that the water tank is uniformly illuminated, after background subtraction the fish bodies are extracted from image neatly, thus, there is no chance that a non-fish object remaining in the subtracted image. And we apply Kuhn-Munkres algorithm to perform data association which could guarantee that it is a one to one association, there is no chance that a fish is assigned to two trackers either.

It is verified by the experiment results that the accuracy of body rectangle fitting is over 99%, which provides valuable body geometry data for biologists and physicists to investigate biological characteristics and hydrodynamics of fish swimming. When severe occlusion occurs and detection fails for consecutive 5 frames, the trajectory may be interrupted, this problem is solve by tracklets relinking, after this postprocess, nearly 90% interrupted trajectories can be successfully reconnected. When two fish overlap and are too close to each other, fish body rectangles may be wrongly fitted onto another fish in detection stage, however, using motion continuity and tracking results of the previous frames, part of the wrongly fitted bodies can be corrected. The tracking system outperforms the proposed system itself without body fitting in CTR and AIT in all data sets, and when fish density is higher, the proposed system outperforms idTracker in terms of both CTR and AIT.

**Fig 9:**
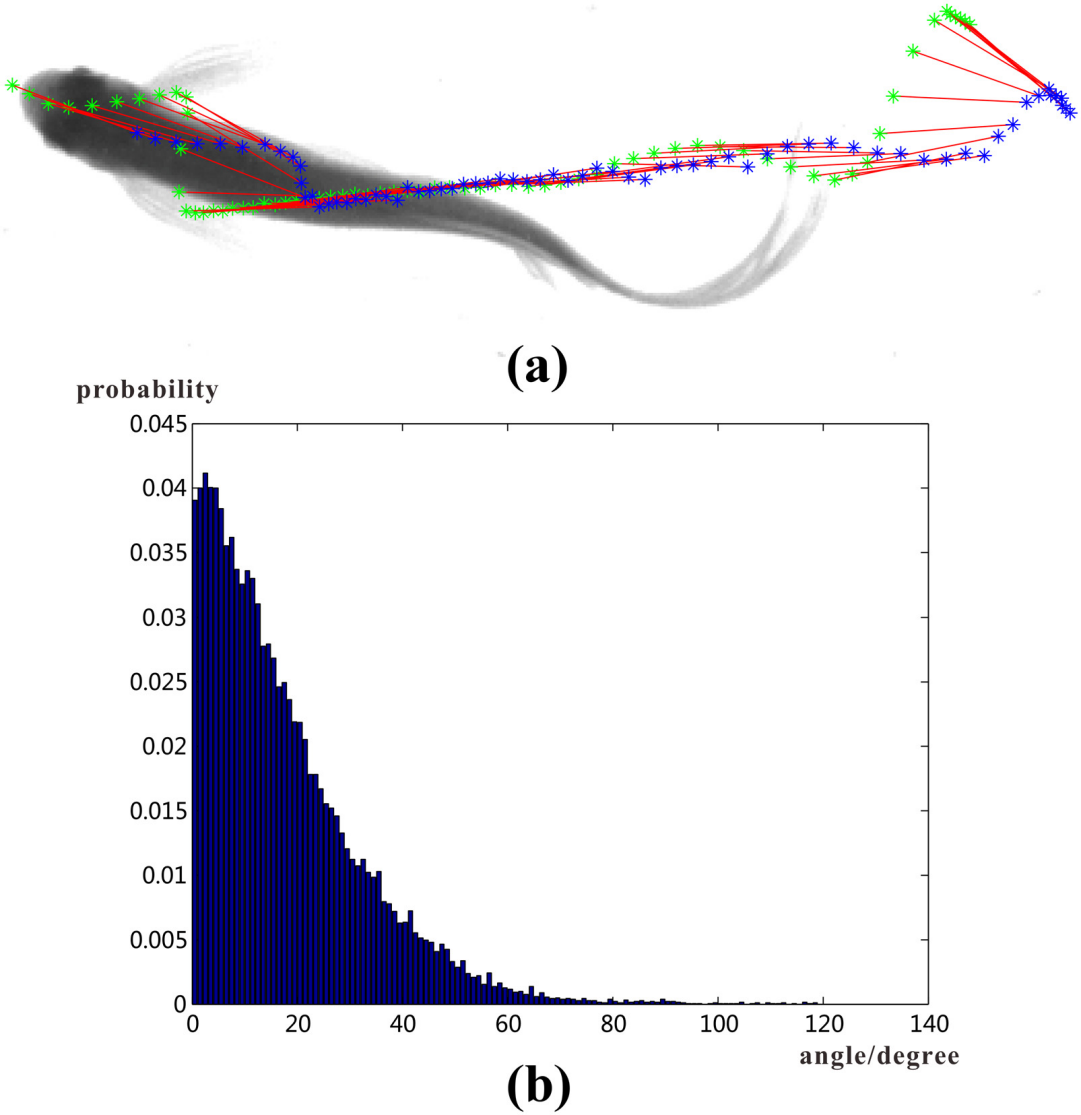
Discussion of tracking result. (a). Trajectory of a fish’s nose point and *J*_1_. Green dots plot the trajectory of nose point *N* in consecutive frames, blue dots plot the trajectory of *J*_1_; (b). Angle difference distribution of fish velocity direction and its head orientation. X-axis is the angle interval in degree, Y-axis is the possibility of angle between fish head orientation and its velocity direction dropping in the angle interval.

In the tracking system, joint *J*_1_ is applied to represent location of the fish head. Fig 9a shows the trajectory of joint *J*_1_ and nose point *N* of one fish in consecutive frames, it can be observed from the figure that the trajectory of *J*_1_ is smoother, which makes it easier to predict new state in Kalman filter. So using coordinates of joint *J*_1_ in tracking stage helps to improve the performance of the tracking system.

The limitations of the proposed tracking system lie in: when the frame rate is low, state prediction and data association is challenging, which may result in more trajectory interruptions and higher possibility of incorrect identification. And the resolution of the video should be guaranteed or there may be problems in head detection and body fitting. In tracklets relinking, only information of head rectangle of each fish is used, which may be not enough for effective relinking.

The relationship between fish velocity direction and head orientation is also analyzed. The statistics show that the fish velocity direction (the velocity is defined as displacement of center of head rectangle *rec*_1_ in two consecutive frames) and its head orientation (the orientation of head rectangle) is inconsistent (see Fig 9b), the probability of the angle difference larger than 10 degree is about 15.93%, when the direction of the fish changes abruptly, the difference will be even larger. So fish velocity direction instead of head orientation should be used in behavior analysis because the former one describes the velocity direction of fish motion, while the latter one is only the orientation of fish head at some moment.

## Conclusion

We have proposed in this paper a tracking system capable of tracking a group of zebrafish swimming in shallow water. A novel fish model is proposed to represent the fish body. In detection stage the location and orientation of each fish head is accurately detected via boundary curvature when the fish head is not occluded, and then the fish body is fitted by linked rectangles. The tracking stage is done by firstly tracking fish head using Kalman filter, then fitting the body part and judging correctness of head tracking. Experiment results show that this system is capable of tracking a zebrafish group with frequent occlusions. The system can also be applied to tracking other species of fish with similar appearance.

With the detailed data of fish body geometry obtained by the proposed tracking system, more research on zebrafish behavior associated with fish body can be accomplished. We performed some analysis on the difference of head orientation and the velocity direction of zebrafish, the result showed that direction of fish velocity should be used in behavior statistical analysis instead of head orientation.

## Supporting Information

S1 Video Tracking result of a group of 10 zebrafish. The left image shows the tracking result of the whole fish group, while the right one focuses on the area of a single fish.
(MP4)

S1 File Source code. Source code of the proposed tracking system.
(ZIP)
