# Supplementary figures and images for: Automated Planar Tracking the Waving Bodies of Multiple Zebrafish Swimming in Shallow Water (part 2 of 2)
Source: PLoS One. 2016 Apr 29;11(4):e0154714. doi: 10.1371/journal.pone.0154714 (PMC4851353; doi:10.1371/journal.pone.0154714)

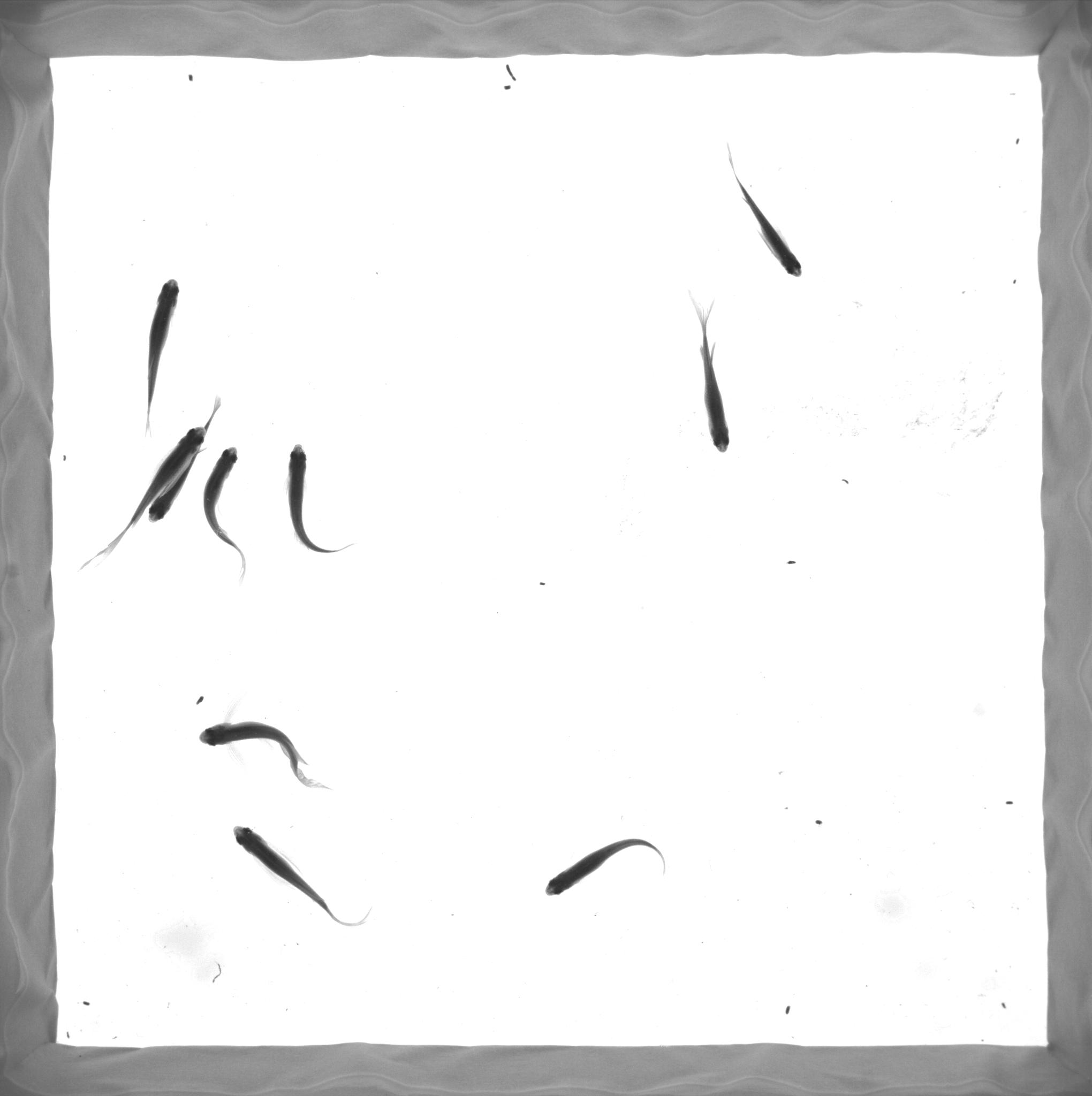

Supplement: S1 File — Source code of the proposed tracking system. (ZIP) [file pone.0154714.s002.zip › code_final/images/CoreView_275_Master_Camera_00099.jpg]

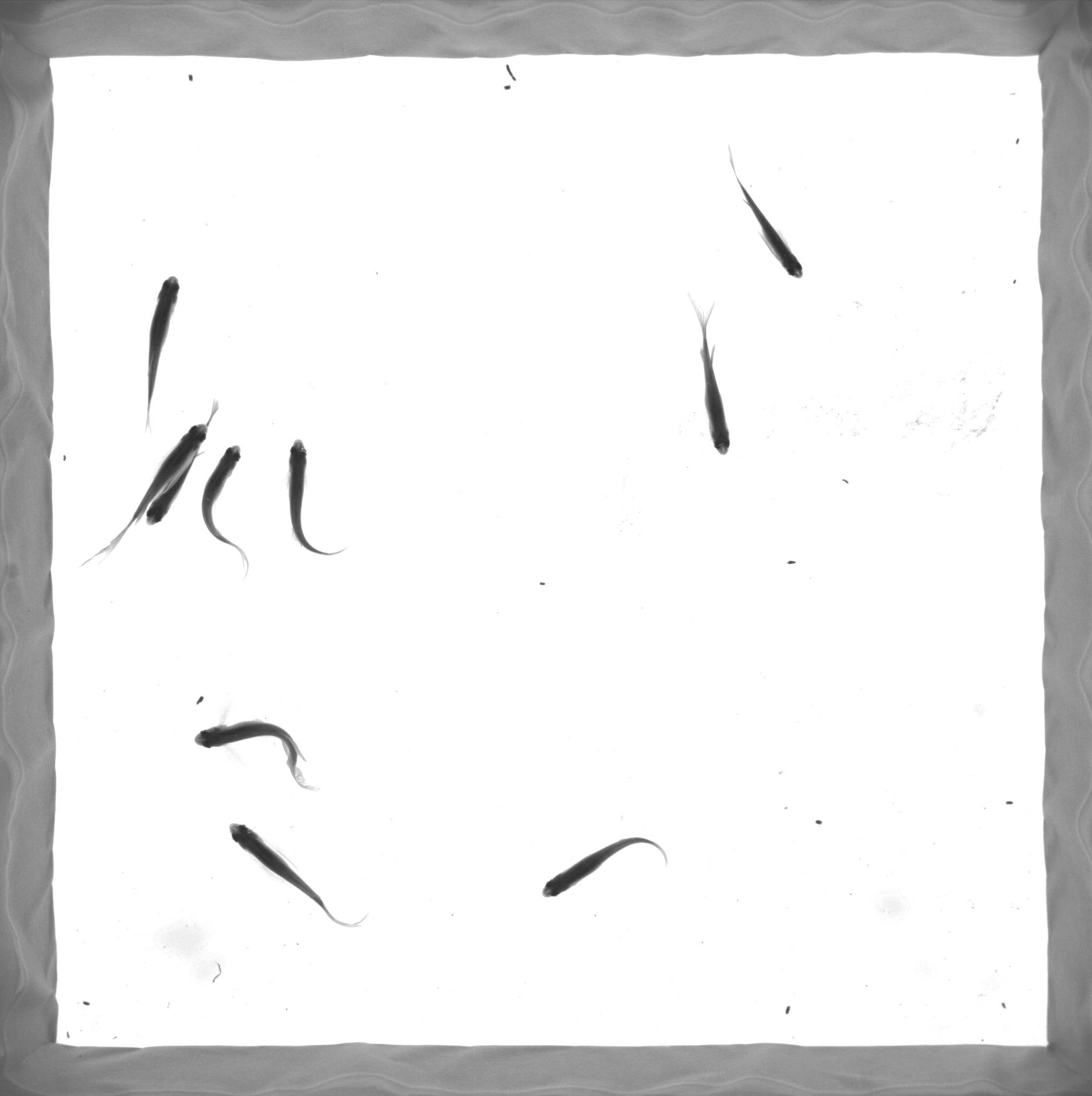

Supplement: S1 File — Source code of the proposed tracking system. (ZIP) [file pone.0154714.s002.zip › code_final/images/CoreView_275_Master_Camera_00100.jpg]

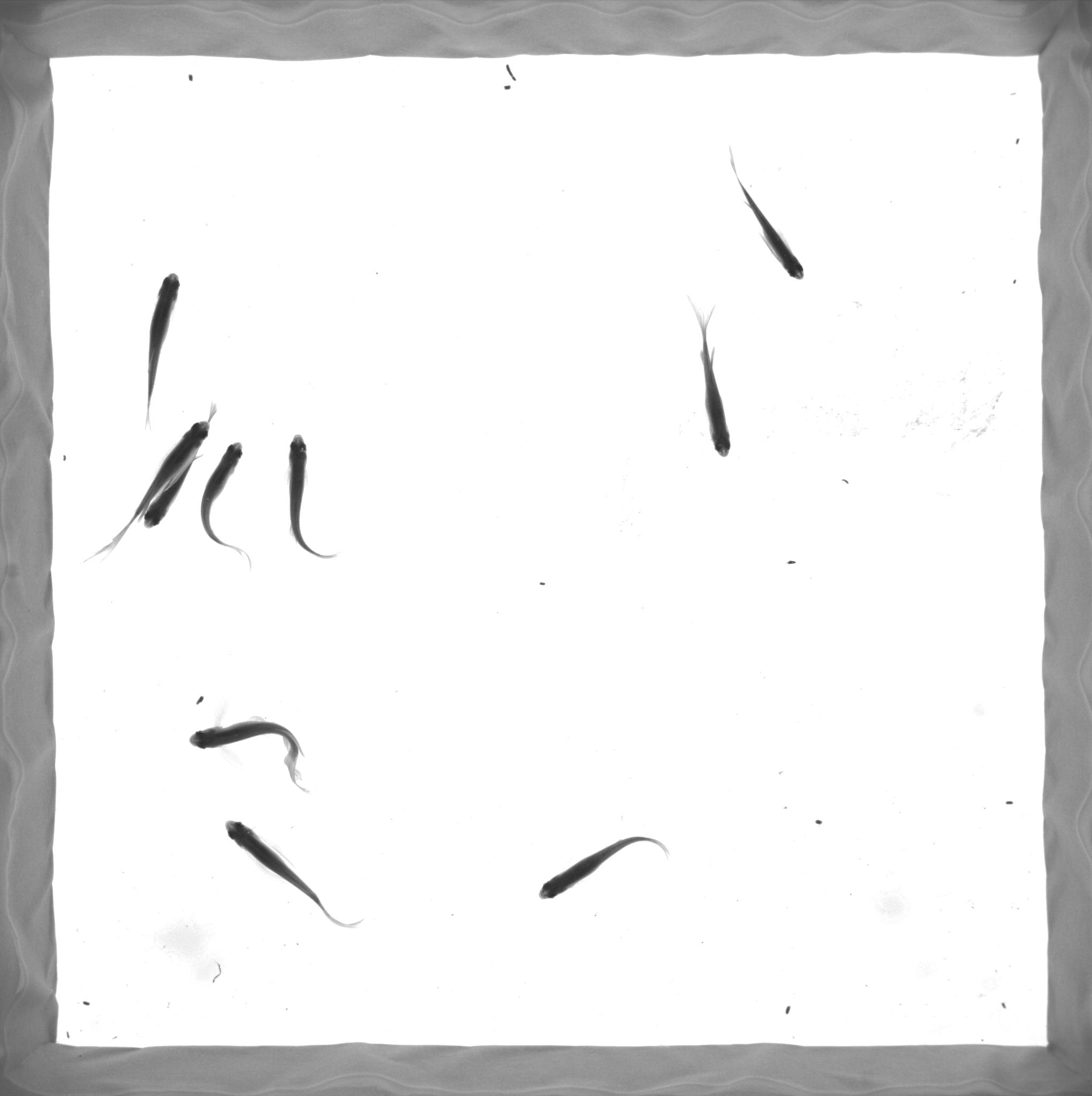

Supplement: S1 File — Source code of the proposed tracking system. (ZIP) [file pone.0154714.s002.zip › code_final/images/CoreView_275_Master_Camera_00101.jpg]

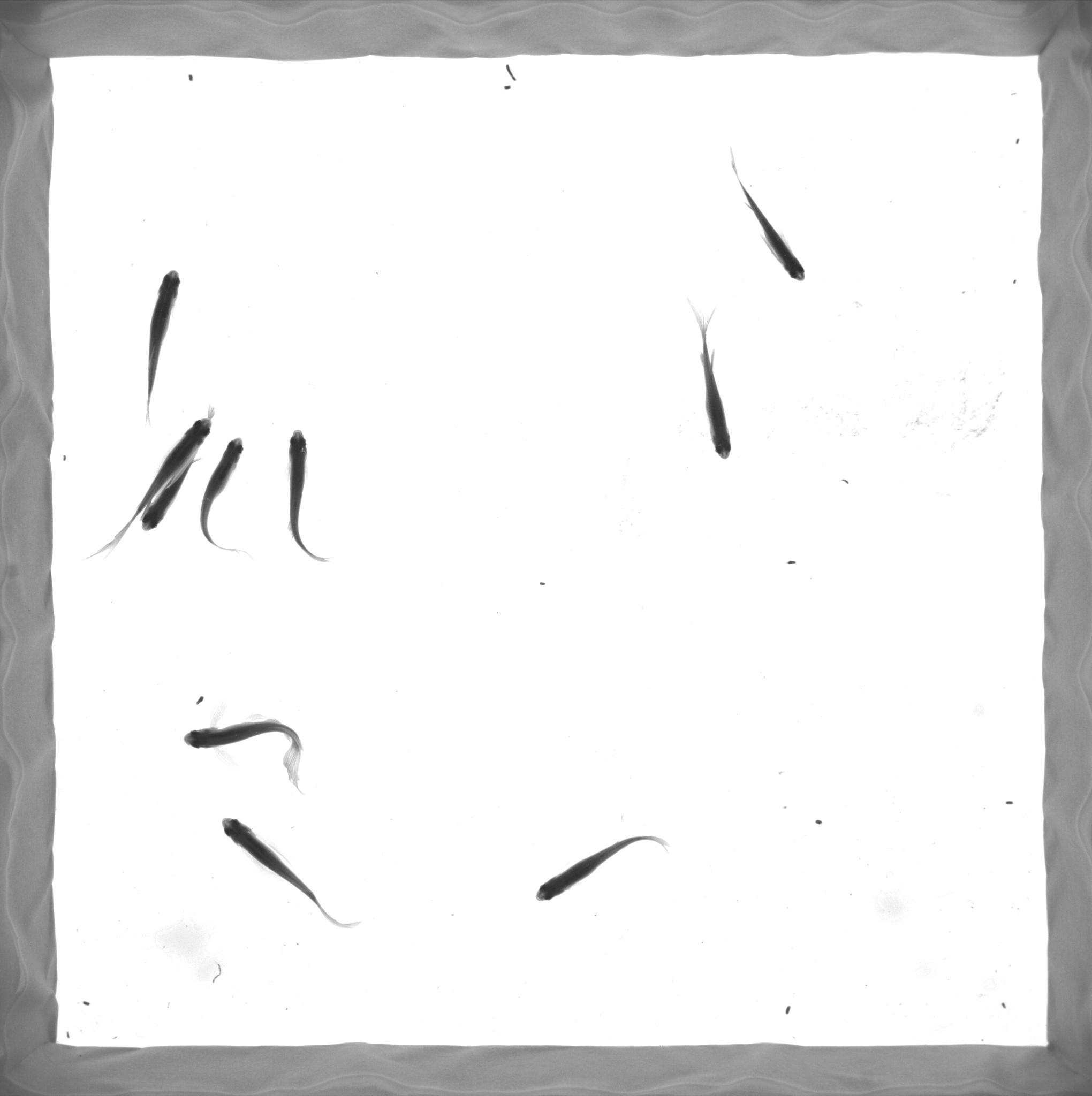

Supplement: S1 File — Source code of the proposed tracking system. (ZIP) [file pone.0154714.s002.zip › code_final/images/CoreView_275_Master_Camera_00102.jpg]

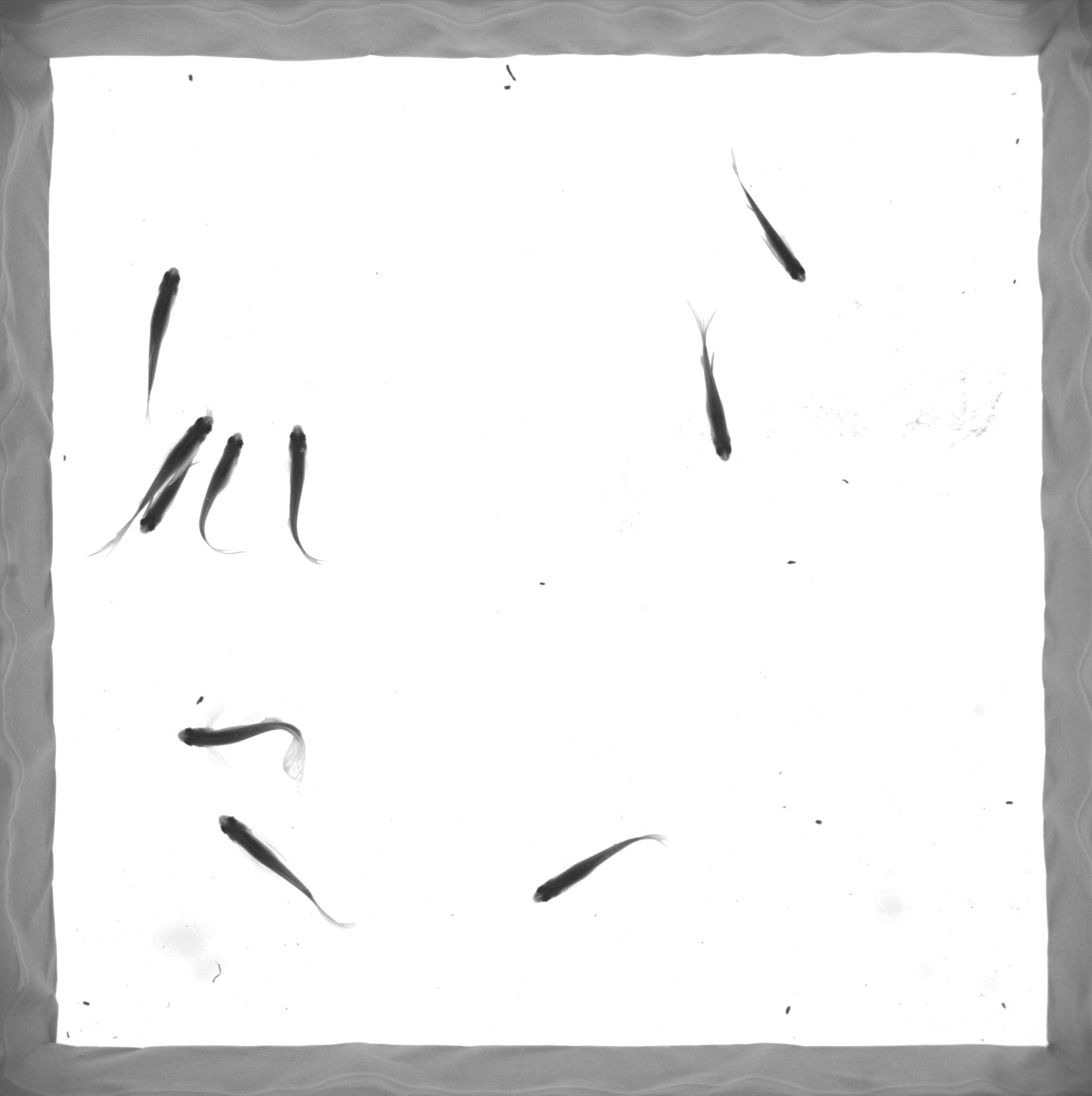

Supplement: S1 File — Source code of the proposed tracking system. (ZIP) [file pone.0154714.s002.zip › code_final/images/CoreView_275_Master_Camera_00103.jpg]

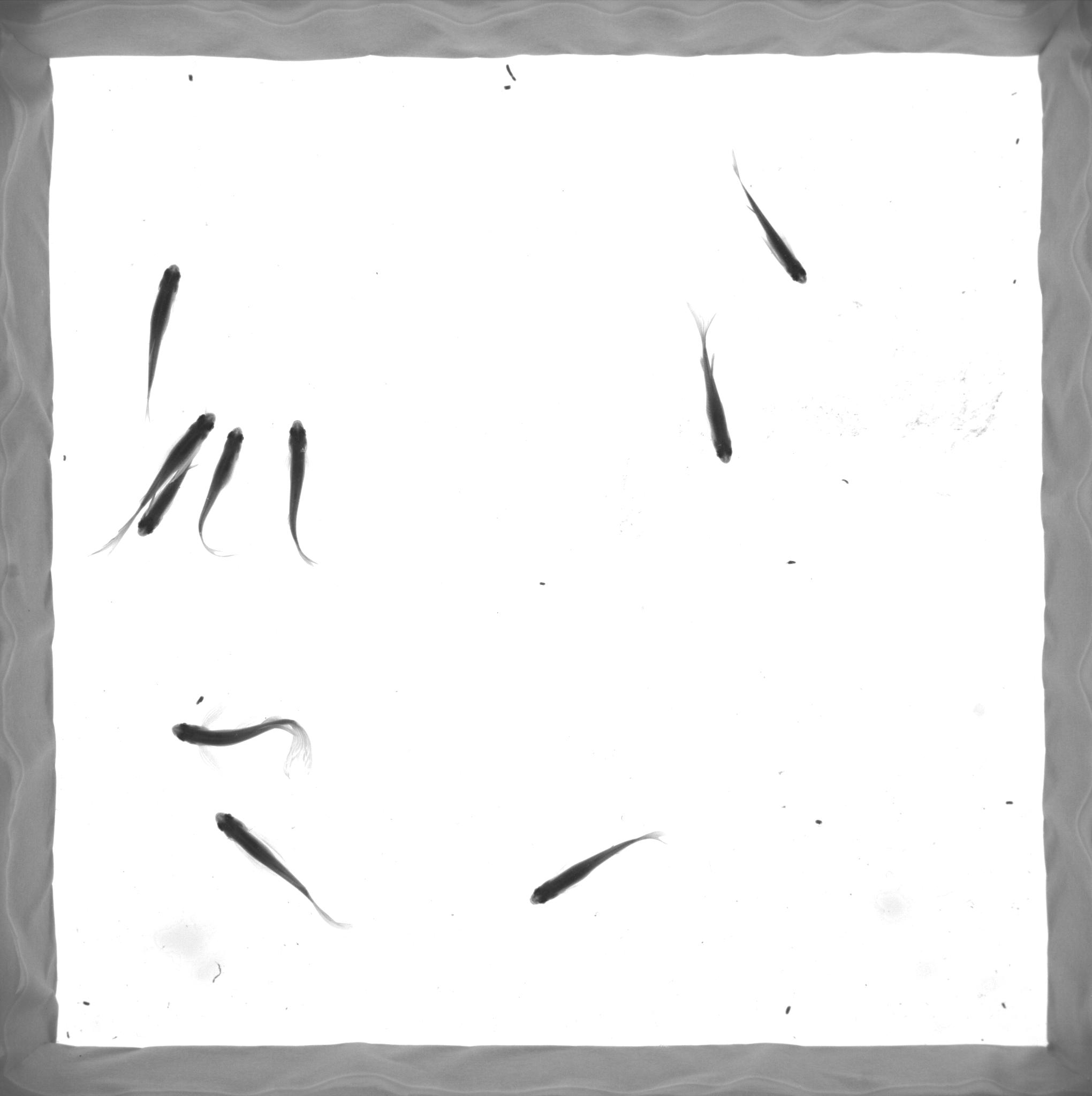

Supplement: S1 File — Source code of the proposed tracking system. (ZIP) [file pone.0154714.s002.zip › code_final/images/CoreView_275_Master_Camera_00104.jpg]

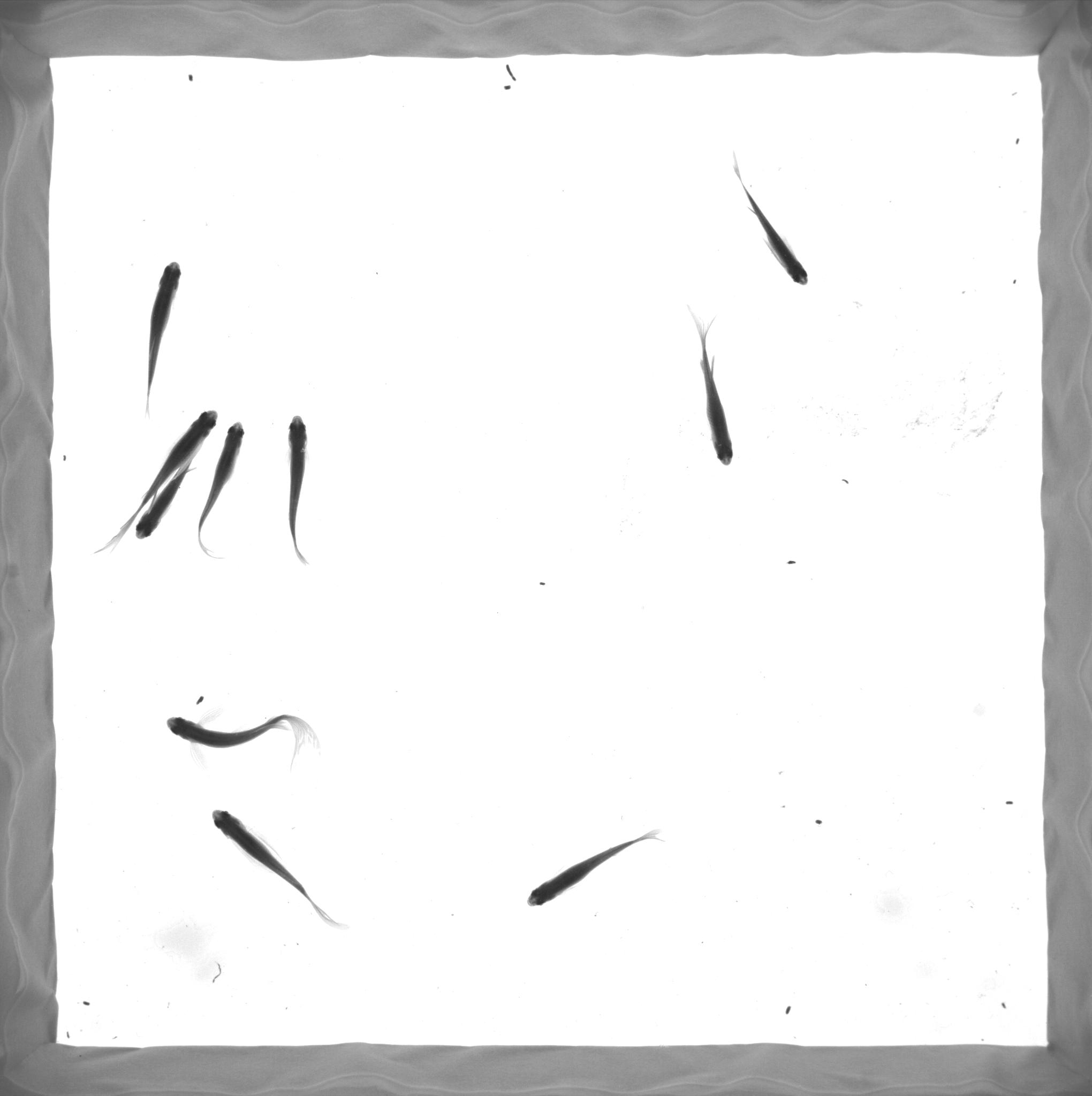

Supplement: S1 File — Source code of the proposed tracking system. (ZIP) [file pone.0154714.s002.zip › code_final/images/CoreView_275_Master_Camera_00105.jpg]

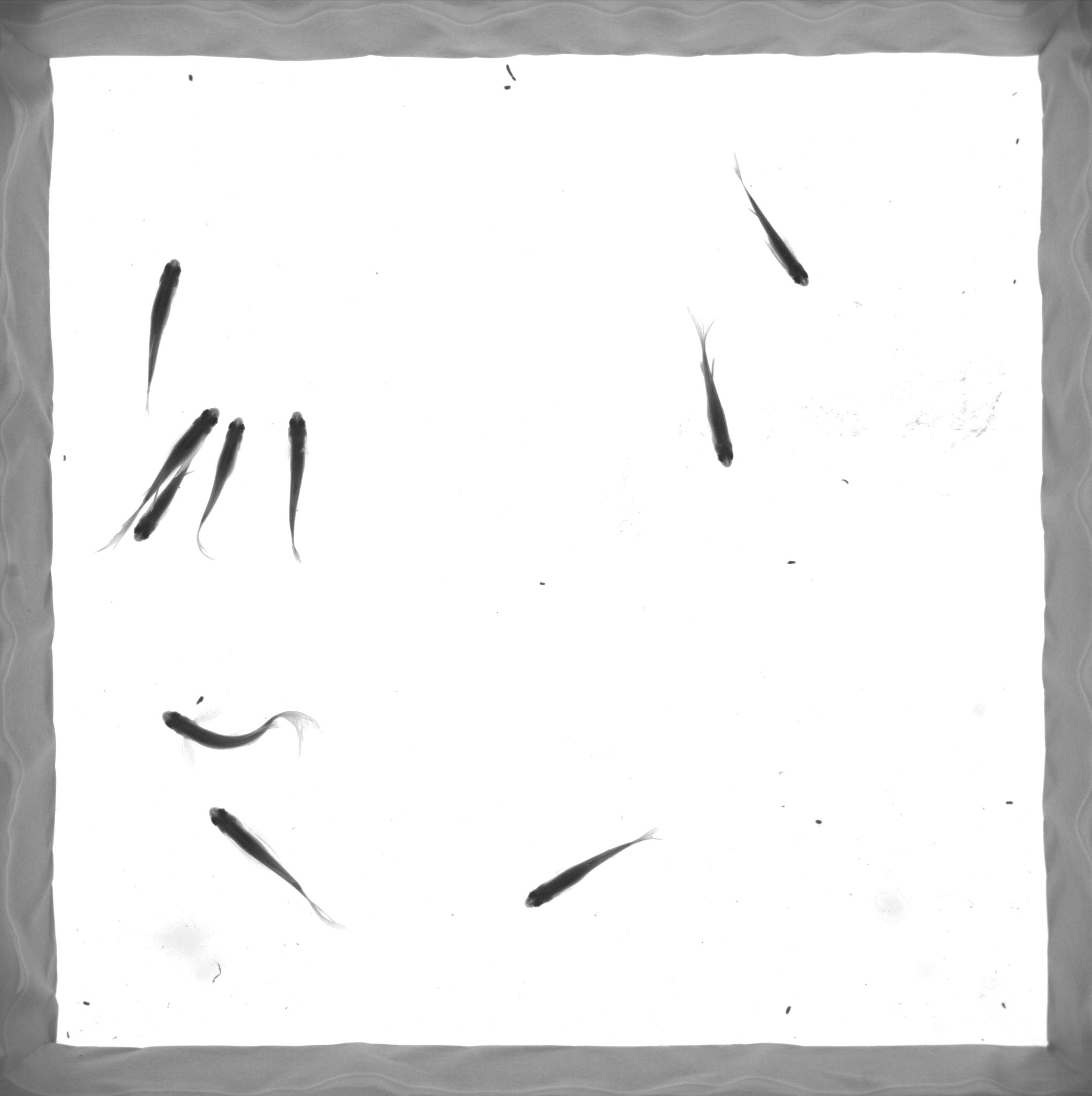

Supplement: S1 File — Source code of the proposed tracking system. (ZIP) [file pone.0154714.s002.zip › code_final/images/CoreView_275_Master_Camera_00106.jpg]

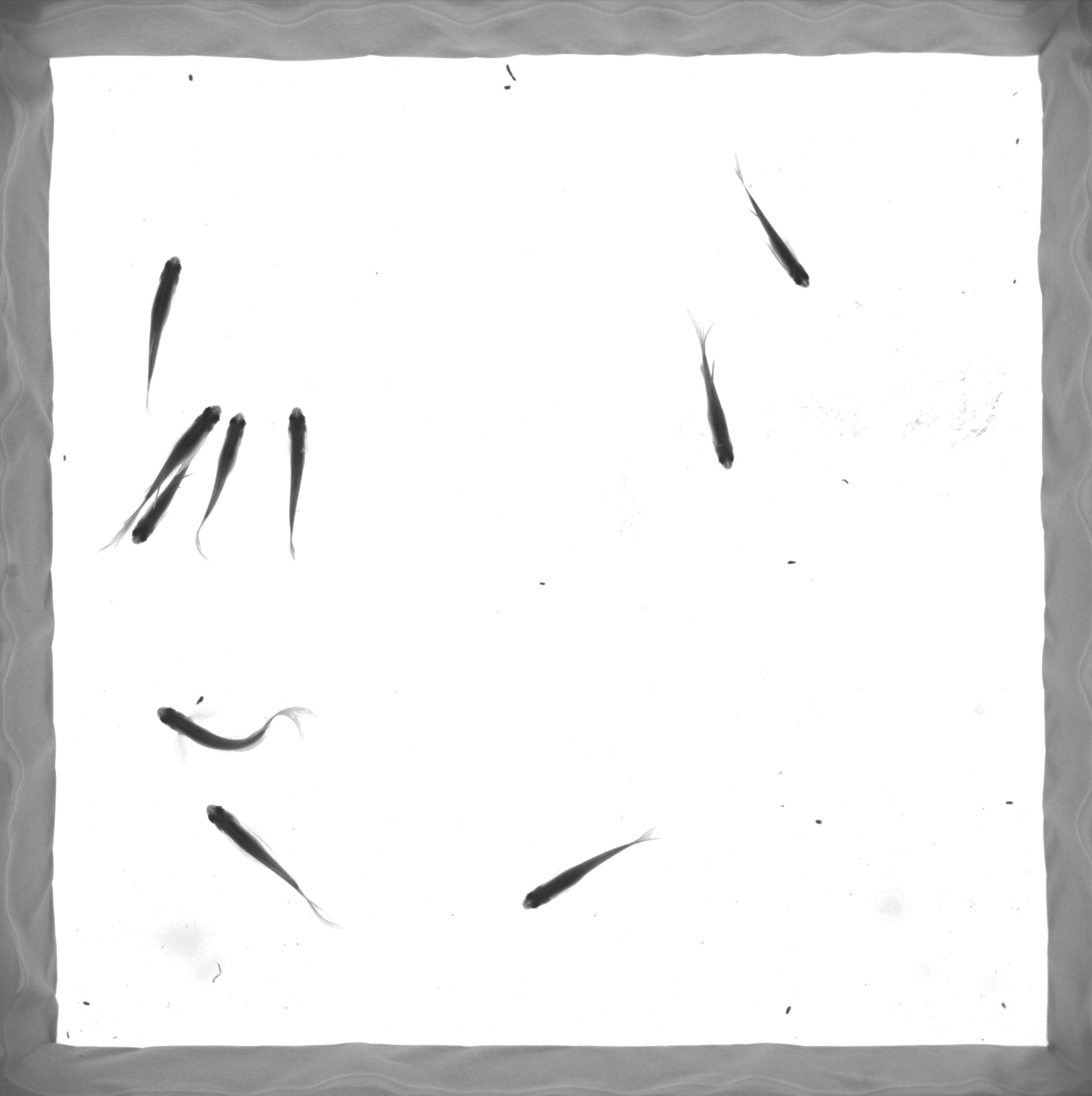

Supplement: S1 File — Source code of the proposed tracking system. (ZIP) [file pone.0154714.s002.zip › code_final/images/CoreView_275_Master_Camera_00107.jpg]

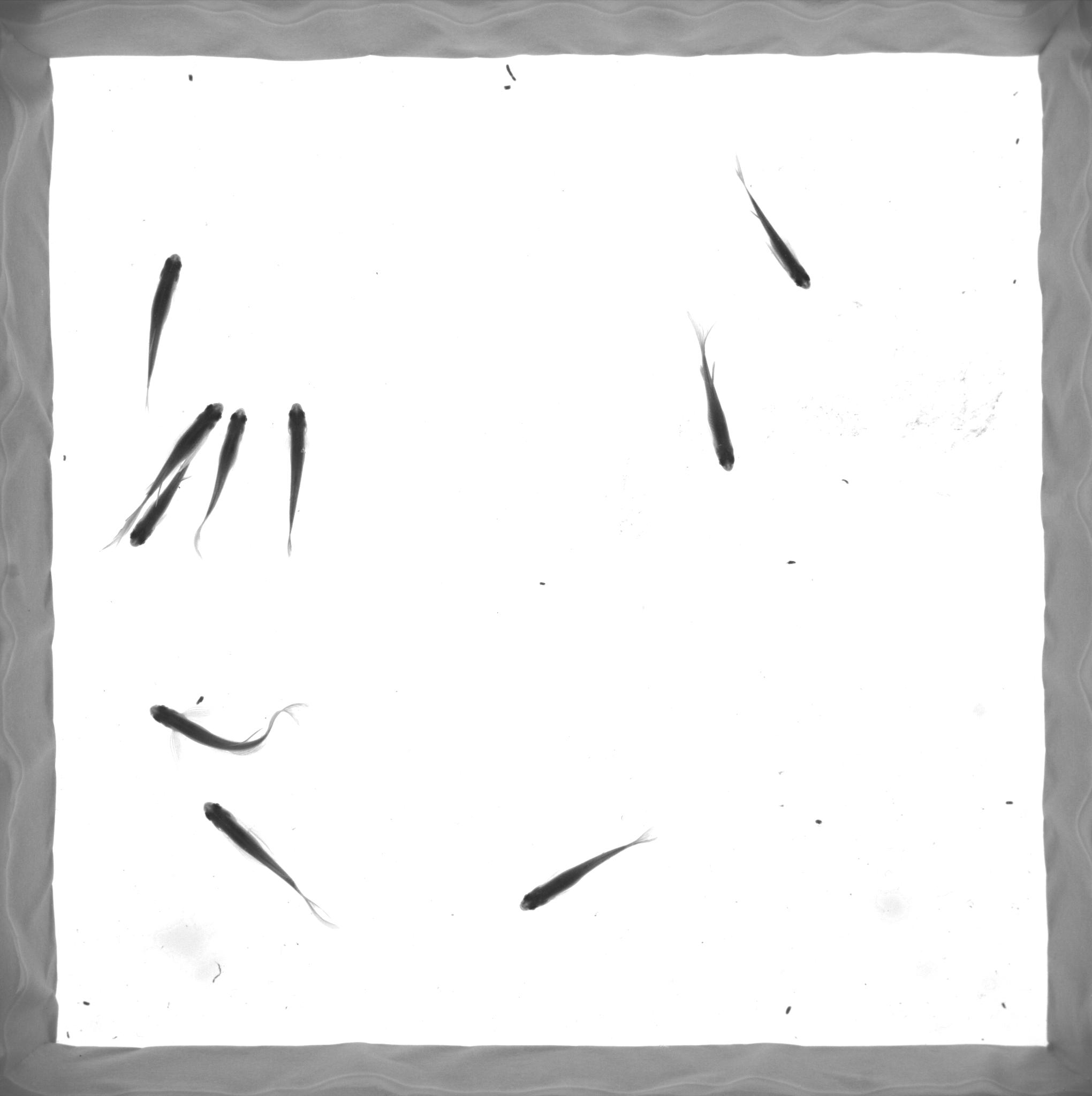

Supplement: S1 File — Source code of the proposed tracking system. (ZIP) [file pone.0154714.s002.zip › code_final/images/CoreView_275_Master_Camera_00108.jpg]

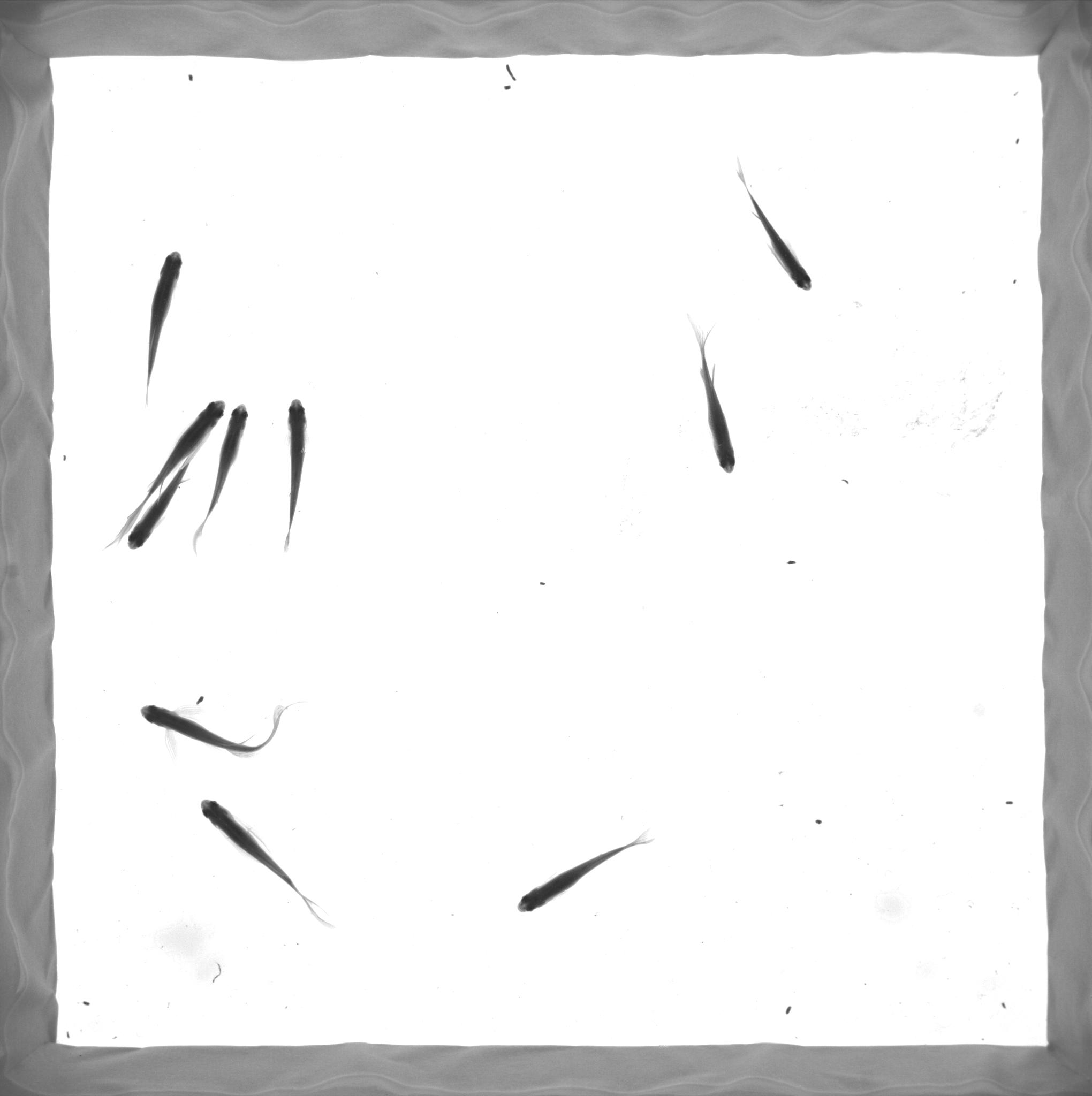

Supplement: S1 File — Source code of the proposed tracking system. (ZIP) [file pone.0154714.s002.zip › code_final/images/CoreView_275_Master_Camera_00109.jpg]

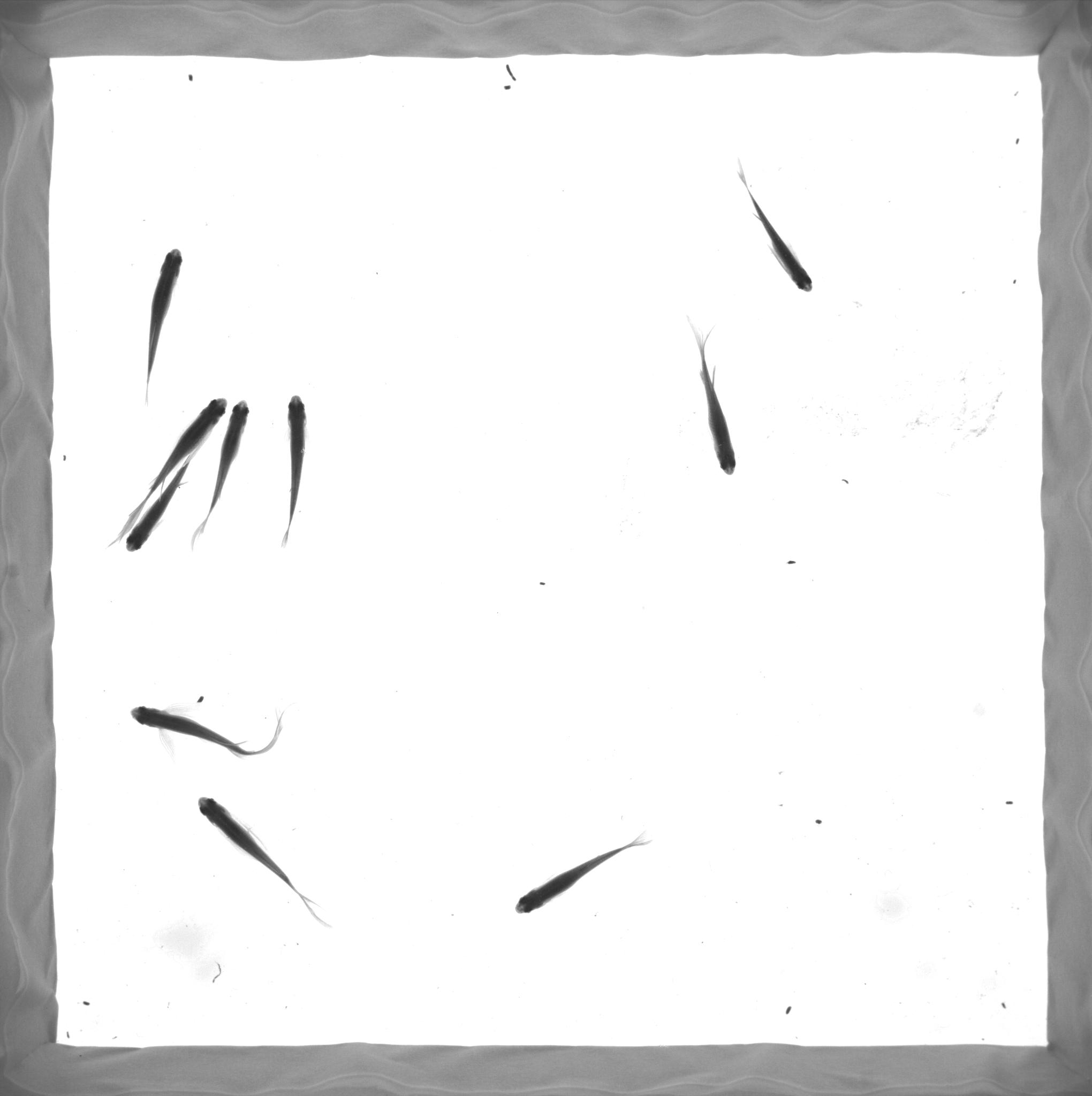

Supplement: S1 File — Source code of the proposed tracking system. (ZIP) [file pone.0154714.s002.zip › code_final/images/CoreView_275_Master_Camera_00110.jpg]

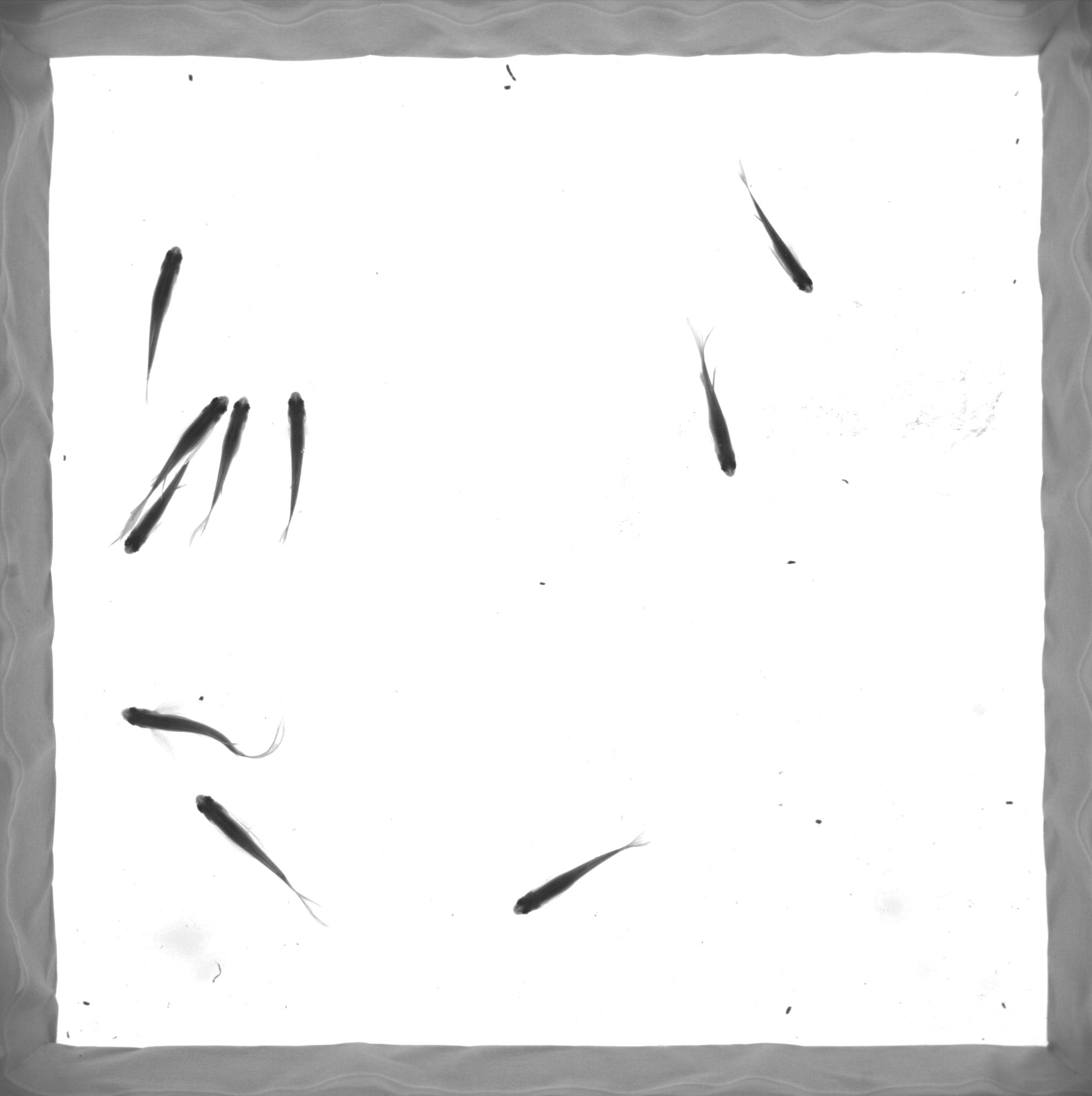

Supplement: S1 File — Source code of the proposed tracking system. (ZIP) [file pone.0154714.s002.zip › code_final/images/CoreView_275_Master_Camera_00111.jpg]

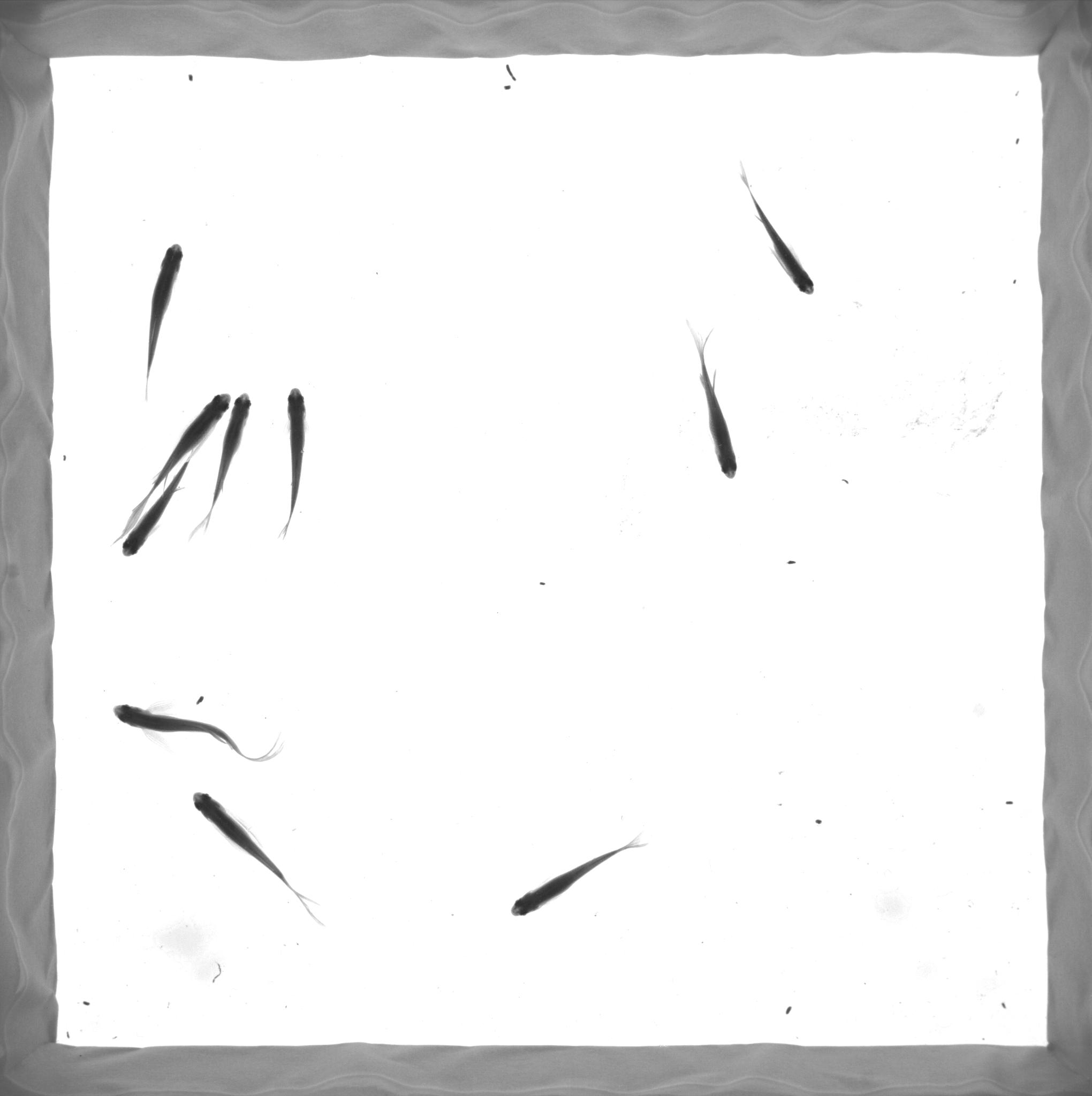

Supplement: S1 File — Source code of the proposed tracking system. (ZIP) [file pone.0154714.s002.zip › code_final/images/CoreView_275_Master_Camera_00112.jpg]

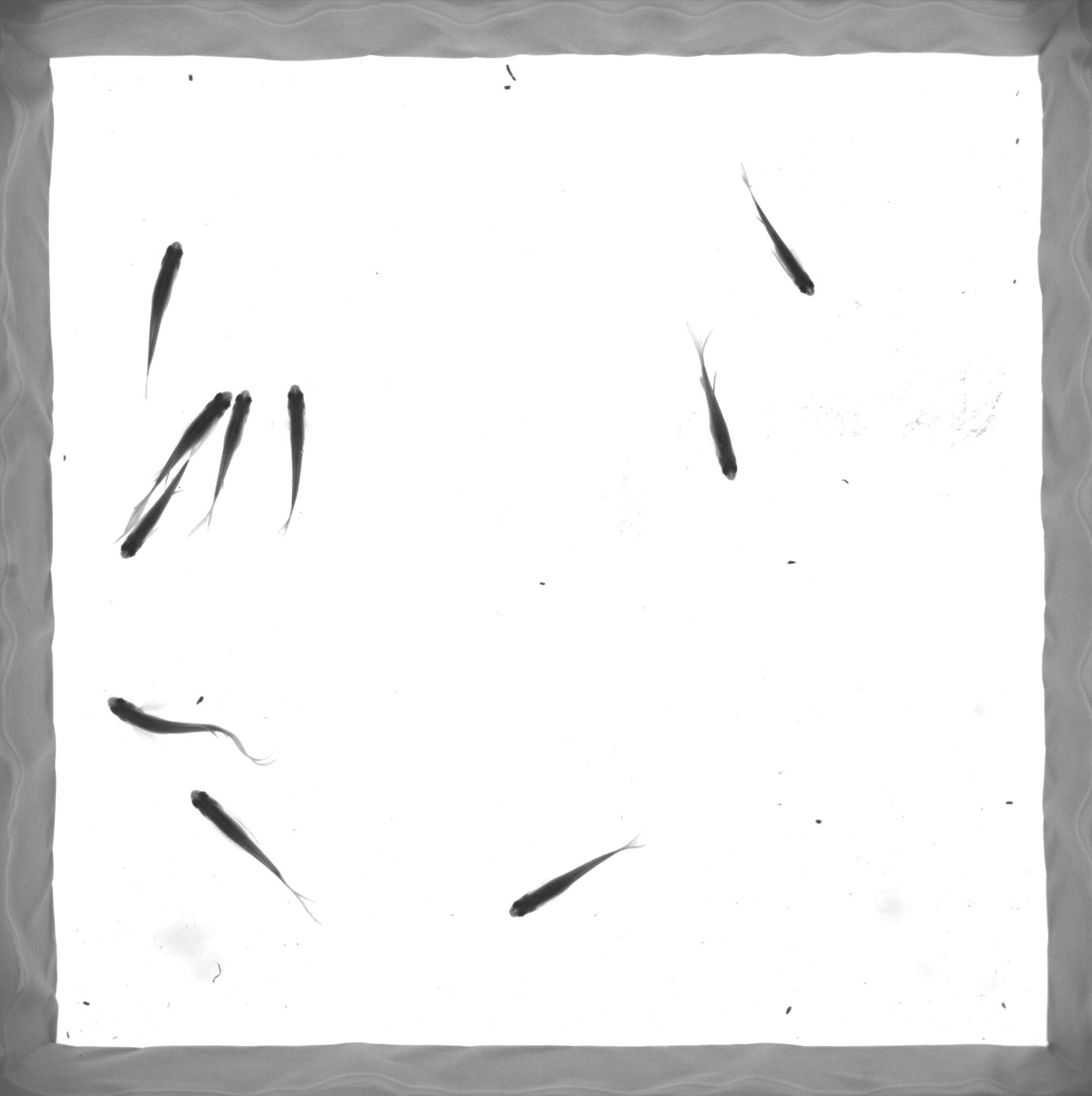

Supplement: S1 File — Source code of the proposed tracking system. (ZIP) [file pone.0154714.s002.zip › code_final/images/CoreView_275_Master_Camera_00113.jpg]

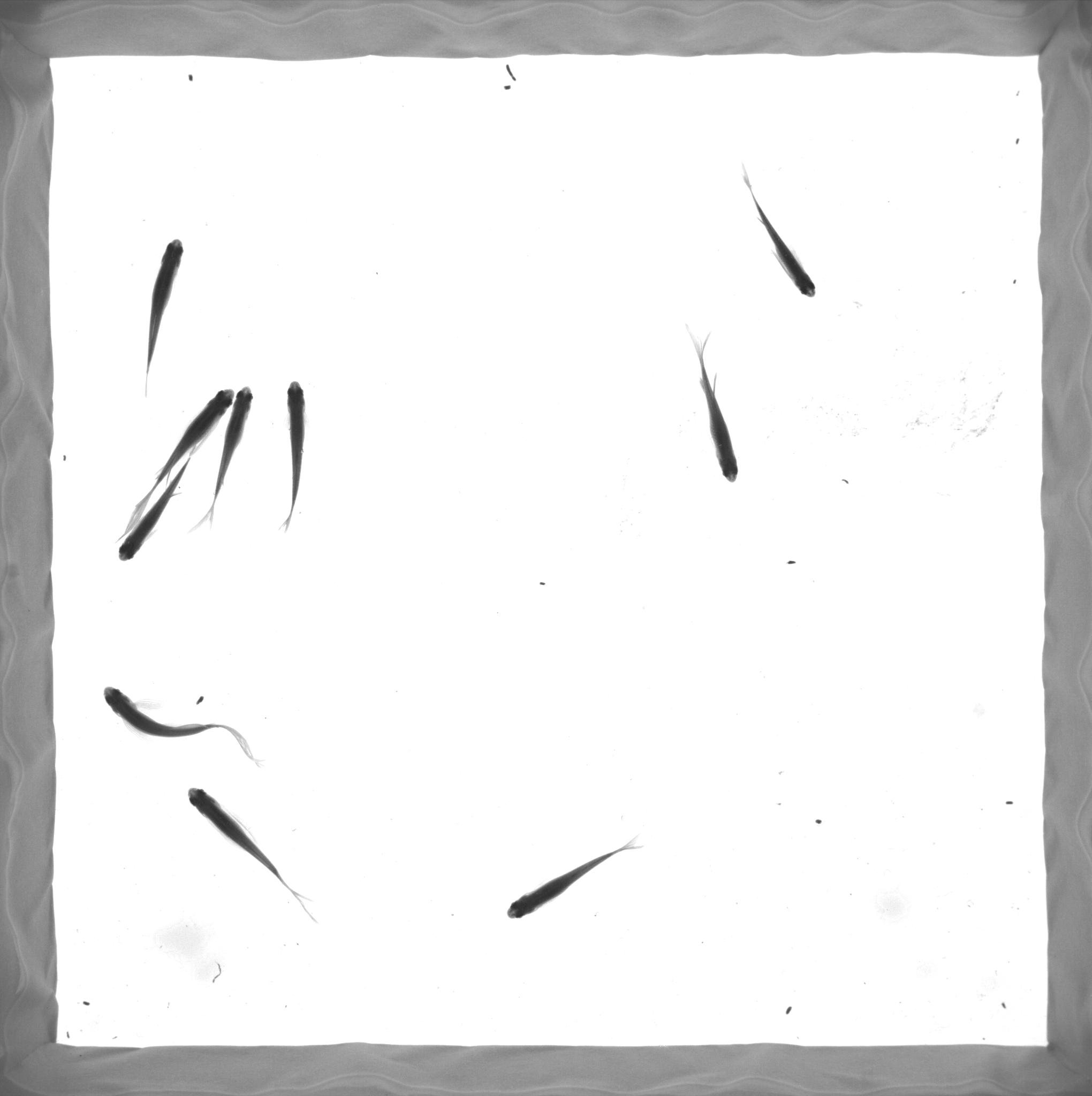

Supplement: S1 File — Source code of the proposed tracking system. (ZIP) [file pone.0154714.s002.zip › code_final/images/CoreView_275_Master_Camera_00114.jpg]

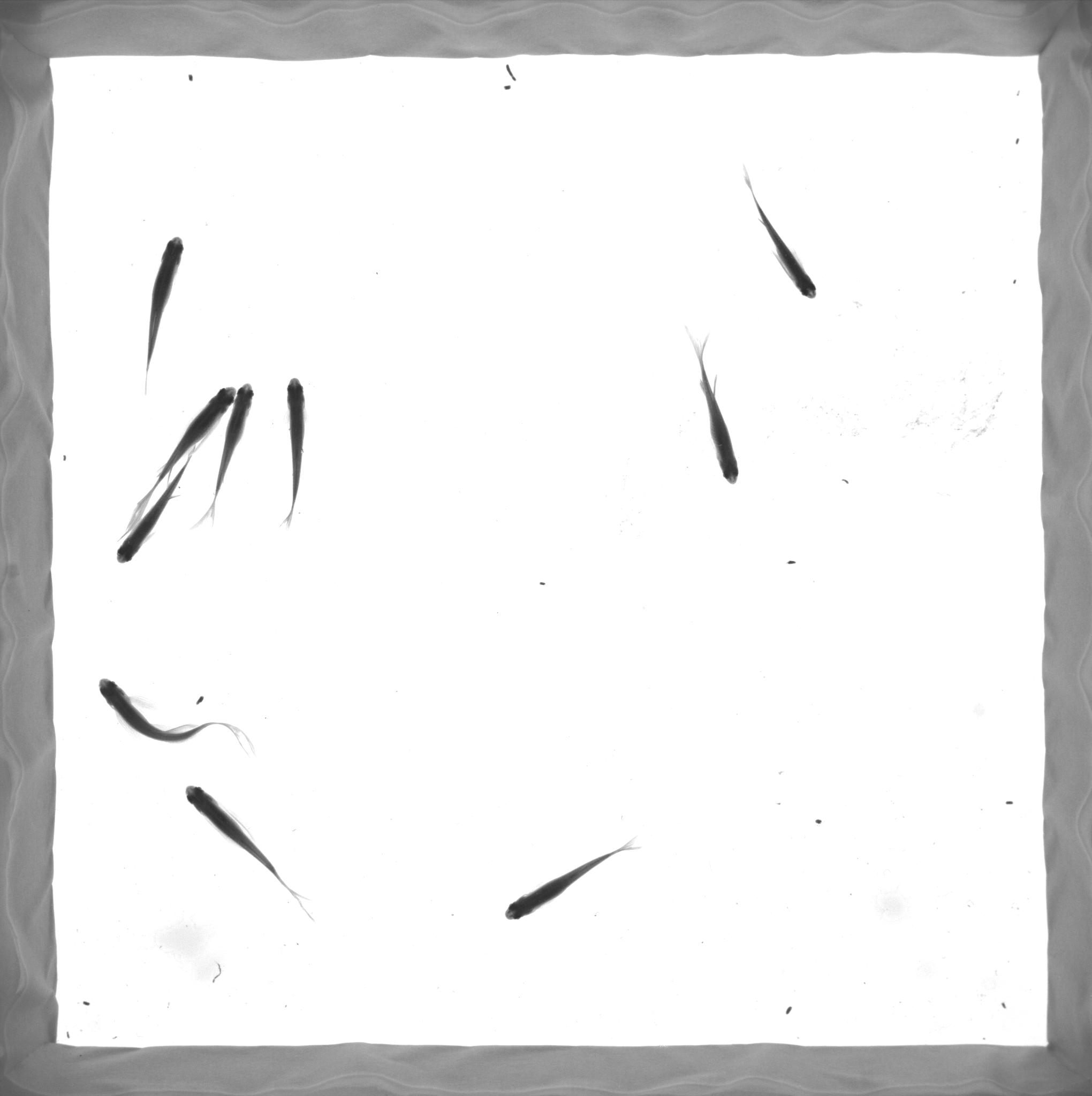

Supplement: S1 File — Source code of the proposed tracking system. (ZIP) [file pone.0154714.s002.zip › code_final/images/CoreView_275_Master_Camera_00115.jpg]

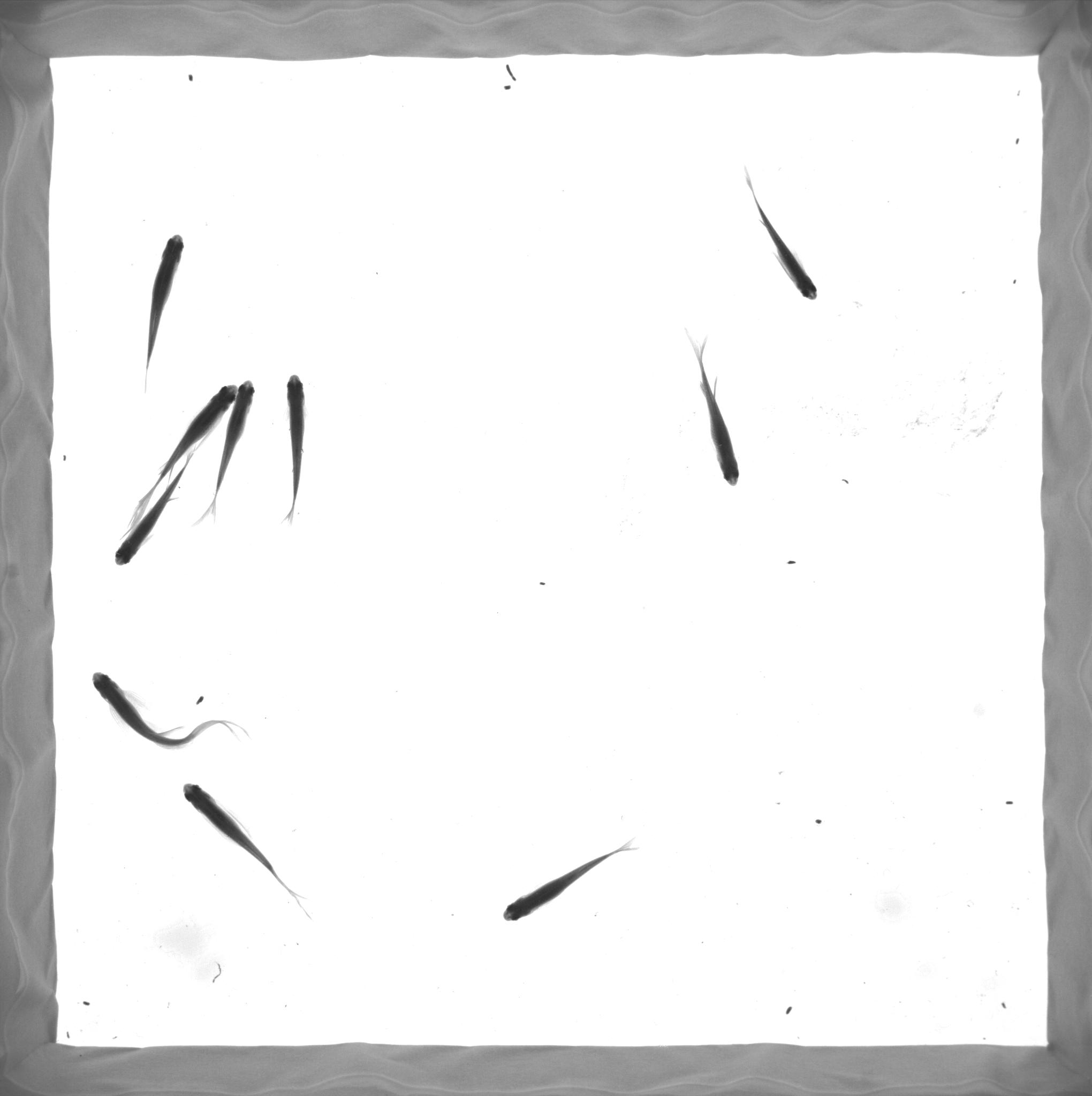

Supplement: S1 File — Source code of the proposed tracking system. (ZIP) [file pone.0154714.s002.zip › code_final/images/CoreView_275_Master_Camera_00116.jpg]

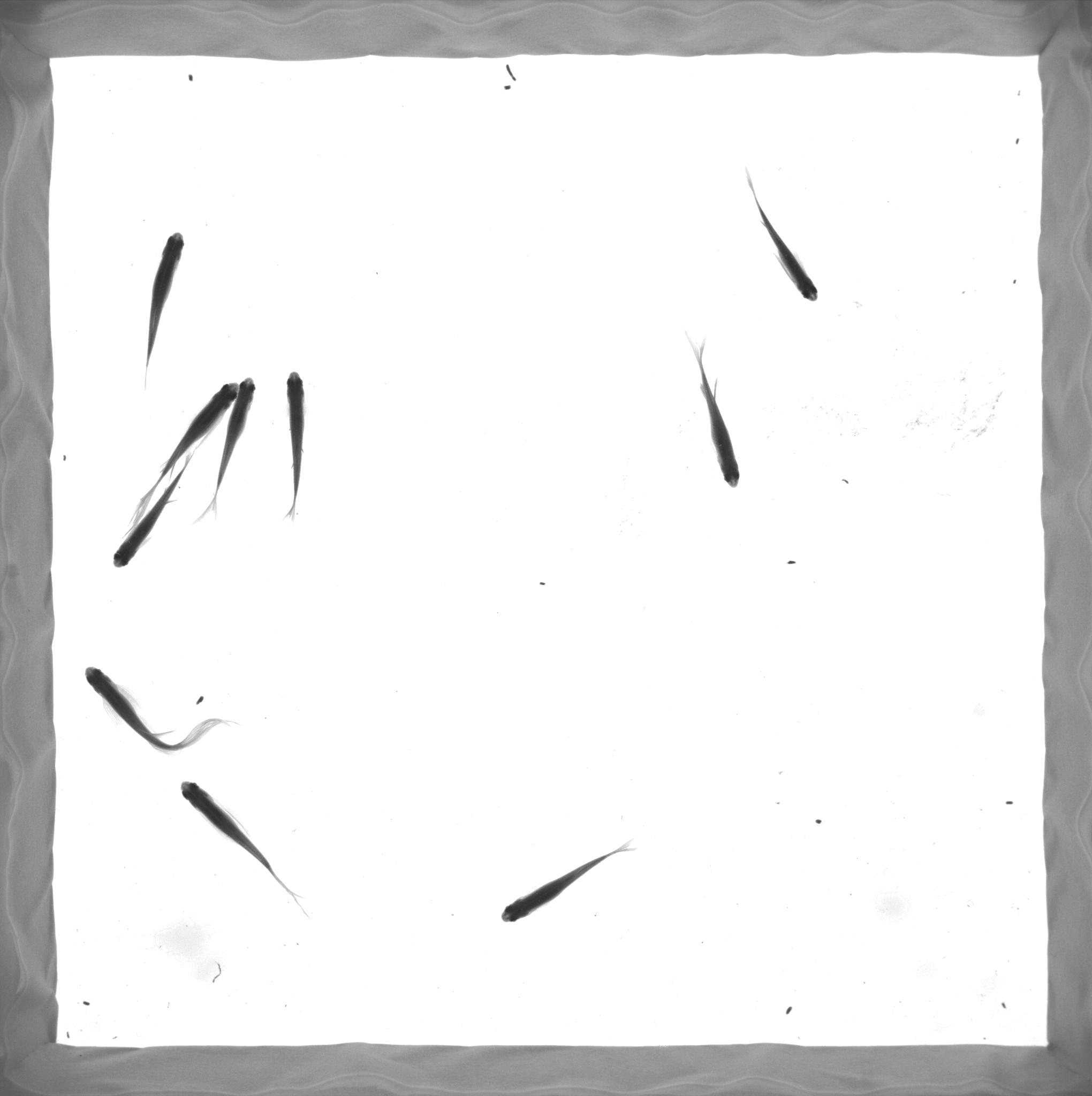

Supplement: S1 File — Source code of the proposed tracking system. (ZIP) [file pone.0154714.s002.zip › code_final/images/CoreView_275_Master_Camera_00117.jpg]

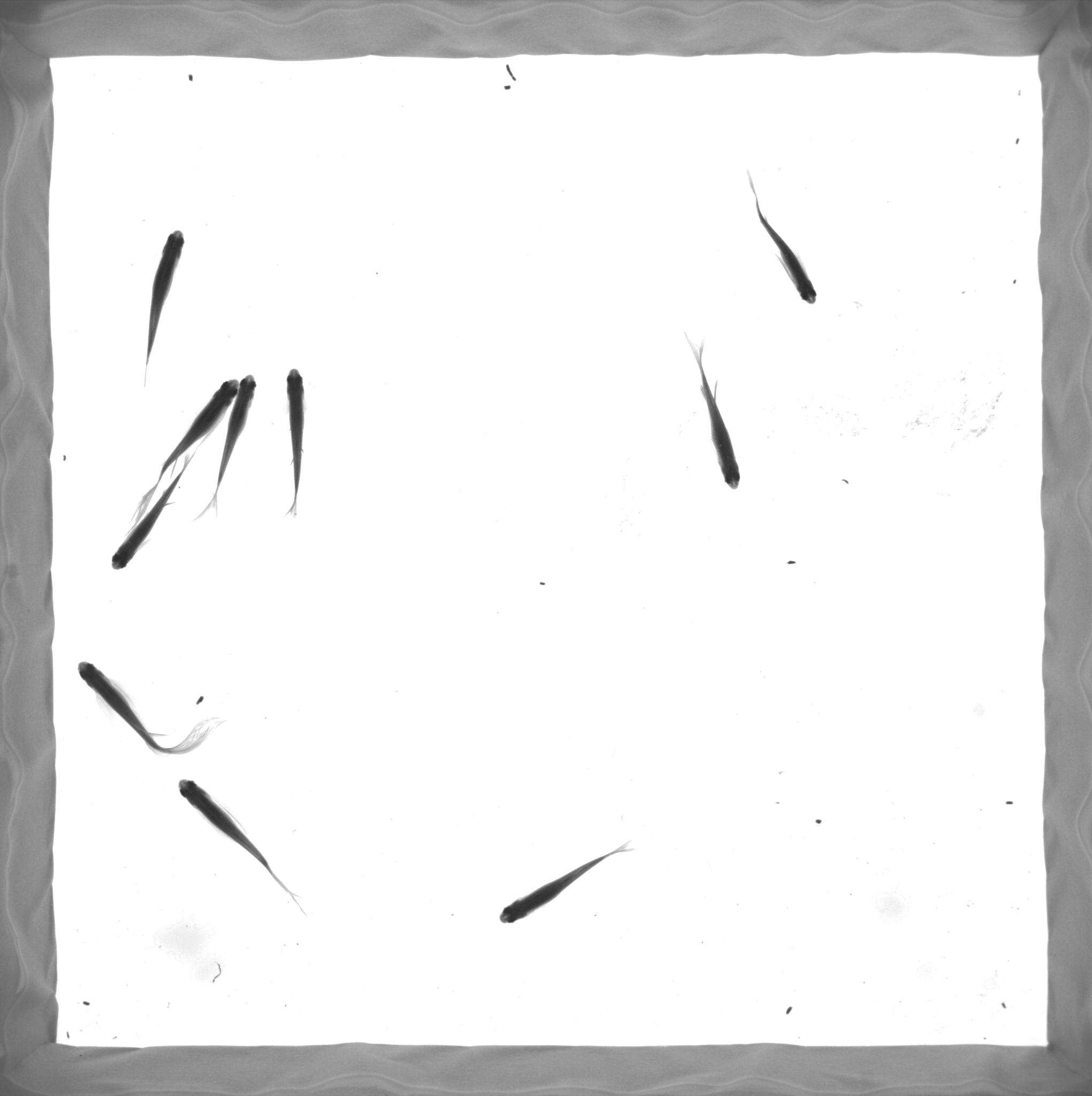

Supplement: S1 File — Source code of the proposed tracking system. (ZIP) [file pone.0154714.s002.zip › code_final/images/CoreView_275_Master_Camera_00118.jpg]

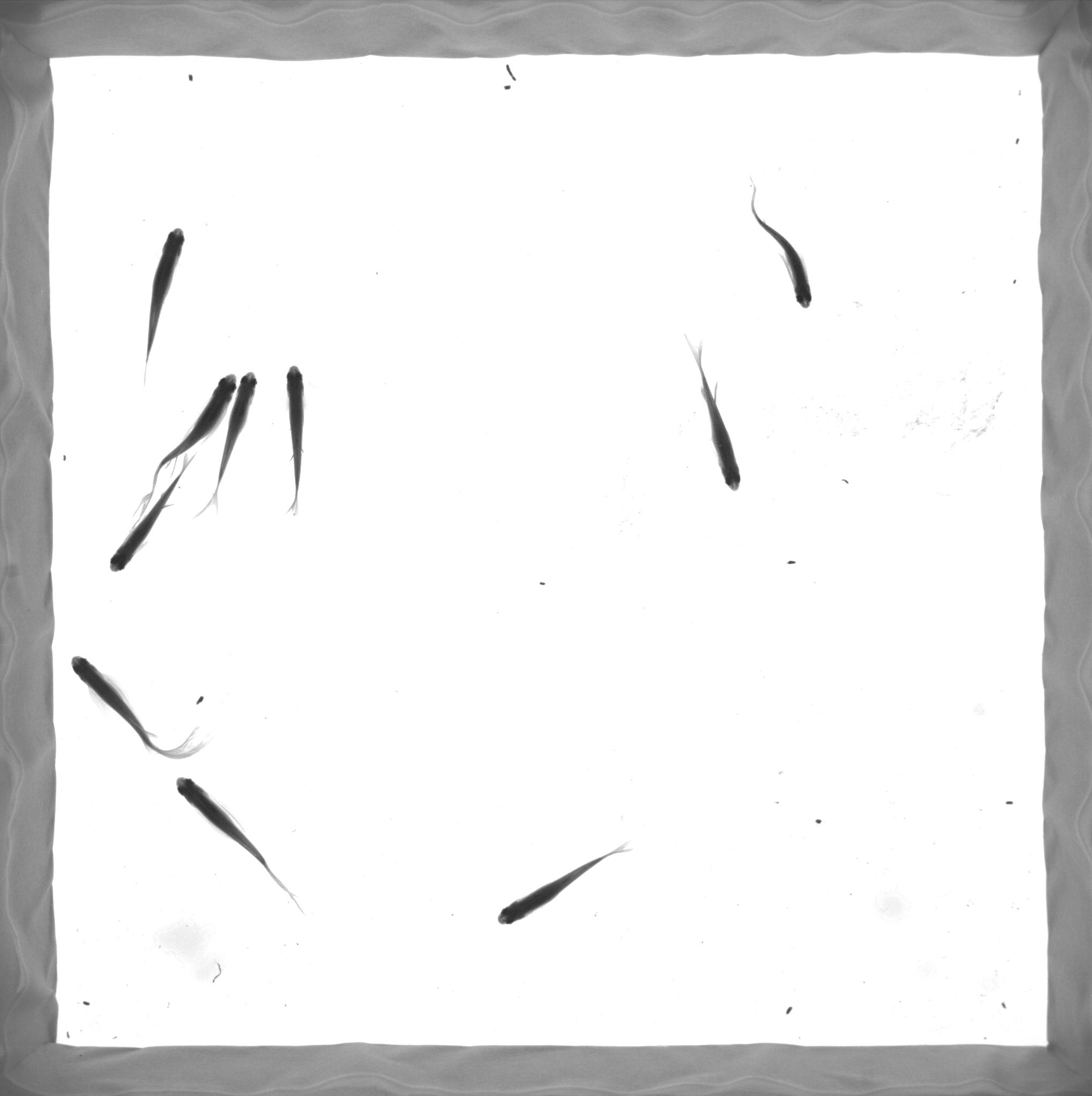

Supplement: S1 File — Source code of the proposed tracking system. (ZIP) [file pone.0154714.s002.zip › code_final/images/CoreView_275_Master_Camera_00119.jpg]

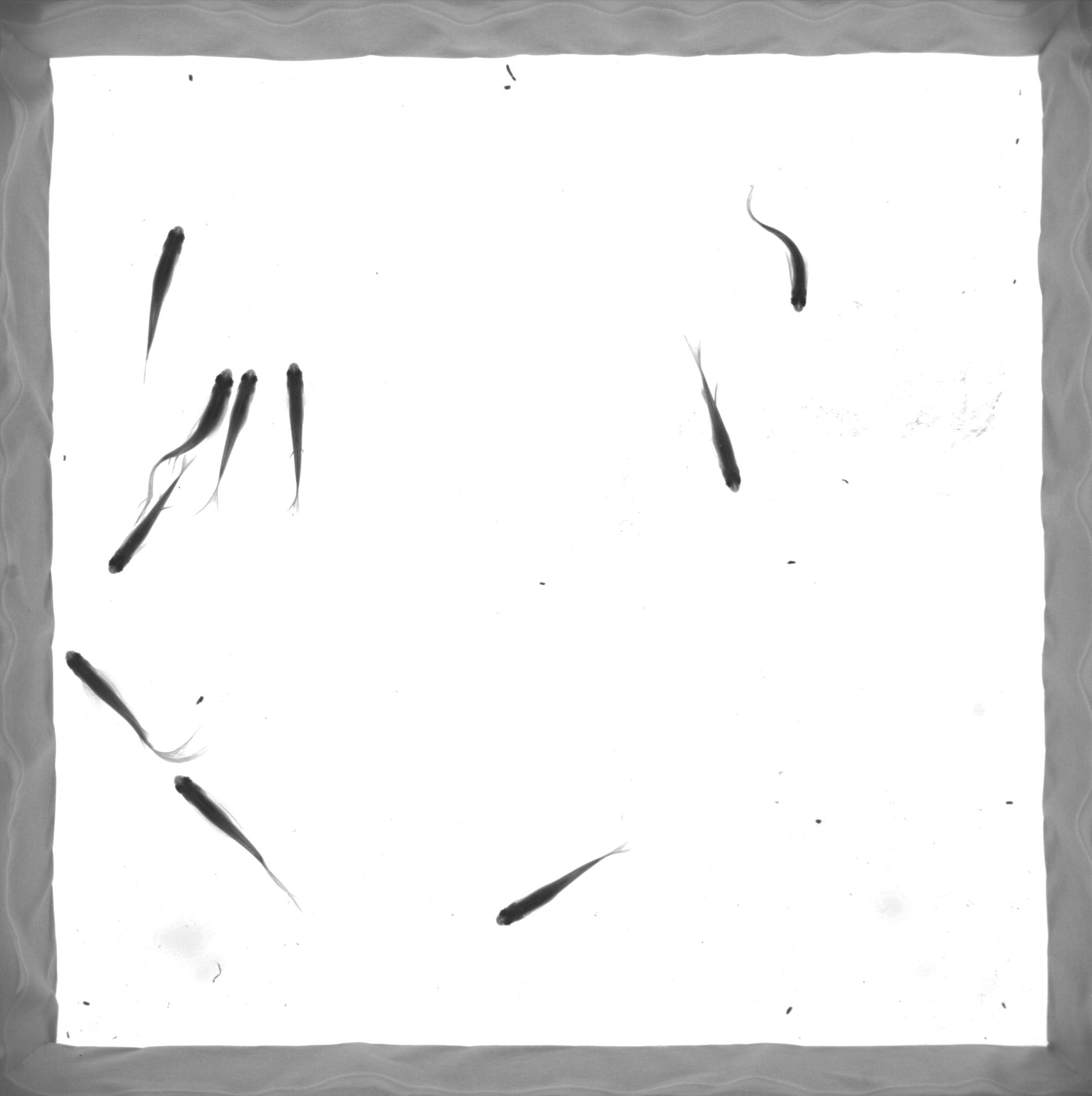

Supplement: S1 File — Source code of the proposed tracking system. (ZIP) [file pone.0154714.s002.zip › code_final/images/CoreView_275_Master_Camera_00120.jpg]

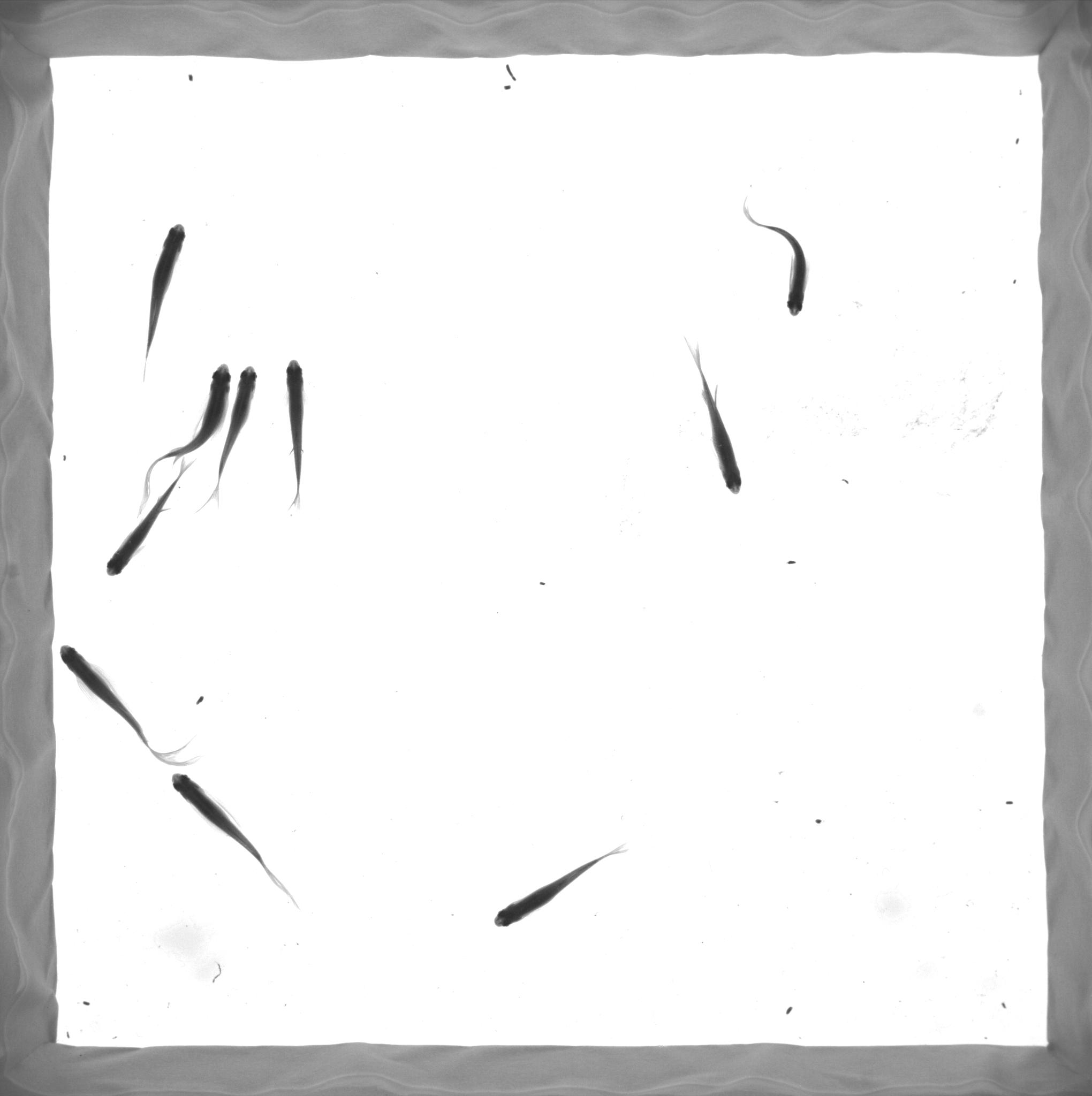

Supplement: S1 File — Source code of the proposed tracking system. (ZIP) [file pone.0154714.s002.zip › code_final/images/CoreView_275_Master_Camera_00121.jpg]

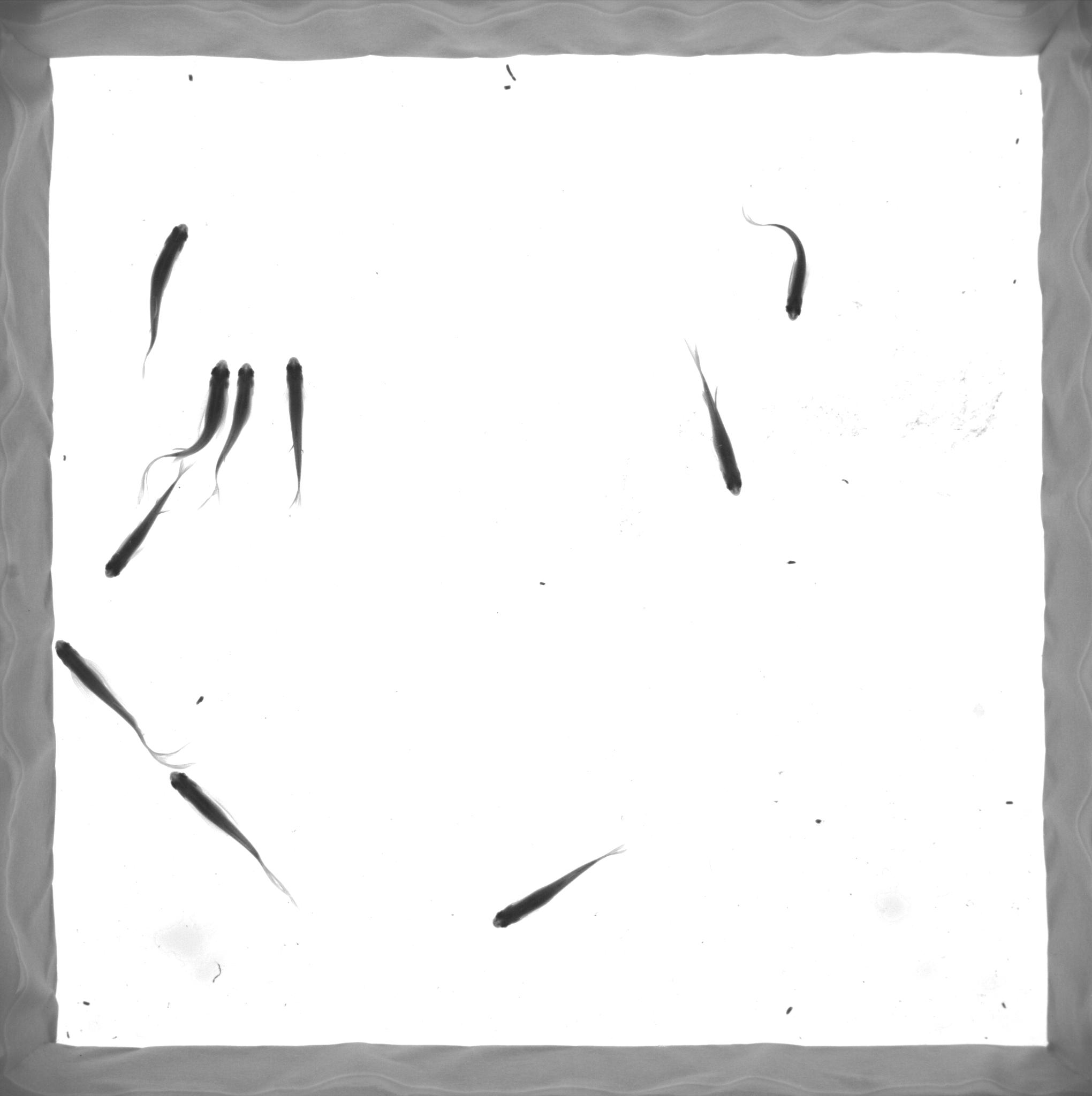

Supplement: S1 File — Source code of the proposed tracking system. (ZIP) [file pone.0154714.s002.zip › code_final/images/CoreView_275_Master_Camera_00122.jpg]

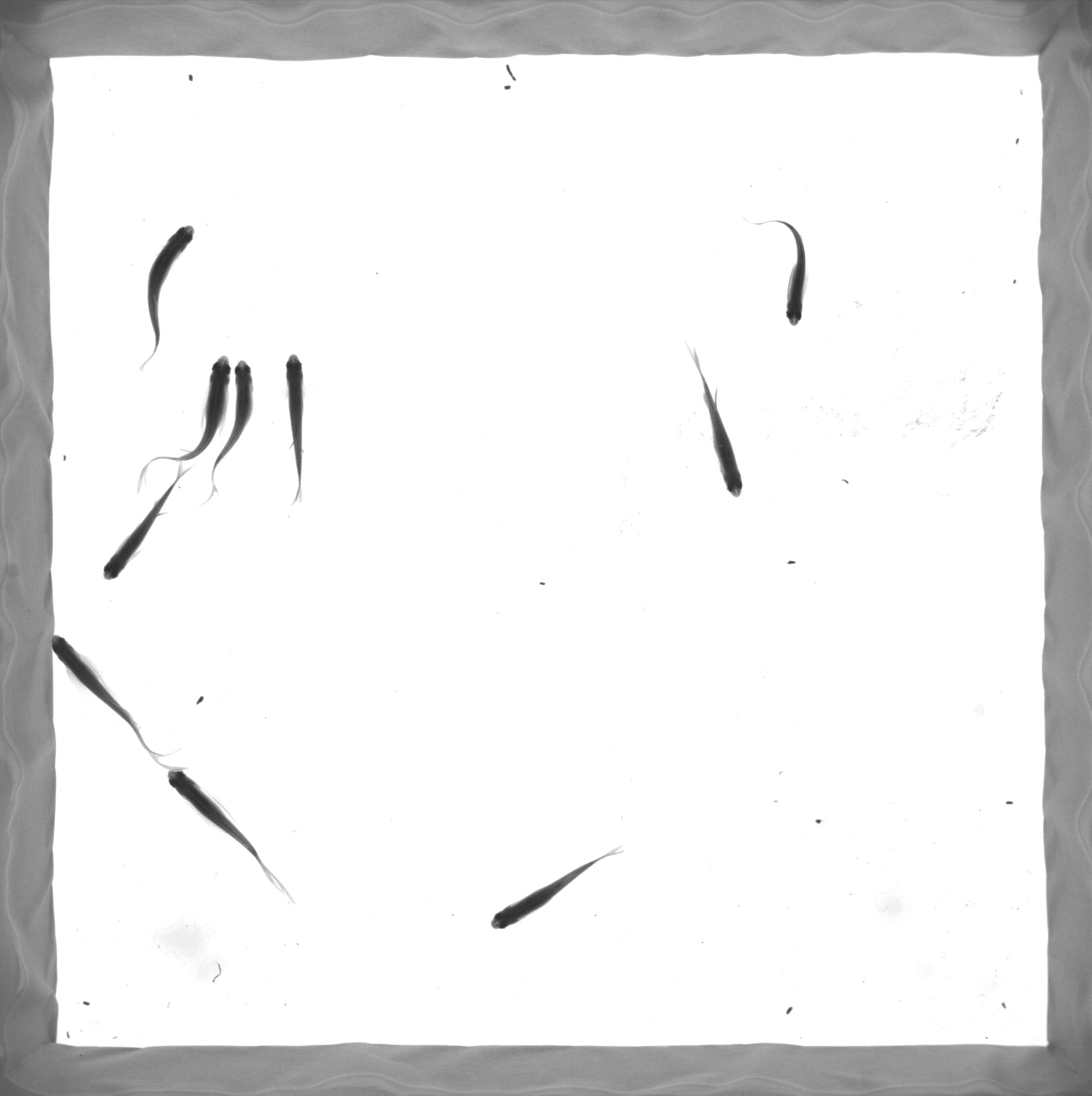

Supplement: S1 File — Source code of the proposed tracking system. (ZIP) [file pone.0154714.s002.zip › code_final/images/CoreView_275_Master_Camera_00123.jpg]

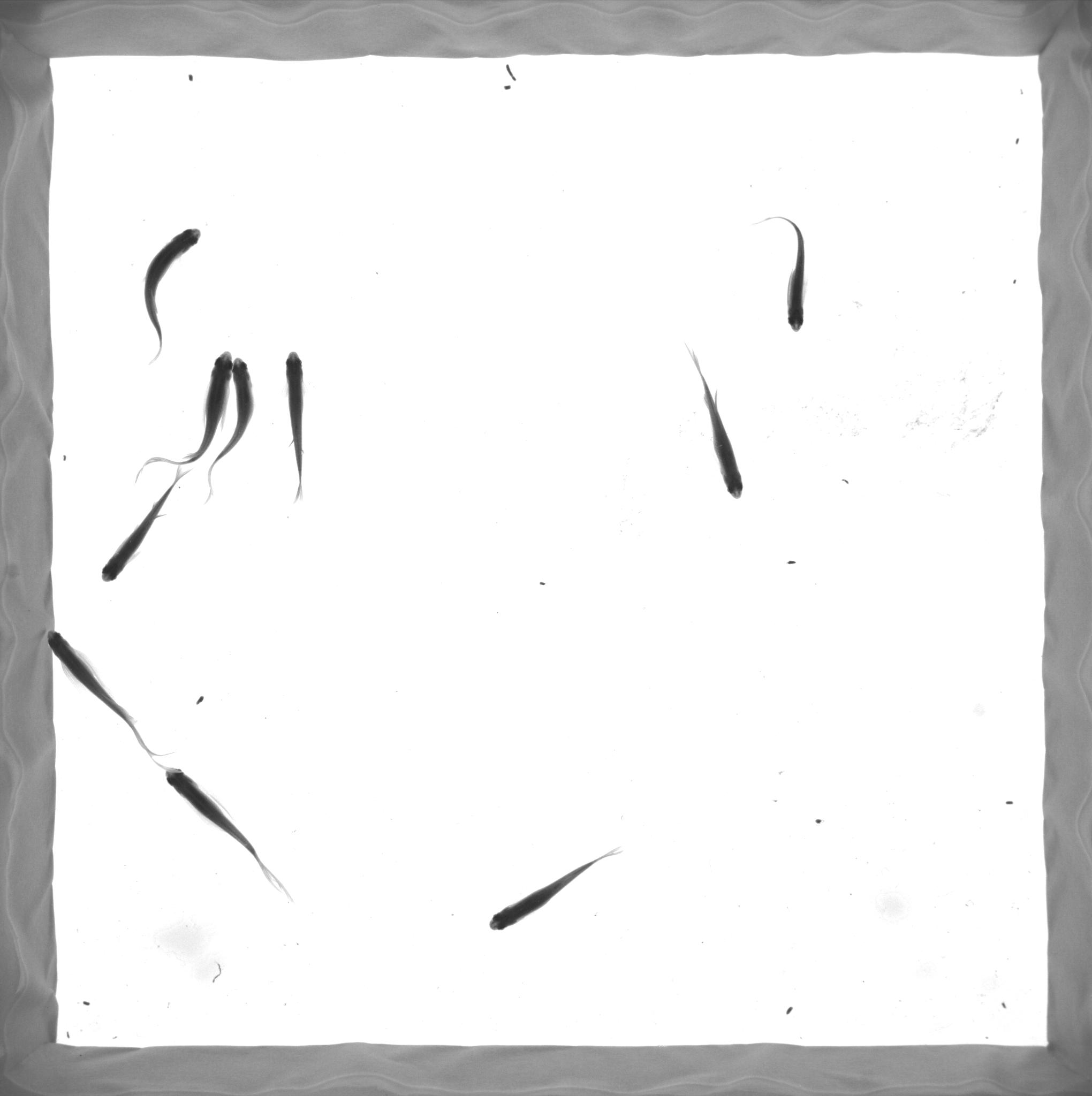

Supplement: S1 File — Source code of the proposed tracking system. (ZIP) [file pone.0154714.s002.zip › code_final/images/CoreView_275_Master_Camera_00124.jpg]

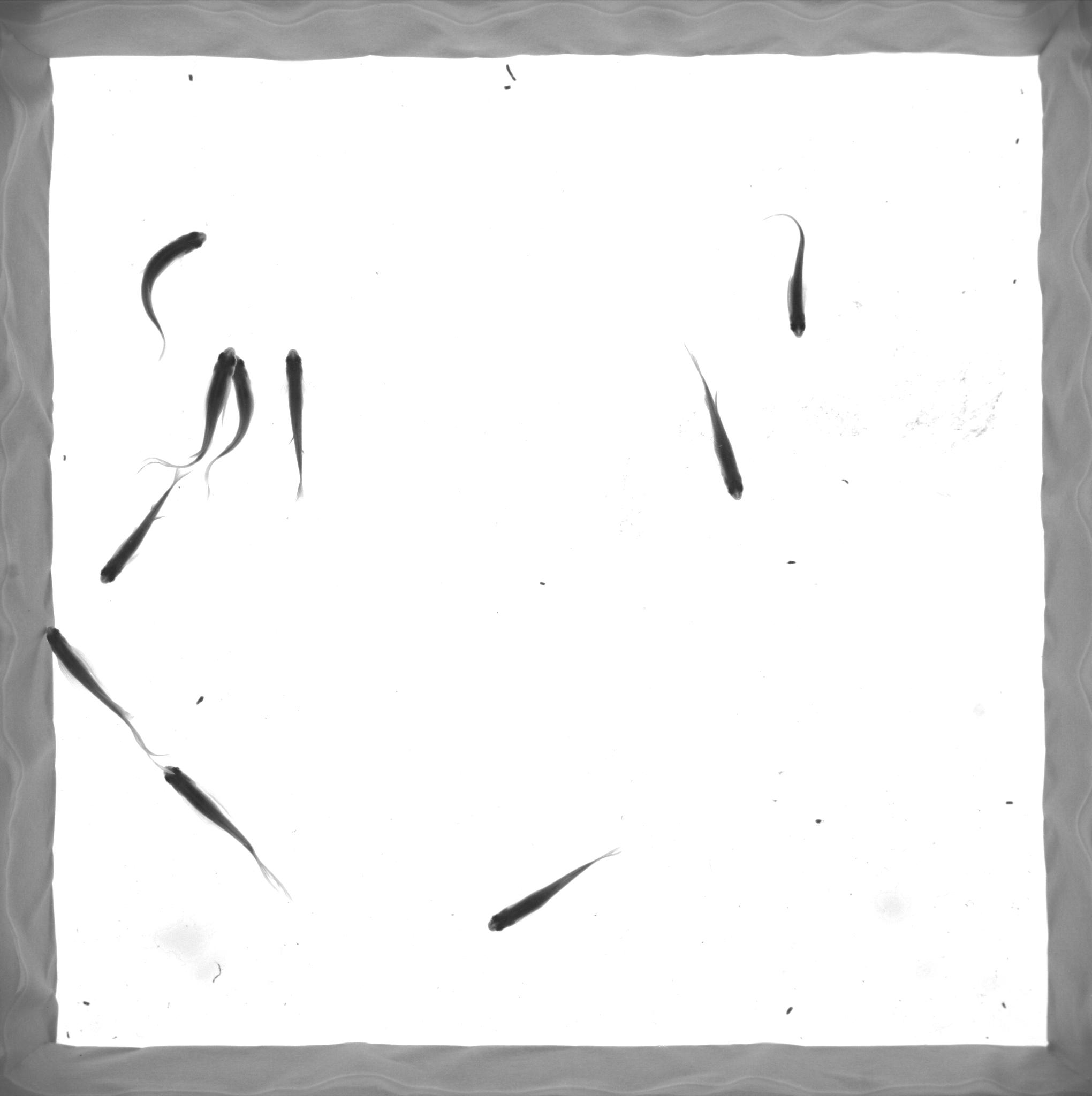

Supplement: S1 File — Source code of the proposed tracking system. (ZIP) [file pone.0154714.s002.zip › code_final/images/CoreView_275_Master_Camera_00125.jpg]

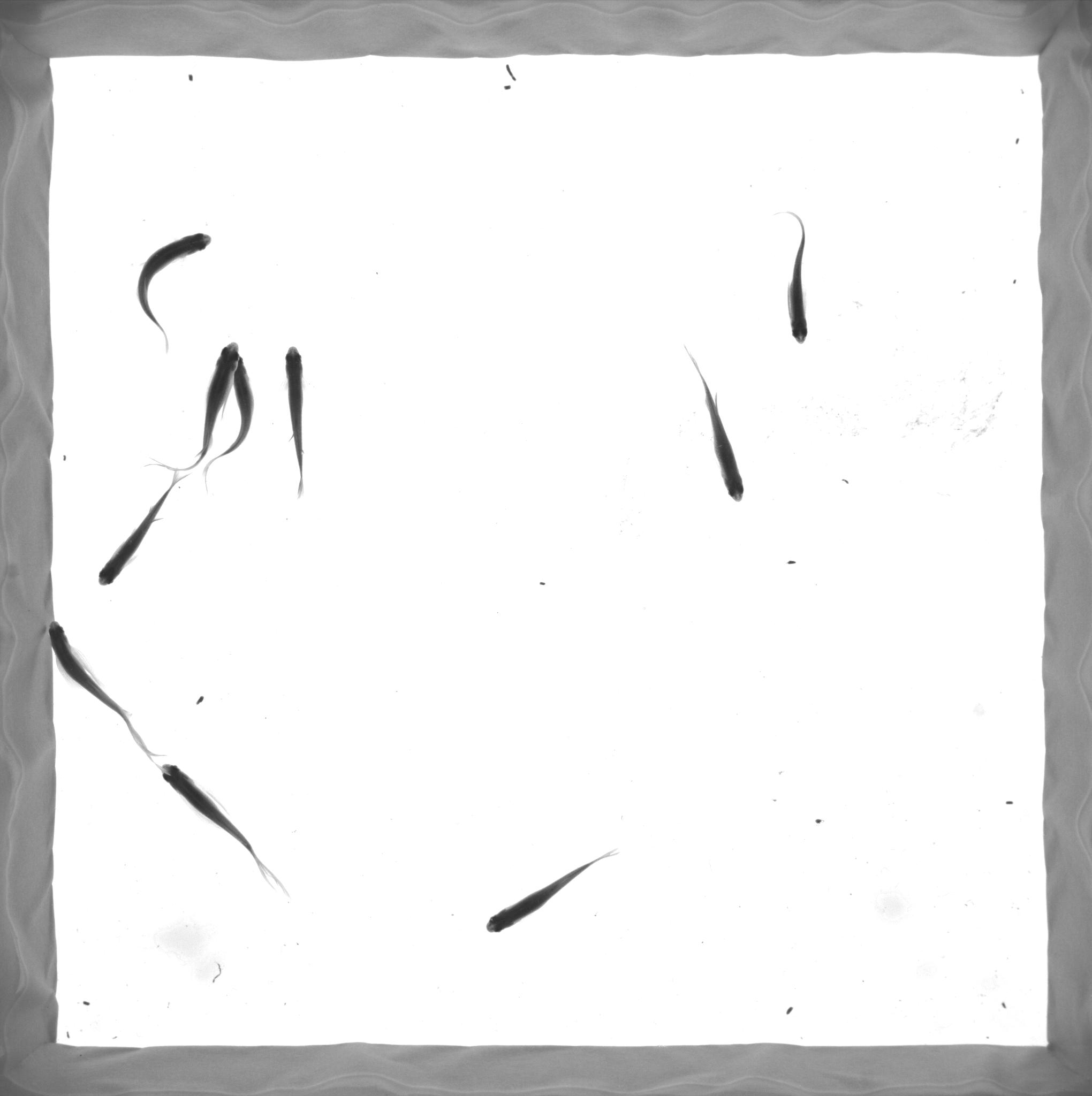

Supplement: S1 File — Source code of the proposed tracking system. (ZIP) [file pone.0154714.s002.zip › code_final/images/CoreView_275_Master_Camera_00126.jpg]

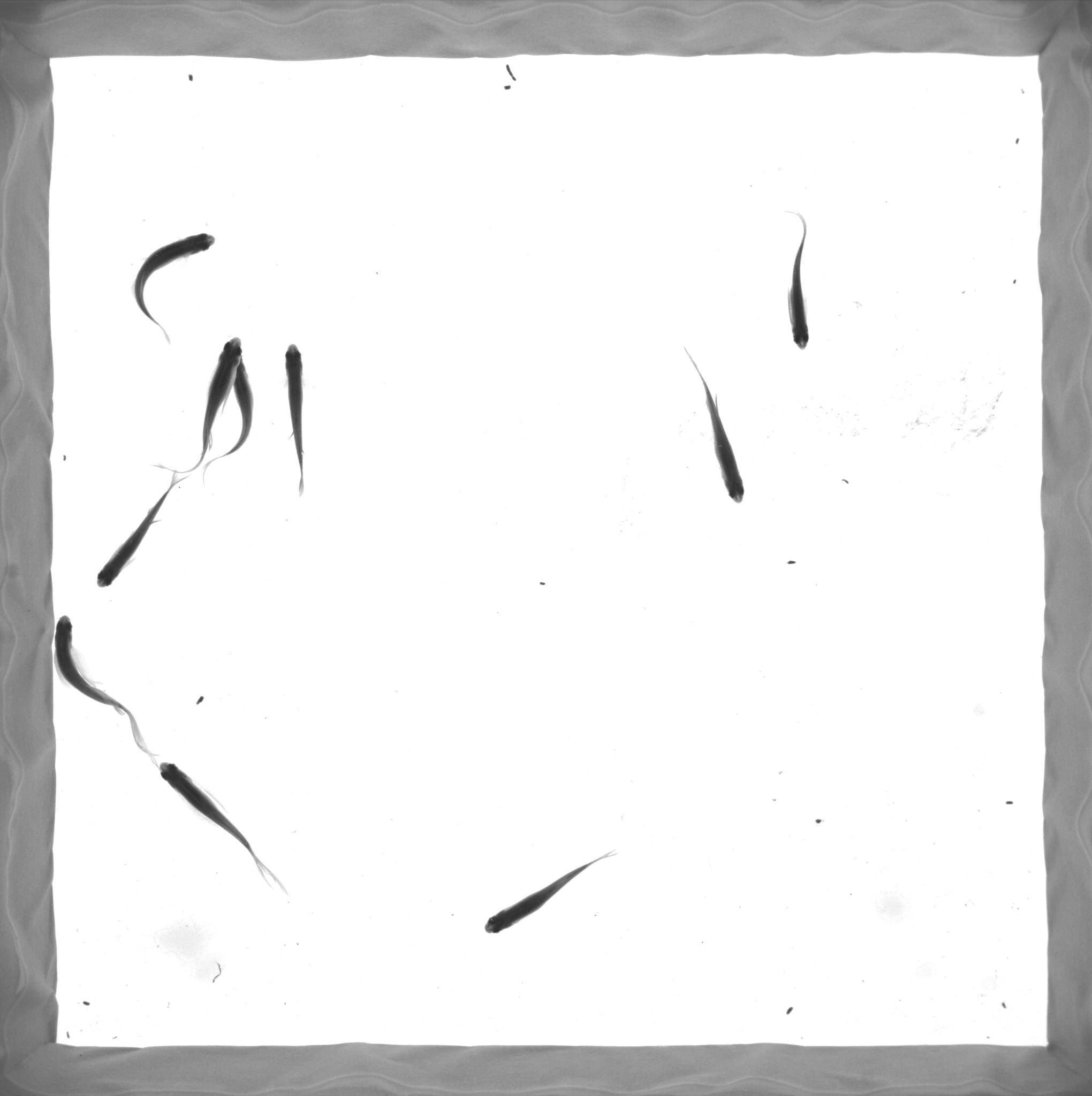

Supplement: S1 File — Source code of the proposed tracking system. (ZIP) [file pone.0154714.s002.zip › code_final/images/CoreView_275_Master_Camera_00127.jpg]

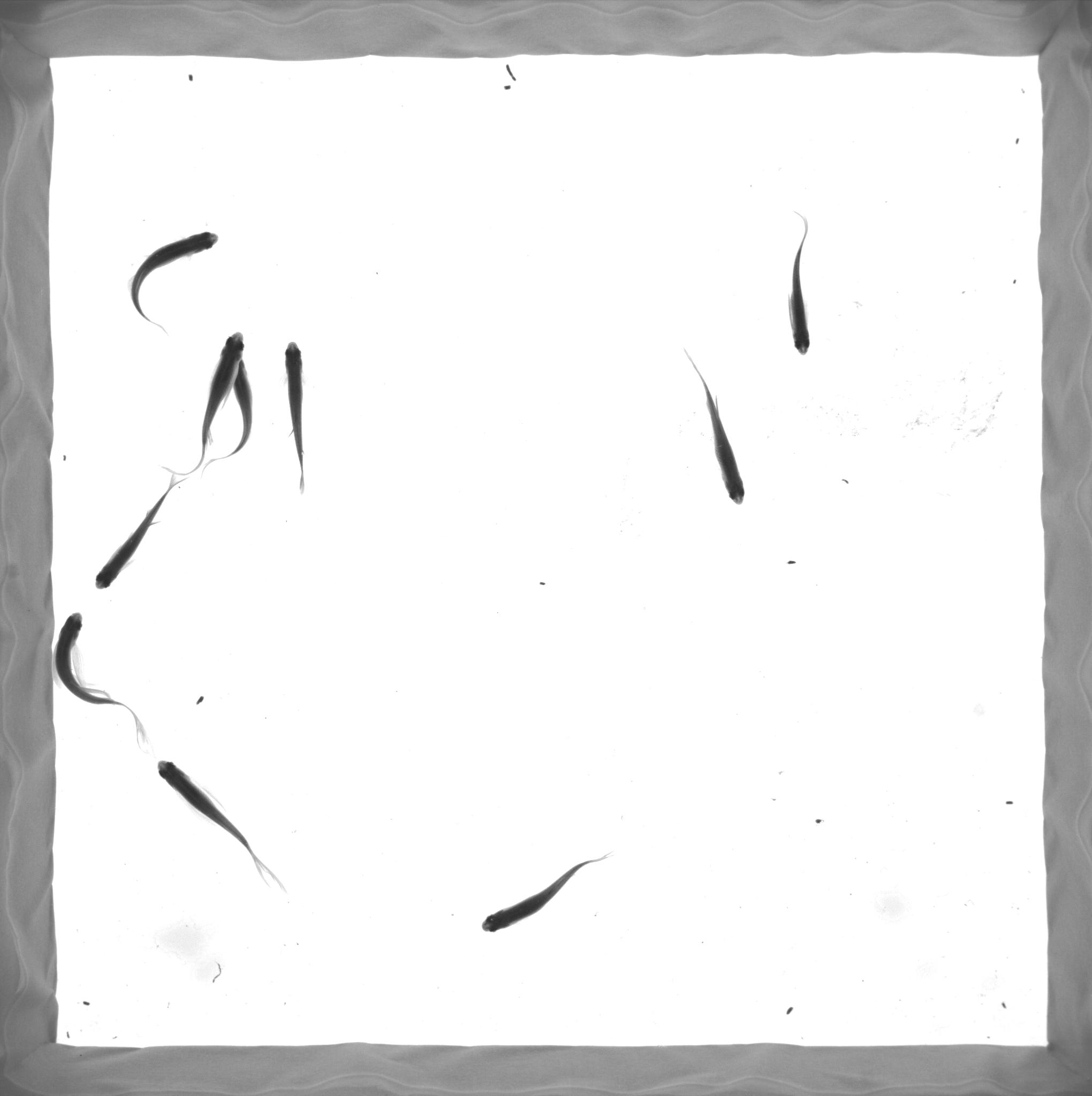

Supplement: S1 File — Source code of the proposed tracking system. (ZIP) [file pone.0154714.s002.zip › code_final/images/CoreView_275_Master_Camera_00128.jpg]

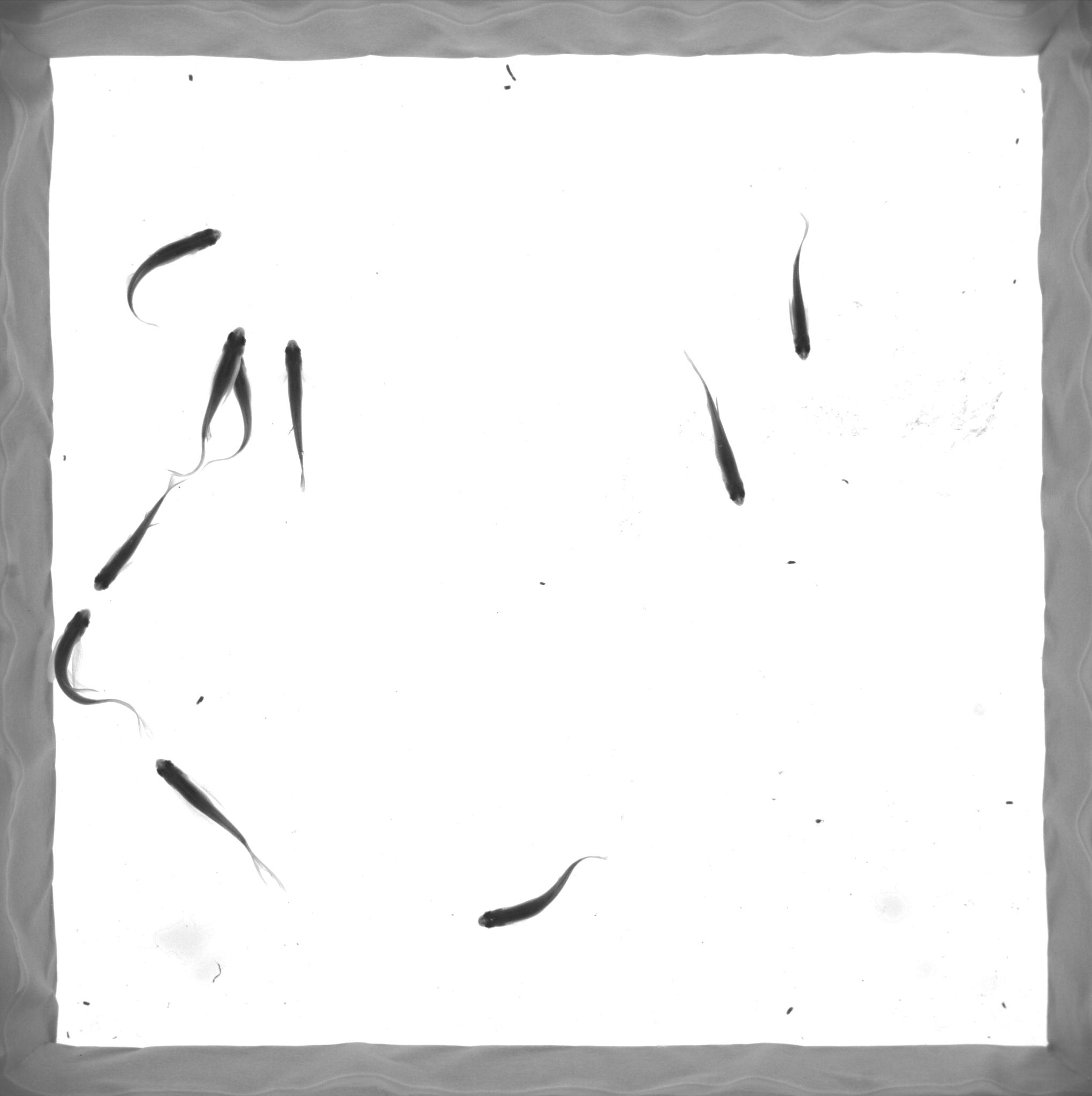

Supplement: S1 File — Source code of the proposed tracking system. (ZIP) [file pone.0154714.s002.zip › code_final/images/CoreView_275_Master_Camera_00129.jpg]

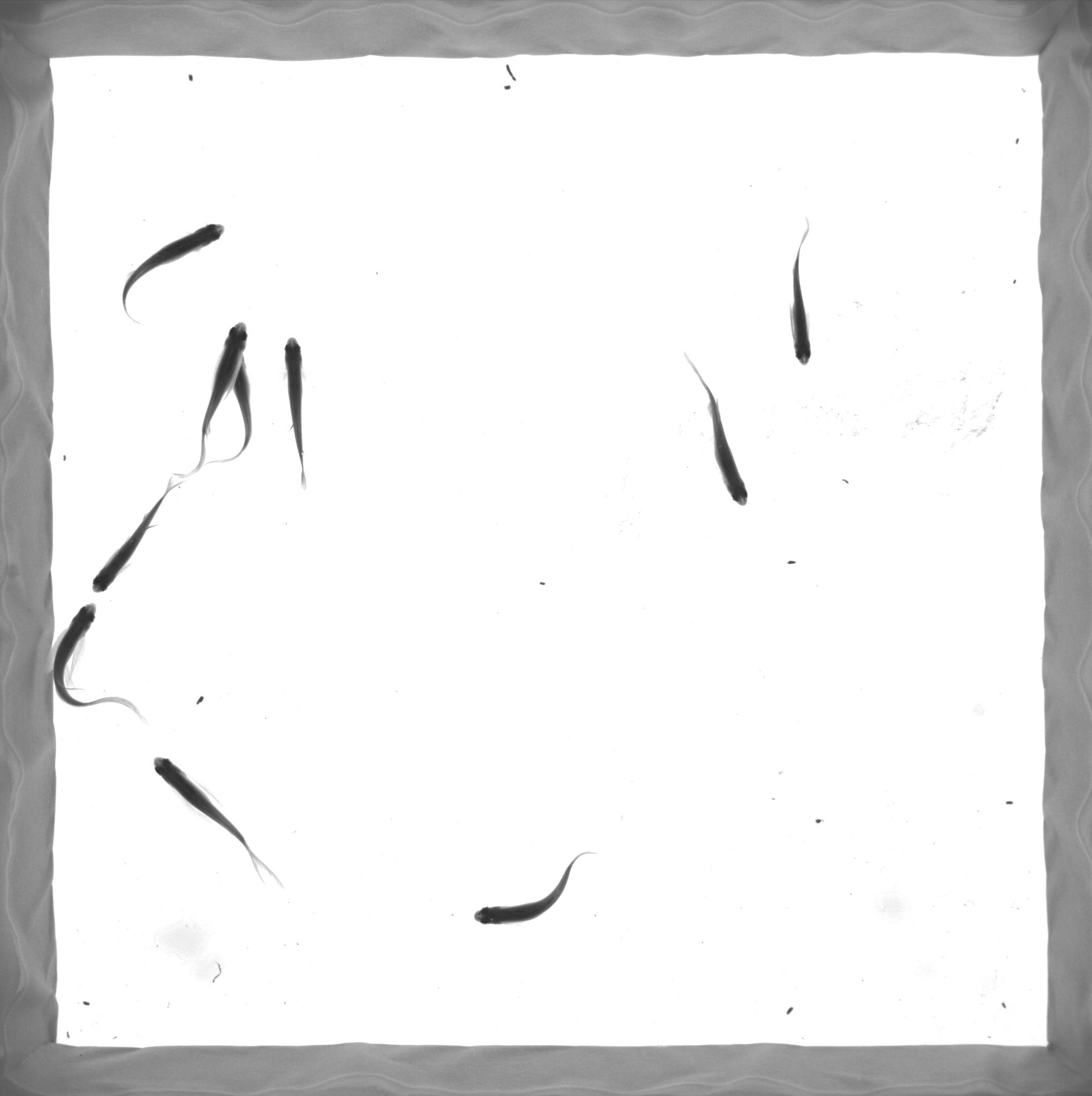

Supplement: S1 File — Source code of the proposed tracking system. (ZIP) [file pone.0154714.s002.zip › code_final/images/CoreView_275_Master_Camera_00130.jpg]

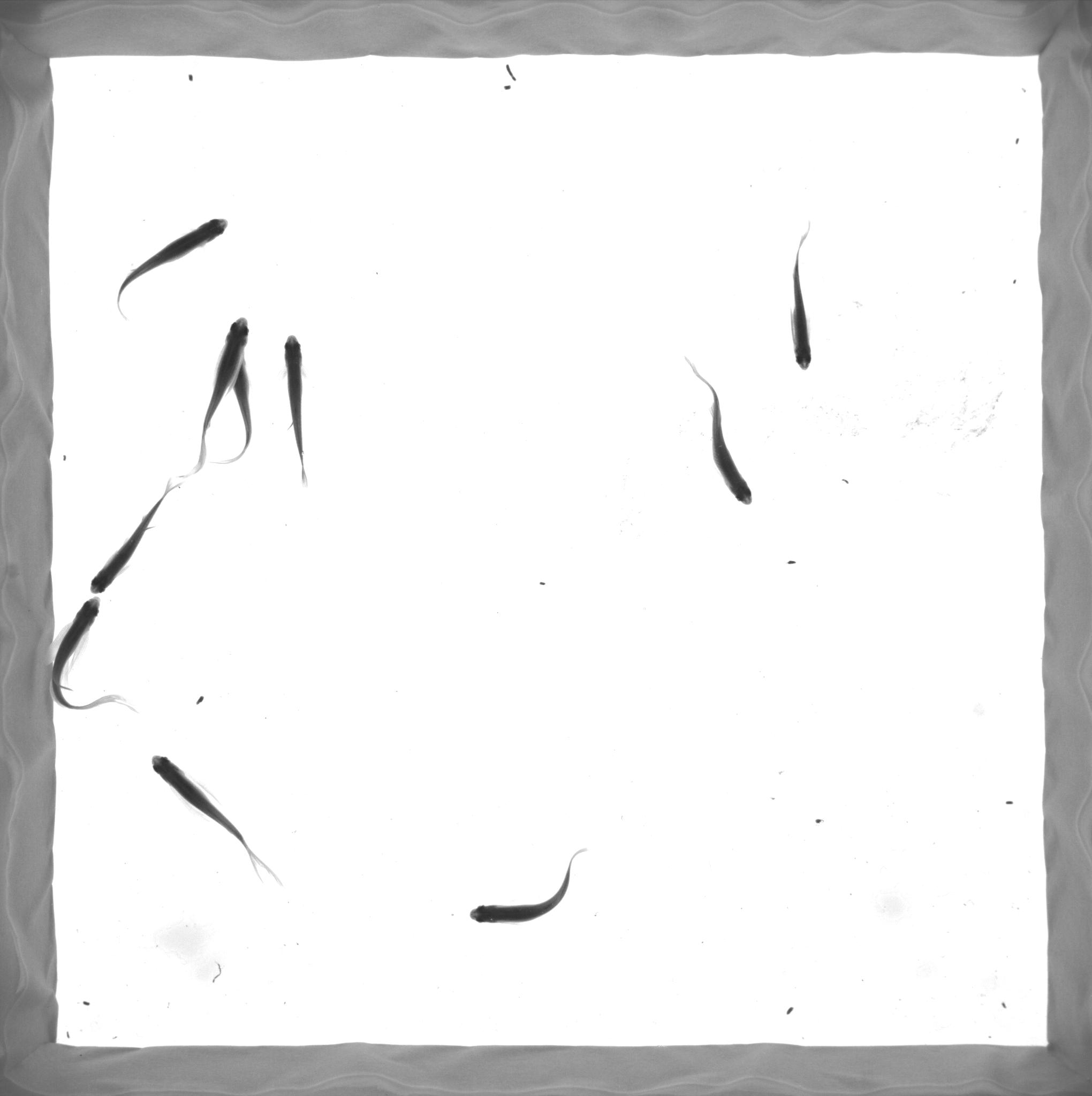

Supplement: S1 File — Source code of the proposed tracking system. (ZIP) [file pone.0154714.s002.zip › code_final/images/CoreView_275_Master_Camera_00131.jpg]

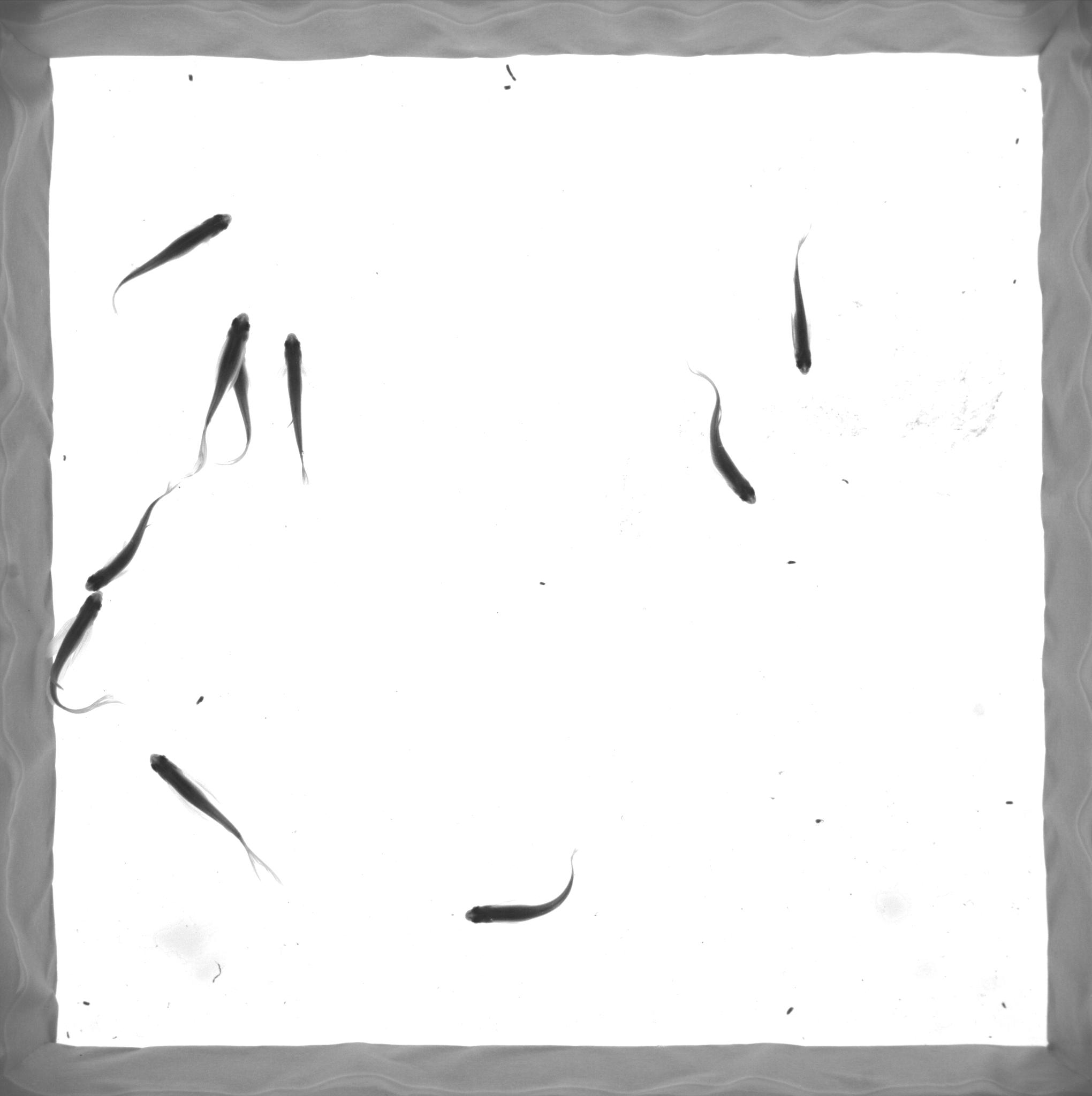

Supplement: S1 File — Source code of the proposed tracking system. (ZIP) [file pone.0154714.s002.zip › code_final/images/CoreView_275_Master_Camera_00132.jpg]

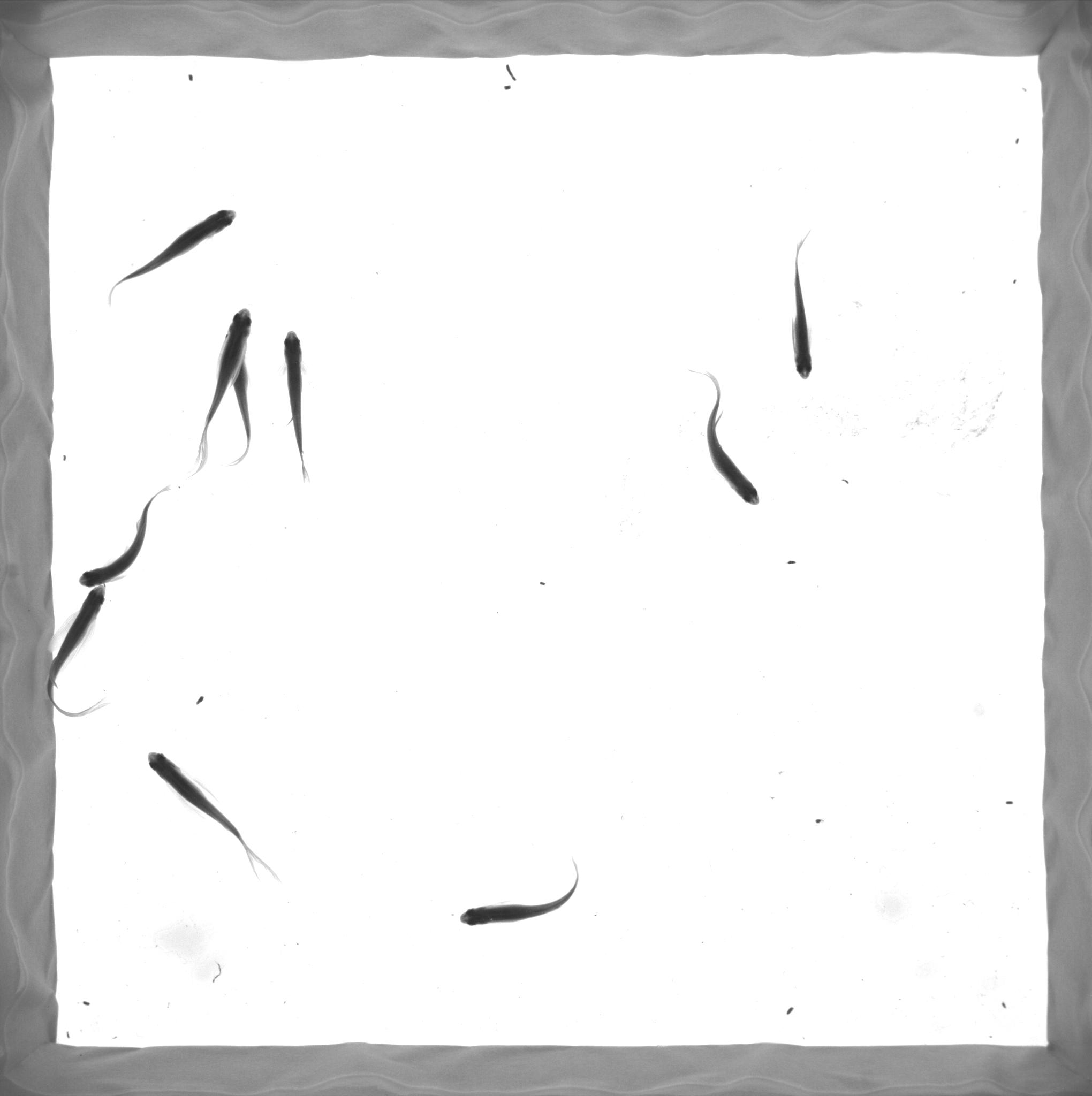

Supplement: S1 File — Source code of the proposed tracking system. (ZIP) [file pone.0154714.s002.zip › code_final/images/CoreView_275_Master_Camera_00133.jpg]

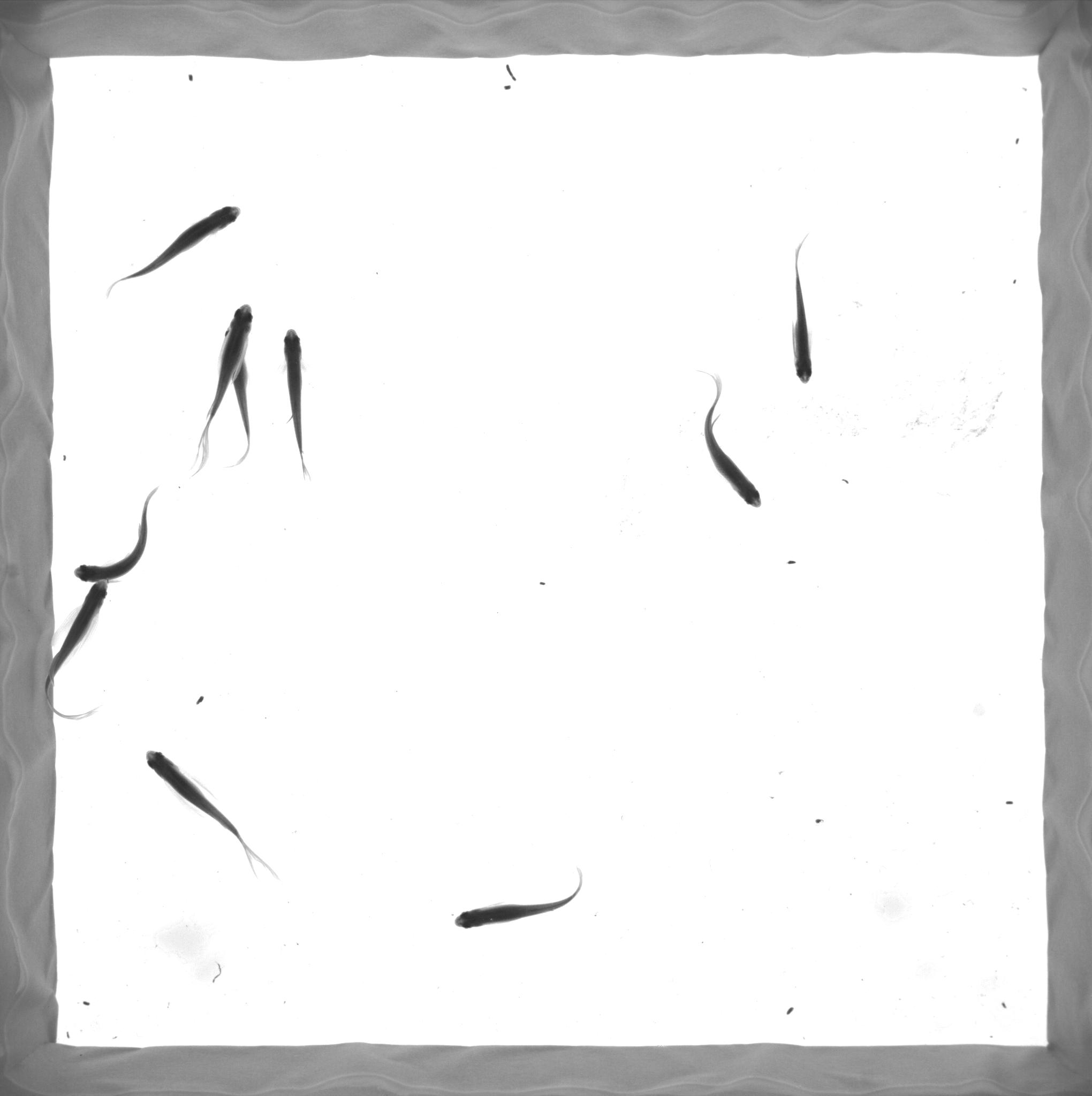

Supplement: S1 File — Source code of the proposed tracking system. (ZIP) [file pone.0154714.s002.zip › code_final/images/CoreView_275_Master_Camera_00134.jpg]

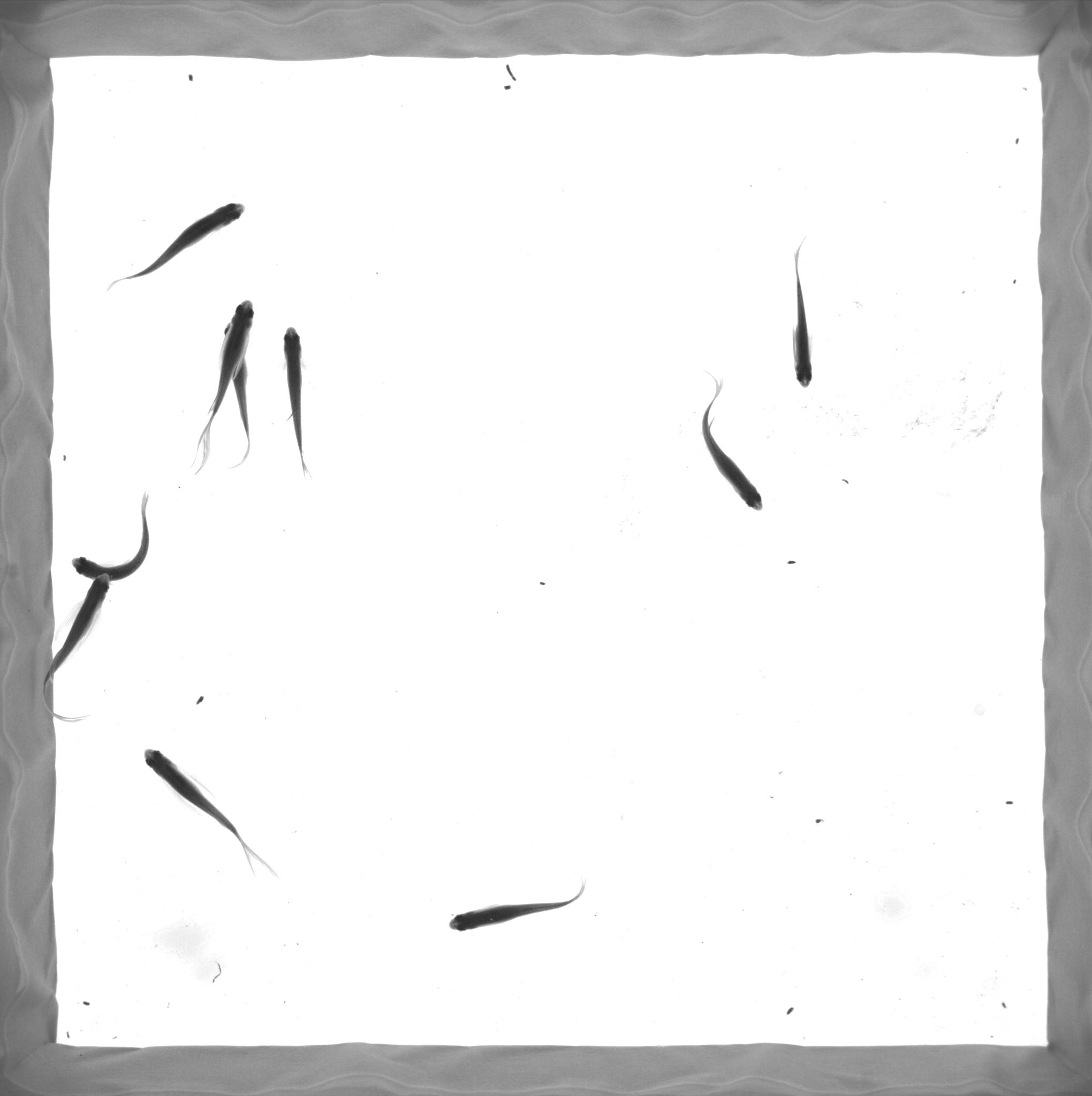

Supplement: S1 File — Source code of the proposed tracking system. (ZIP) [file pone.0154714.s002.zip › code_final/images/CoreView_275_Master_Camera_00135.jpg]

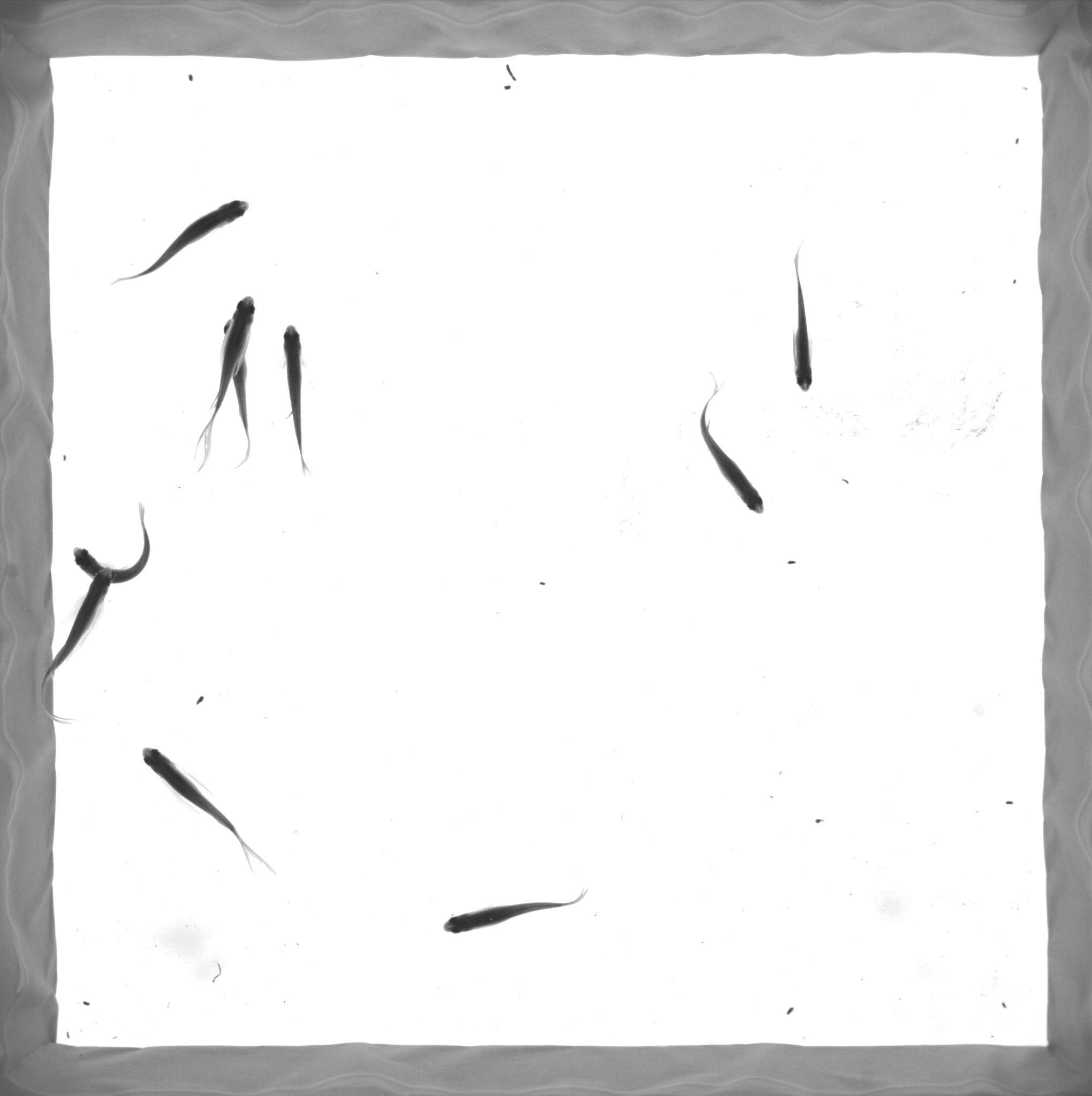

Supplement: S1 File — Source code of the proposed tracking system. (ZIP) [file pone.0154714.s002.zip › code_final/images/CoreView_275_Master_Camera_00136.jpg]

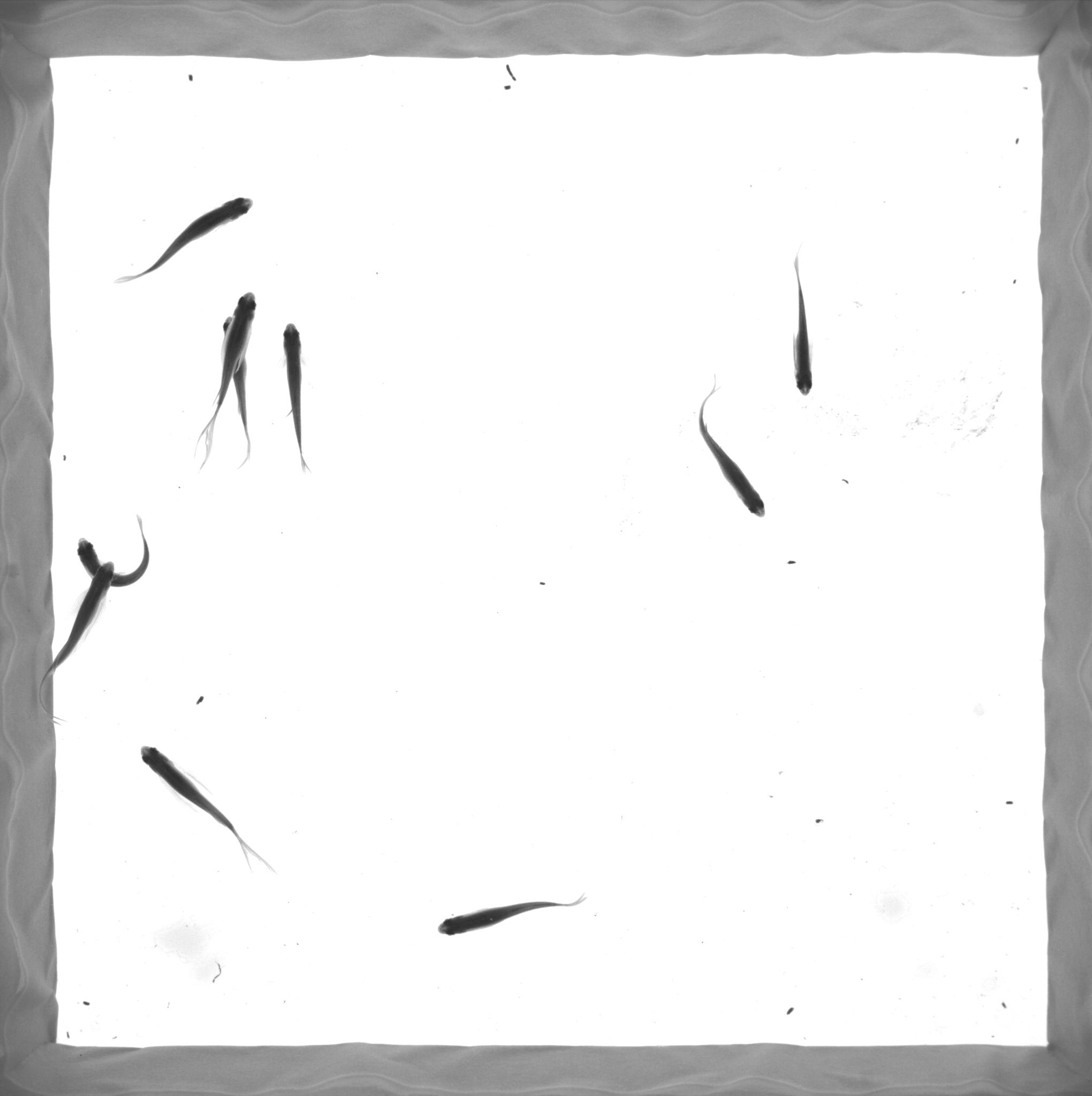

Supplement: S1 File — Source code of the proposed tracking system. (ZIP) [file pone.0154714.s002.zip › code_final/images/CoreView_275_Master_Camera_00137.jpg]

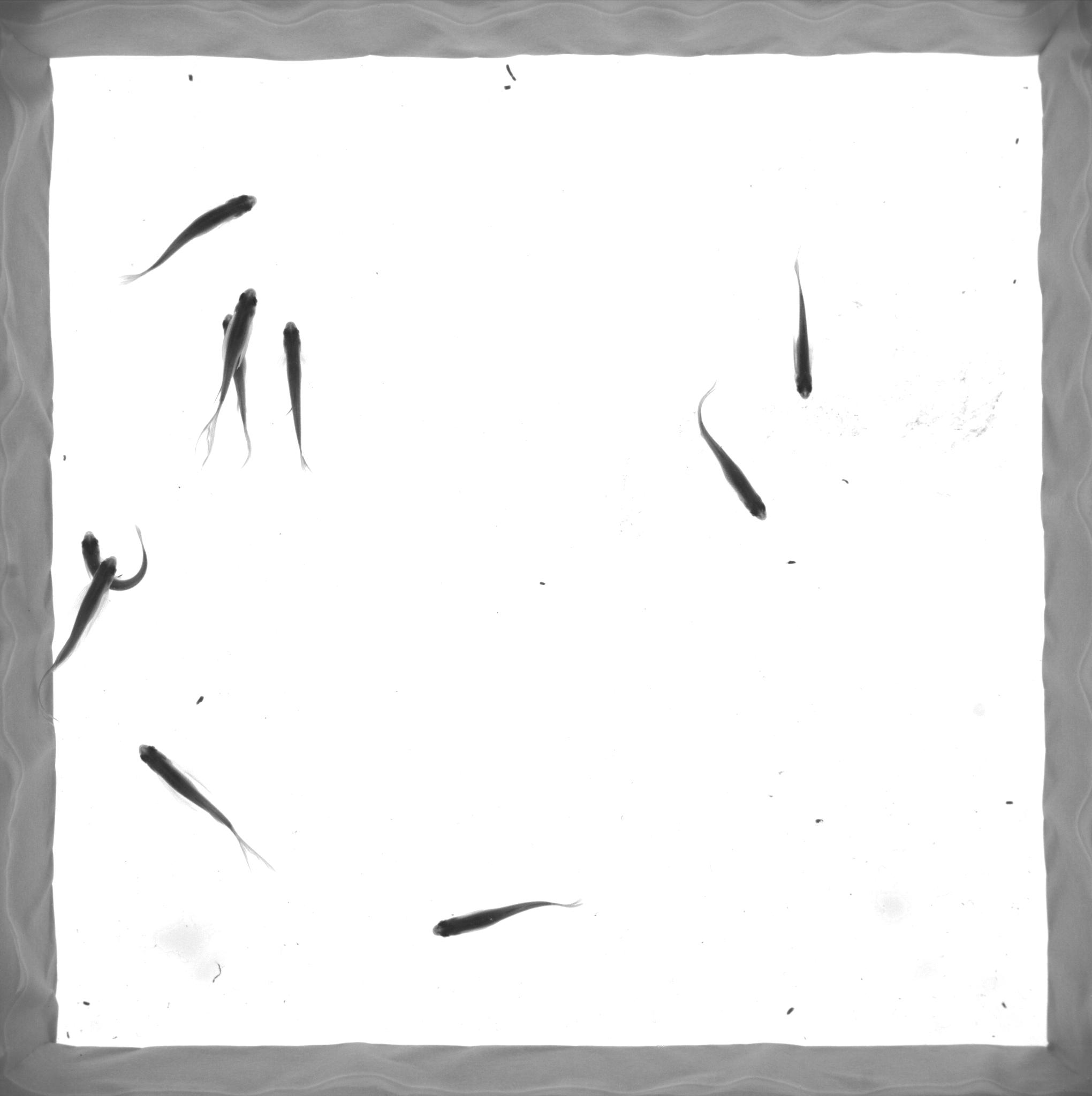

Supplement: S1 File — Source code of the proposed tracking system. (ZIP) [file pone.0154714.s002.zip › code_final/images/CoreView_275_Master_Camera_00138.jpg]

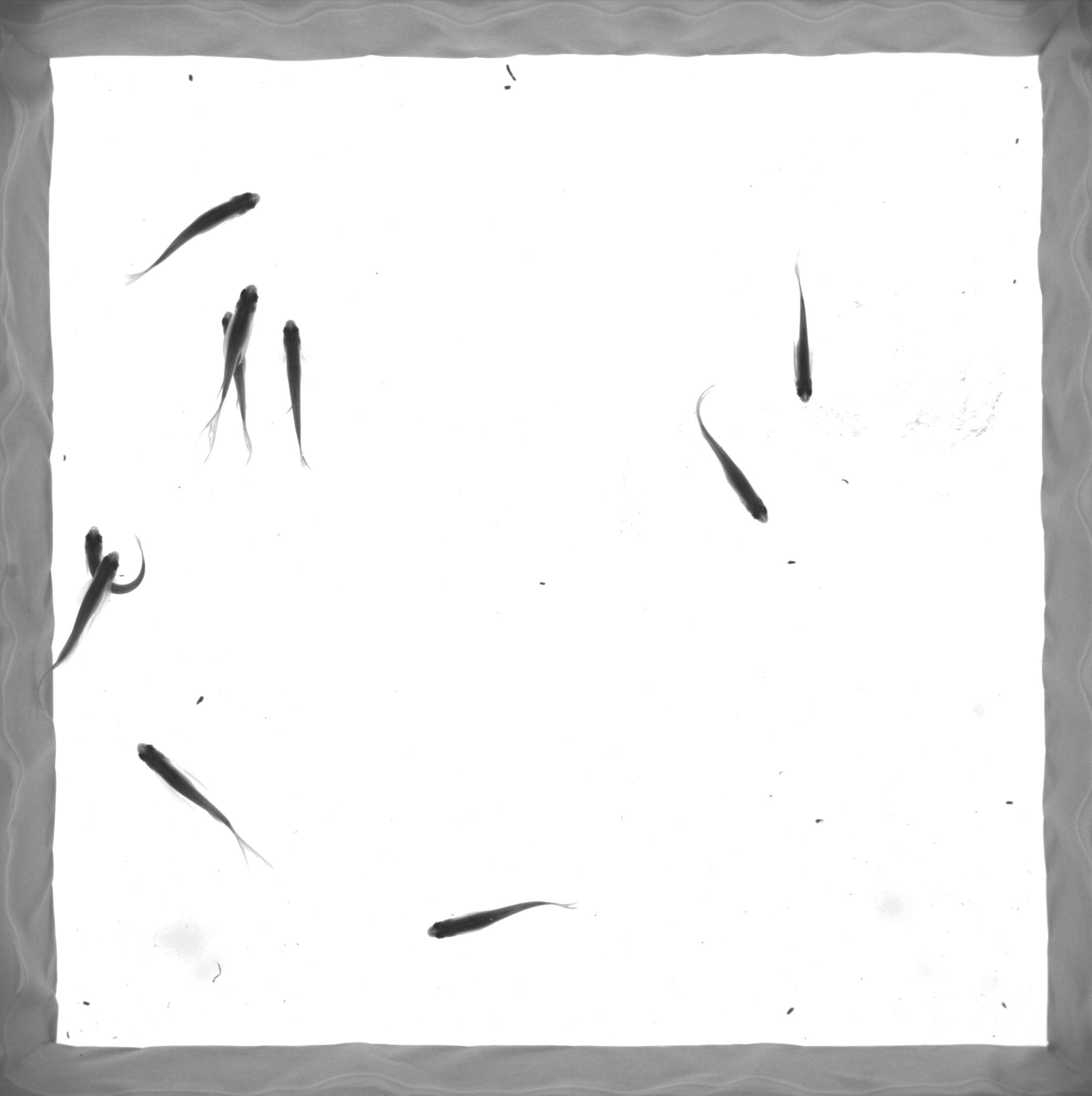

Supplement: S1 File — Source code of the proposed tracking system. (ZIP) [file pone.0154714.s002.zip › code_final/images/CoreView_275_Master_Camera_00139.jpg]

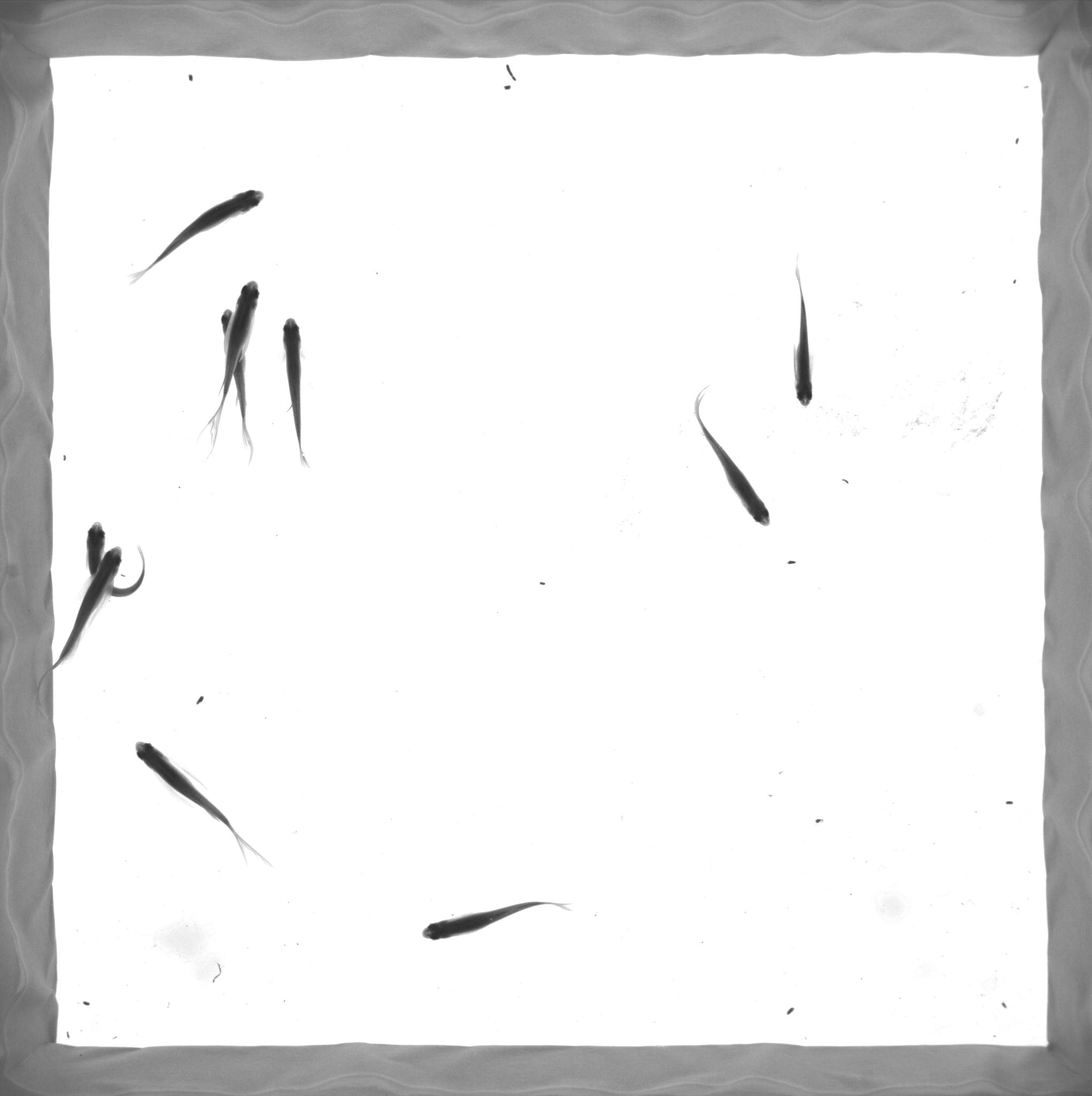

Supplement: S1 File — Source code of the proposed tracking system. (ZIP) [file pone.0154714.s002.zip › code_final/images/CoreView_275_Master_Camera_00140.jpg]

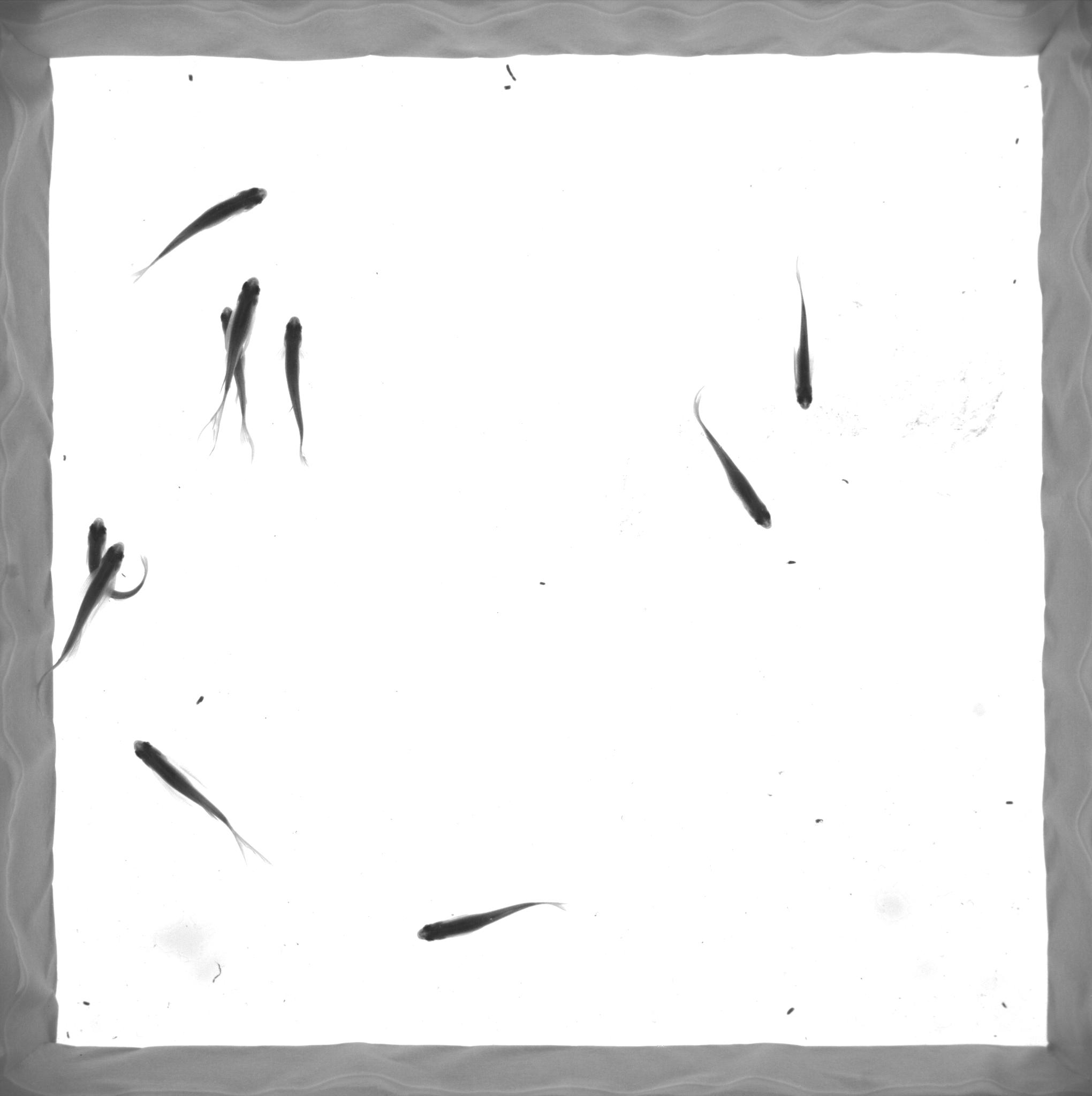

Supplement: S1 File — Source code of the proposed tracking system. (ZIP) [file pone.0154714.s002.zip › code_final/images/CoreView_275_Master_Camera_00141.jpg]

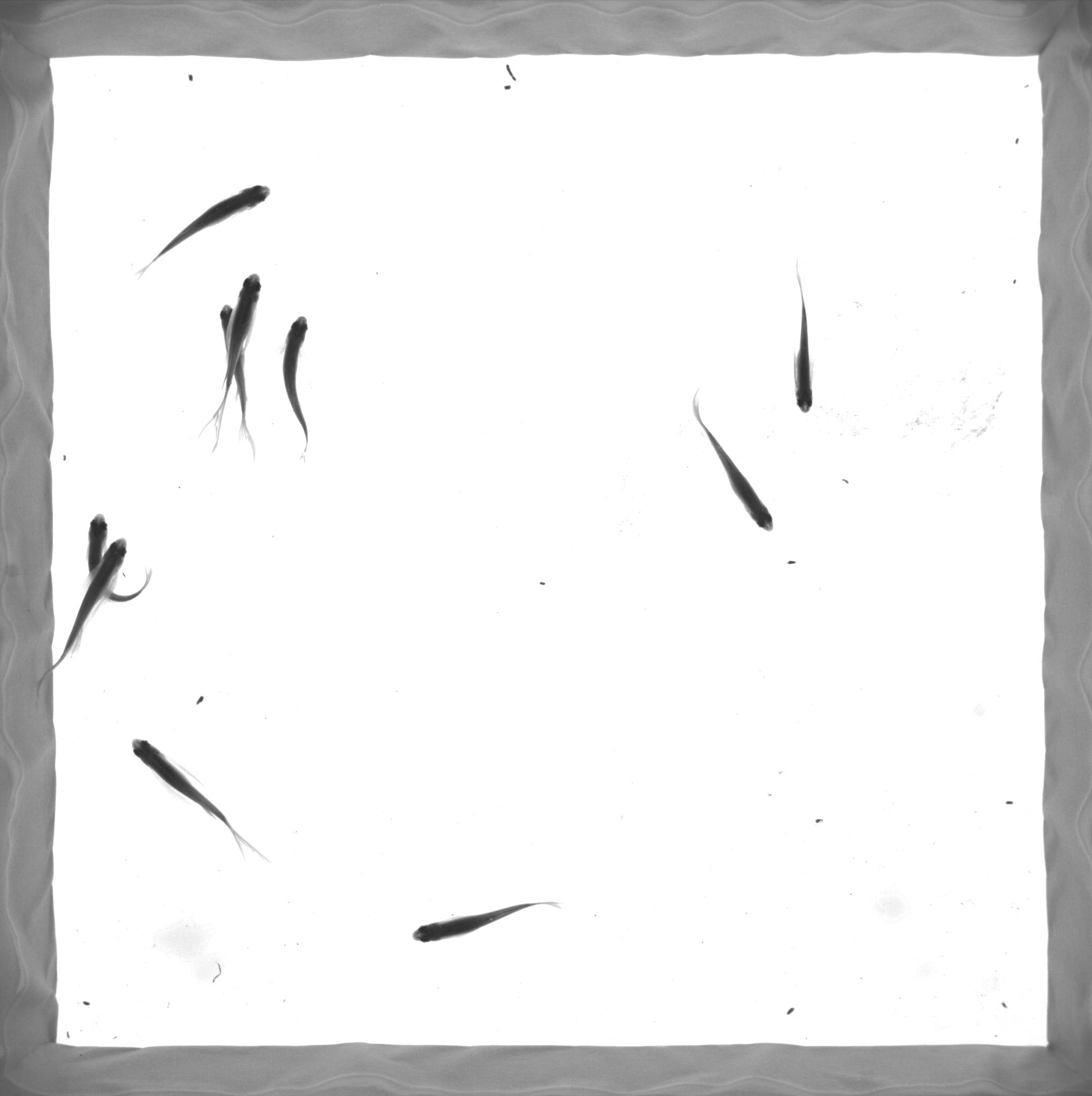

Supplement: S1 File — Source code of the proposed tracking system. (ZIP) [file pone.0154714.s002.zip › code_final/images/CoreView_275_Master_Camera_00142.jpg]

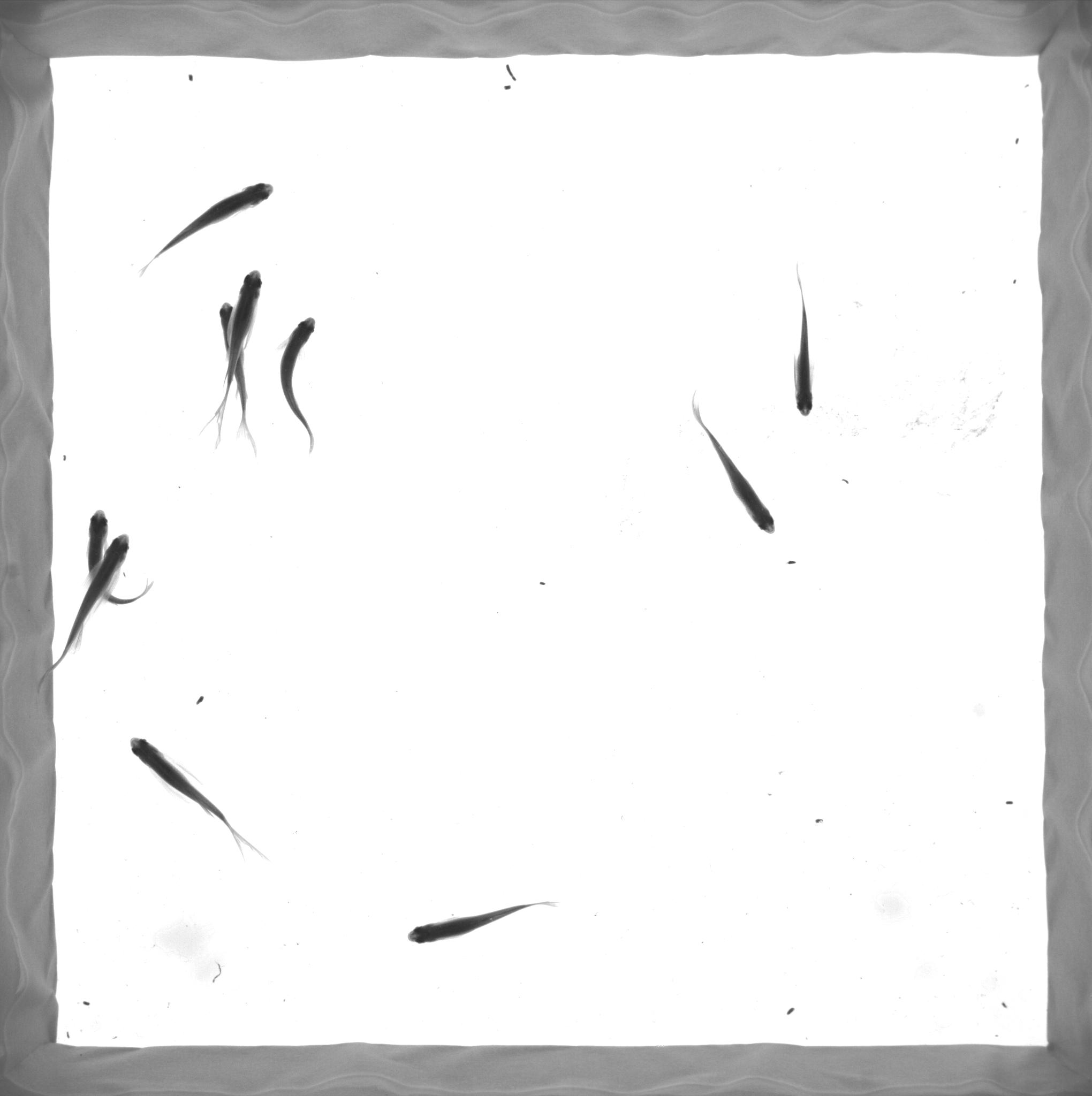

Supplement: S1 File — Source code of the proposed tracking system. (ZIP) [file pone.0154714.s002.zip › code_final/images/CoreView_275_Master_Camera_00143.jpg]

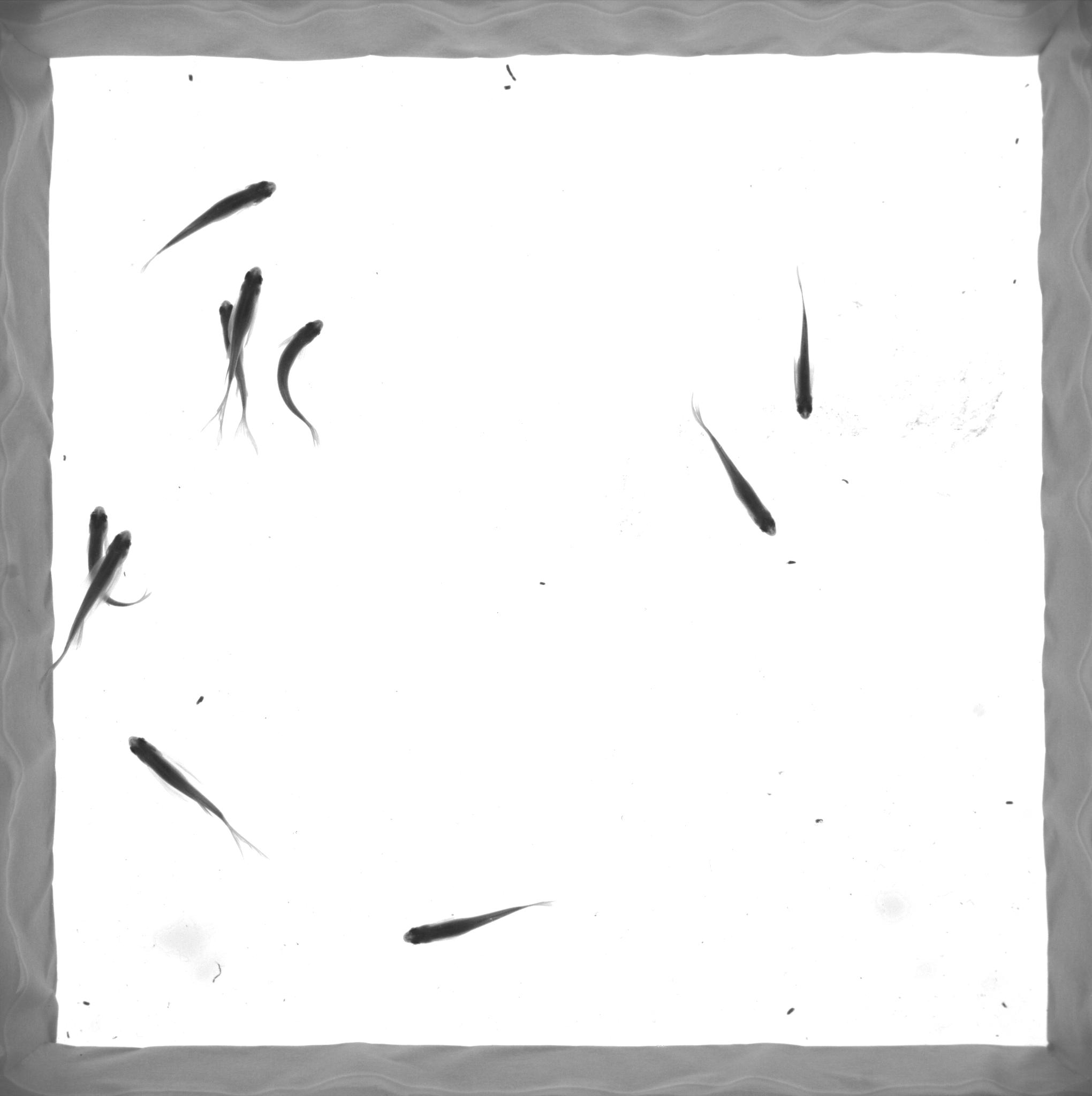

Supplement: S1 File — Source code of the proposed tracking system. (ZIP) [file pone.0154714.s002.zip › code_final/images/CoreView_275_Master_Camera_00144.jpg]

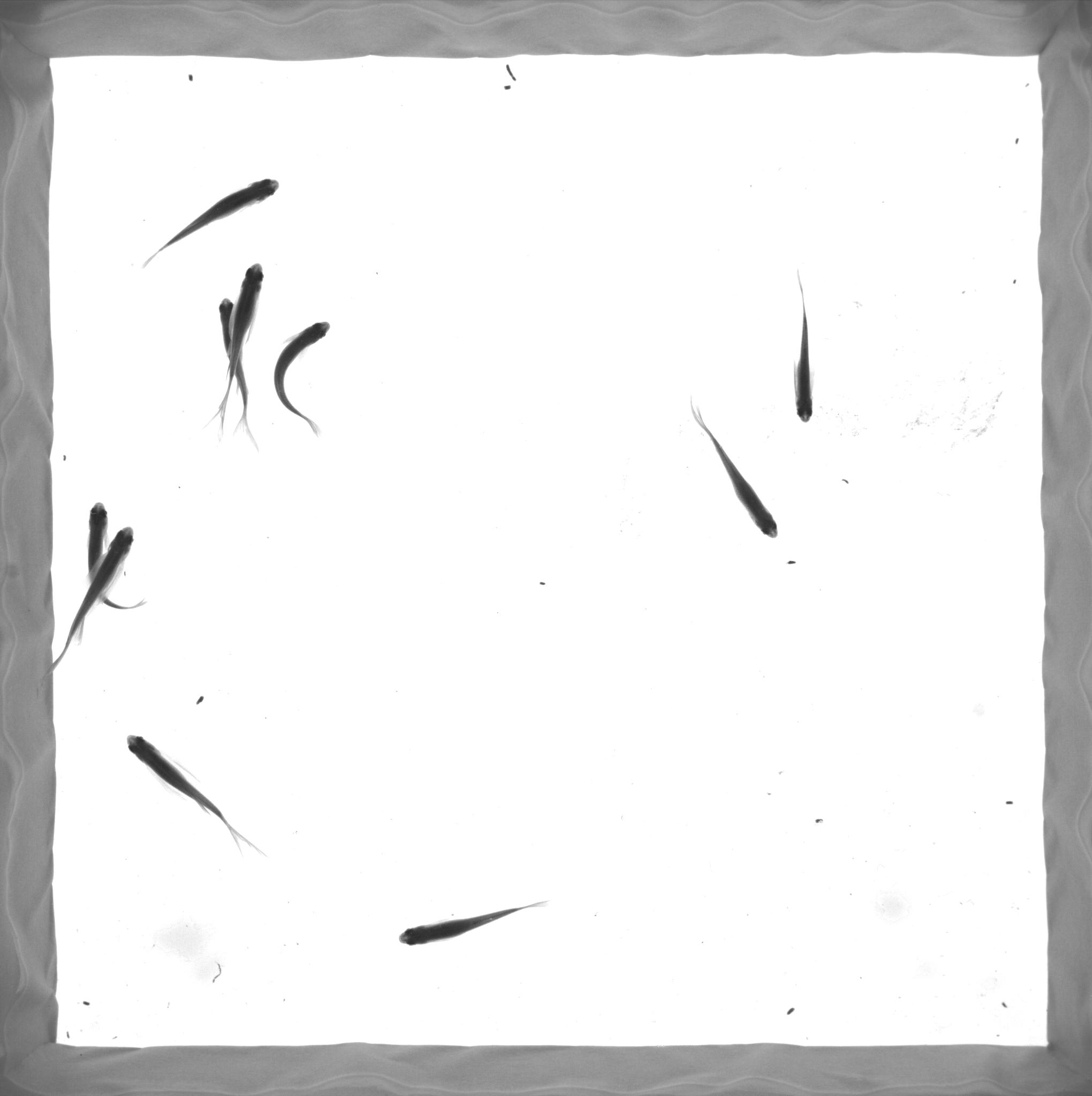

Supplement: S1 File — Source code of the proposed tracking system. (ZIP) [file pone.0154714.s002.zip › code_final/images/CoreView_275_Master_Camera_00145.jpg]

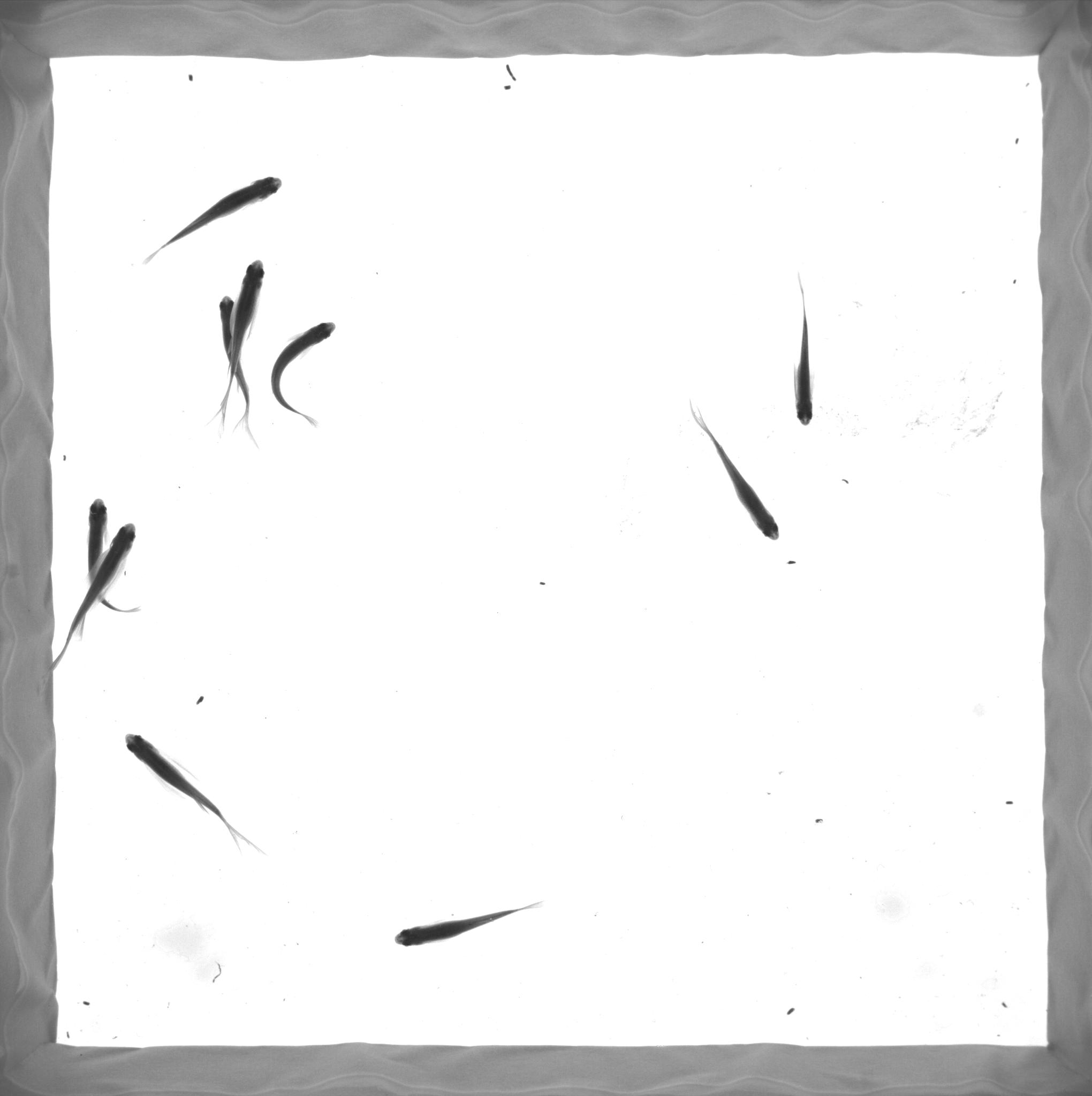

Supplement: S1 File — Source code of the proposed tracking system. (ZIP) [file pone.0154714.s002.zip › code_final/images/CoreView_275_Master_Camera_00146.jpg]

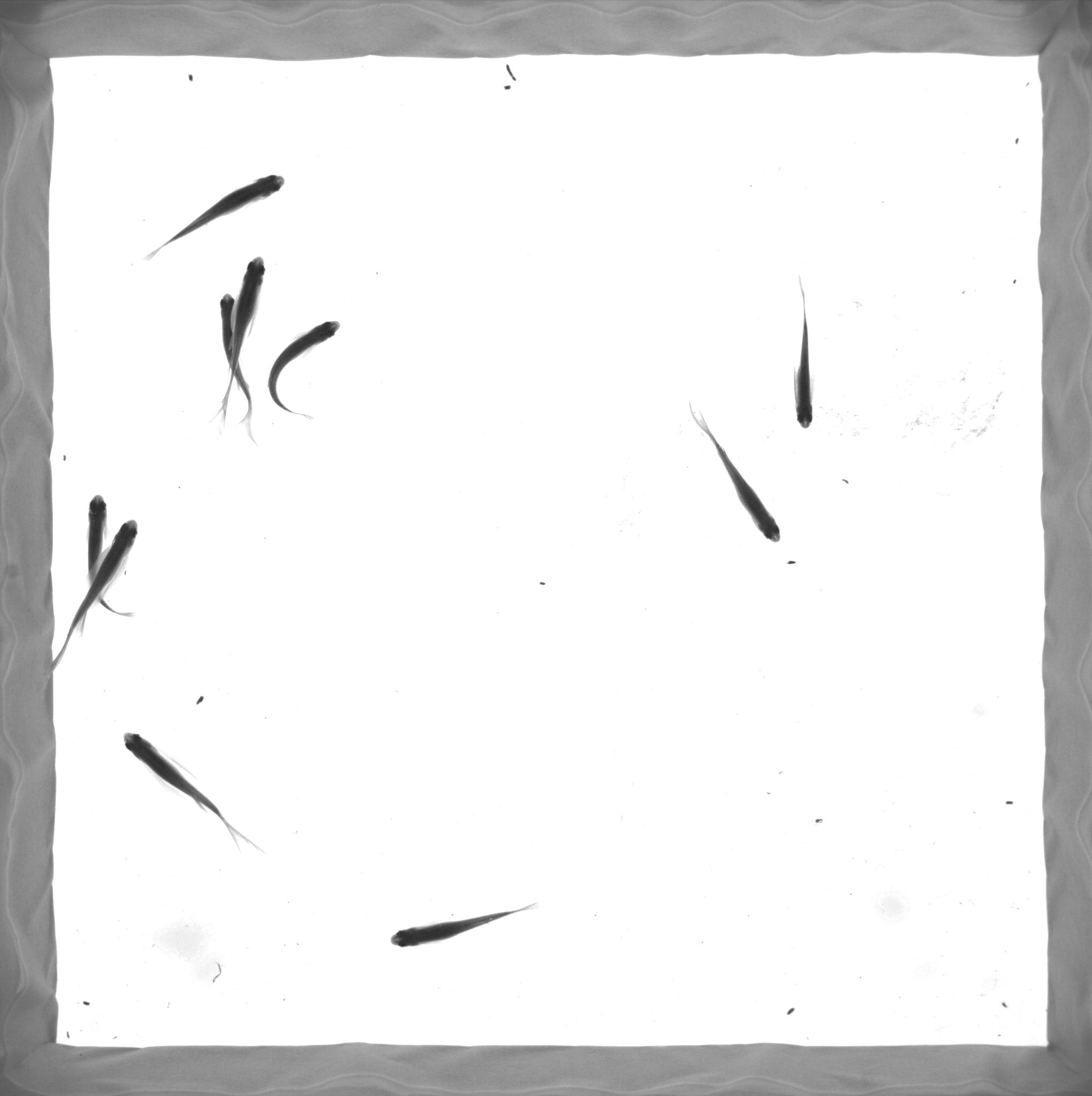

Supplement: S1 File — Source code of the proposed tracking system. (ZIP) [file pone.0154714.s002.zip › code_final/images/CoreView_275_Master_Camera_00147.jpg]

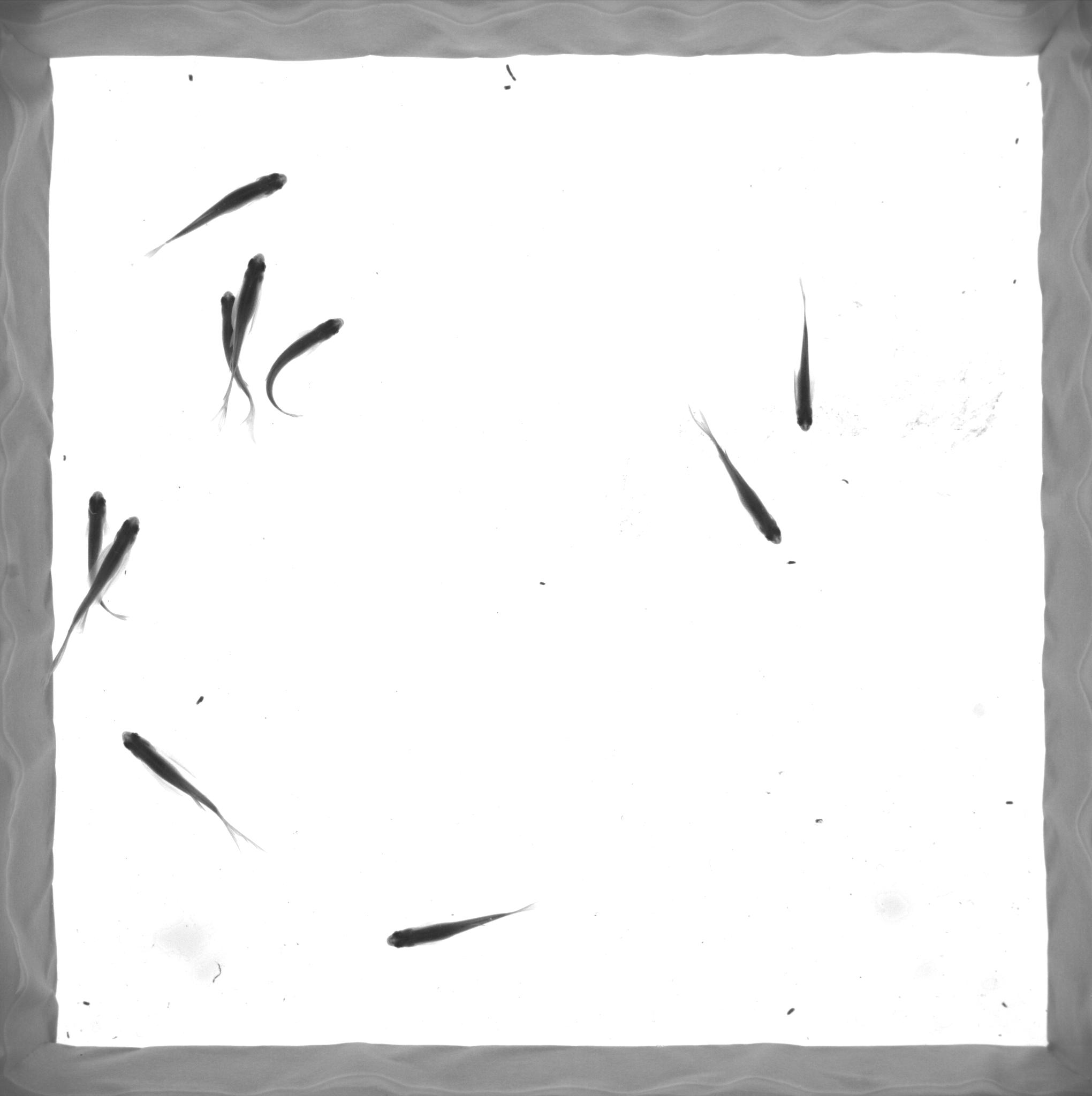

Supplement: S1 File — Source code of the proposed tracking system. (ZIP) [file pone.0154714.s002.zip › code_final/images/CoreView_275_Master_Camera_00148.jpg]

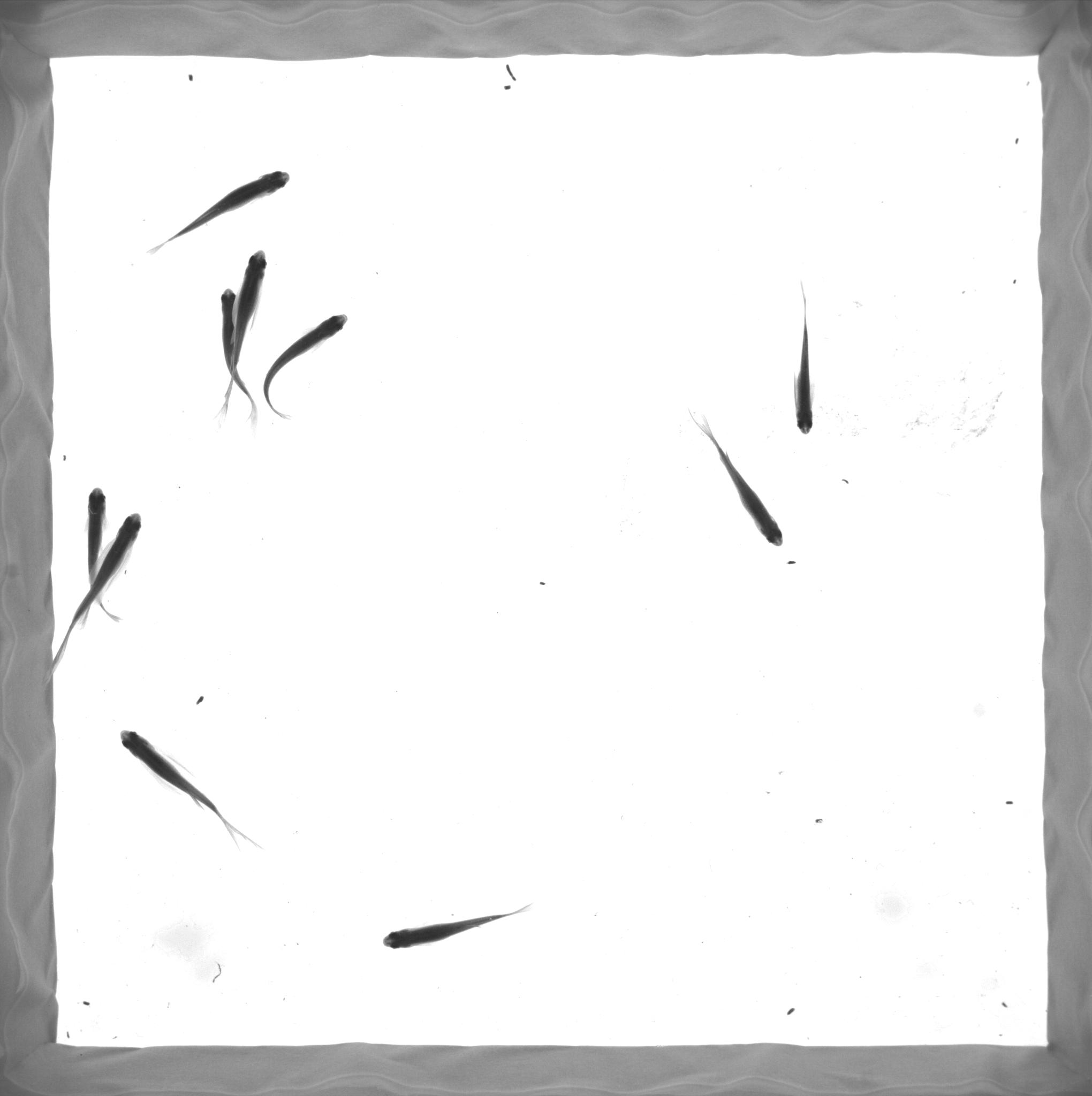

Supplement: S1 File — Source code of the proposed tracking system. (ZIP) [file pone.0154714.s002.zip › code_final/images/CoreView_275_Master_Camera_00149.jpg]

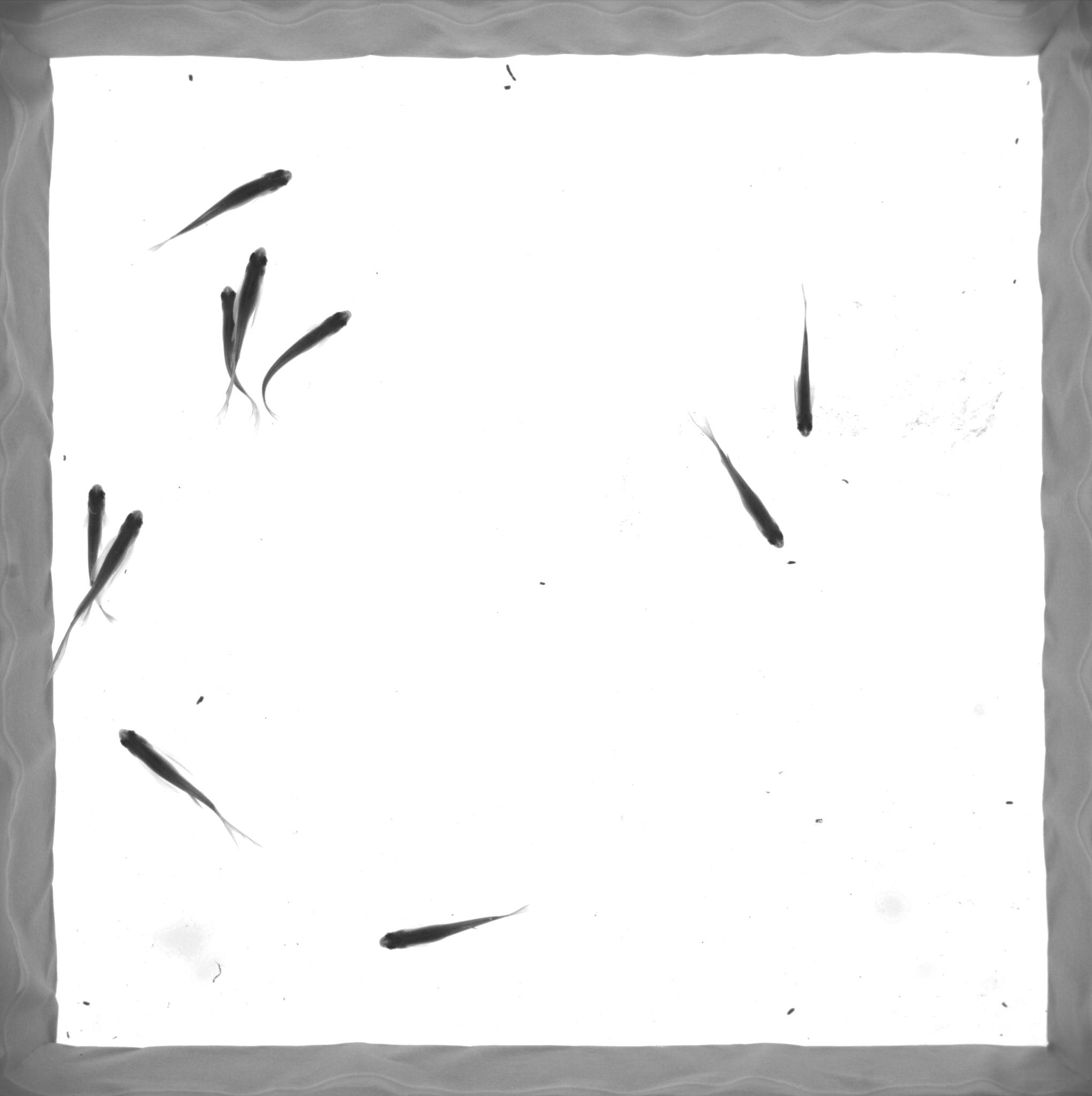

Supplement: S1 File — Source code of the proposed tracking system. (ZIP) [file pone.0154714.s002.zip › code_final/images/CoreView_275_Master_Camera_00150.jpg]

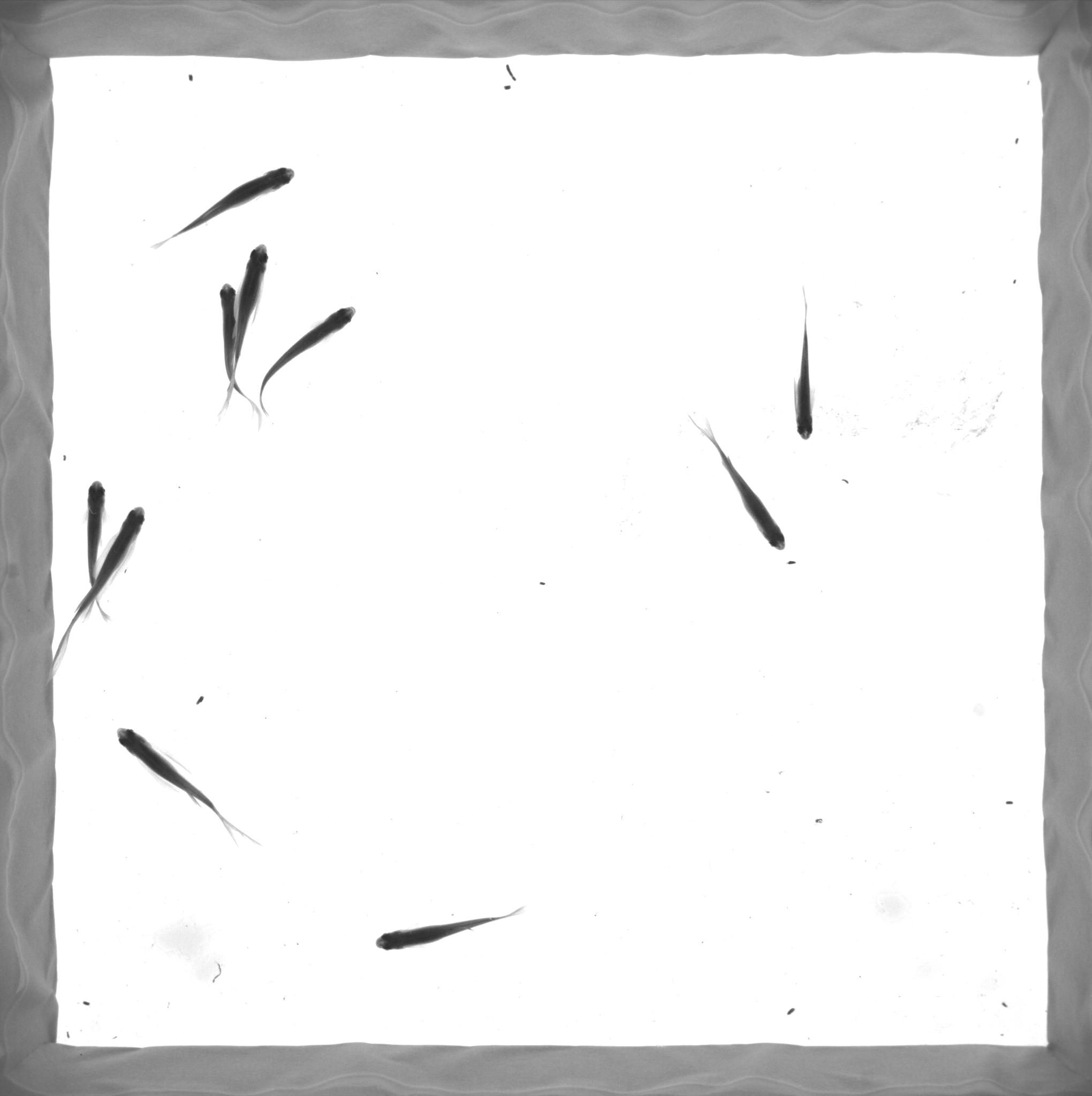

Supplement: S1 File — Source code of the proposed tracking system. (ZIP) [file pone.0154714.s002.zip › code_final/images/CoreView_275_Master_Camera_00151.jpg]

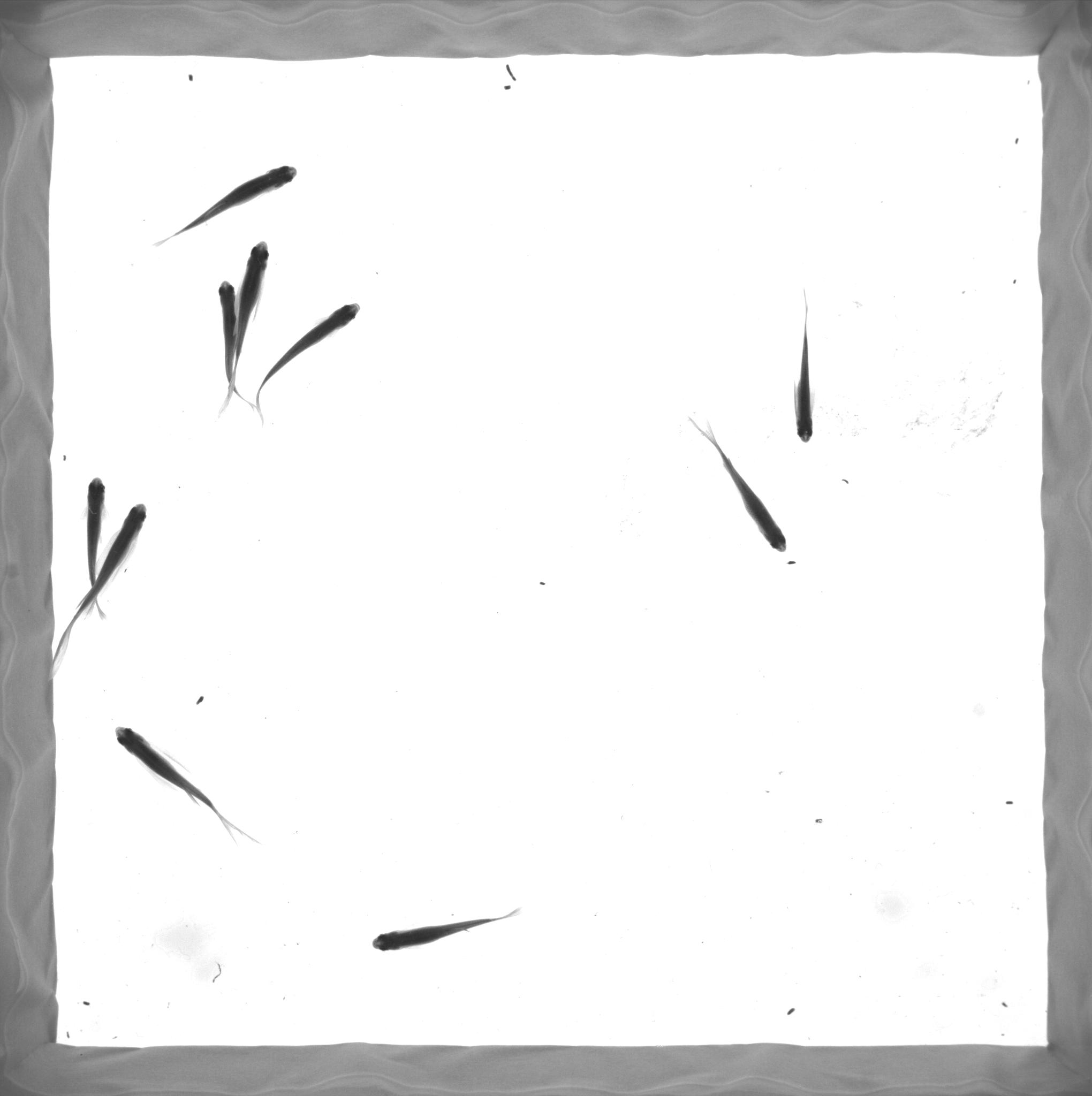

Supplement: S1 File — Source code of the proposed tracking system. (ZIP) [file pone.0154714.s002.zip › code_final/images/CoreView_275_Master_Camera_00152.jpg]

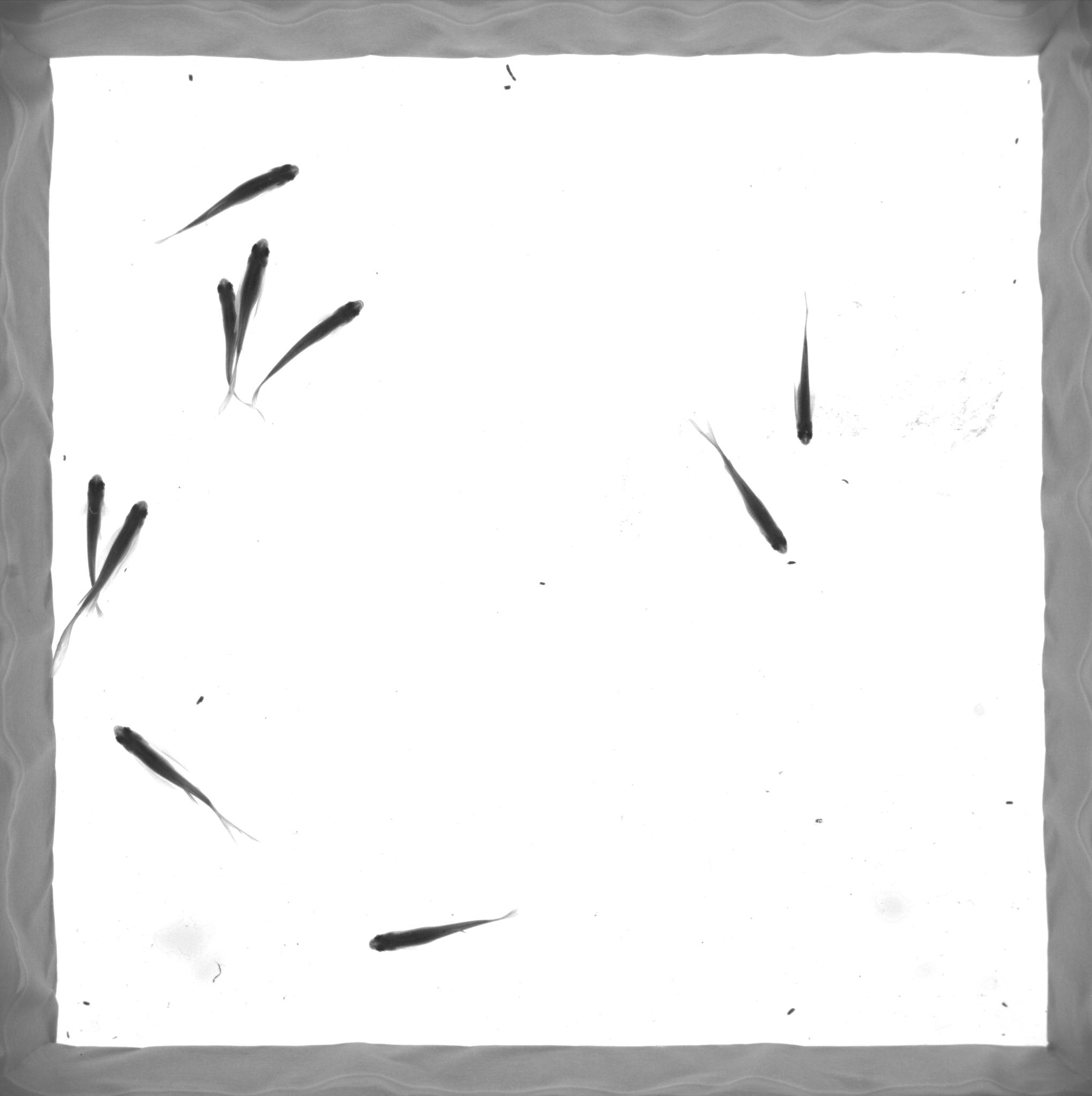

Supplement: S1 File — Source code of the proposed tracking system. (ZIP) [file pone.0154714.s002.zip › code_final/images/CoreView_275_Master_Camera_00153.jpg]

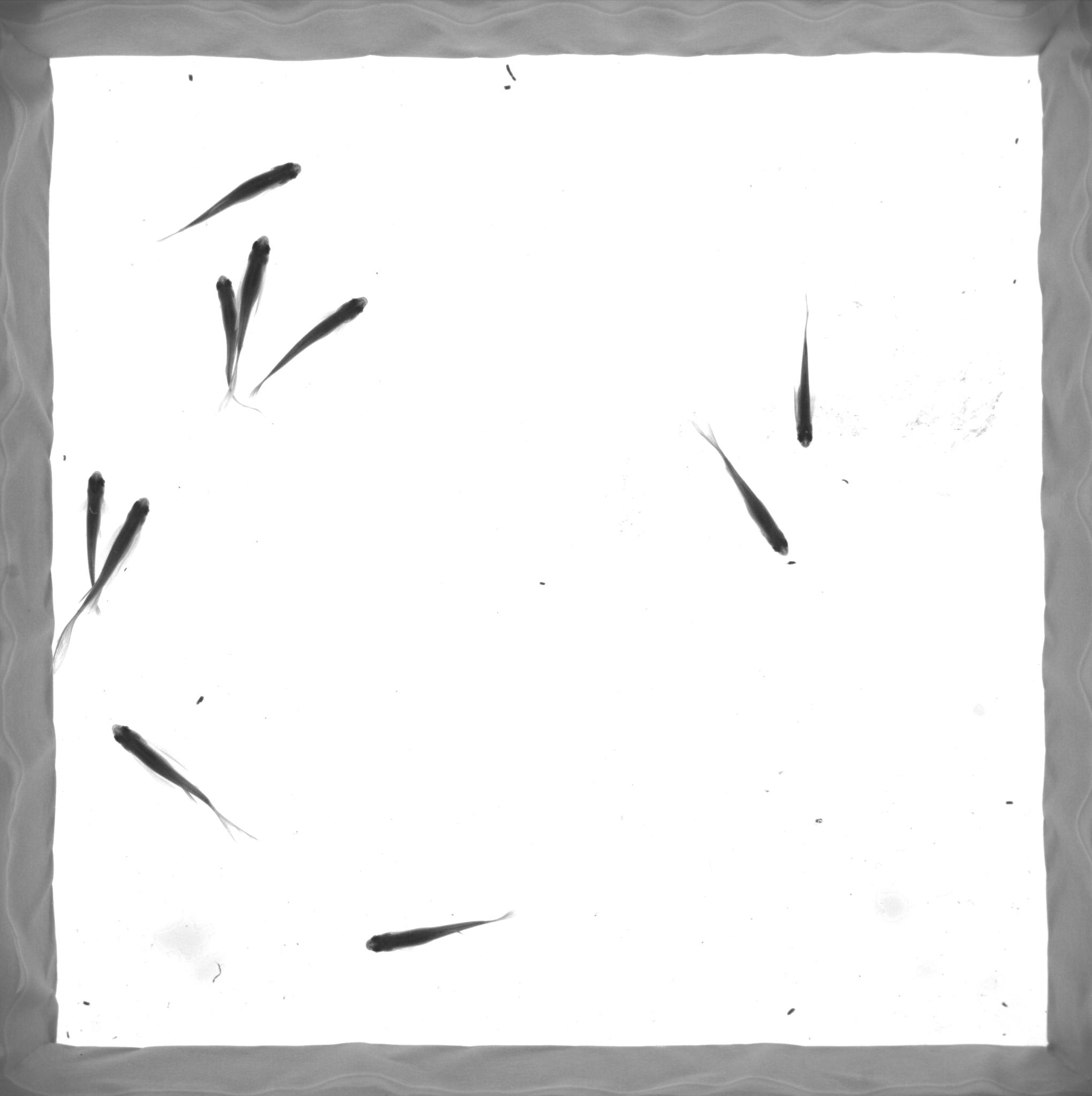

Supplement: S1 File — Source code of the proposed tracking system. (ZIP) [file pone.0154714.s002.zip › code_final/images/CoreView_275_Master_Camera_00154.jpg]

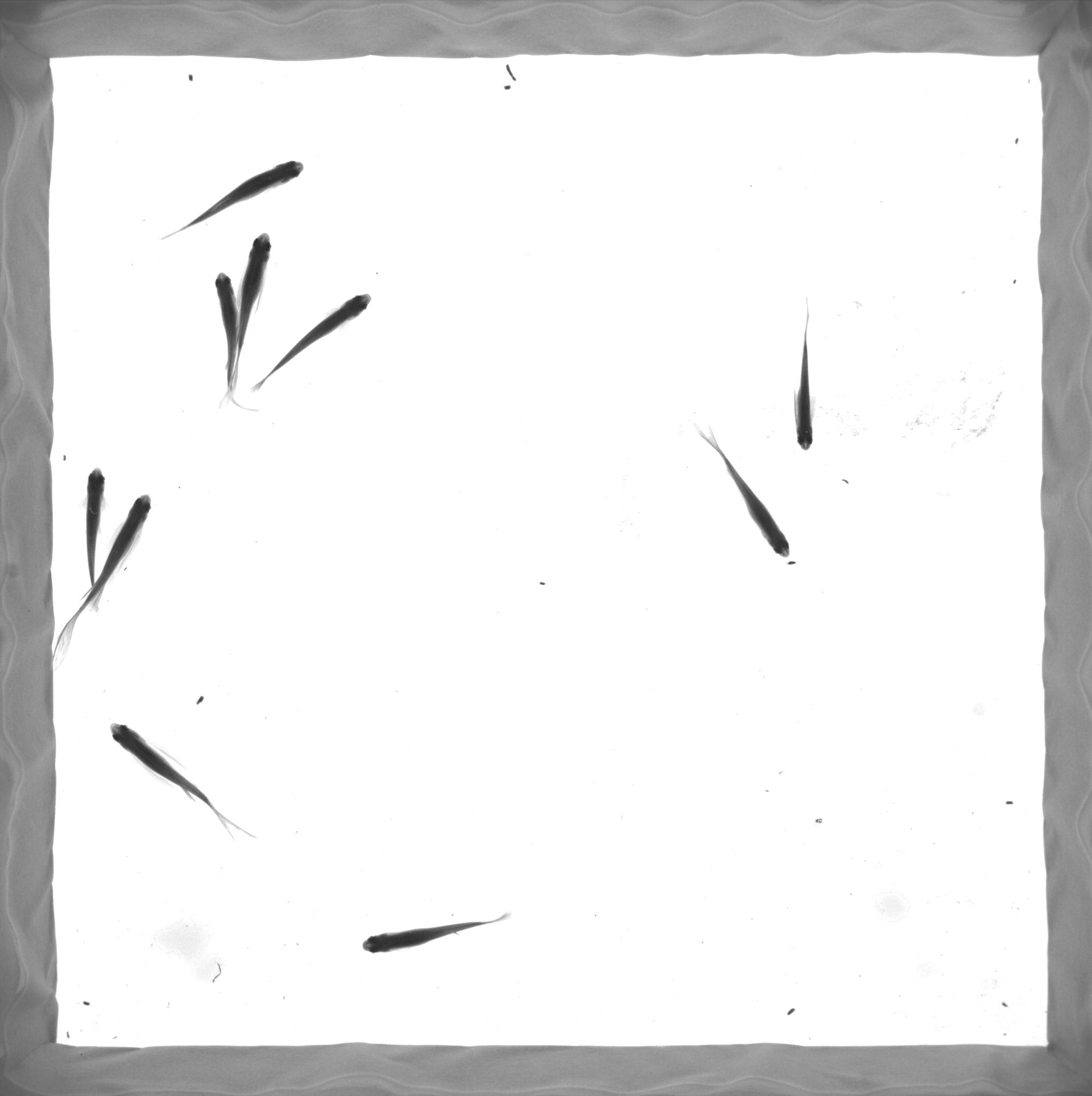

Supplement: S1 File — Source code of the proposed tracking system. (ZIP) [file pone.0154714.s002.zip › code_final/images/CoreView_275_Master_Camera_00155.jpg]

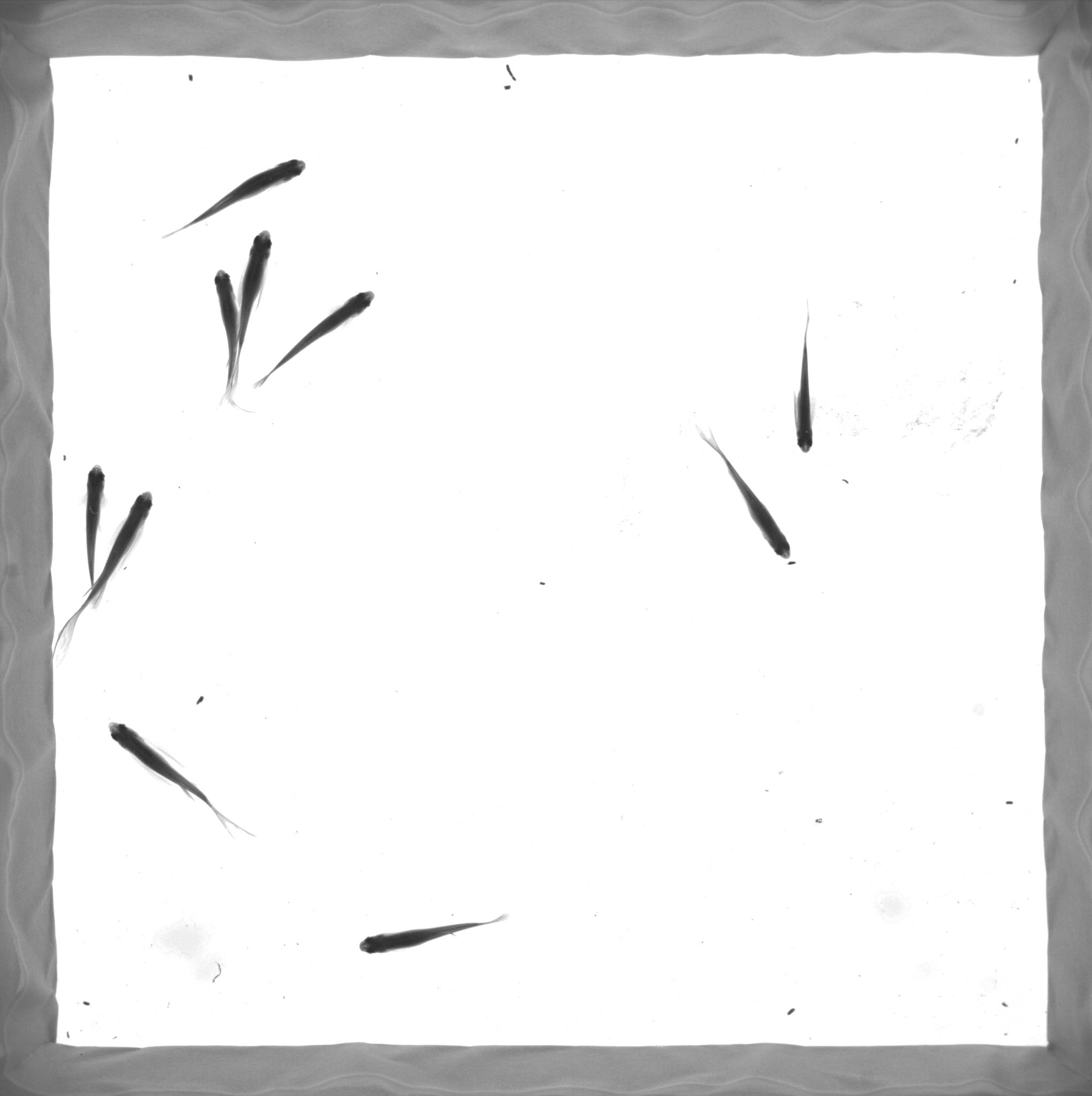

Supplement: S1 File — Source code of the proposed tracking system. (ZIP) [file pone.0154714.s002.zip › code_final/images/CoreView_275_Master_Camera_00156.jpg]

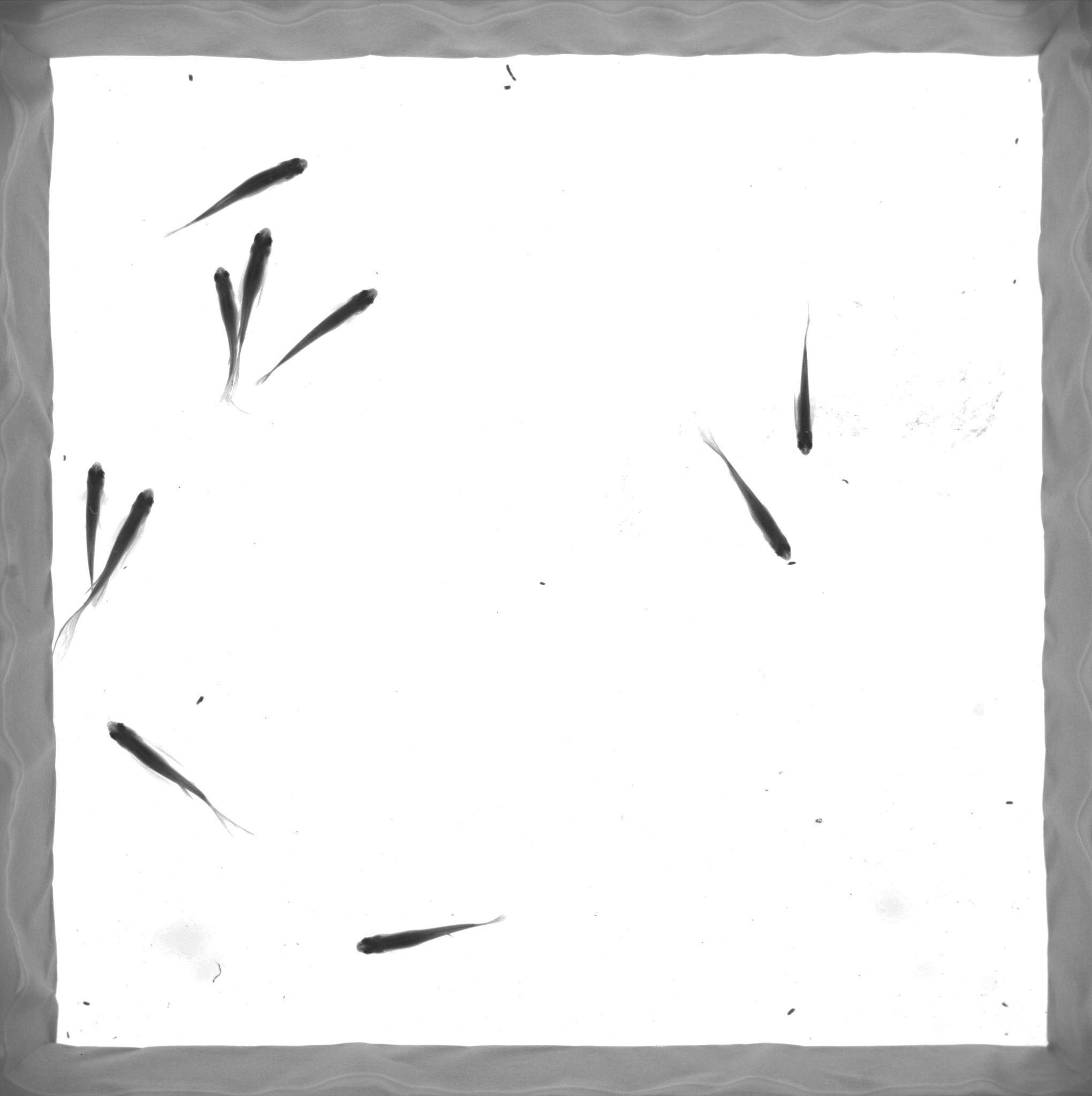

Supplement: S1 File — Source code of the proposed tracking system. (ZIP) [file pone.0154714.s002.zip › code_final/images/CoreView_275_Master_Camera_00157.jpg]

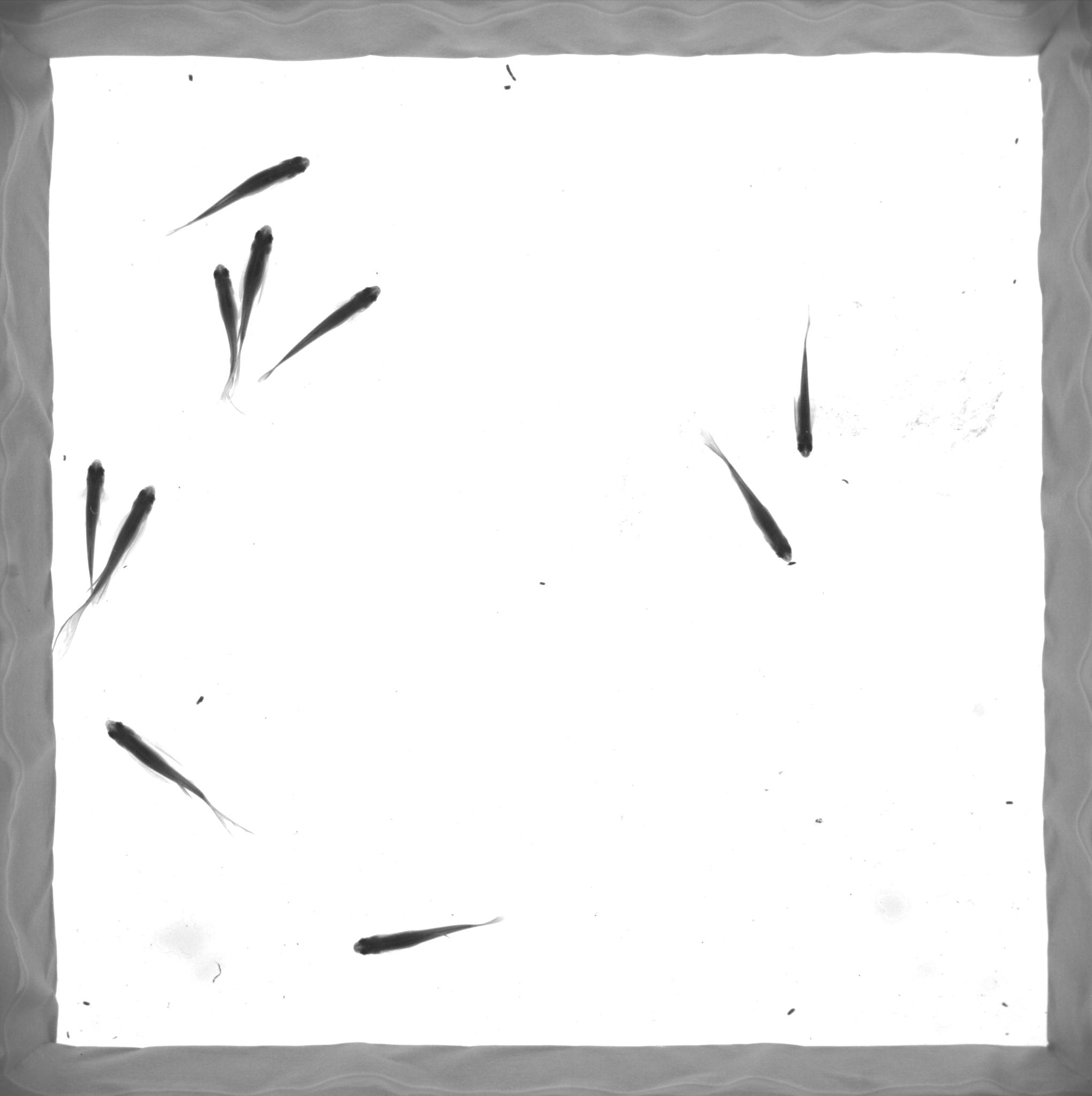

Supplement: S1 File — Source code of the proposed tracking system. (ZIP) [file pone.0154714.s002.zip › code_final/images/CoreView_275_Master_Camera_00158.jpg]

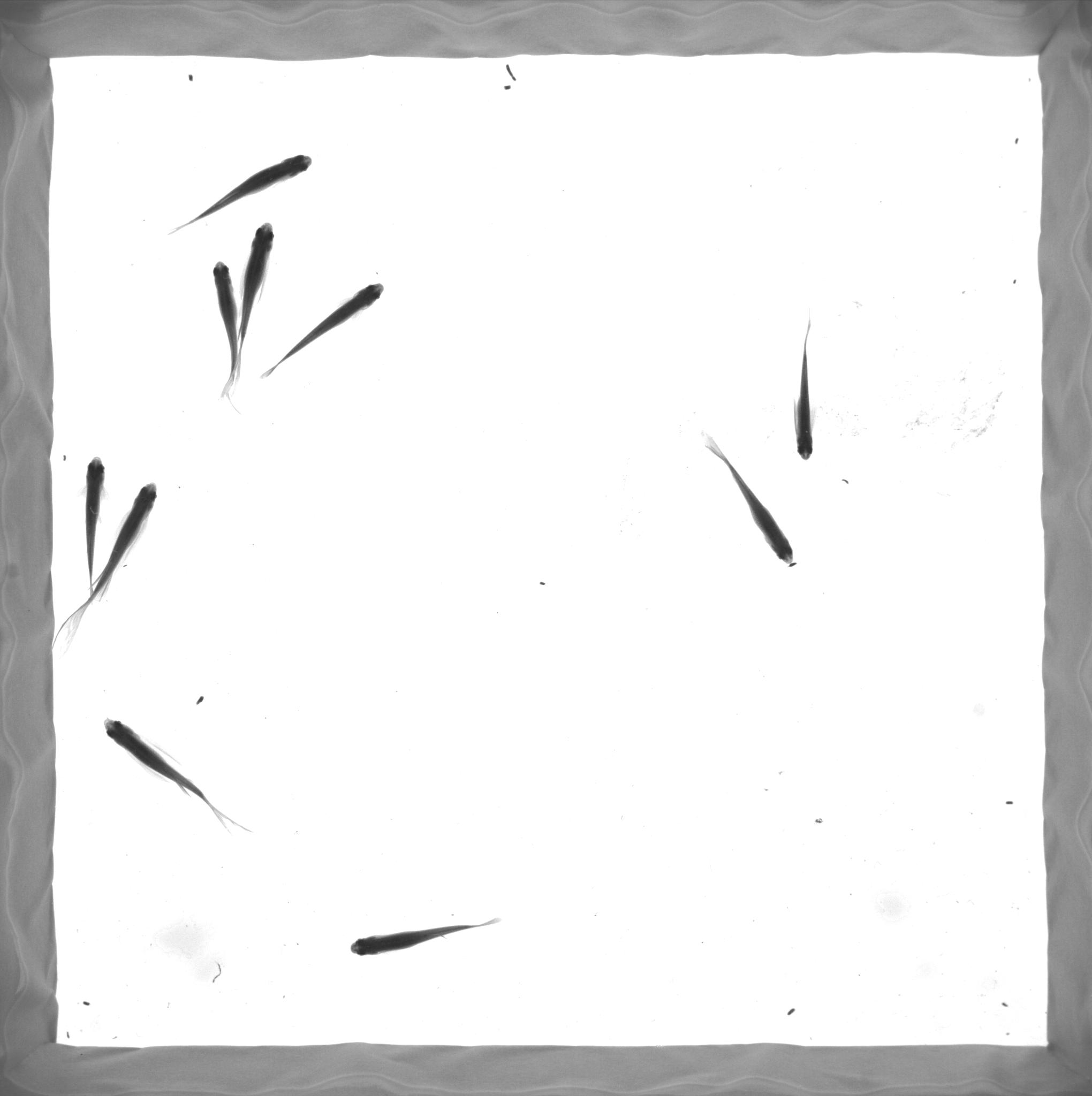

Supplement: S1 File — Source code of the proposed tracking system. (ZIP) [file pone.0154714.s002.zip › code_final/images/CoreView_275_Master_Camera_00159.jpg]

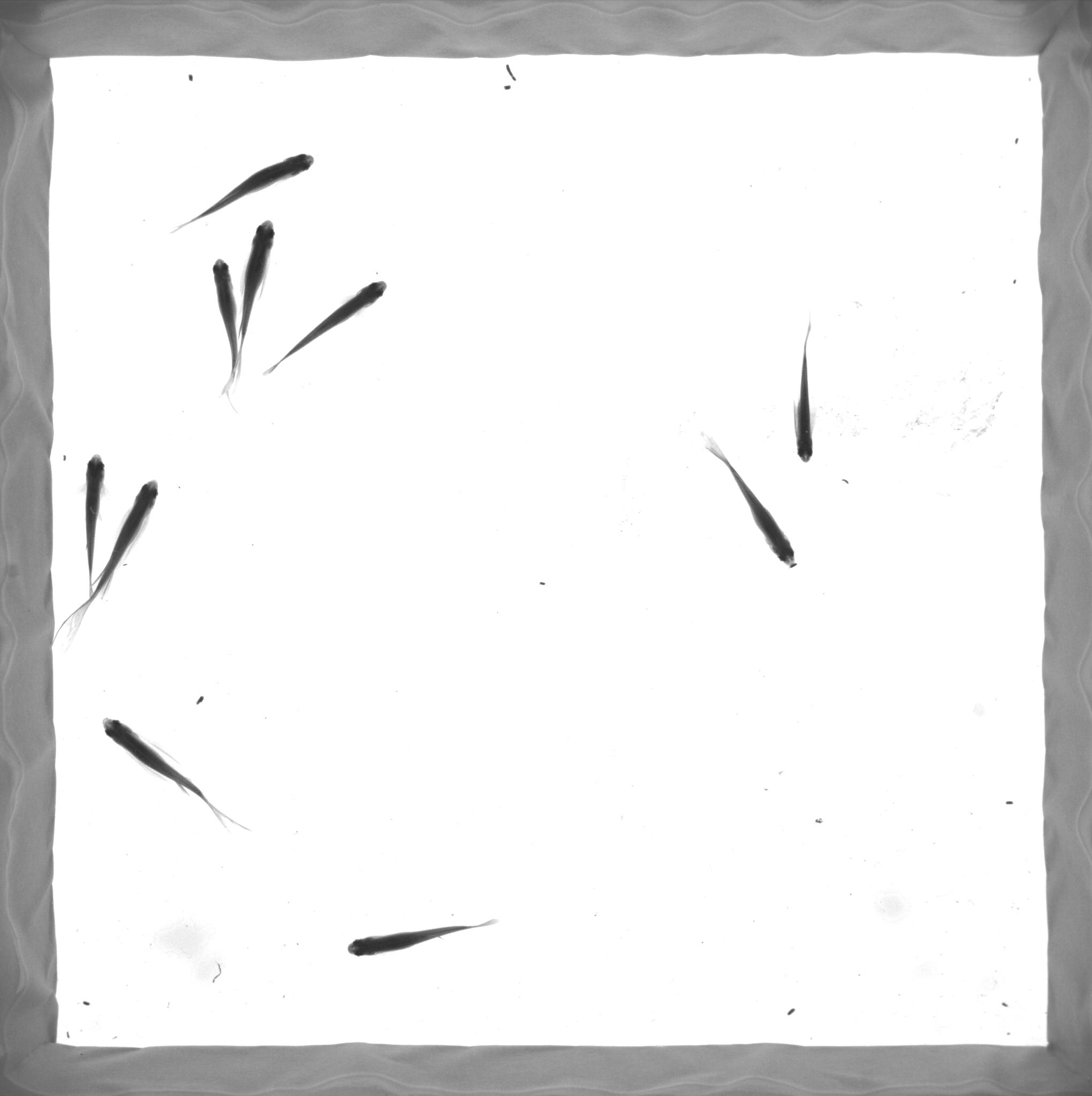

Supplement: S1 File — Source code of the proposed tracking system. (ZIP) [file pone.0154714.s002.zip › code_final/images/CoreView_275_Master_Camera_00160.jpg]

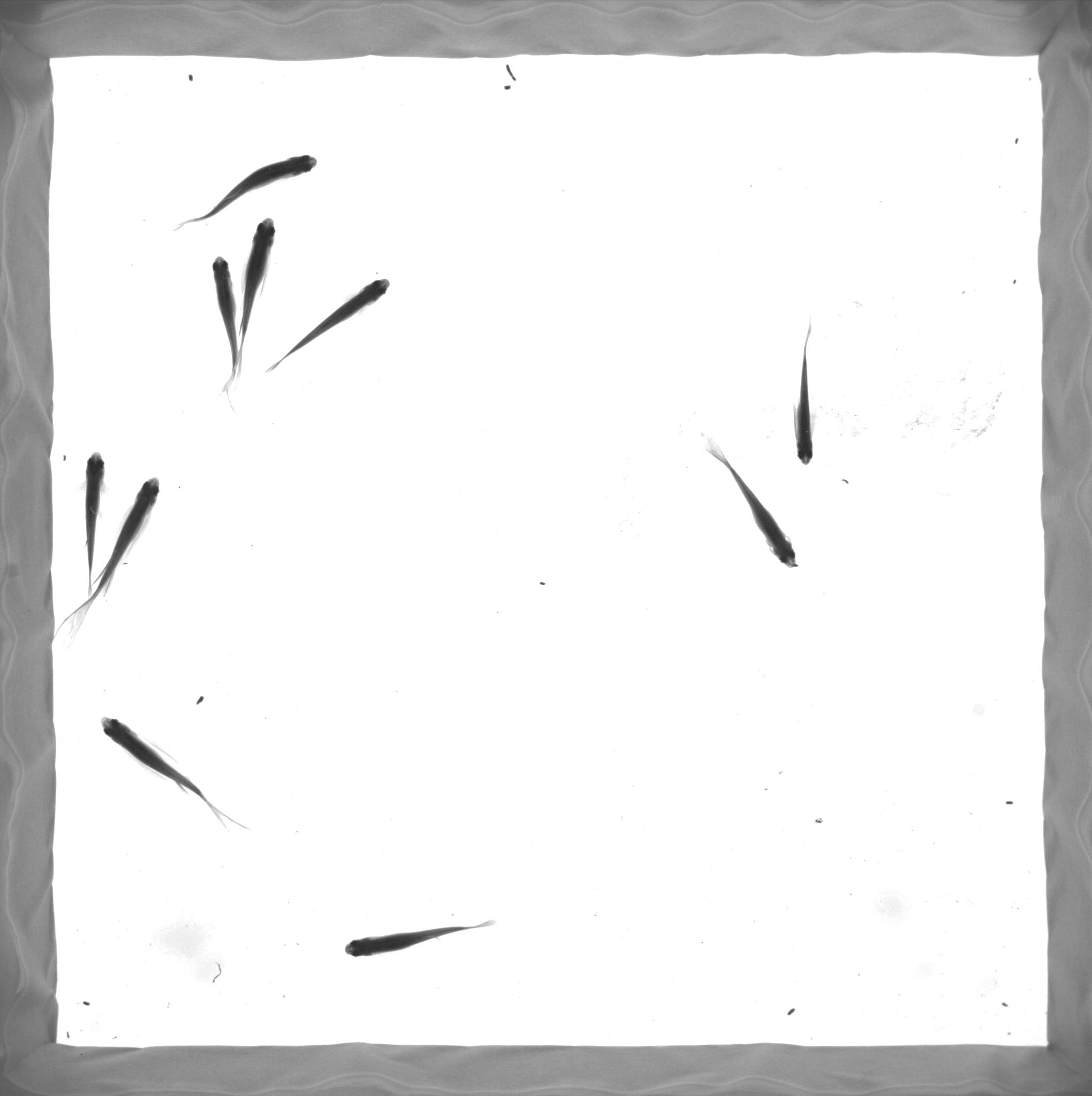

Supplement: S1 File — Source code of the proposed tracking system. (ZIP) [file pone.0154714.s002.zip › code_final/images/CoreView_275_Master_Camera_00161.jpg]

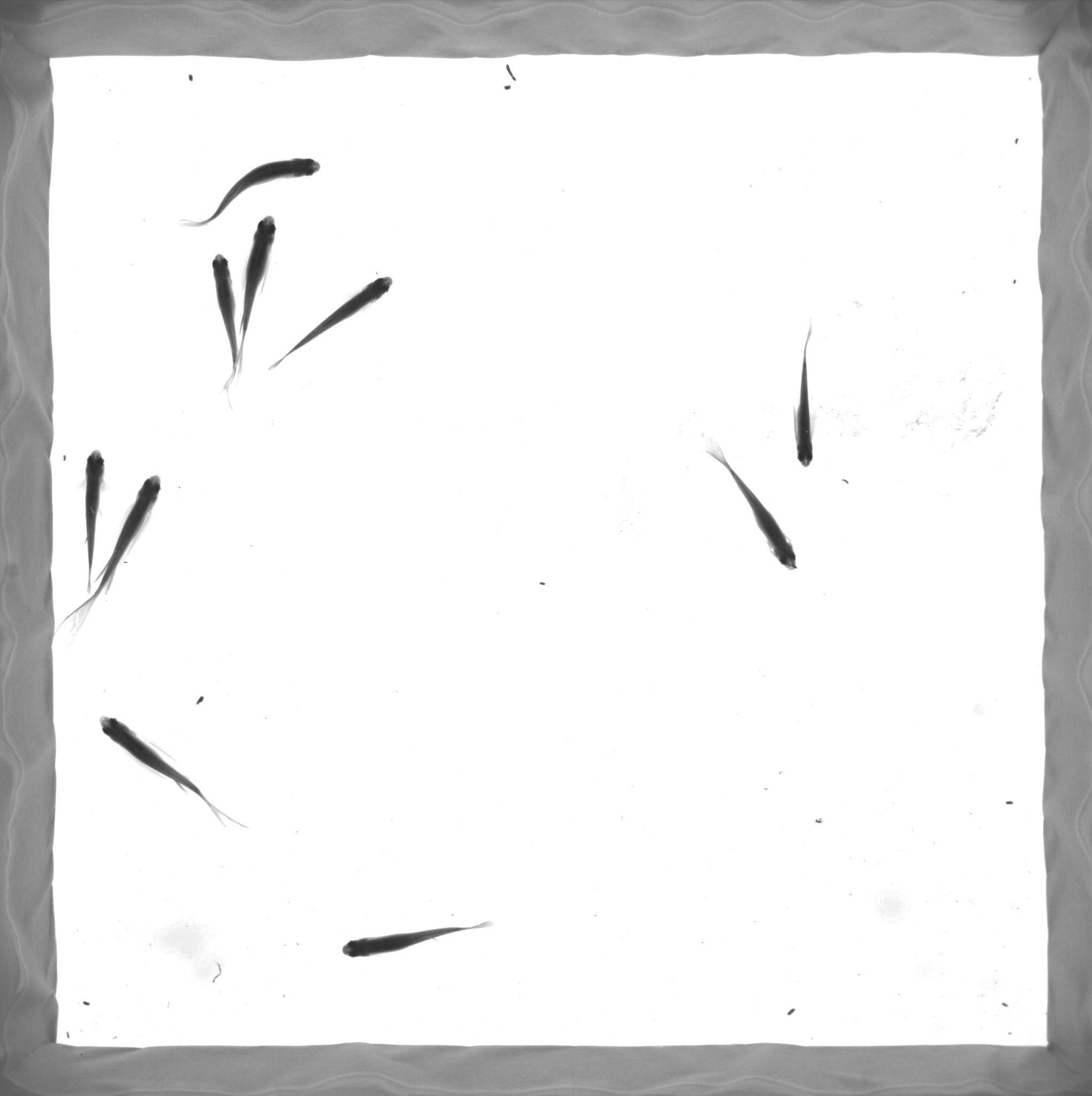

Supplement: S1 File — Source code of the proposed tracking system. (ZIP) [file pone.0154714.s002.zip › code_final/images/CoreView_275_Master_Camera_00162.jpg]

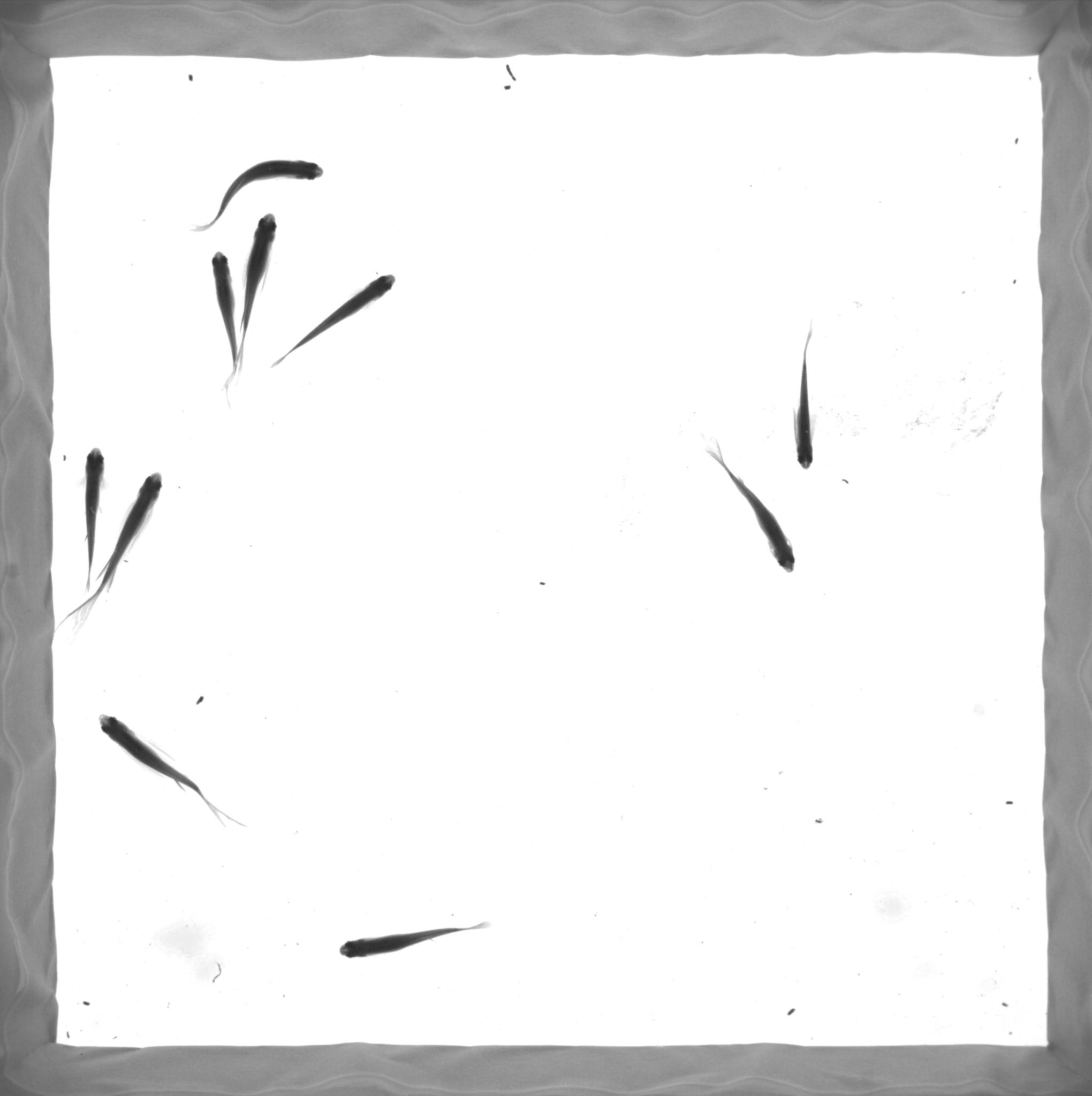

Supplement: S1 File — Source code of the proposed tracking system. (ZIP) [file pone.0154714.s002.zip › code_final/images/CoreView_275_Master_Camera_00163.jpg]

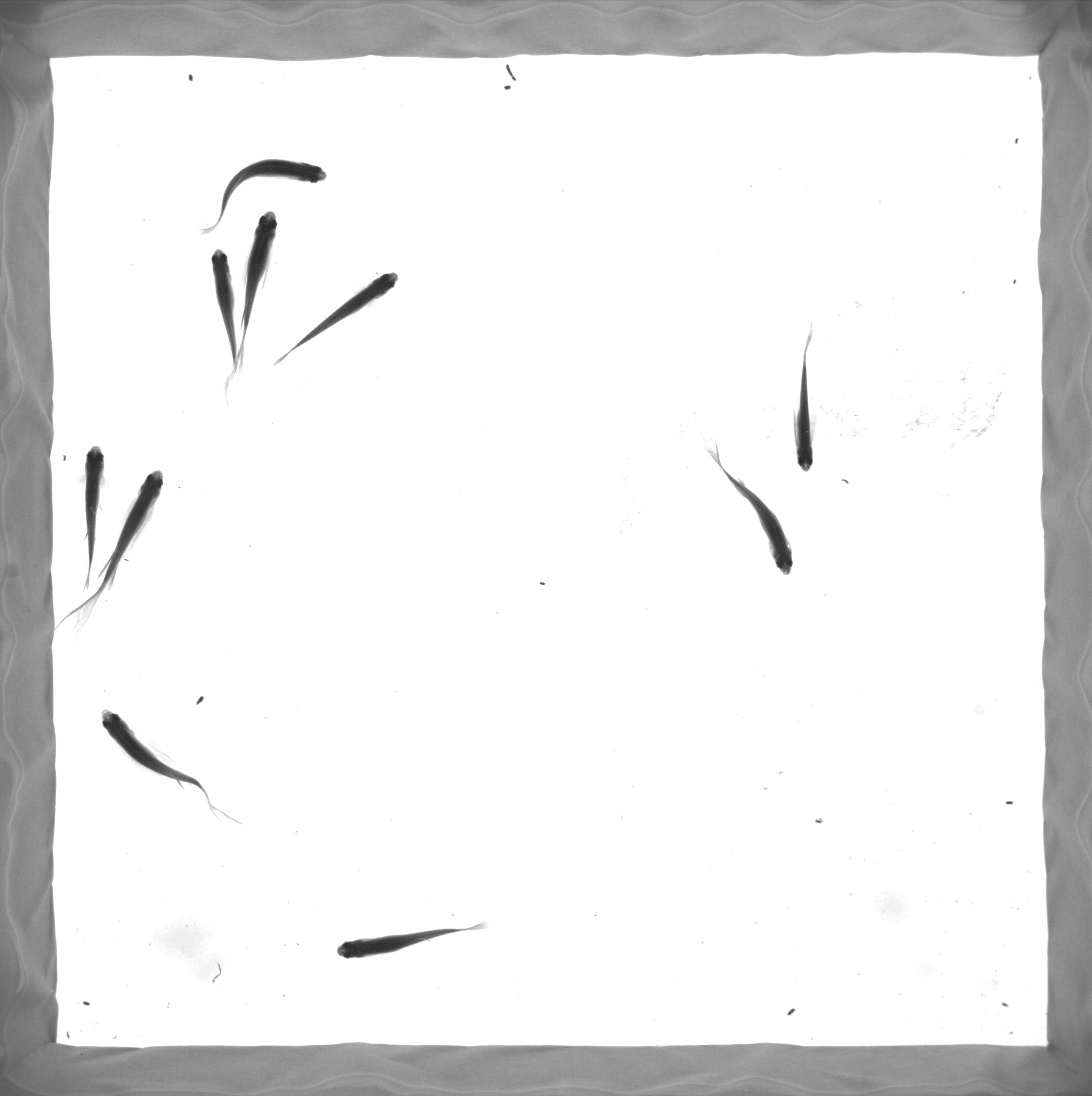

Supplement: S1 File — Source code of the proposed tracking system. (ZIP) [file pone.0154714.s002.zip › code_final/images/CoreView_275_Master_Camera_00164.jpg]

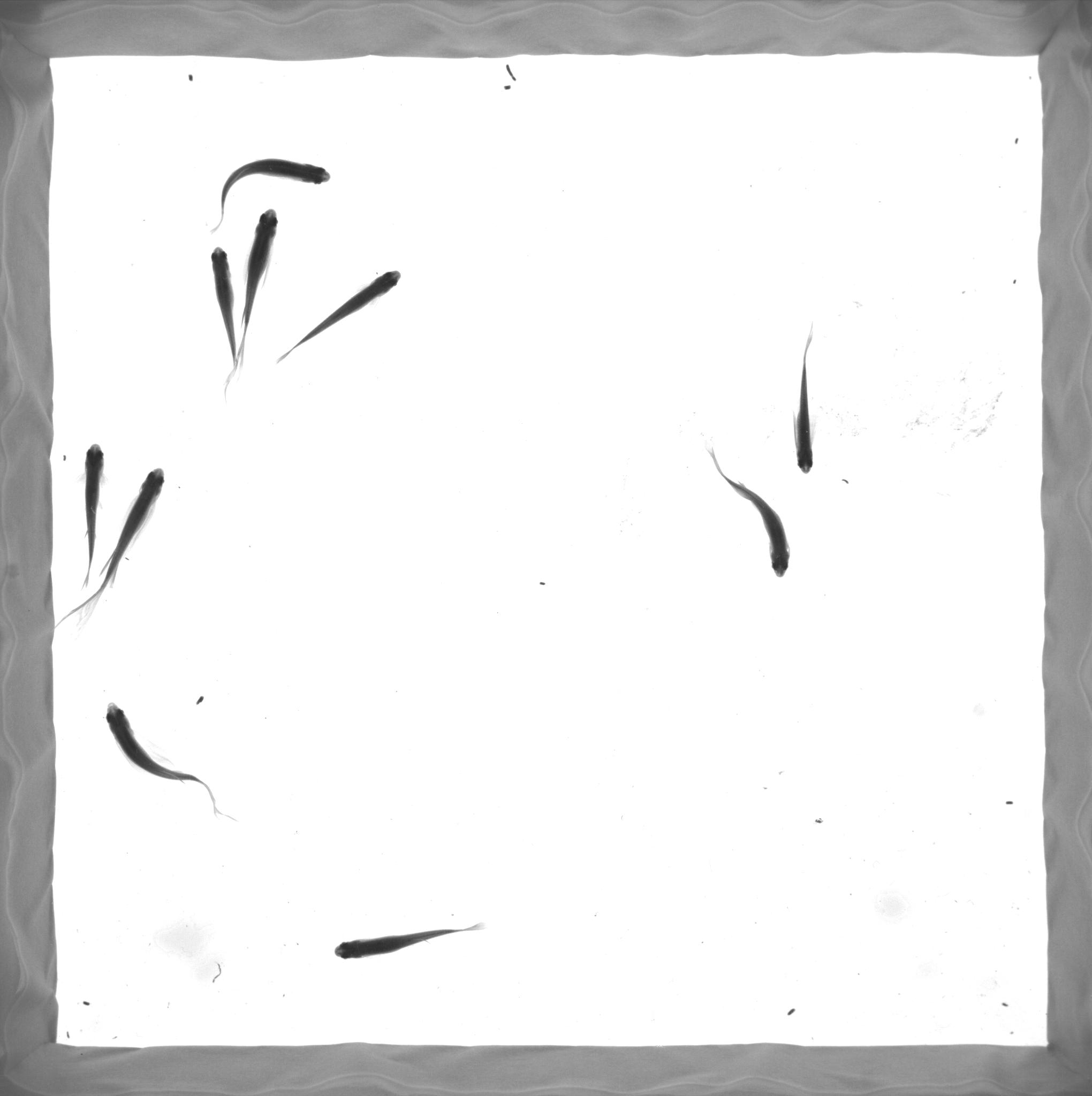

Supplement: S1 File — Source code of the proposed tracking system. (ZIP) [file pone.0154714.s002.zip › code_final/images/CoreView_275_Master_Camera_00165.jpg]

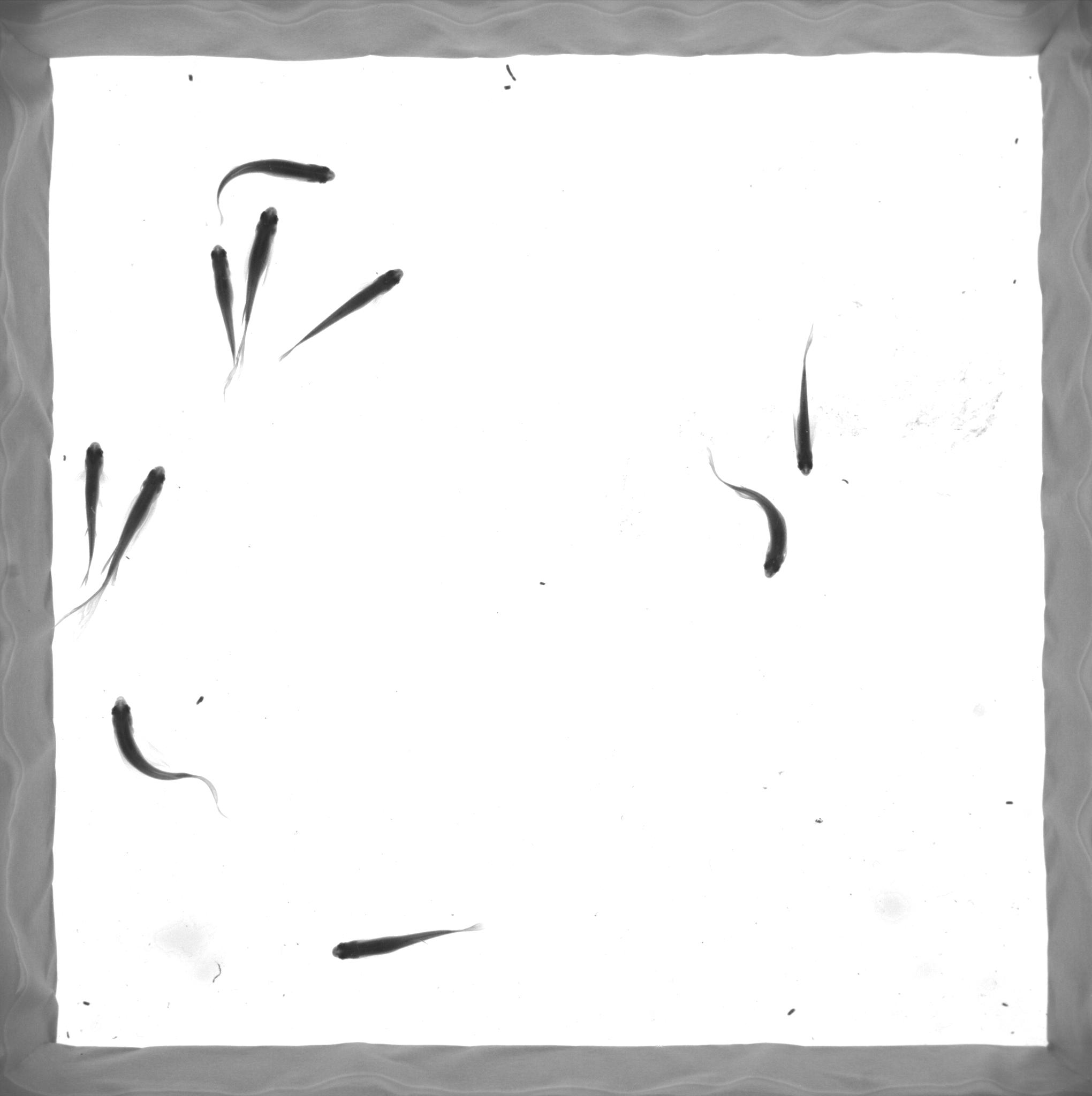

Supplement: S1 File — Source code of the proposed tracking system. (ZIP) [file pone.0154714.s002.zip › code_final/images/CoreView_275_Master_Camera_00166.jpg]

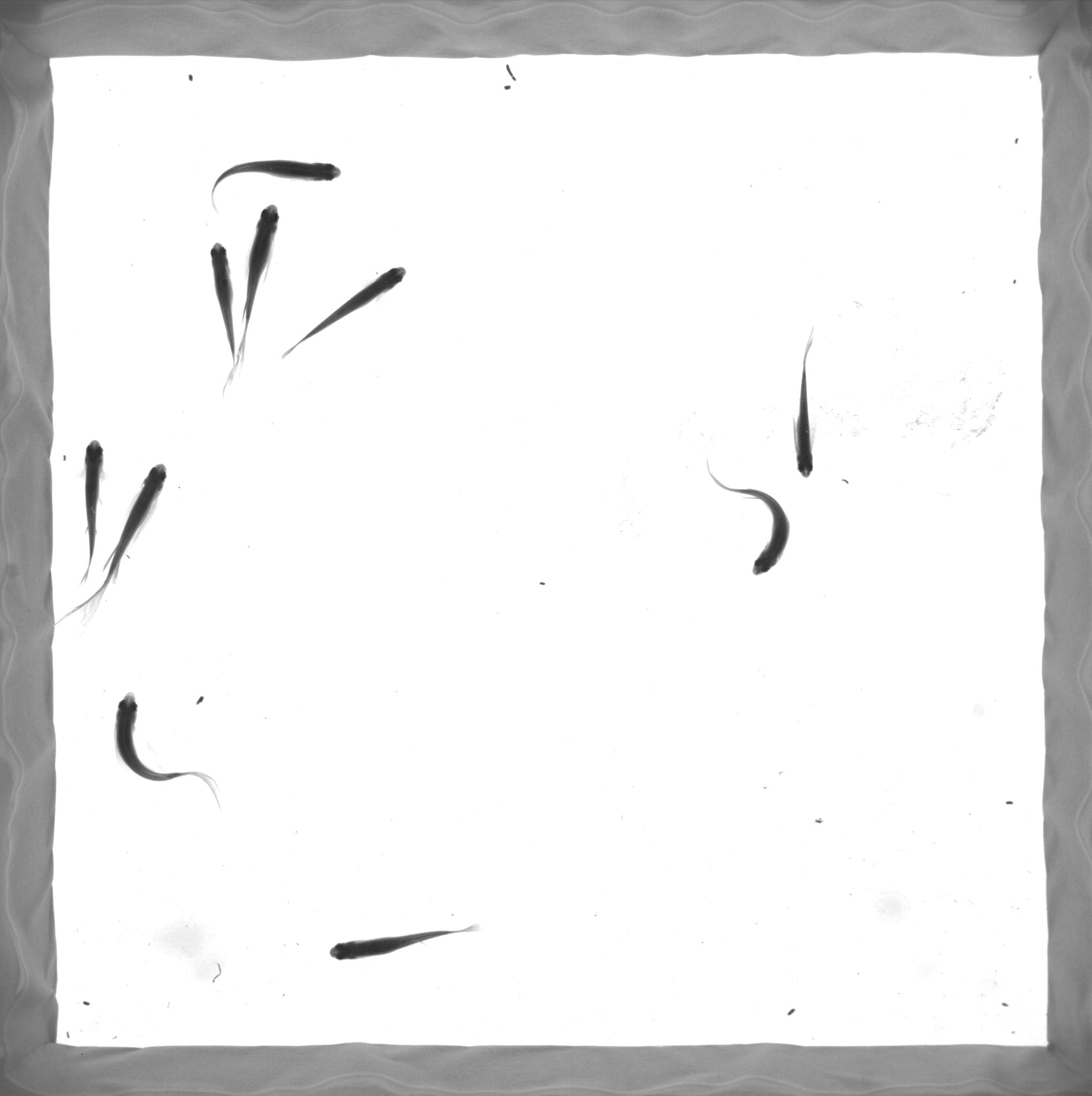

Supplement: S1 File — Source code of the proposed tracking system. (ZIP) [file pone.0154714.s002.zip › code_final/images/CoreView_275_Master_Camera_00167.jpg]

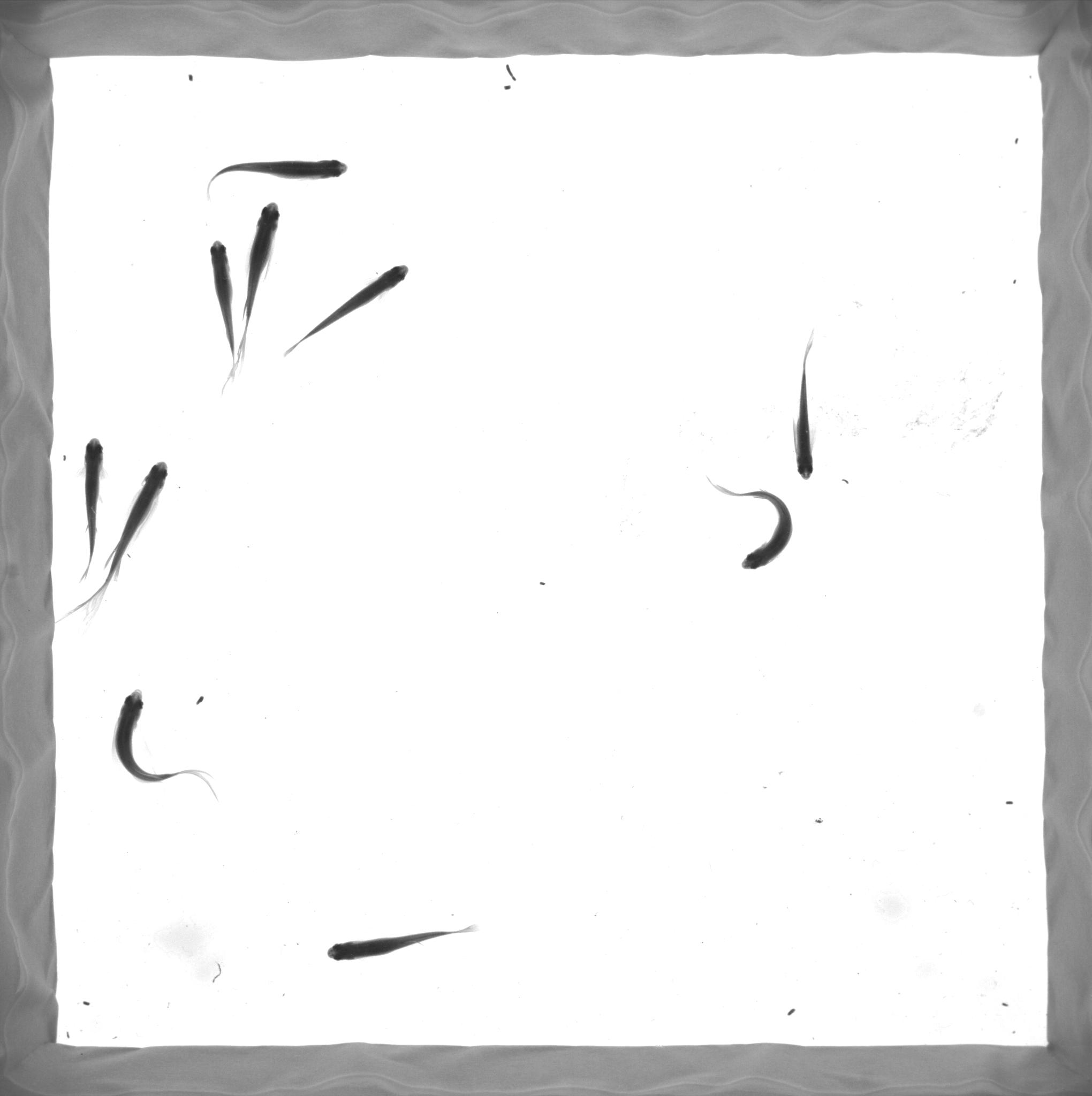

Supplement: S1 File — Source code of the proposed tracking system. (ZIP) [file pone.0154714.s002.zip › code_final/images/CoreView_275_Master_Camera_00168.jpg]

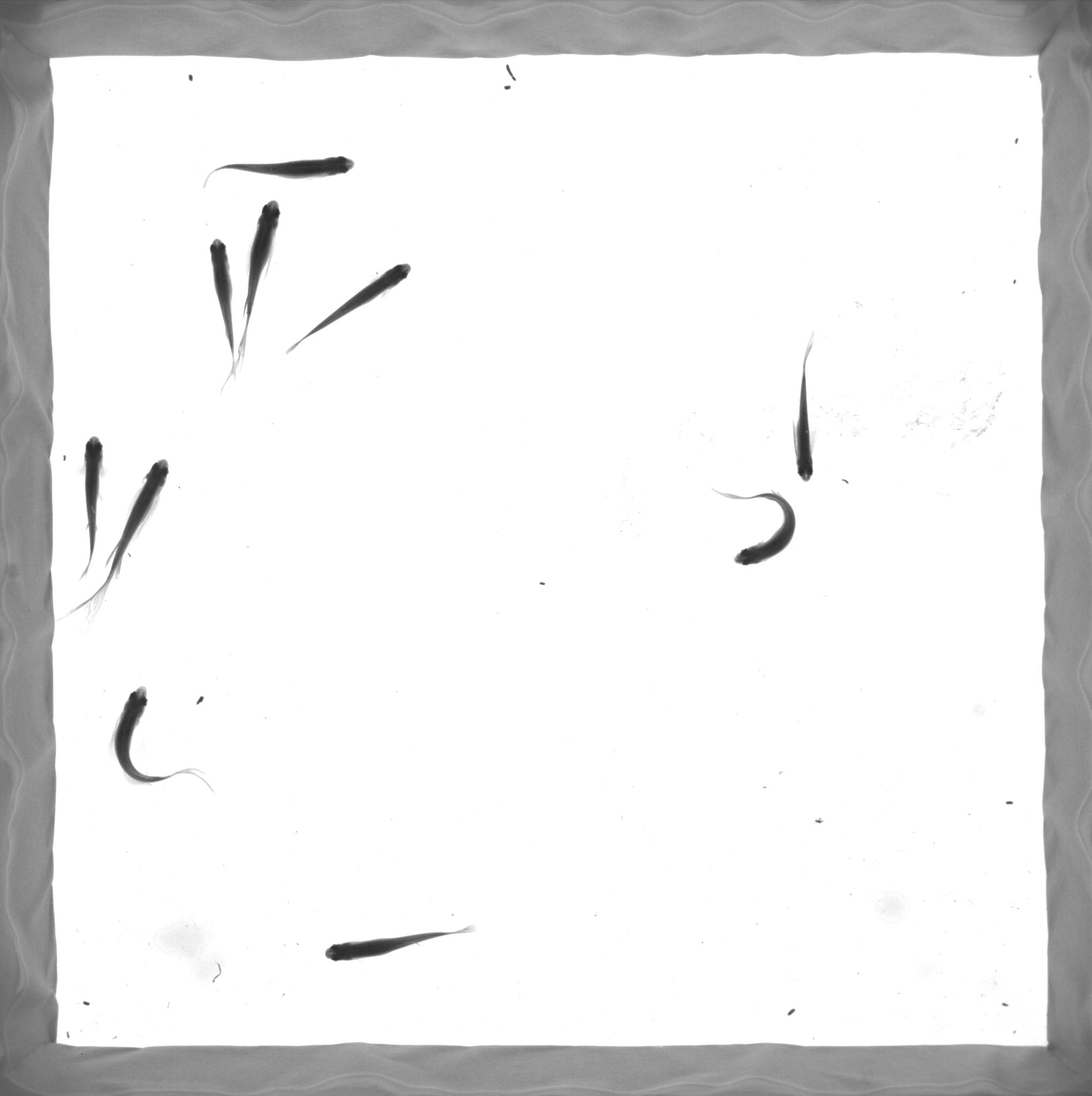

Supplement: S1 File — Source code of the proposed tracking system. (ZIP) [file pone.0154714.s002.zip › code_final/images/CoreView_275_Master_Camera_00169.jpg]

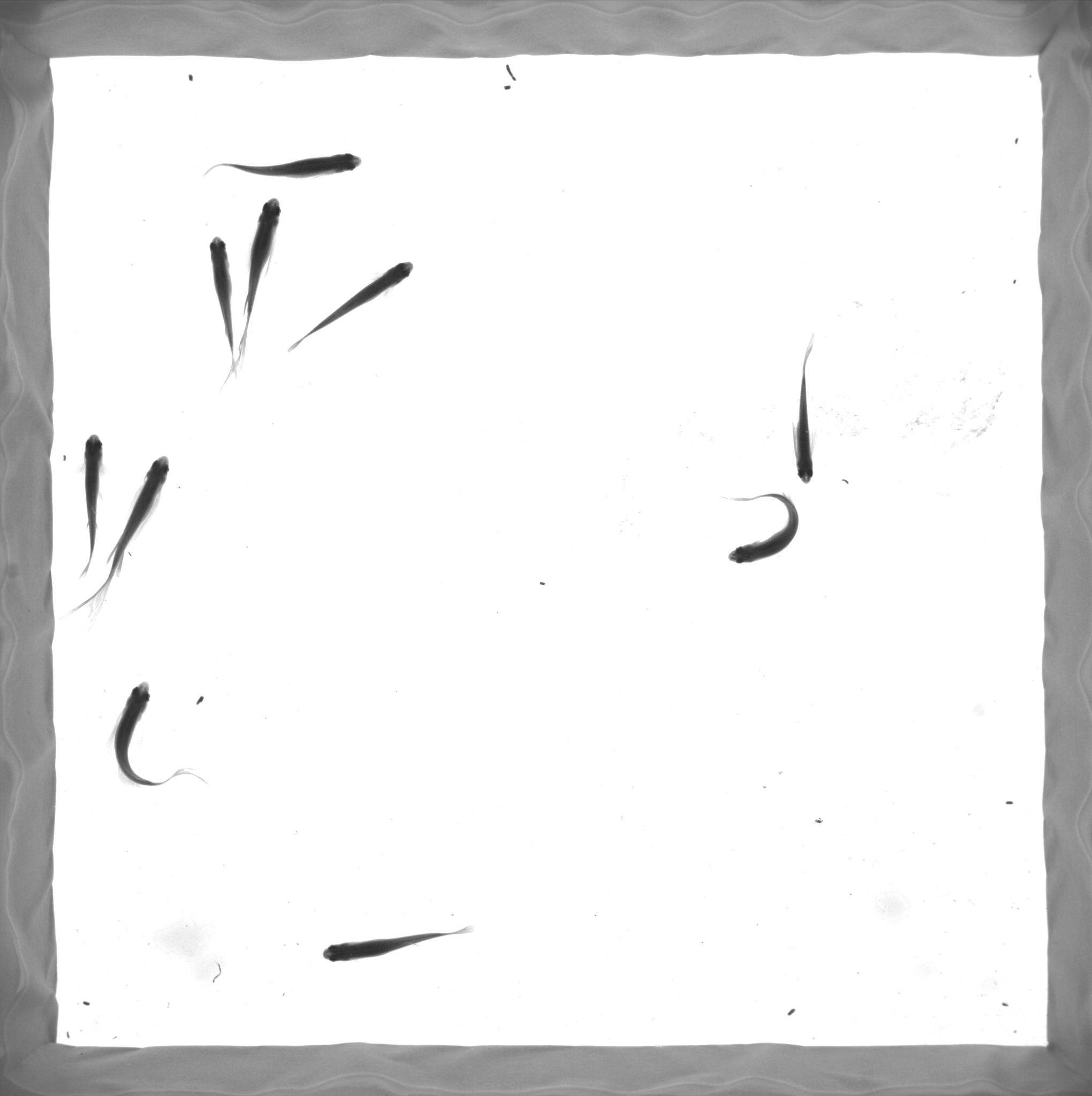

Supplement: S1 File — Source code of the proposed tracking system. (ZIP) [file pone.0154714.s002.zip › code_final/images/CoreView_275_Master_Camera_00170.jpg]

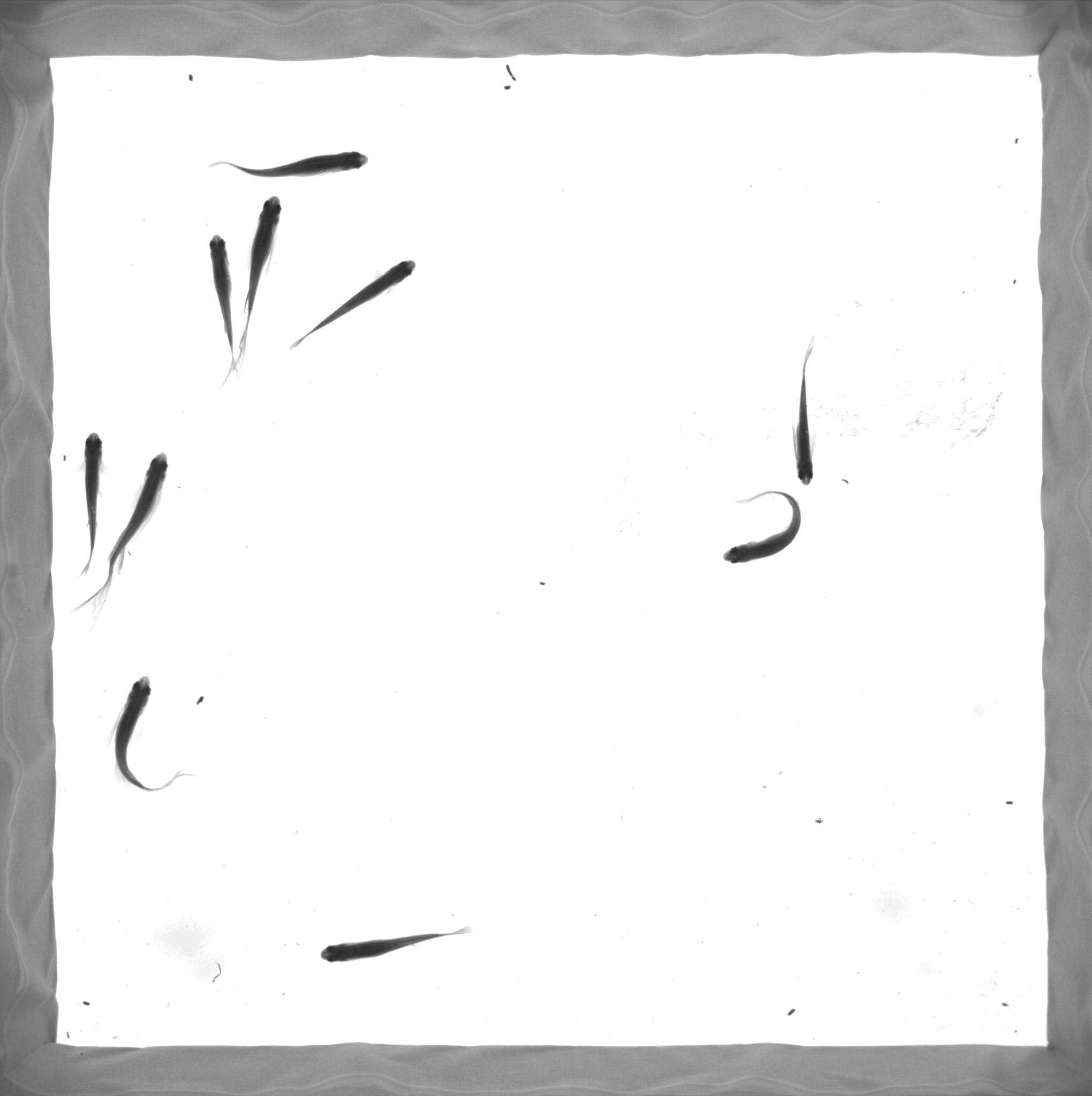

Supplement: S1 File — Source code of the proposed tracking system. (ZIP) [file pone.0154714.s002.zip › code_final/images/CoreView_275_Master_Camera_00171.jpg]

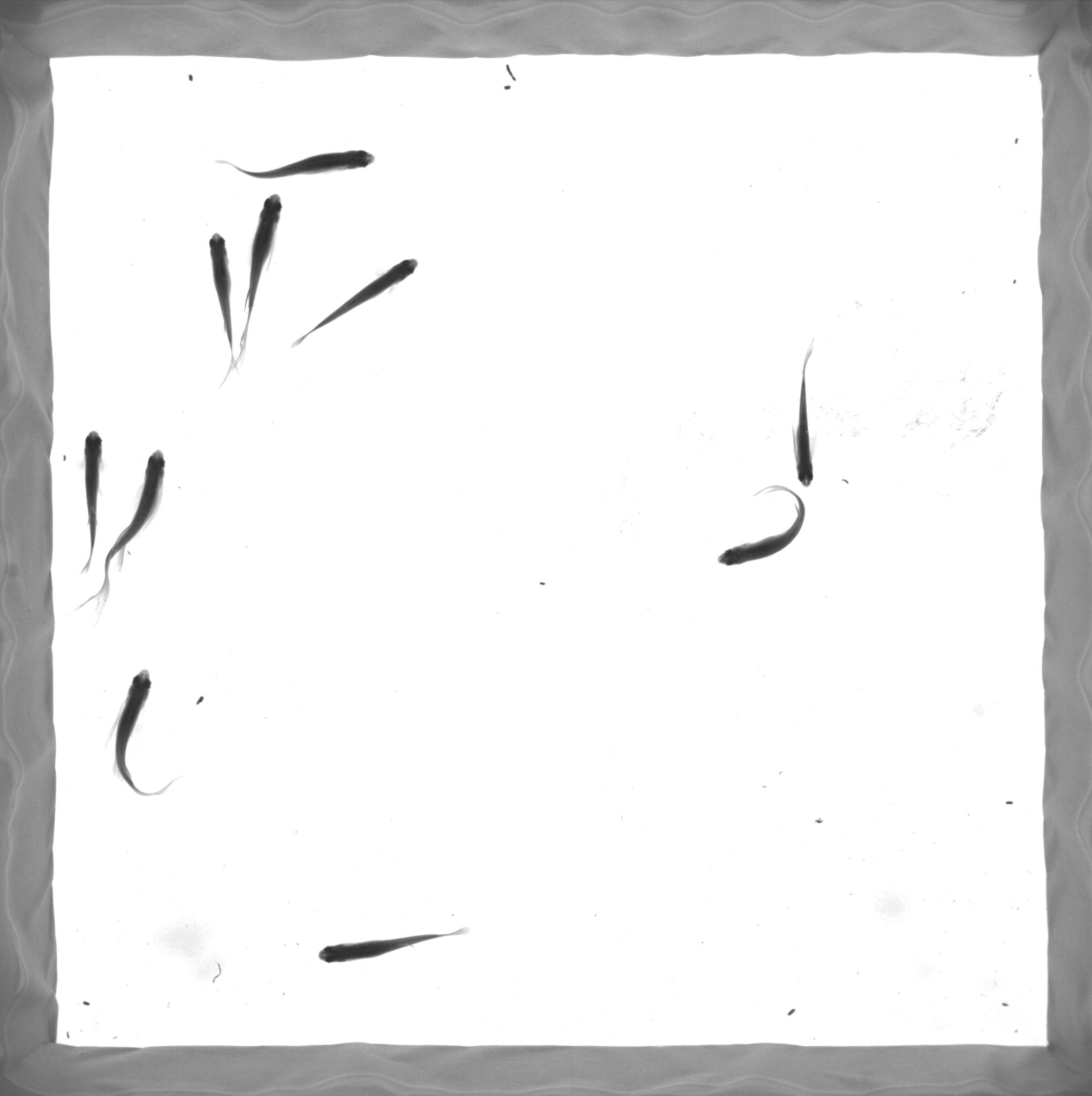

Supplement: S1 File — Source code of the proposed tracking system. (ZIP) [file pone.0154714.s002.zip › code_final/images/CoreView_275_Master_Camera_00172.jpg]

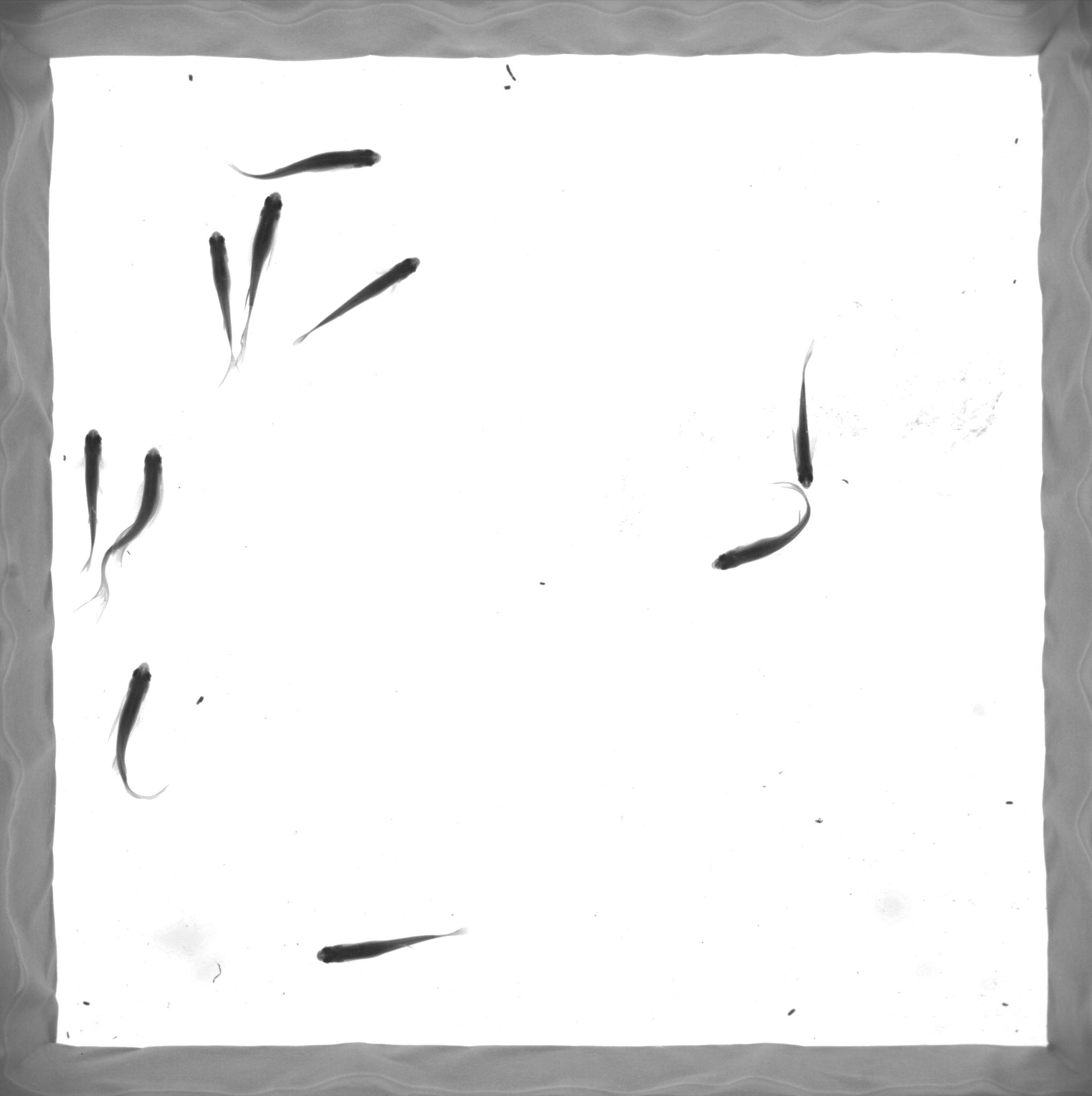

Supplement: S1 File — Source code of the proposed tracking system. (ZIP) [file pone.0154714.s002.zip › code_final/images/CoreView_275_Master_Camera_00173.jpg]

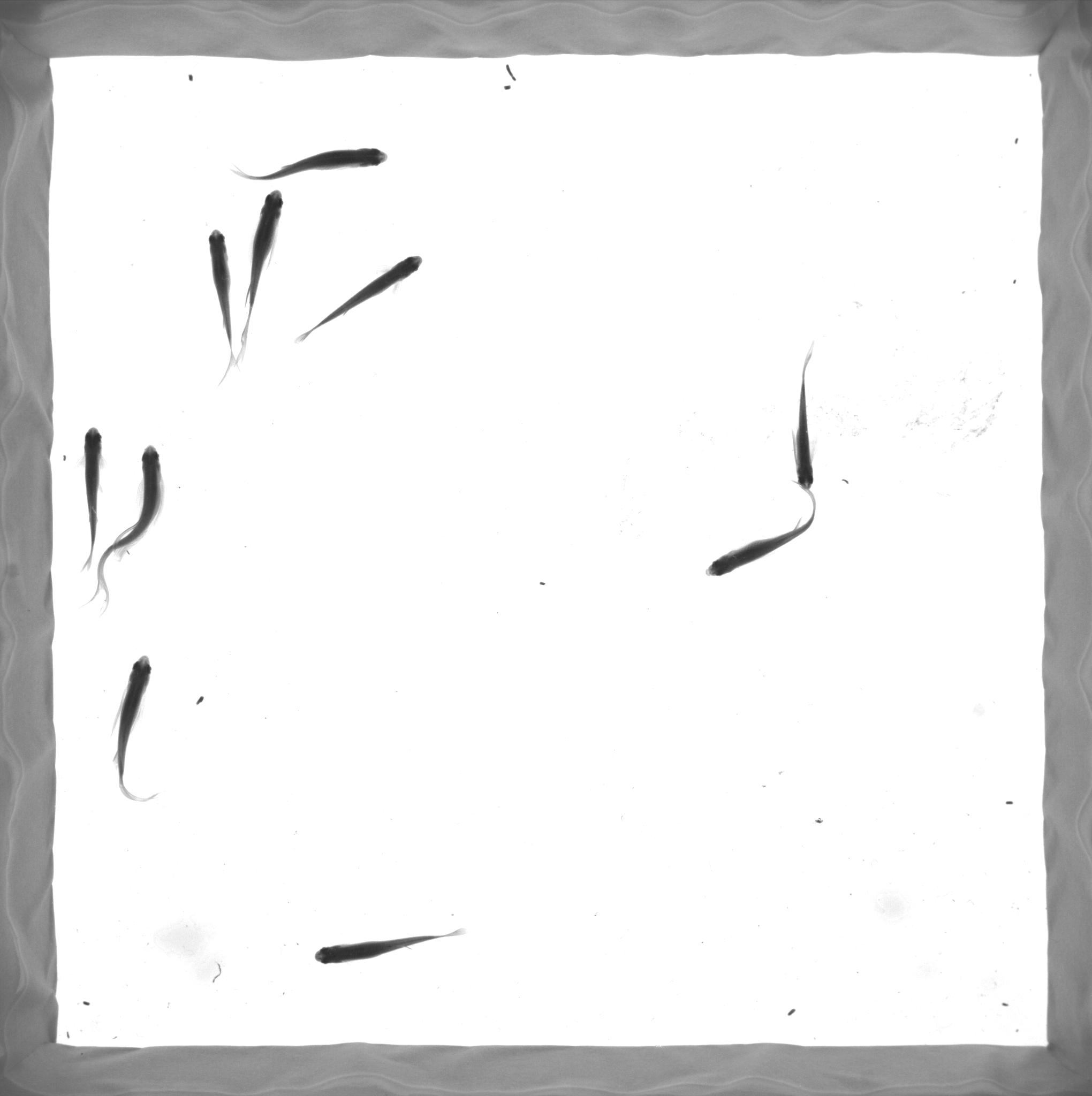

Supplement: S1 File — Source code of the proposed tracking system. (ZIP) [file pone.0154714.s002.zip › code_final/images/CoreView_275_Master_Camera_00174.jpg]

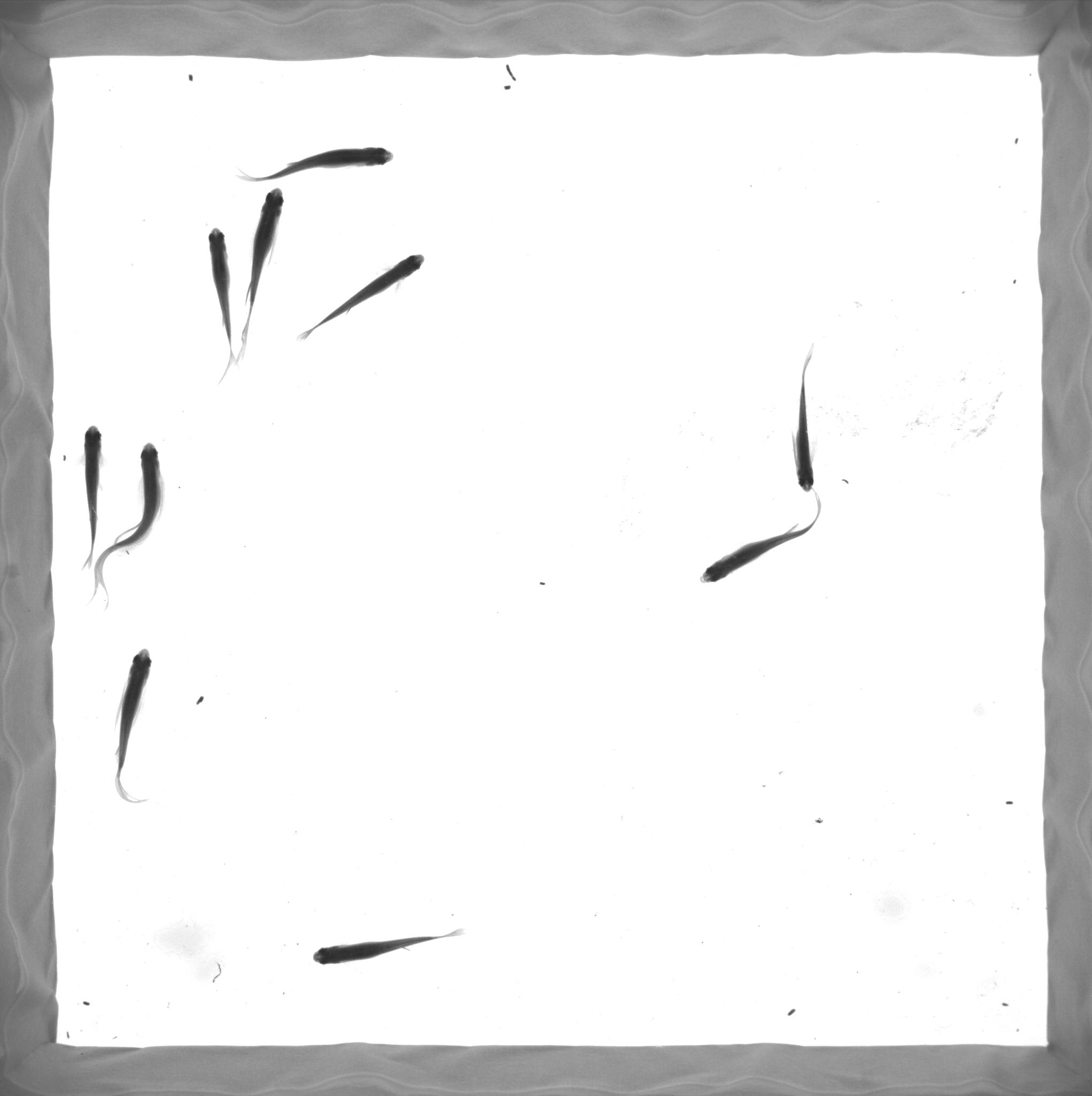

Supplement: S1 File — Source code of the proposed tracking system. (ZIP) [file pone.0154714.s002.zip › code_final/images/CoreView_275_Master_Camera_00175.jpg]

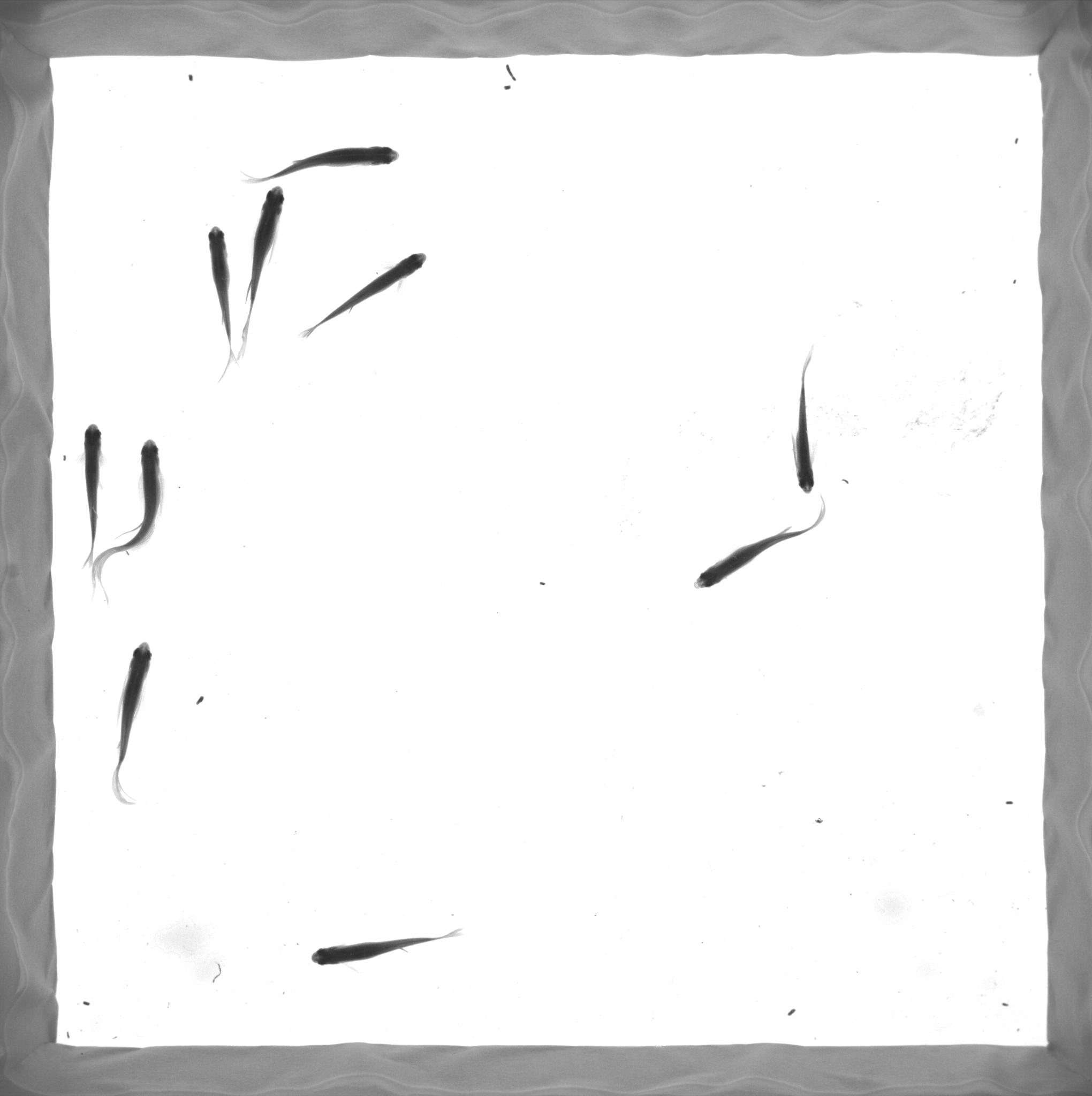

Supplement: S1 File — Source code of the proposed tracking system. (ZIP) [file pone.0154714.s002.zip › code_final/images/CoreView_275_Master_Camera_00176.jpg]

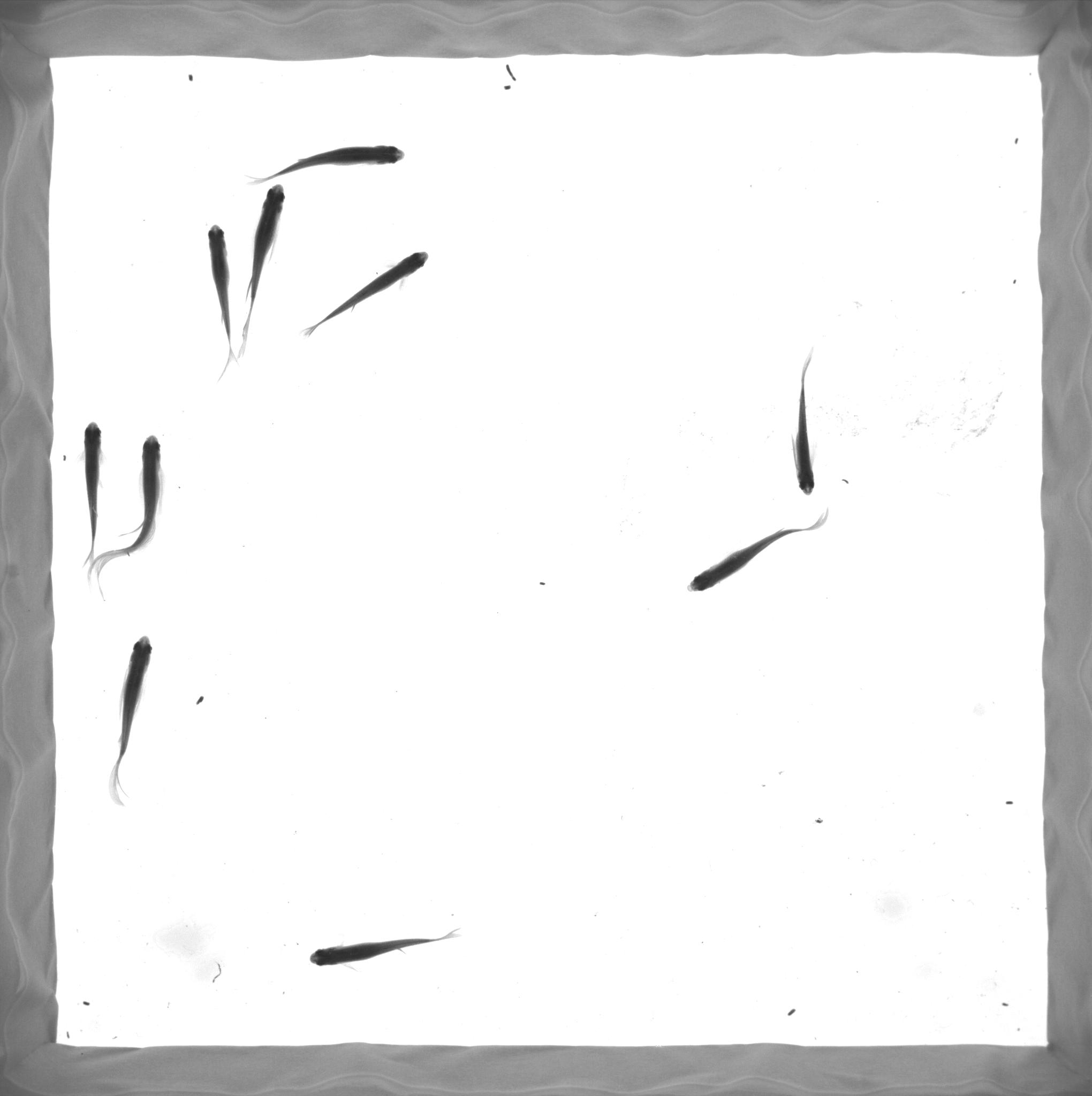

Supplement: S1 File — Source code of the proposed tracking system. (ZIP) [file pone.0154714.s002.zip › code_final/images/CoreView_275_Master_Camera_00177.jpg]

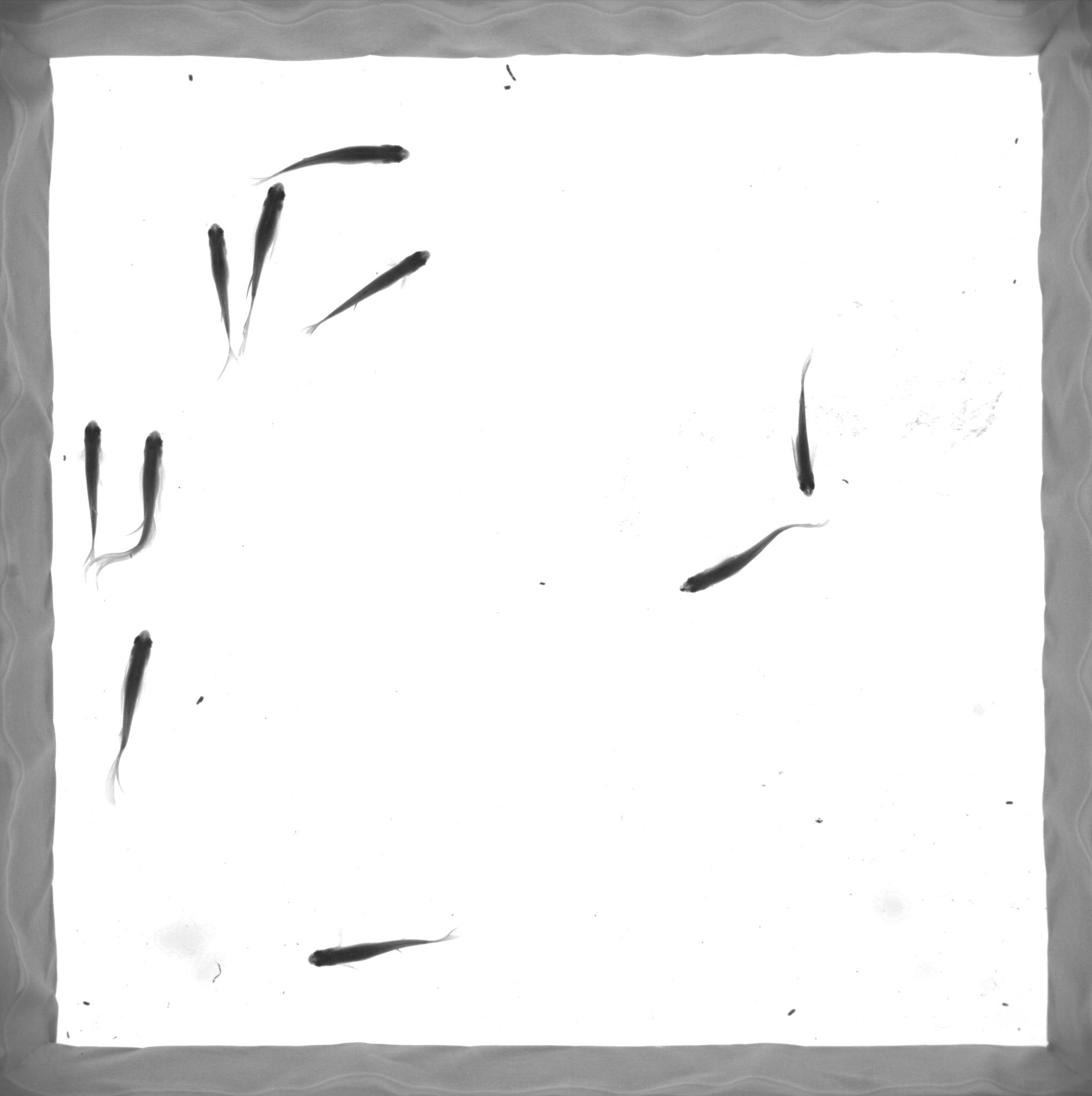

Supplement: S1 File — Source code of the proposed tracking system. (ZIP) [file pone.0154714.s002.zip › code_final/images/CoreView_275_Master_Camera_00178.jpg]

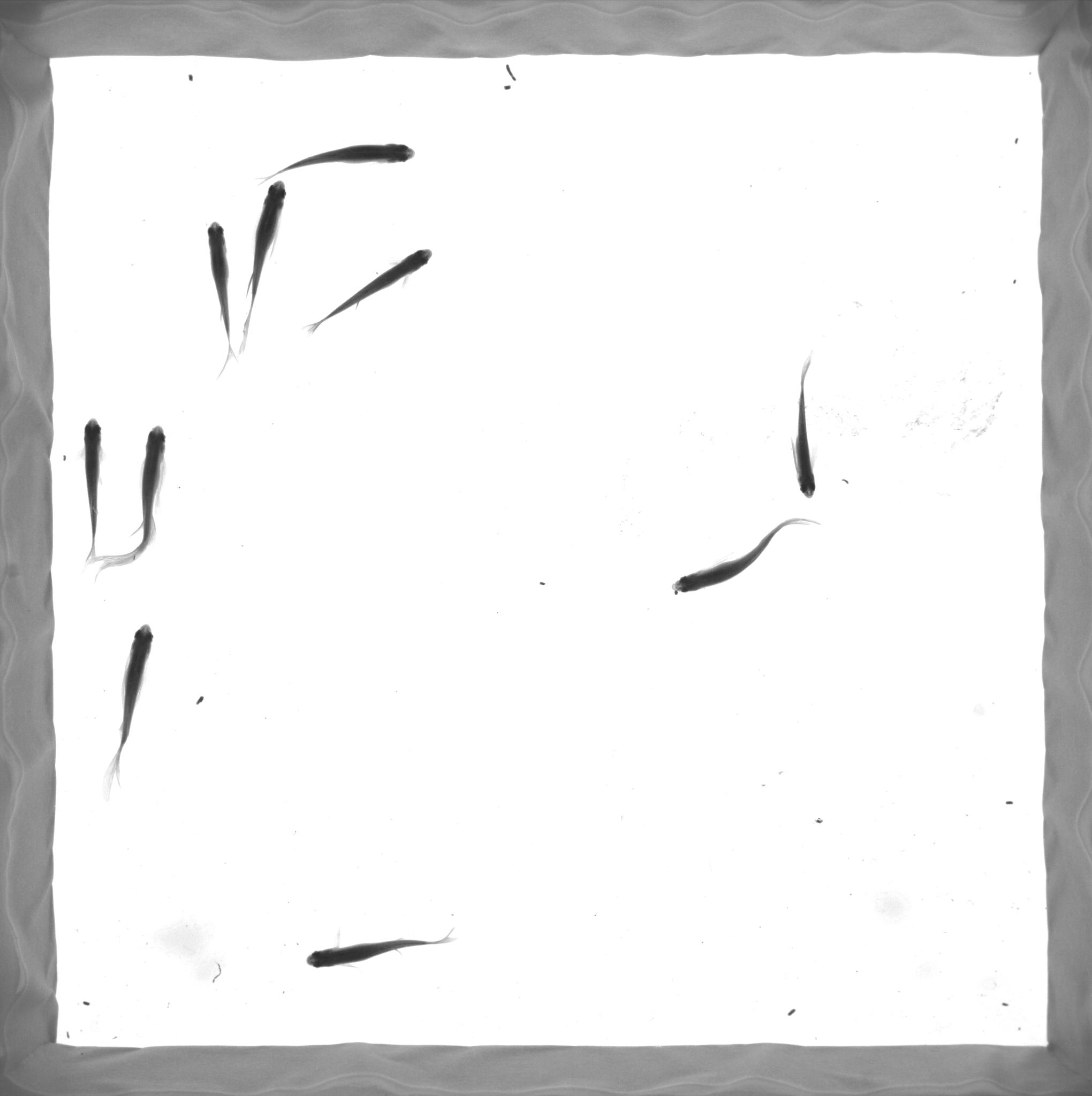

Supplement: S1 File — Source code of the proposed tracking system. (ZIP) [file pone.0154714.s002.zip › code_final/images/CoreView_275_Master_Camera_00179.jpg]

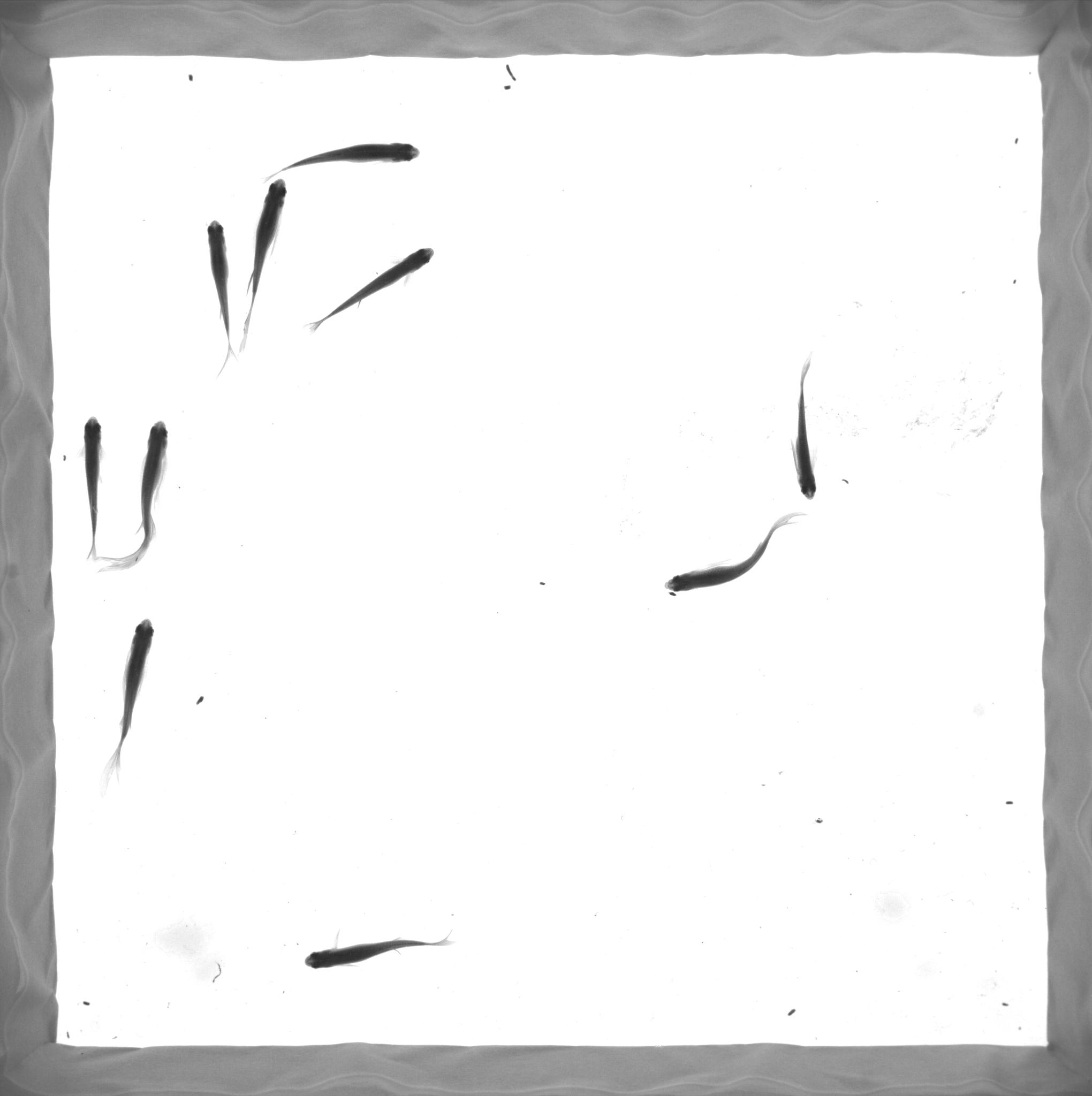

Supplement: S1 File — Source code of the proposed tracking system. (ZIP) [file pone.0154714.s002.zip › code_final/images/CoreView_275_Master_Camera_00180.jpg]

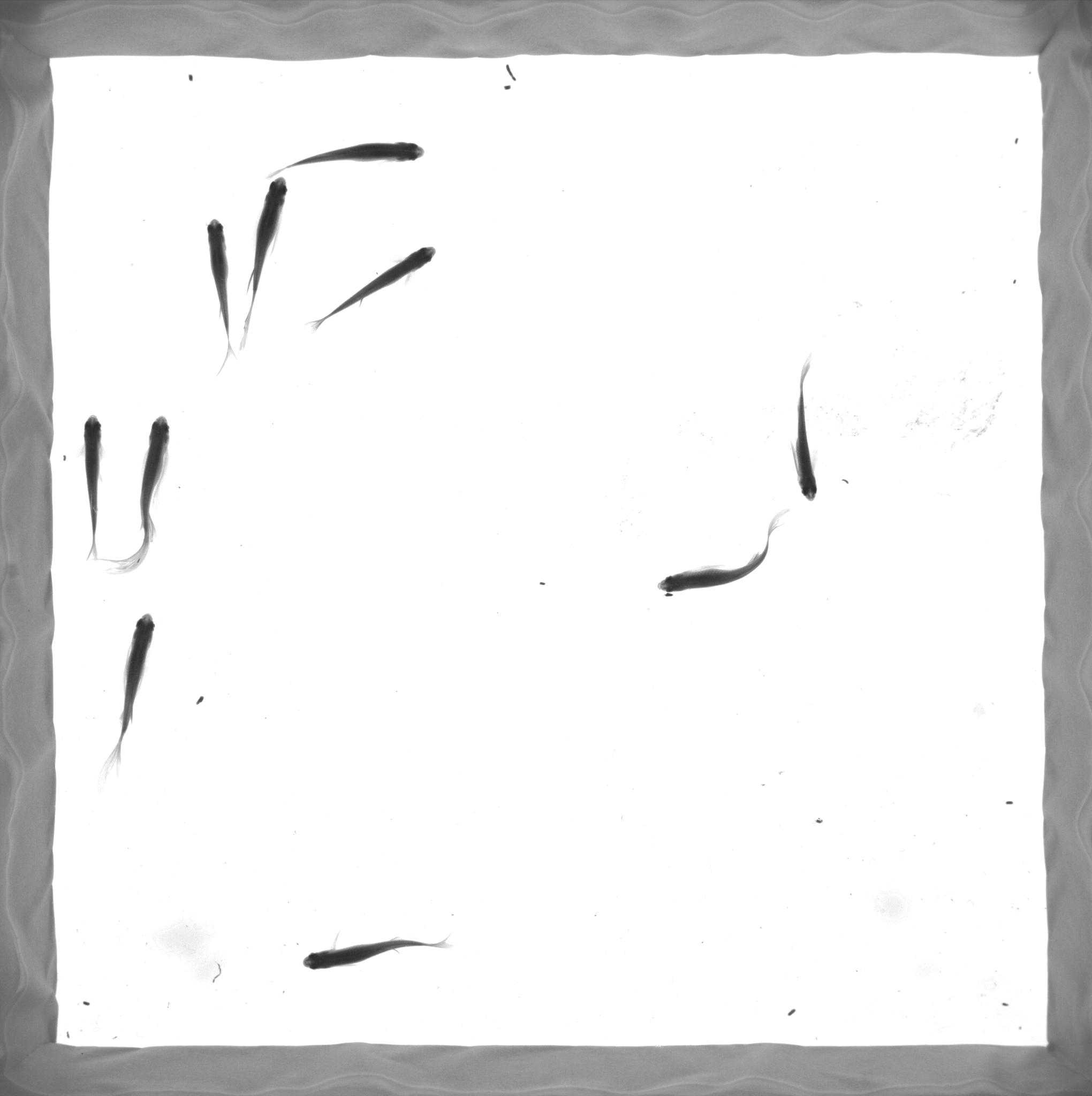

Supplement: S1 File — Source code of the proposed tracking system. (ZIP) [file pone.0154714.s002.zip › code_final/images/CoreView_275_Master_Camera_00181.jpg]

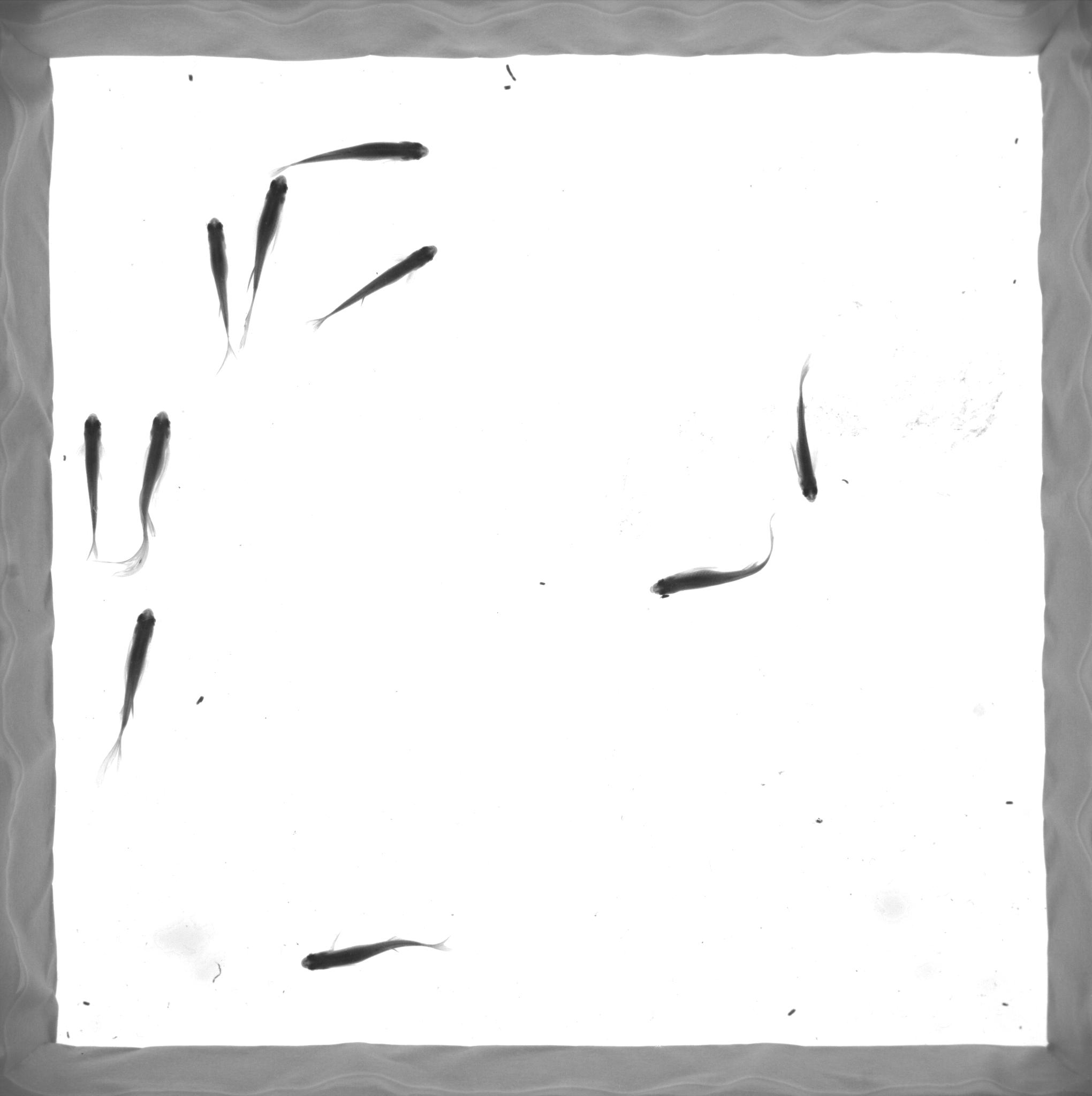

Supplement: S1 File — Source code of the proposed tracking system. (ZIP) [file pone.0154714.s002.zip › code_final/images/CoreView_275_Master_Camera_00182.jpg]

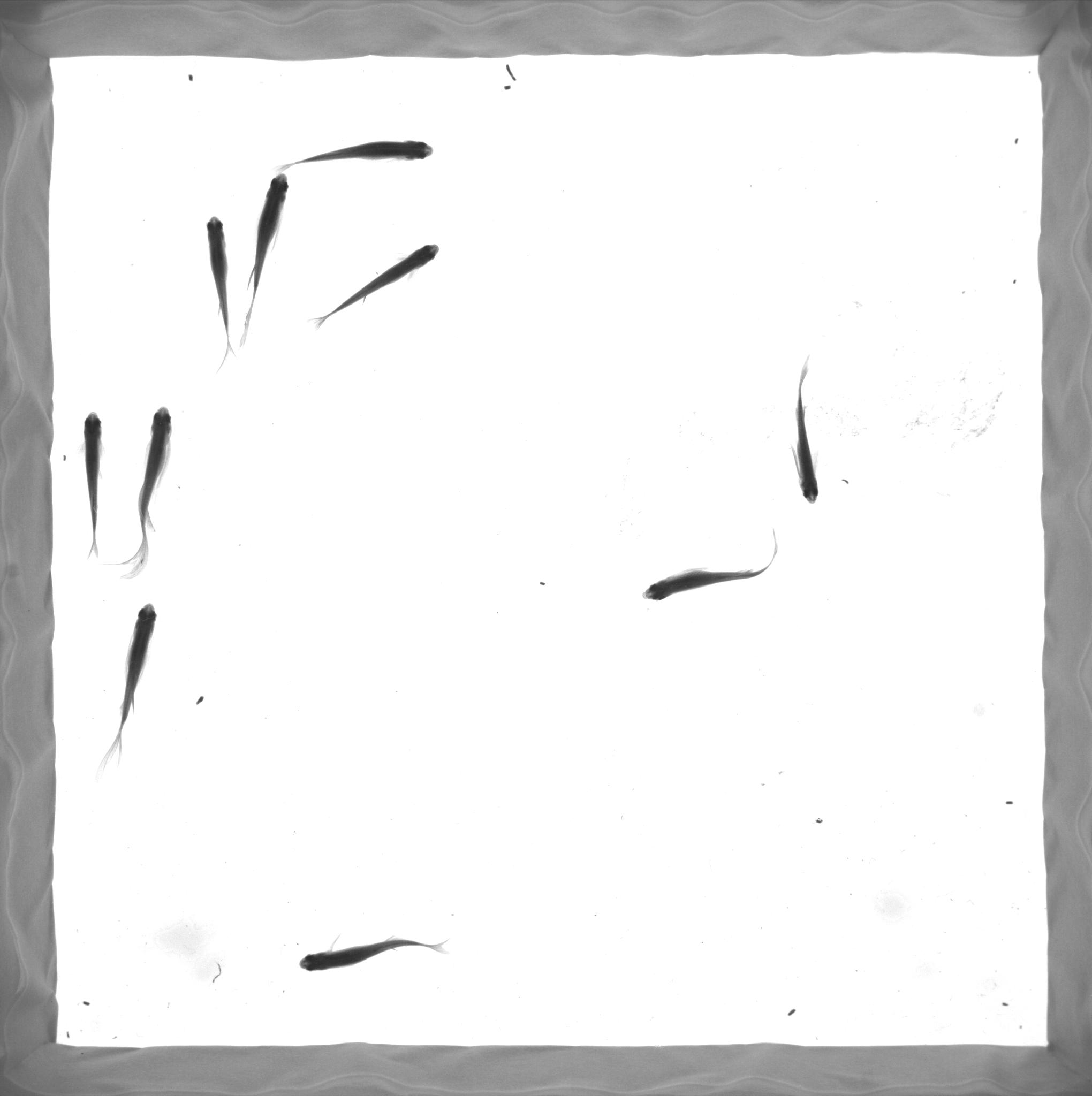

Supplement: S1 File — Source code of the proposed tracking system. (ZIP) [file pone.0154714.s002.zip › code_final/images/CoreView_275_Master_Camera_00183.jpg]

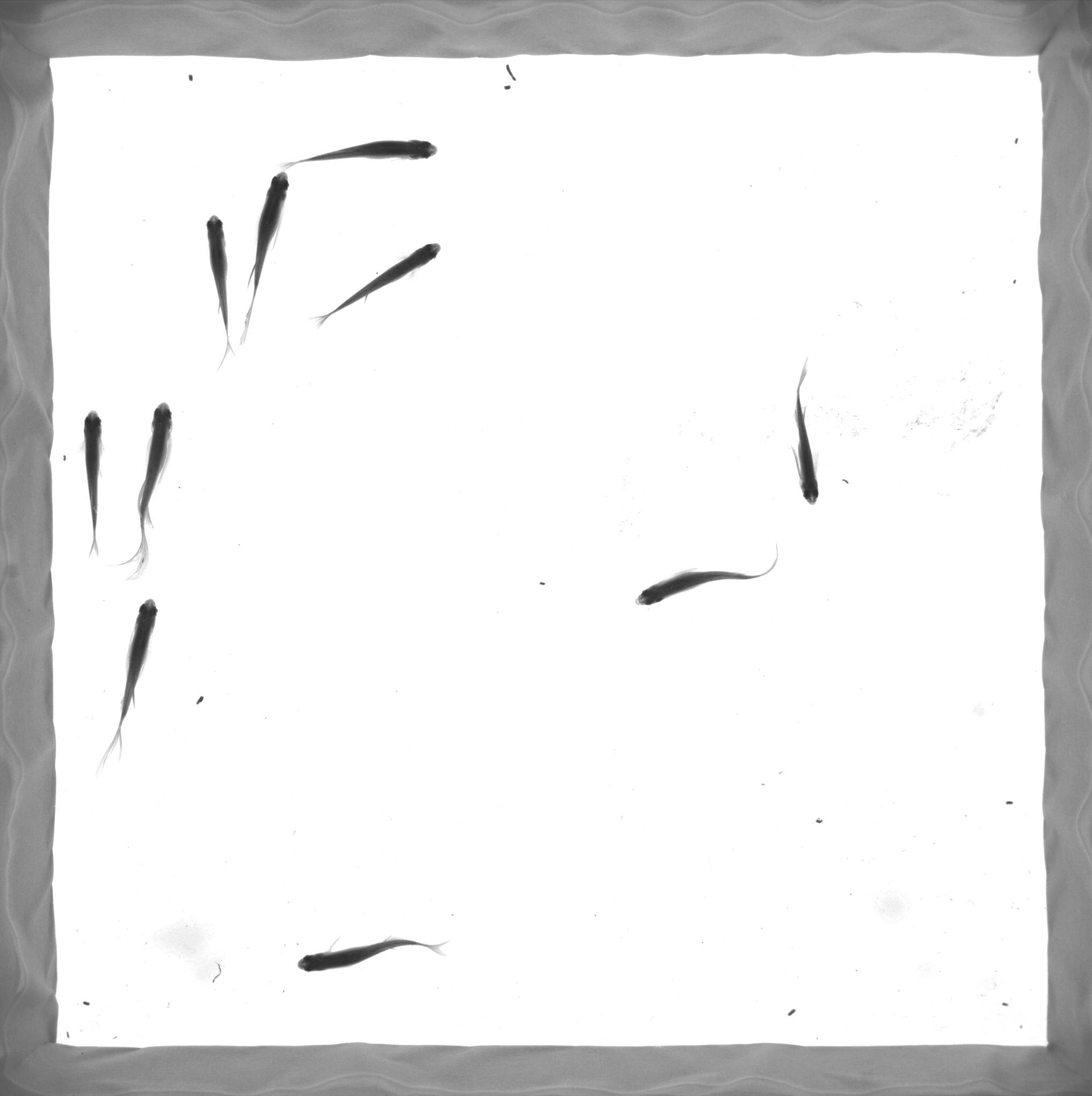

Supplement: S1 File — Source code of the proposed tracking system. (ZIP) [file pone.0154714.s002.zip › code_final/images/CoreView_275_Master_Camera_00184.jpg]

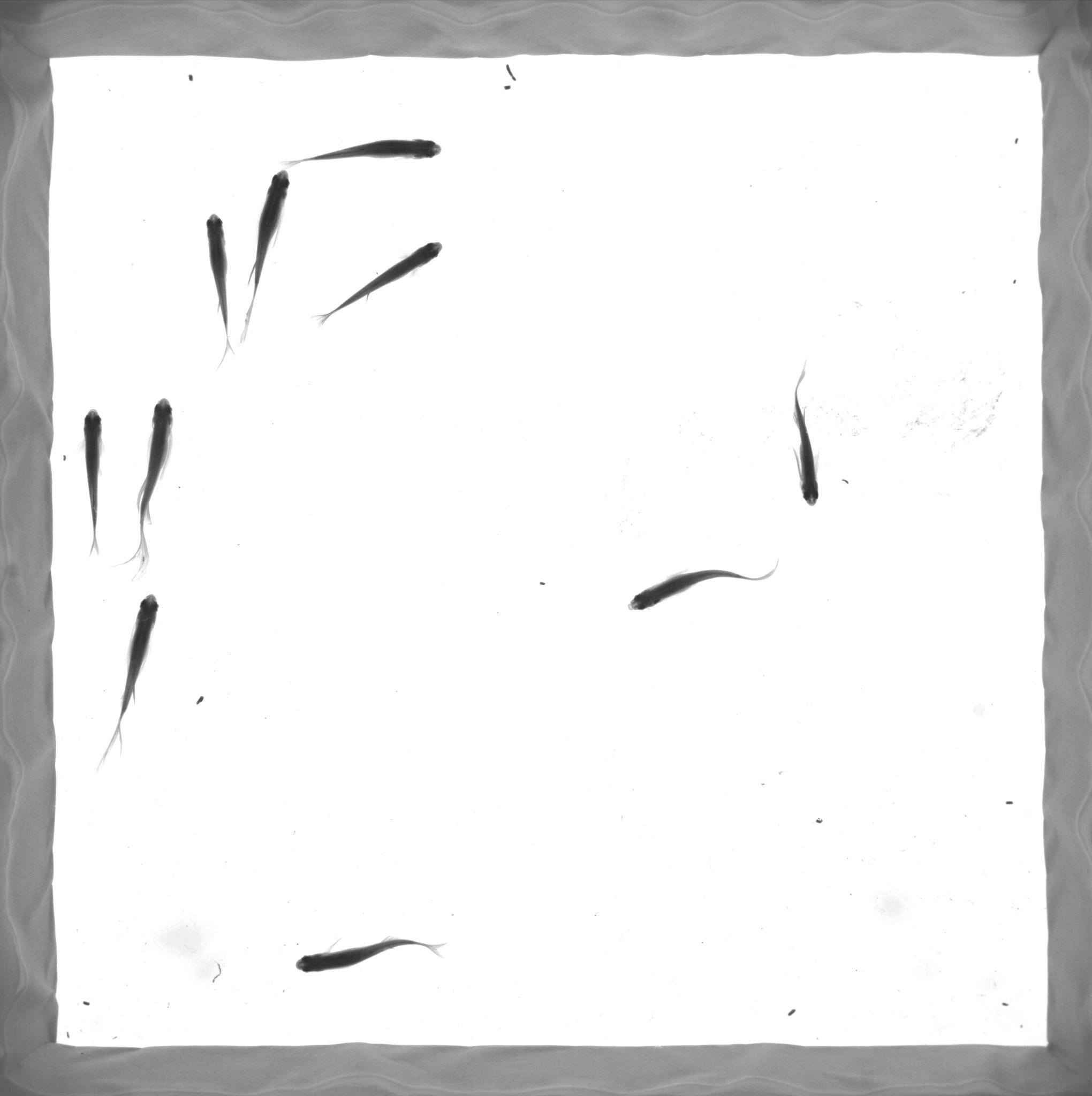

Supplement: S1 File — Source code of the proposed tracking system. (ZIP) [file pone.0154714.s002.zip › code_final/images/CoreView_275_Master_Camera_00185.jpg]

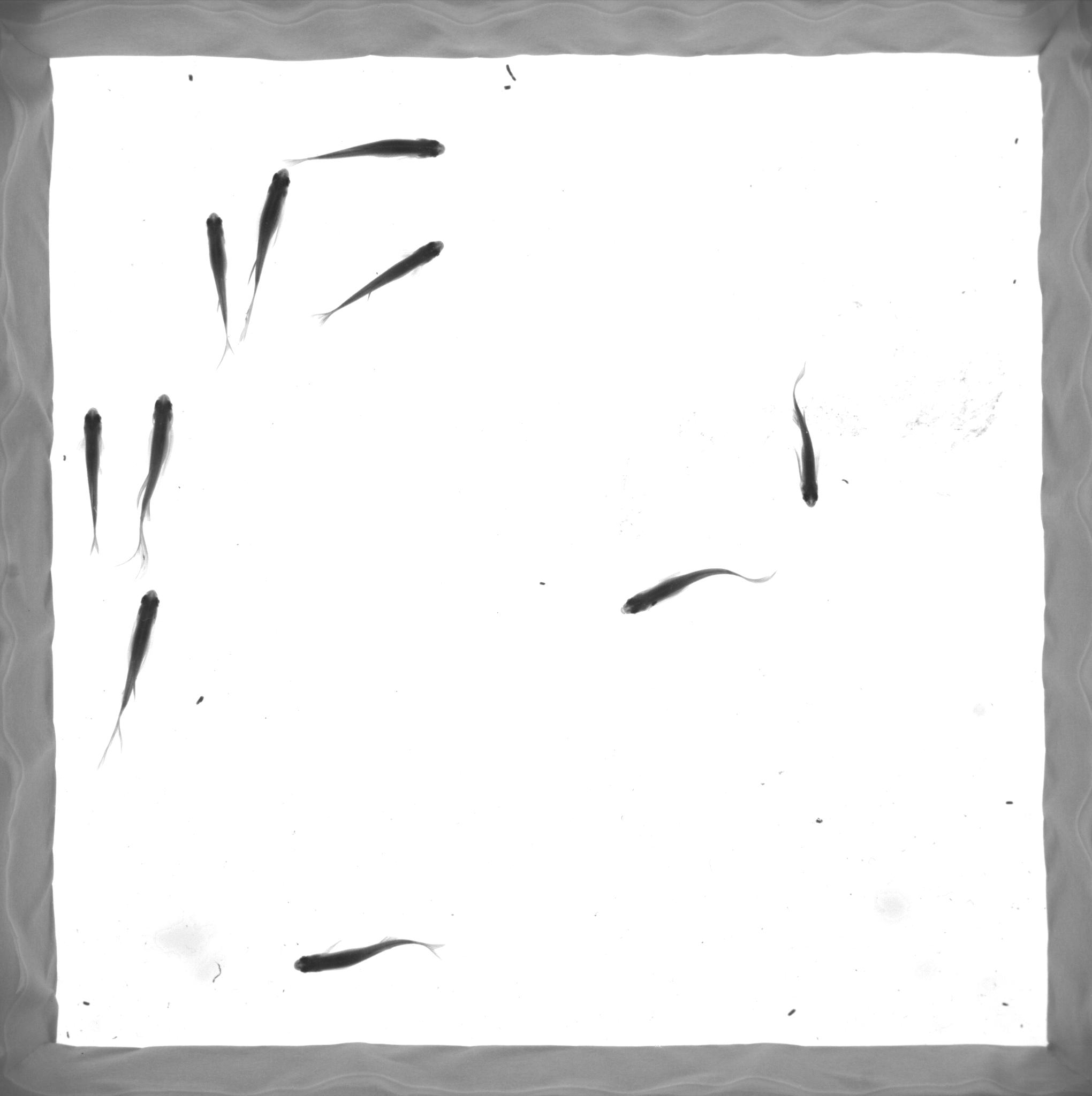

Supplement: S1 File — Source code of the proposed tracking system. (ZIP) [file pone.0154714.s002.zip › code_final/images/CoreView_275_Master_Camera_00186.jpg]

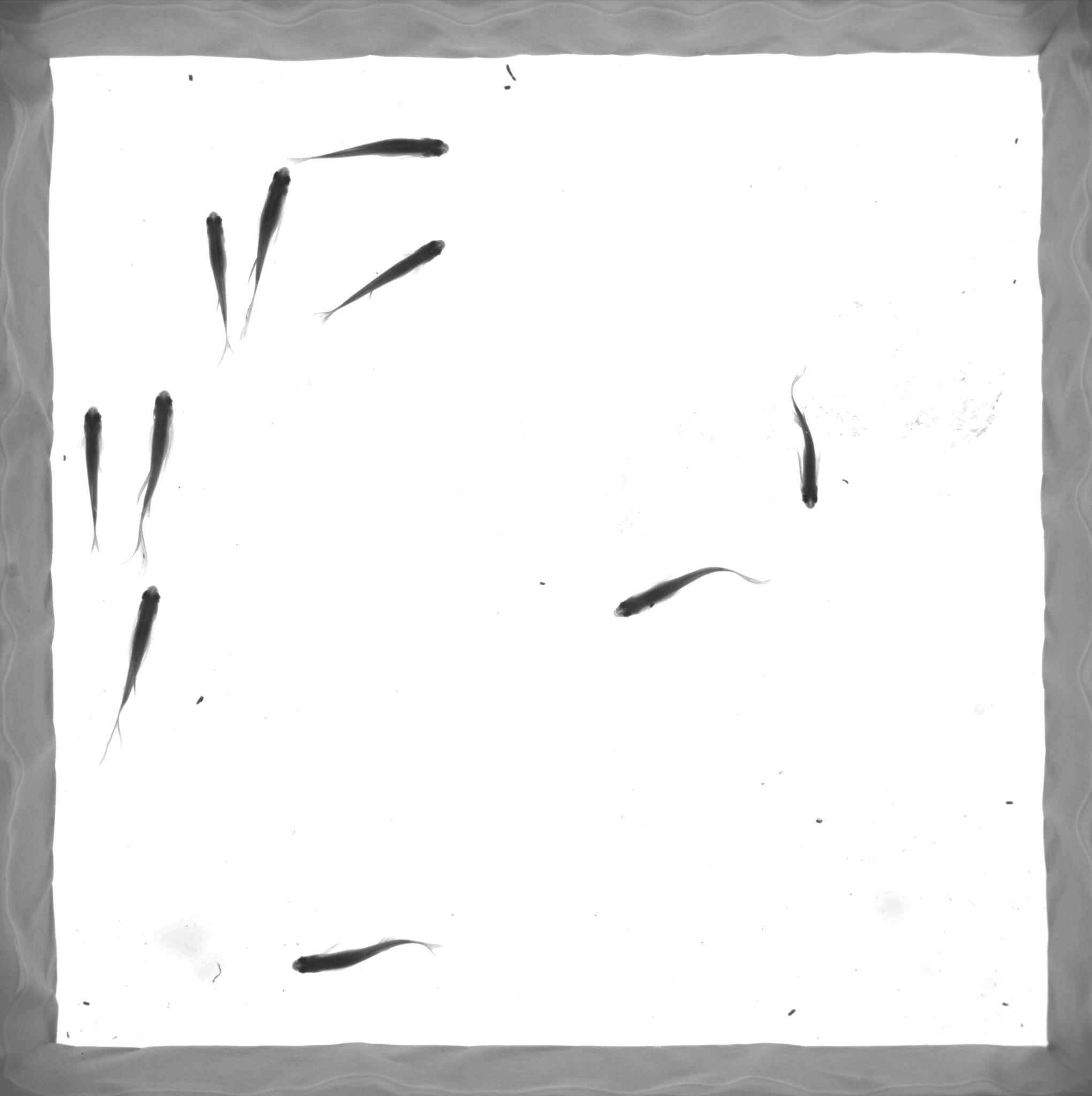

Supplement: S1 File — Source code of the proposed tracking system. (ZIP) [file pone.0154714.s002.zip › code_final/images/CoreView_275_Master_Camera_00187.jpg]

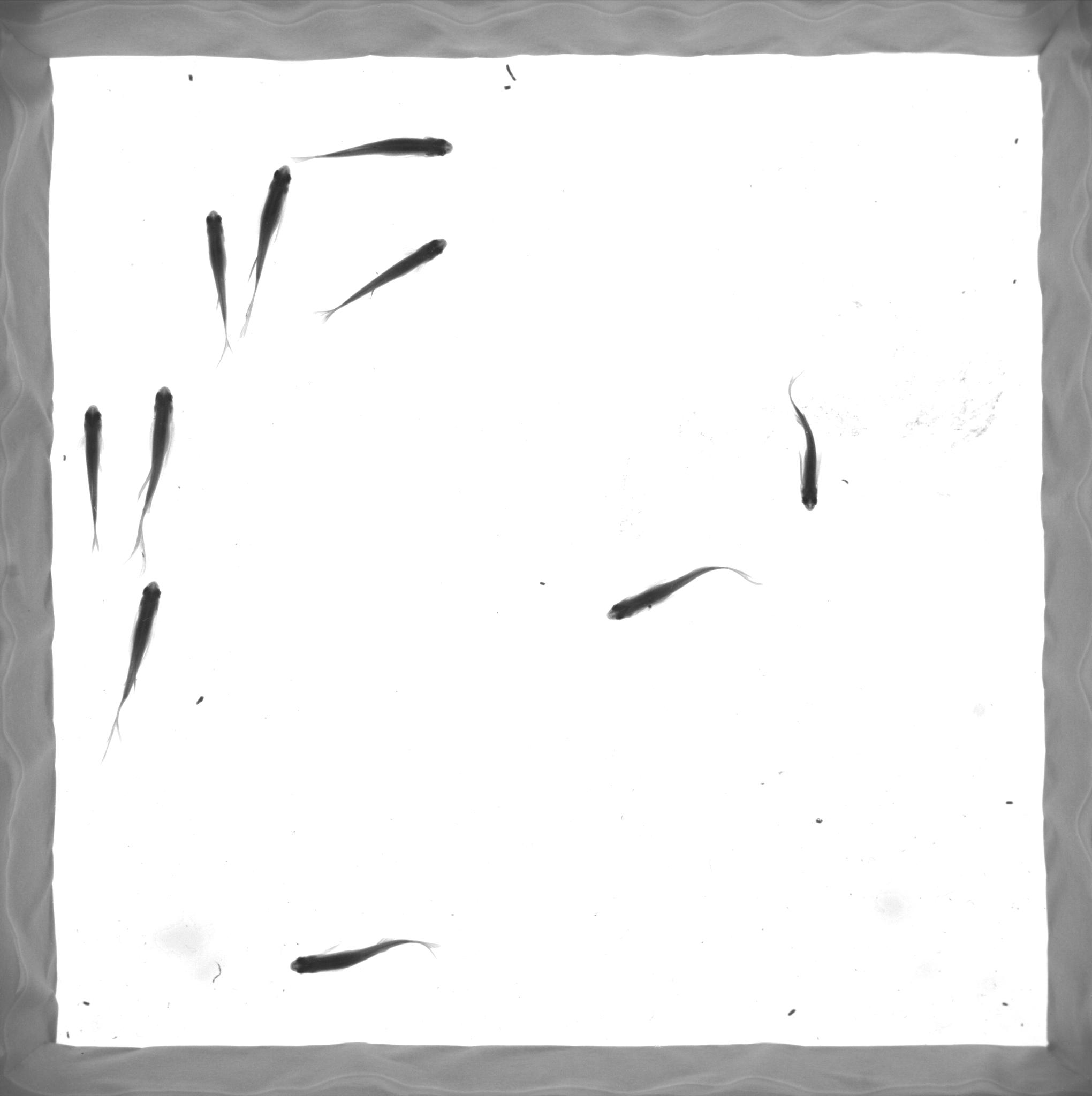

Supplement: S1 File — Source code of the proposed tracking system. (ZIP) [file pone.0154714.s002.zip › code_final/images/CoreView_275_Master_Camera_00188.jpg]

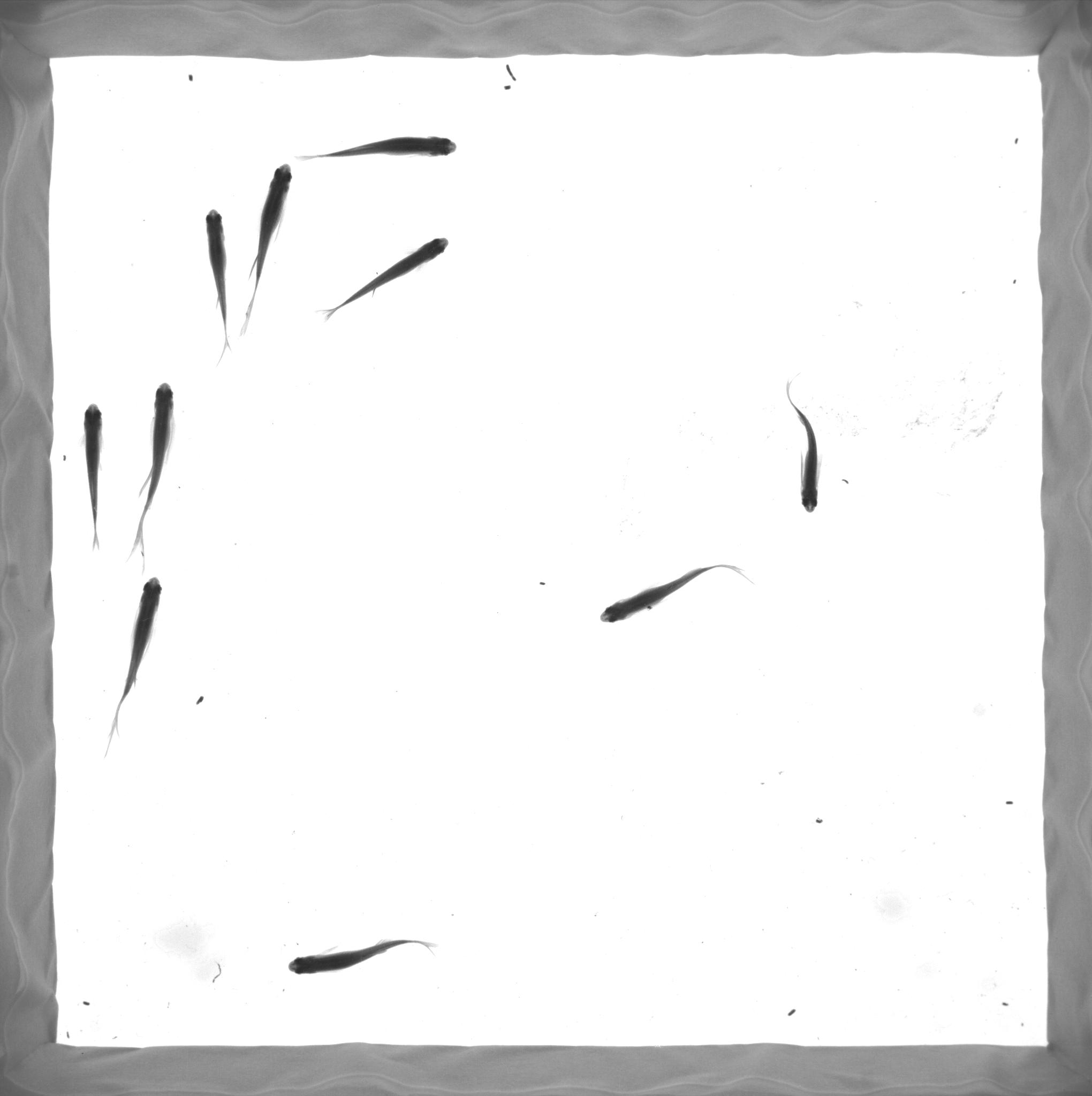

Supplement: S1 File — Source code of the proposed tracking system. (ZIP) [file pone.0154714.s002.zip › code_final/images/CoreView_275_Master_Camera_00189.jpg]

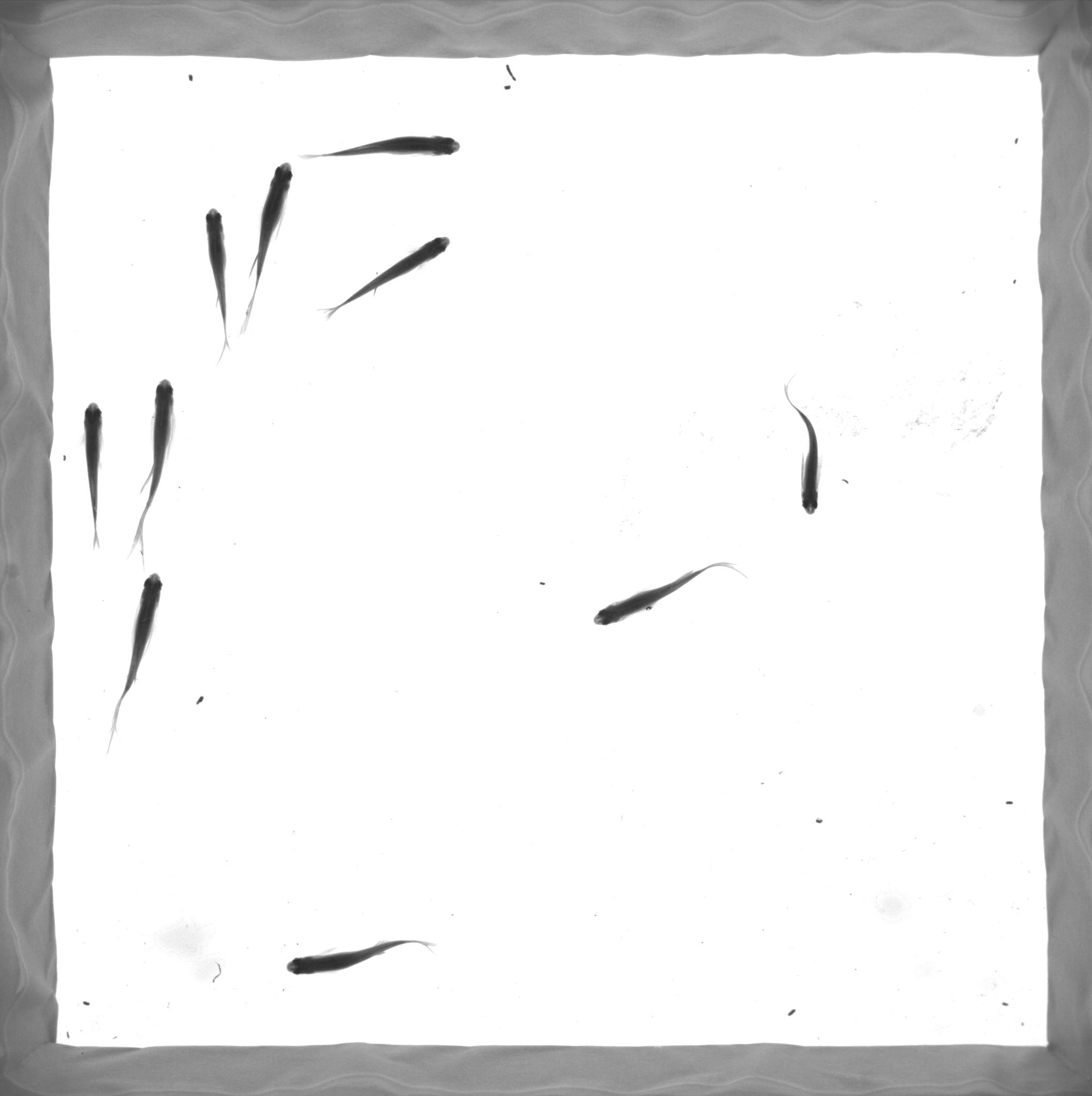

Supplement: S1 File — Source code of the proposed tracking system. (ZIP) [file pone.0154714.s002.zip › code_final/images/CoreView_275_Master_Camera_00190.jpg]

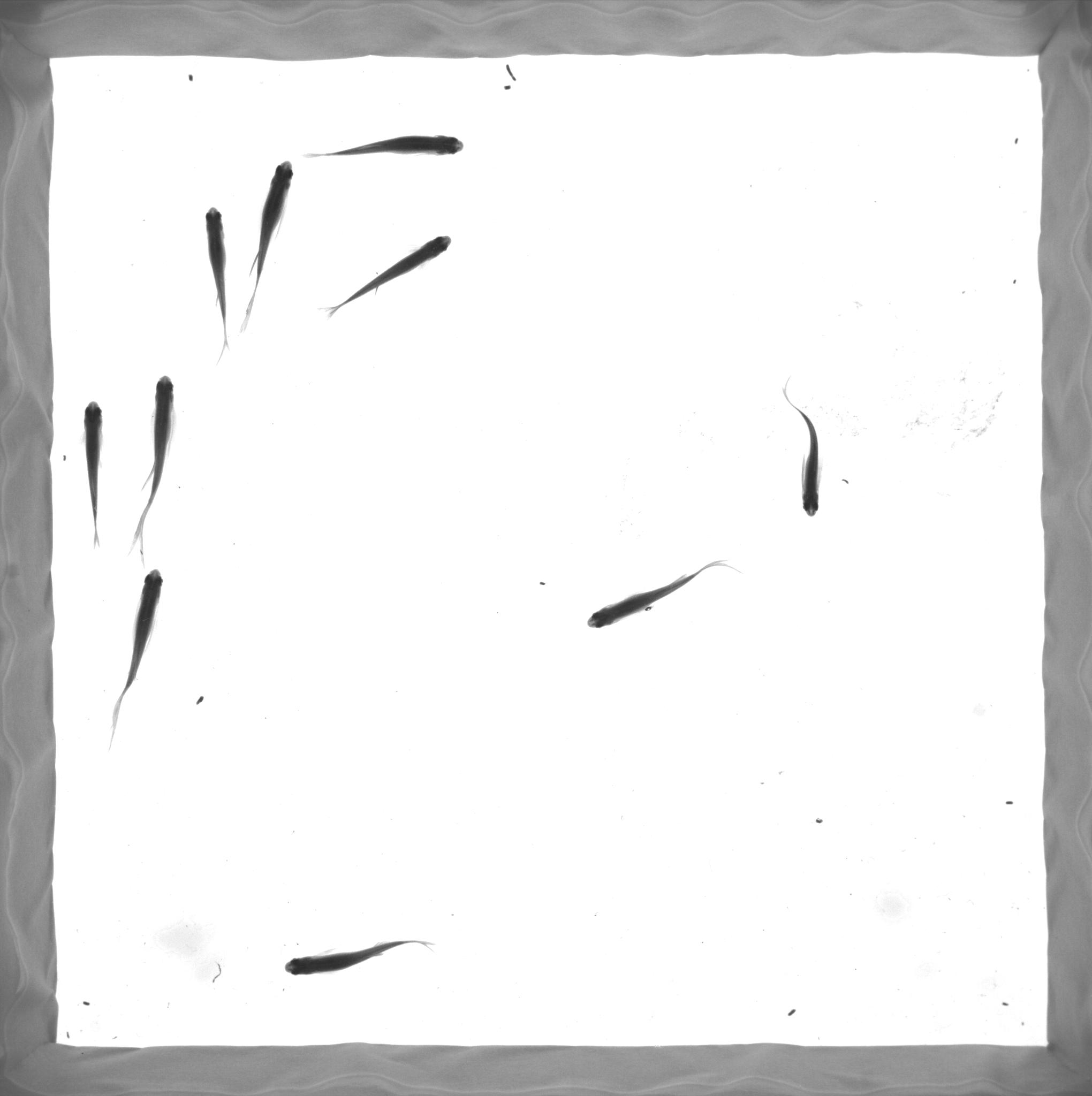

Supplement: S1 File — Source code of the proposed tracking system. (ZIP) [file pone.0154714.s002.zip › code_final/images/CoreView_275_Master_Camera_00191.jpg]

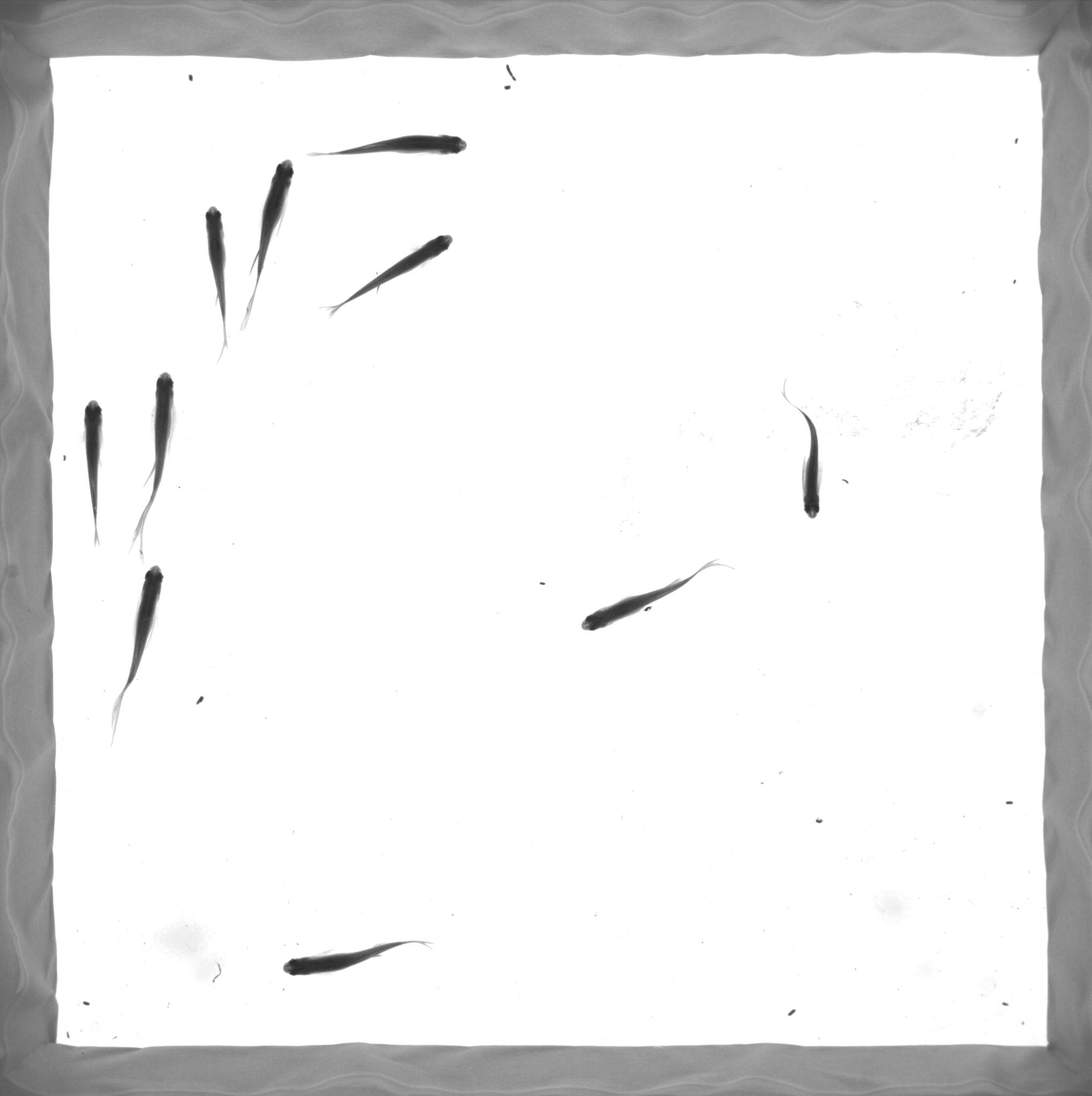

Supplement: S1 File — Source code of the proposed tracking system. (ZIP) [file pone.0154714.s002.zip › code_final/images/CoreView_275_Master_Camera_00192.jpg]

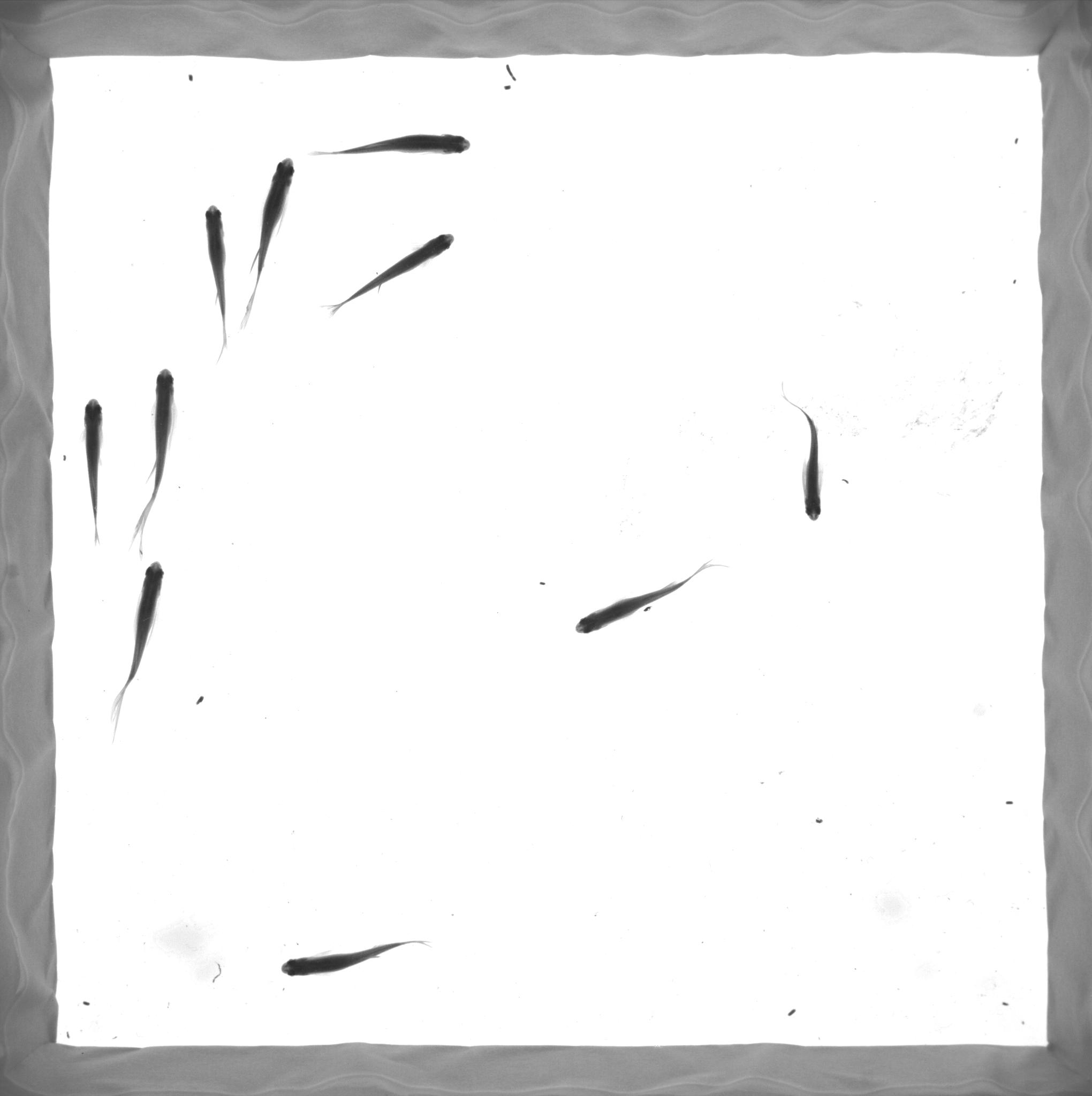

Supplement: S1 File — Source code of the proposed tracking system. (ZIP) [file pone.0154714.s002.zip › code_final/images/CoreView_275_Master_Camera_00193.jpg]
