# Supplementary figures and images for: Automated Planar Tracking the Waving Bodies of Multiple Zebrafish Swimming in Shallow Water (part 1 of 2)
Source: PLoS One. 2016 Apr 29;11(4):e0154714. doi: 10.1371/journal.pone.0154714 (PMC4851353; doi:10.1371/journal.pone.0154714)

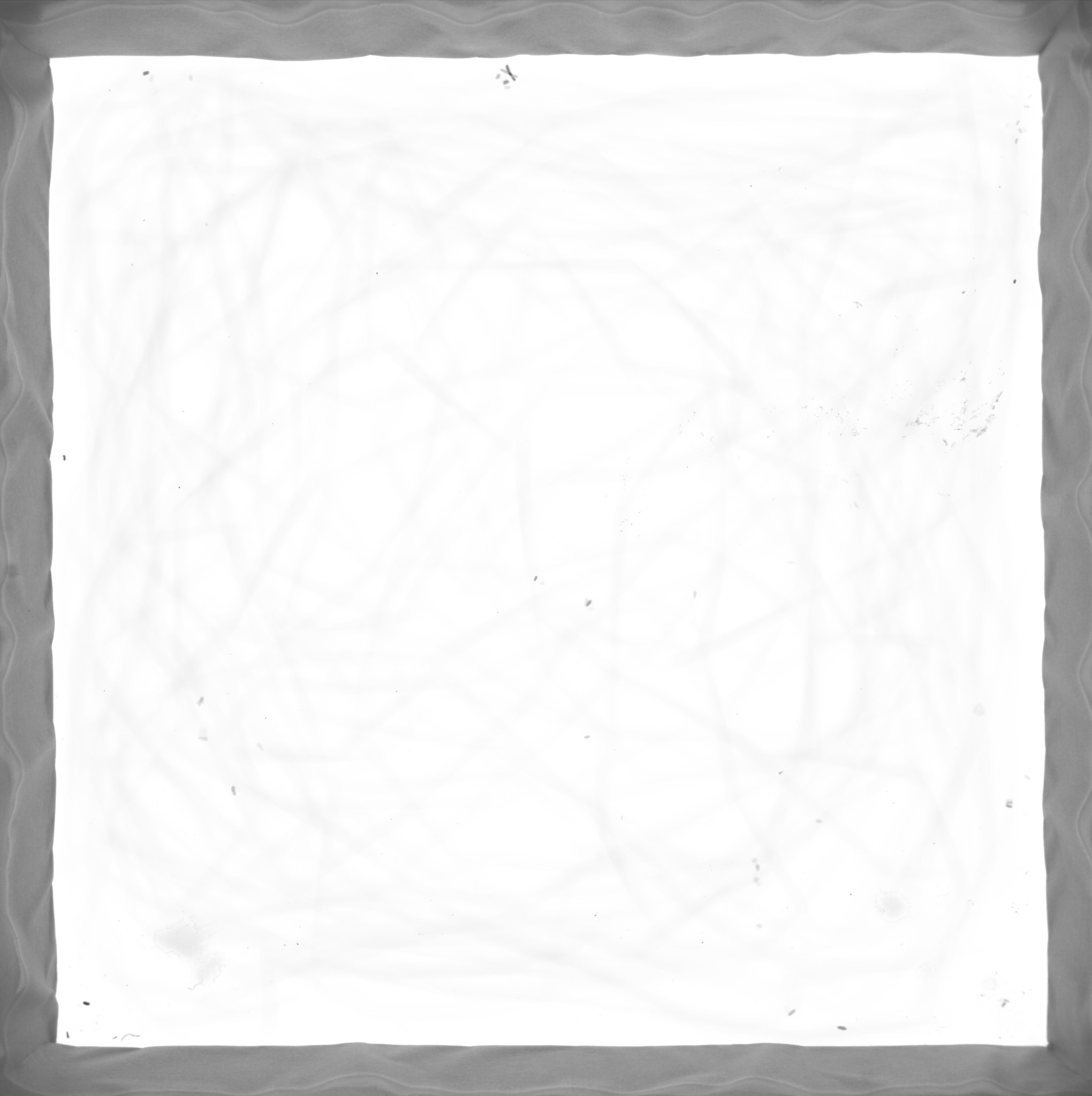

Supplement: S1 File — Source code of the proposed tracking system. (ZIP) [file pone.0154714.s002.zip › code_final/bkd_275.bmp]

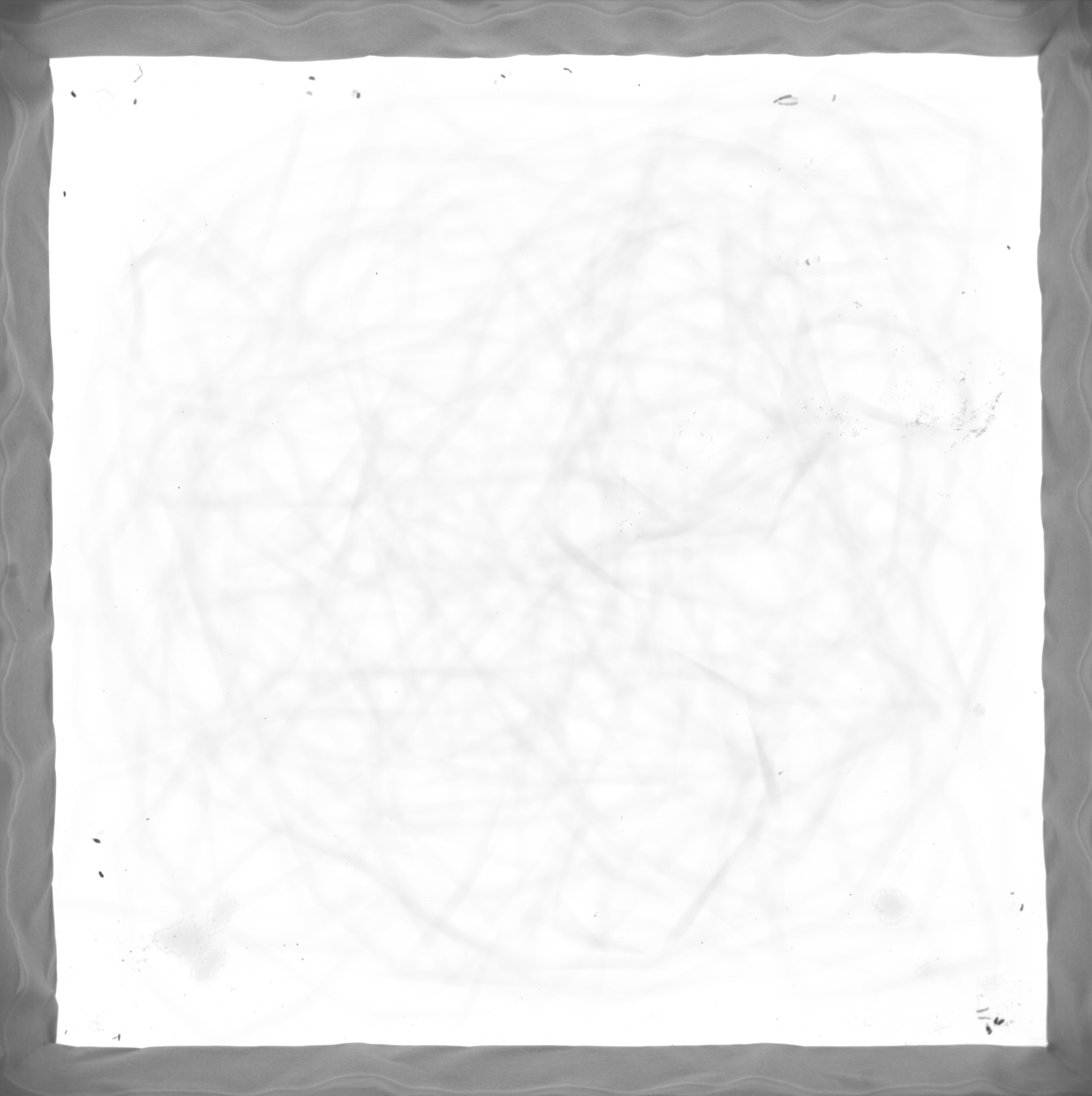

Supplement: S1 File — Source code of the proposed tracking system. (ZIP) [file pone.0154714.s002.zip › code_final/bkd_278.bmp]

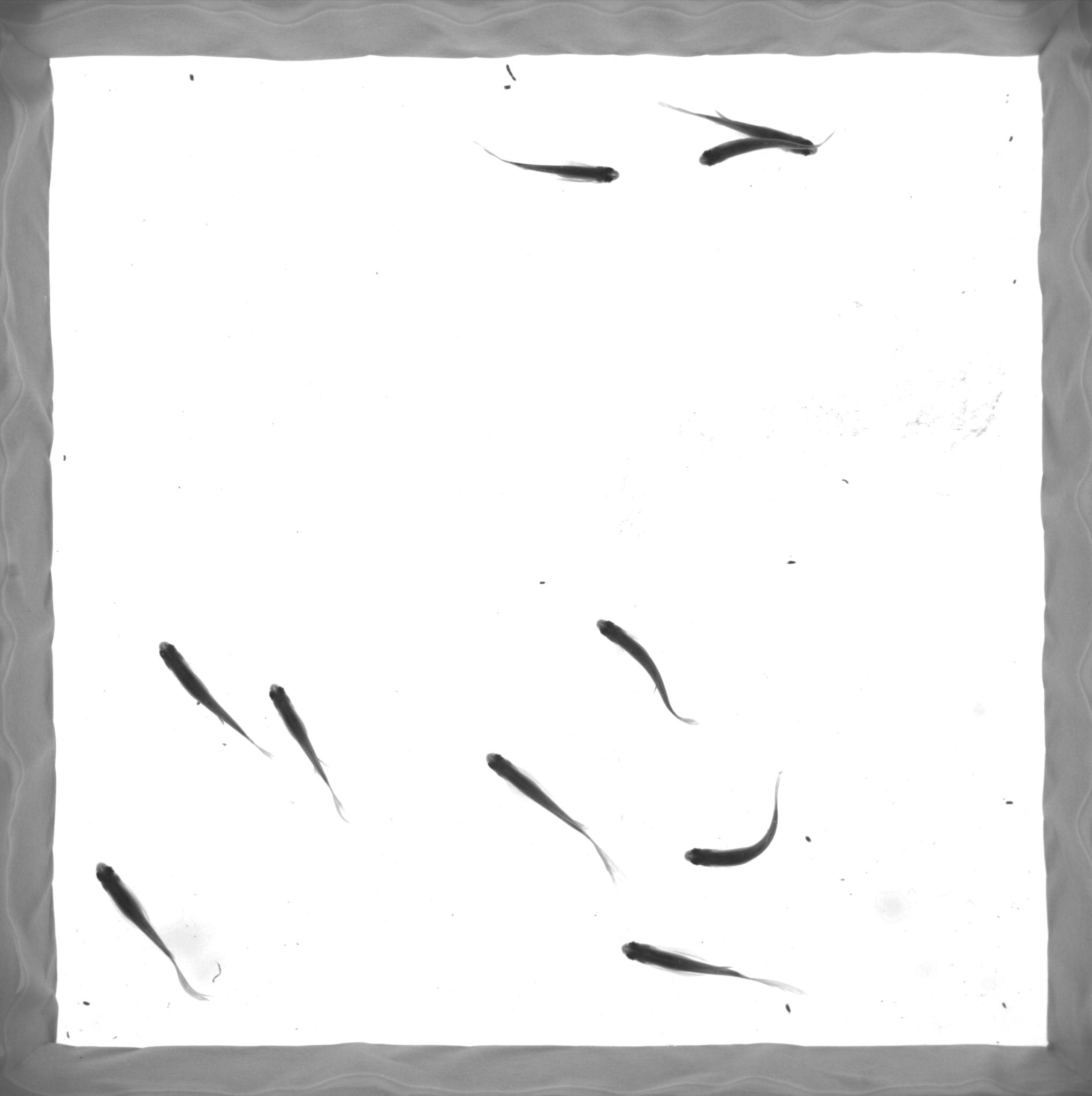

Supplement: S1 File — Source code of the proposed tracking system. (ZIP) [file pone.0154714.s002.zip › code_final/images/CoreView_275_Master_Camera_00001.jpg]

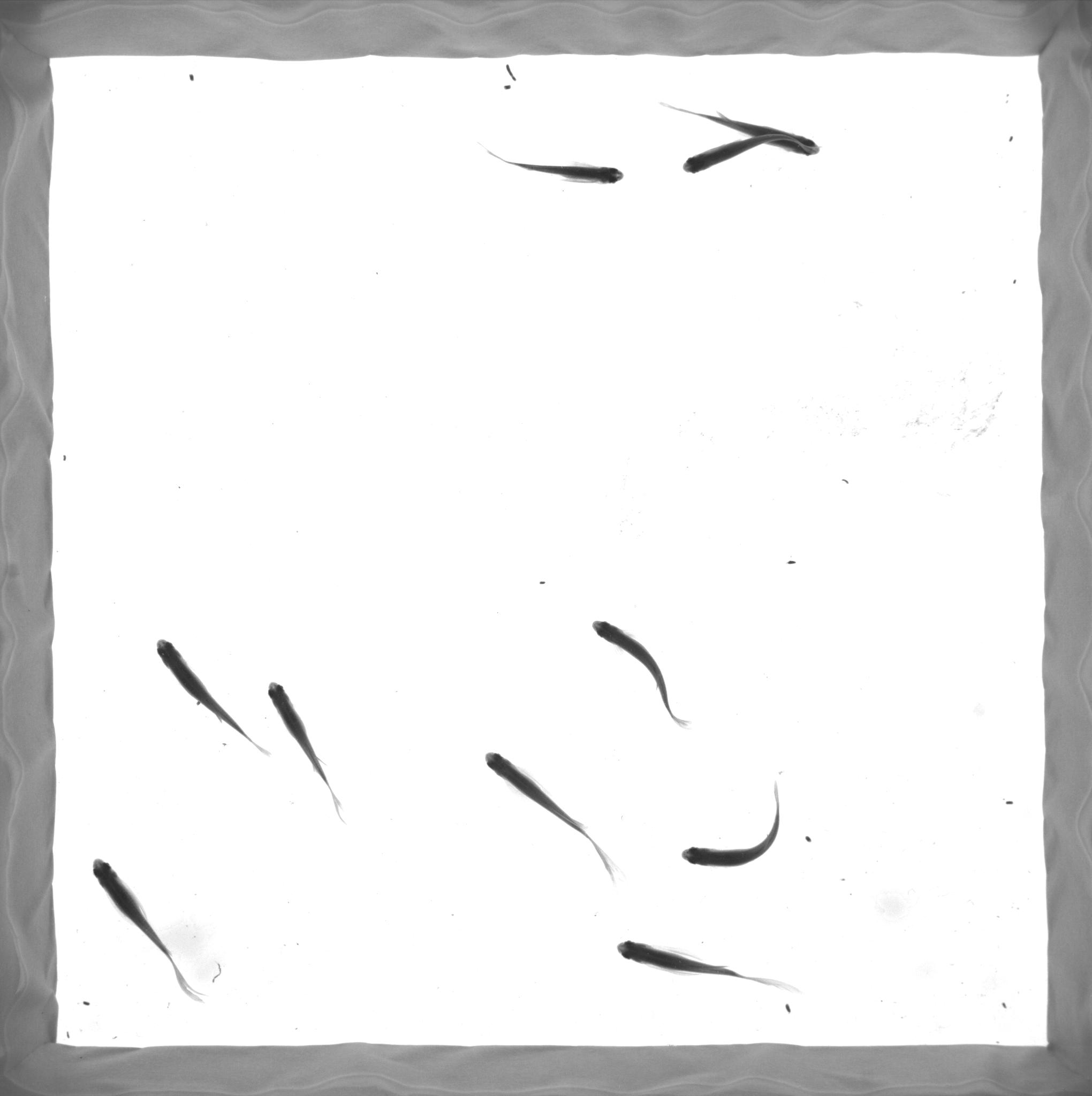

Supplement: S1 File — Source code of the proposed tracking system. (ZIP) [file pone.0154714.s002.zip › code_final/images/CoreView_275_Master_Camera_00002.jpg]

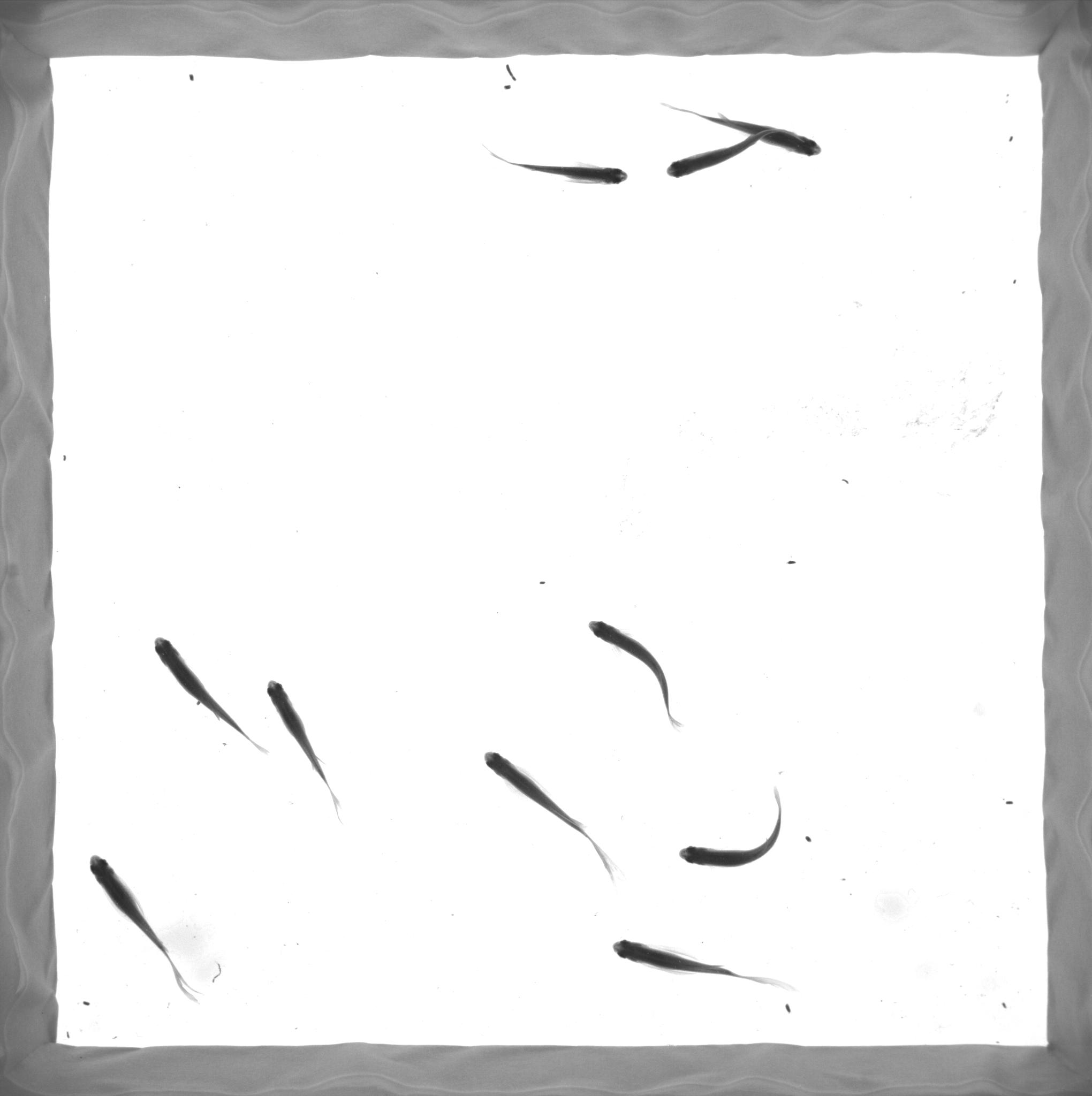

Supplement: S1 File — Source code of the proposed tracking system. (ZIP) [file pone.0154714.s002.zip › code_final/images/CoreView_275_Master_Camera_00003.jpg]

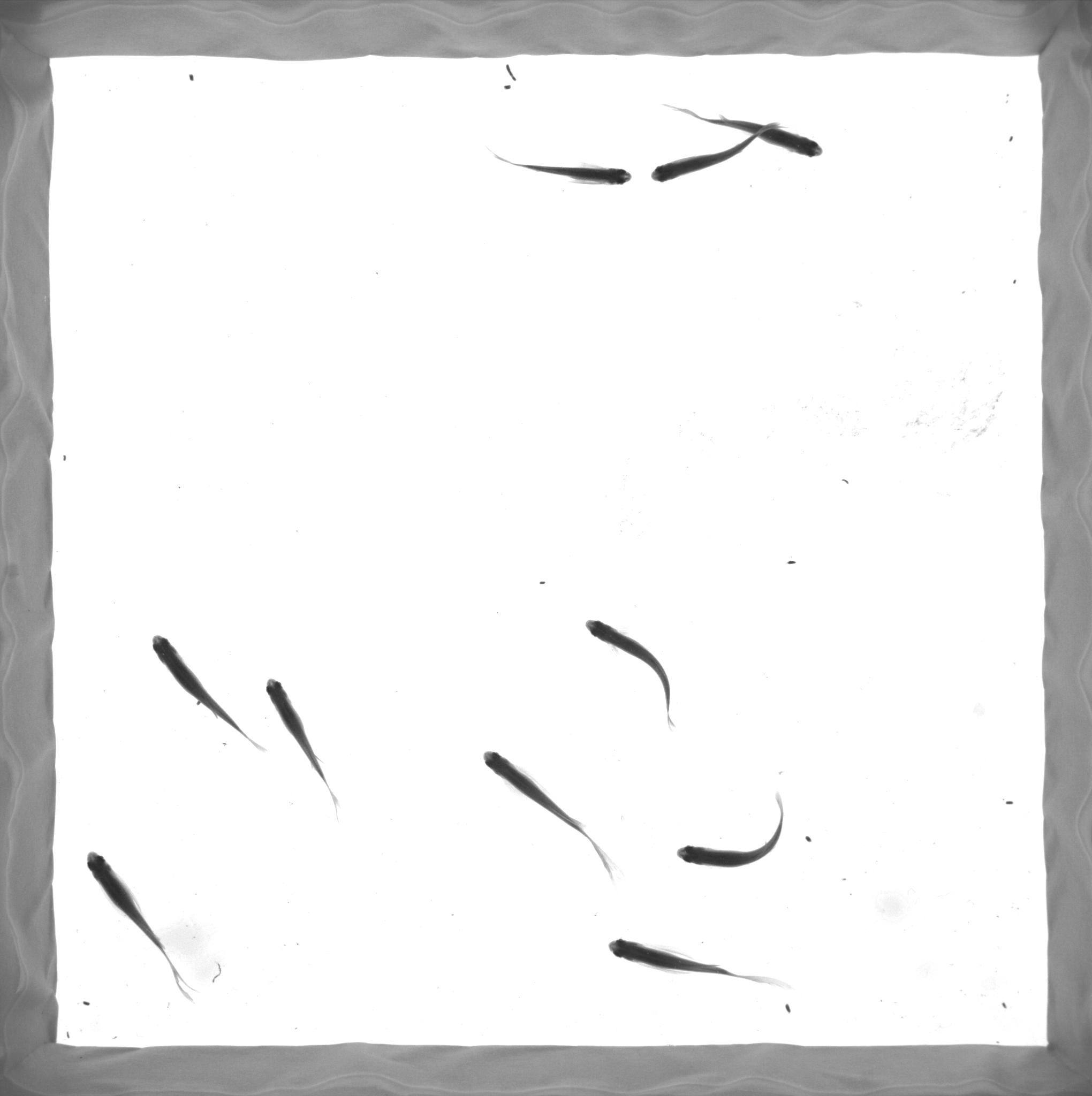

Supplement: S1 File — Source code of the proposed tracking system. (ZIP) [file pone.0154714.s002.zip › code_final/images/CoreView_275_Master_Camera_00004.jpg]

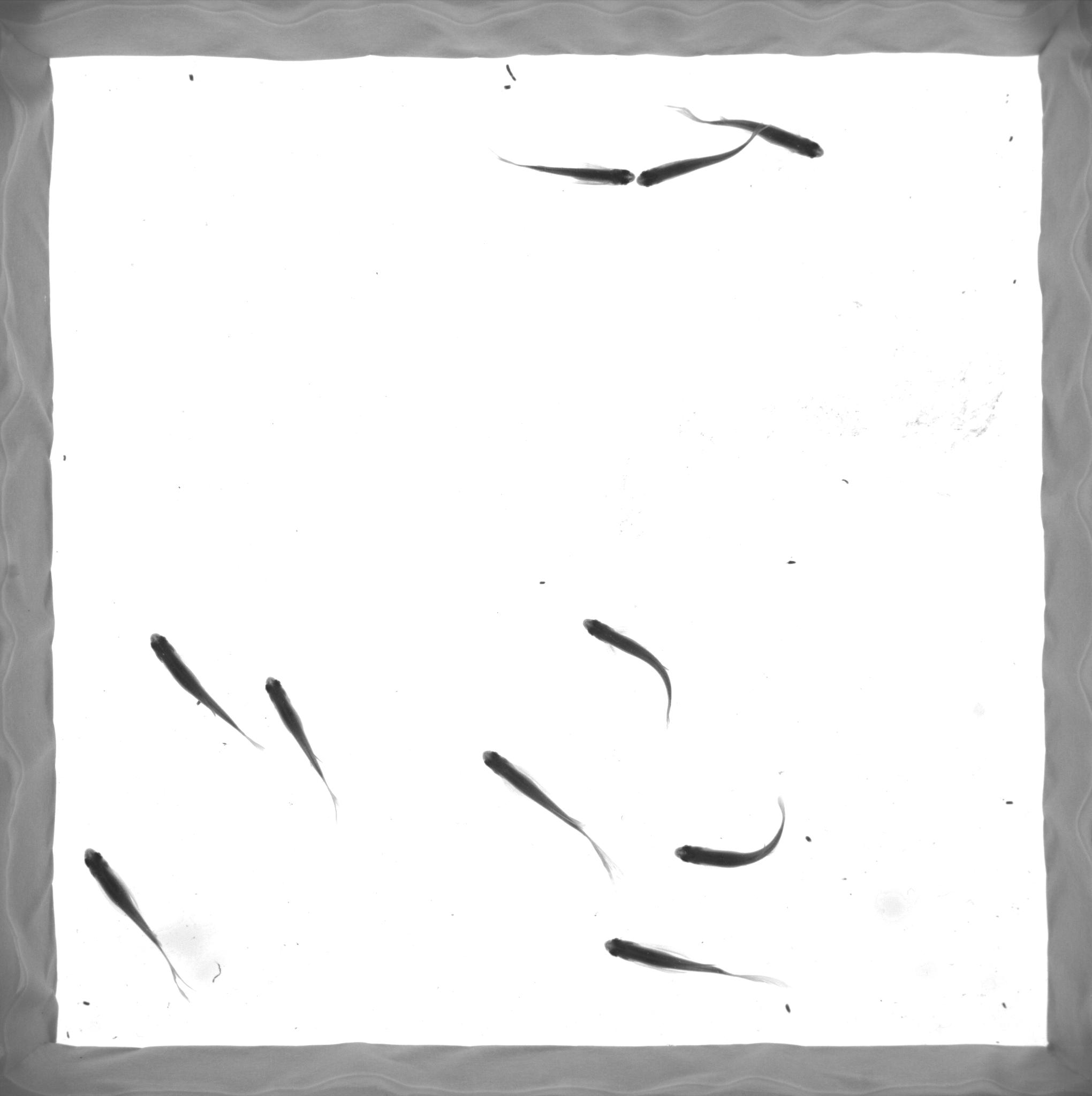

Supplement: S1 File — Source code of the proposed tracking system. (ZIP) [file pone.0154714.s002.zip › code_final/images/CoreView_275_Master_Camera_00005.jpg]

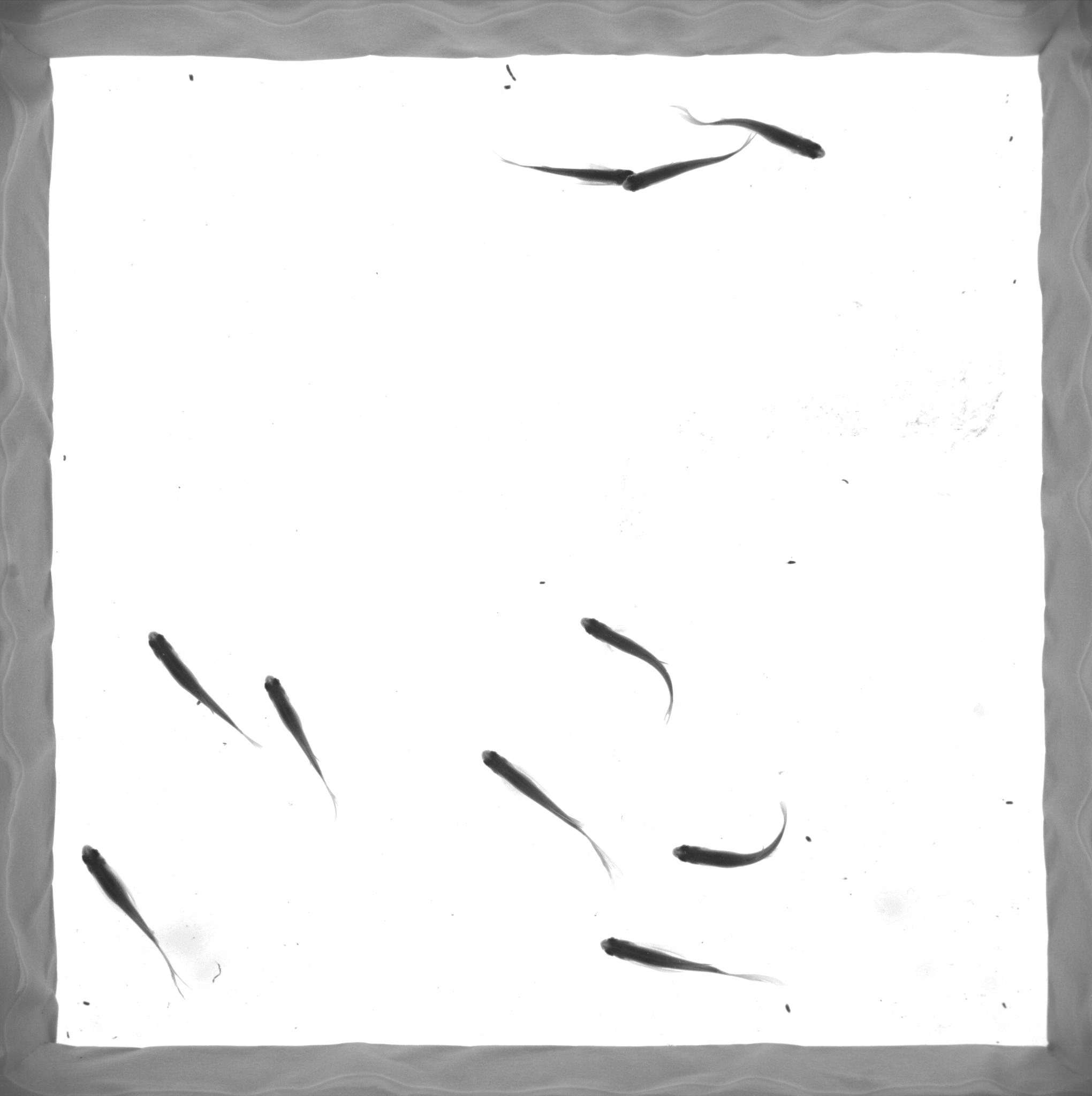

Supplement: S1 File — Source code of the proposed tracking system. (ZIP) [file pone.0154714.s002.zip › code_final/images/CoreView_275_Master_Camera_00006.jpg]

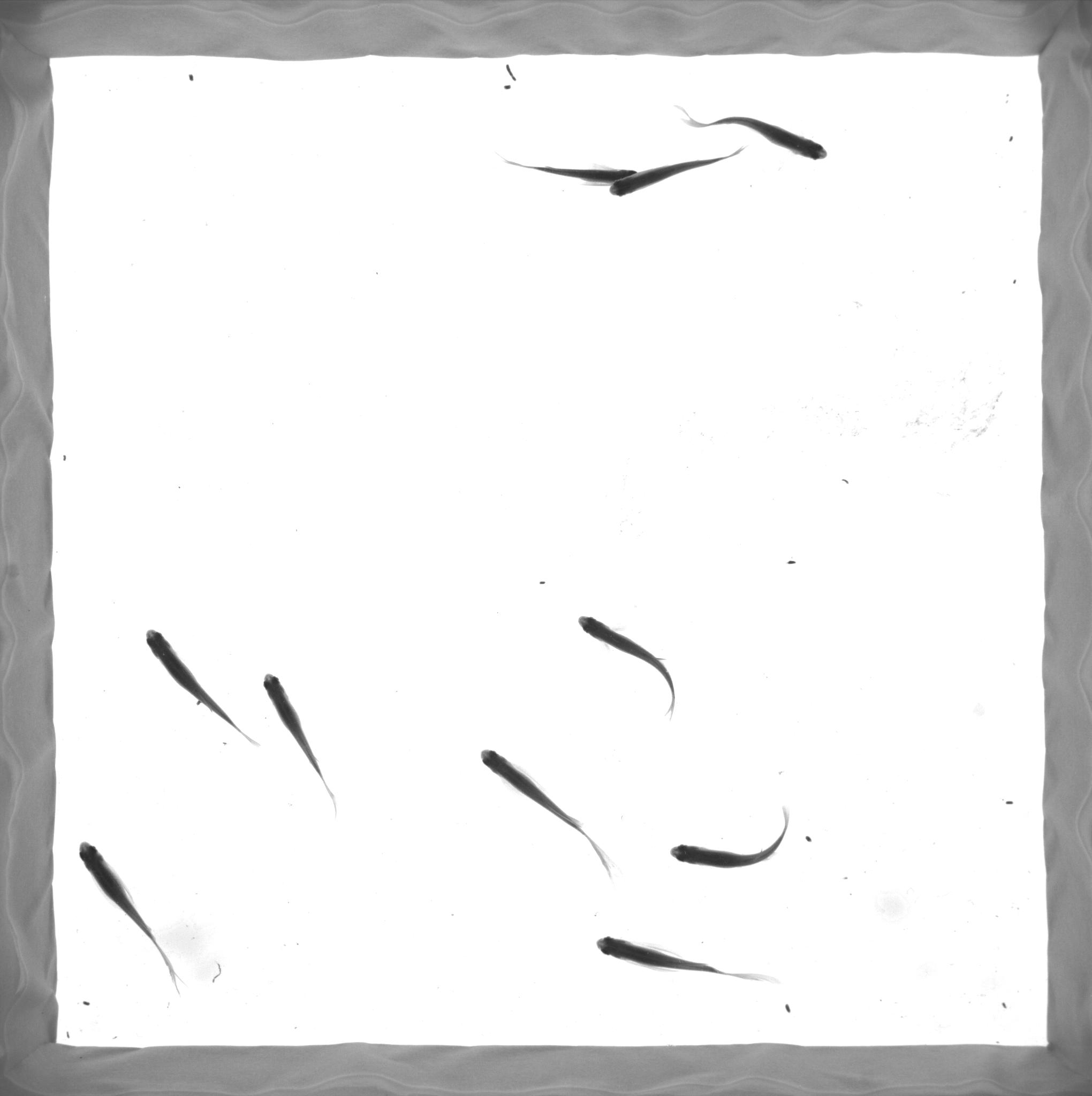

Supplement: S1 File — Source code of the proposed tracking system. (ZIP) [file pone.0154714.s002.zip › code_final/images/CoreView_275_Master_Camera_00007.jpg]

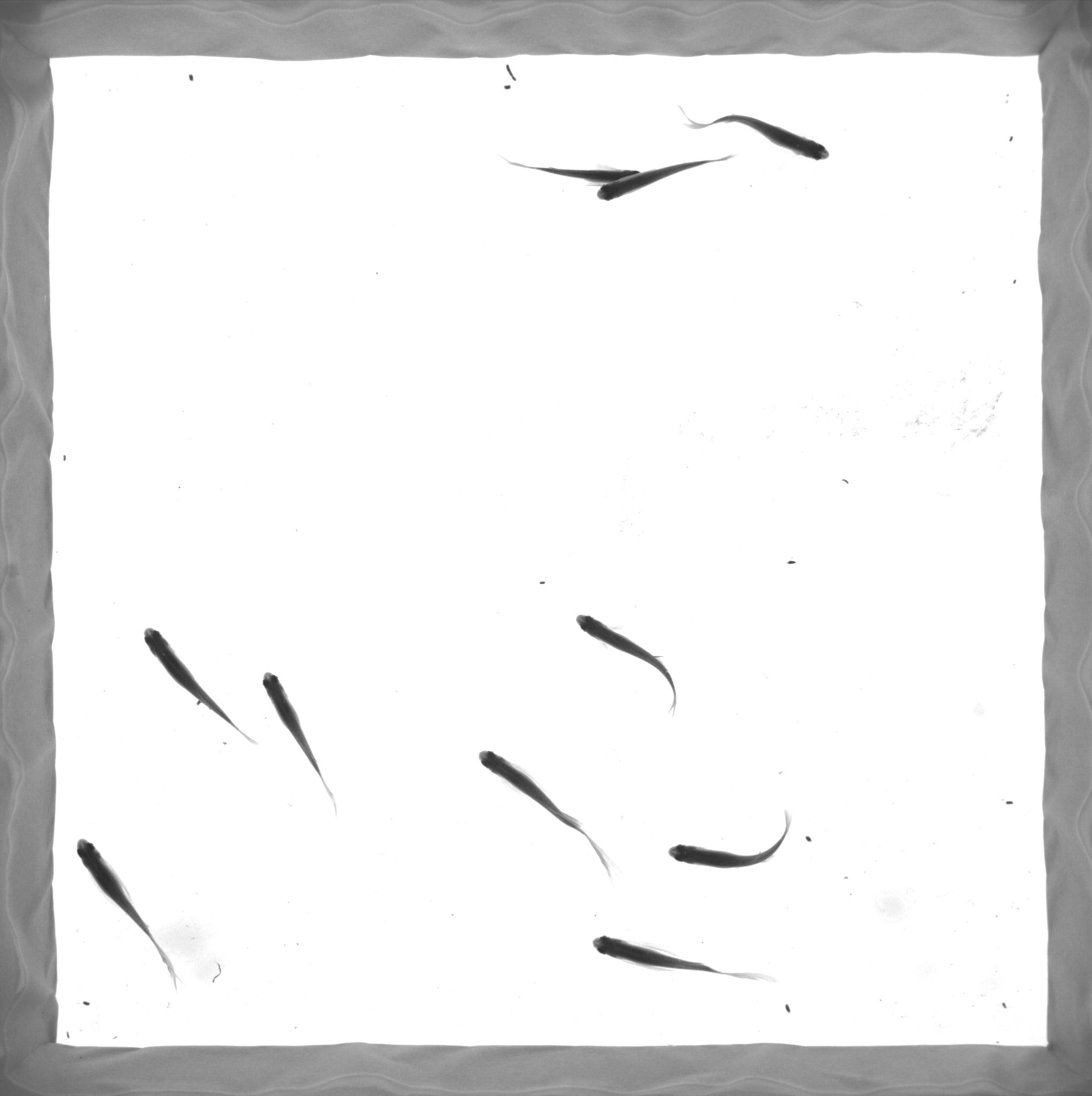

Supplement: S1 File — Source code of the proposed tracking system. (ZIP) [file pone.0154714.s002.zip › code_final/images/CoreView_275_Master_Camera_00008.jpg]

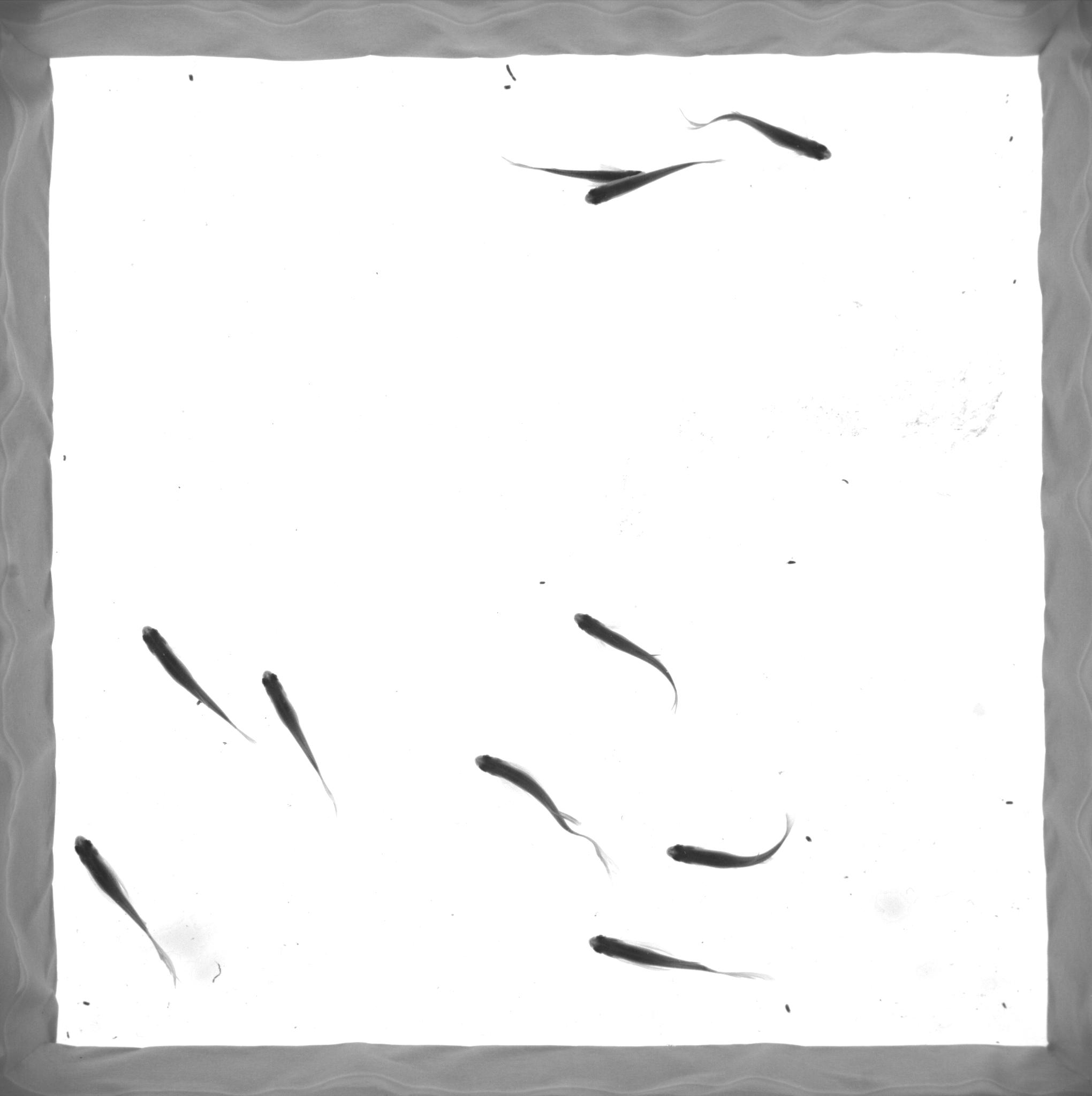

Supplement: S1 File — Source code of the proposed tracking system. (ZIP) [file pone.0154714.s002.zip › code_final/images/CoreView_275_Master_Camera_00009.jpg]

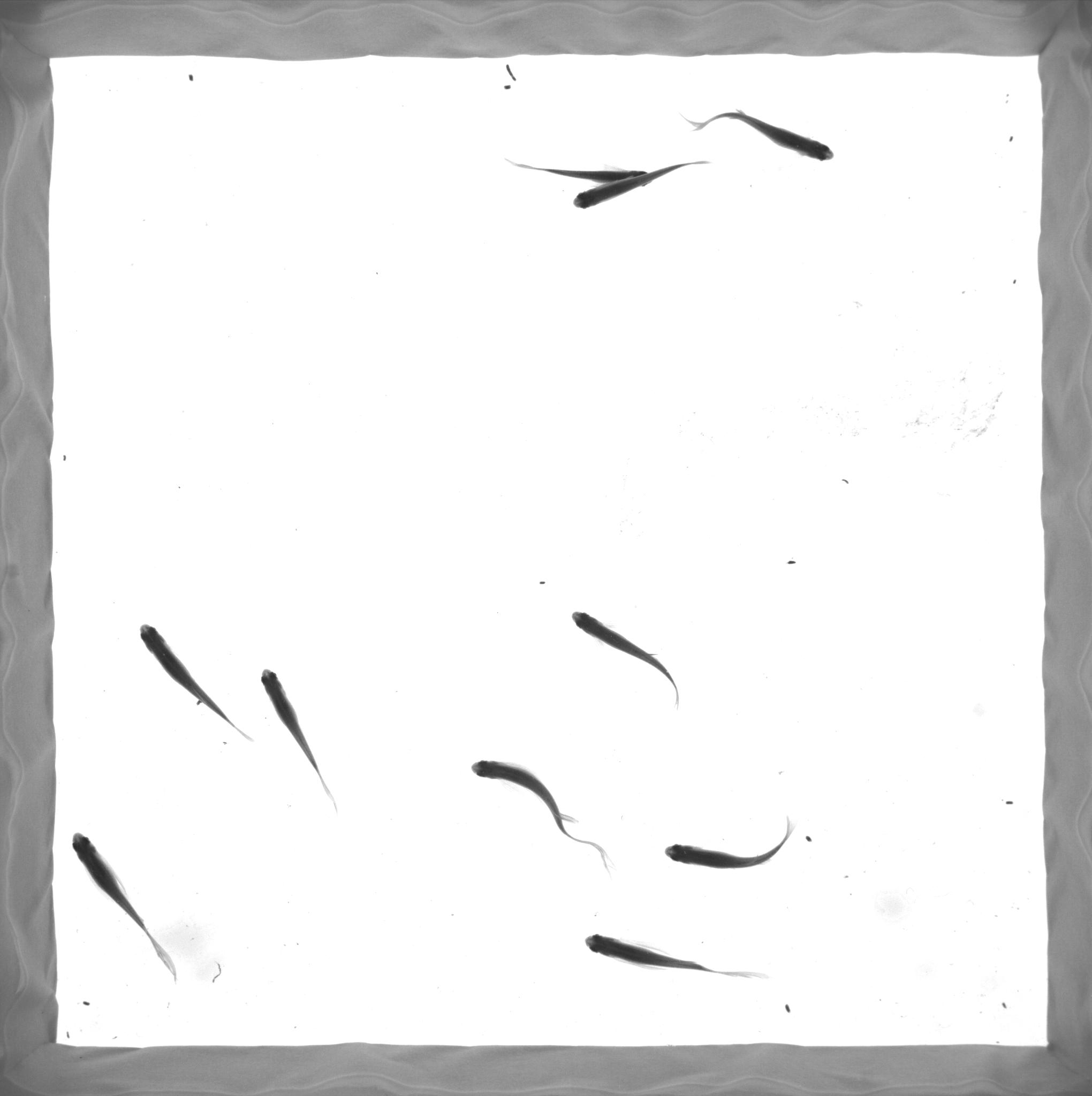

Supplement: S1 File — Source code of the proposed tracking system. (ZIP) [file pone.0154714.s002.zip › code_final/images/CoreView_275_Master_Camera_00010.jpg]

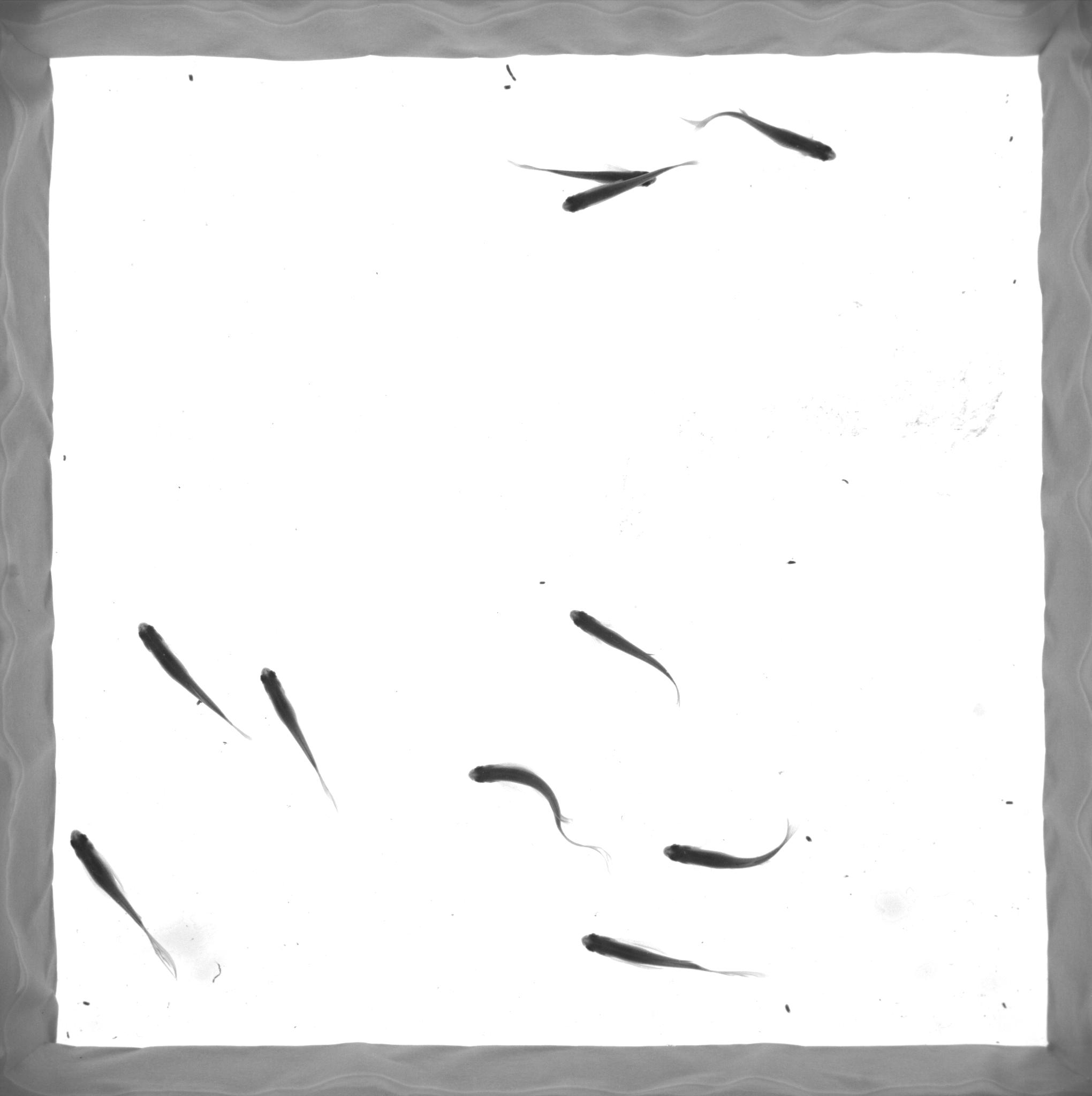

Supplement: S1 File — Source code of the proposed tracking system. (ZIP) [file pone.0154714.s002.zip › code_final/images/CoreView_275_Master_Camera_00011.jpg]

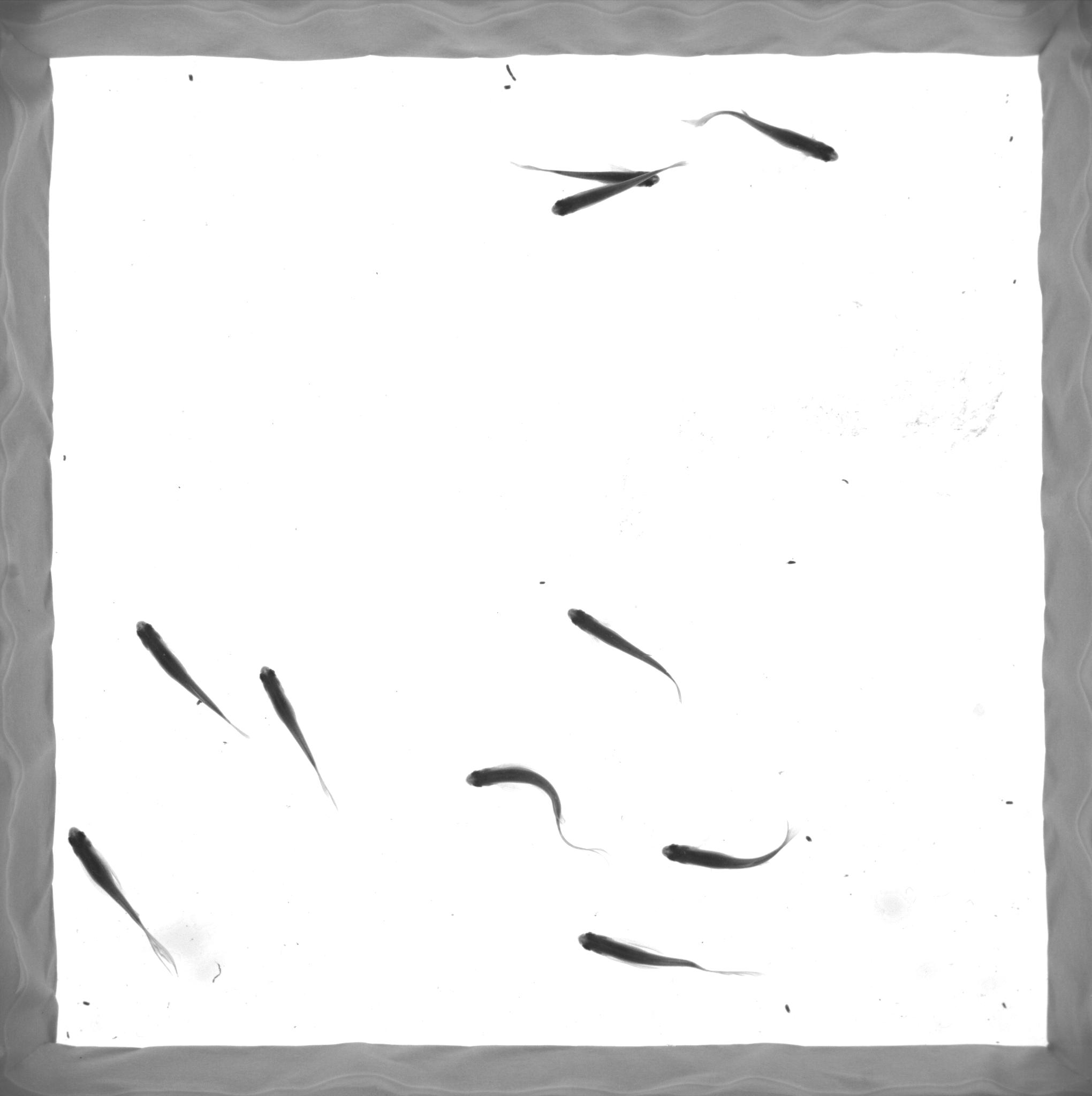

Supplement: S1 File — Source code of the proposed tracking system. (ZIP) [file pone.0154714.s002.zip › code_final/images/CoreView_275_Master_Camera_00012.jpg]

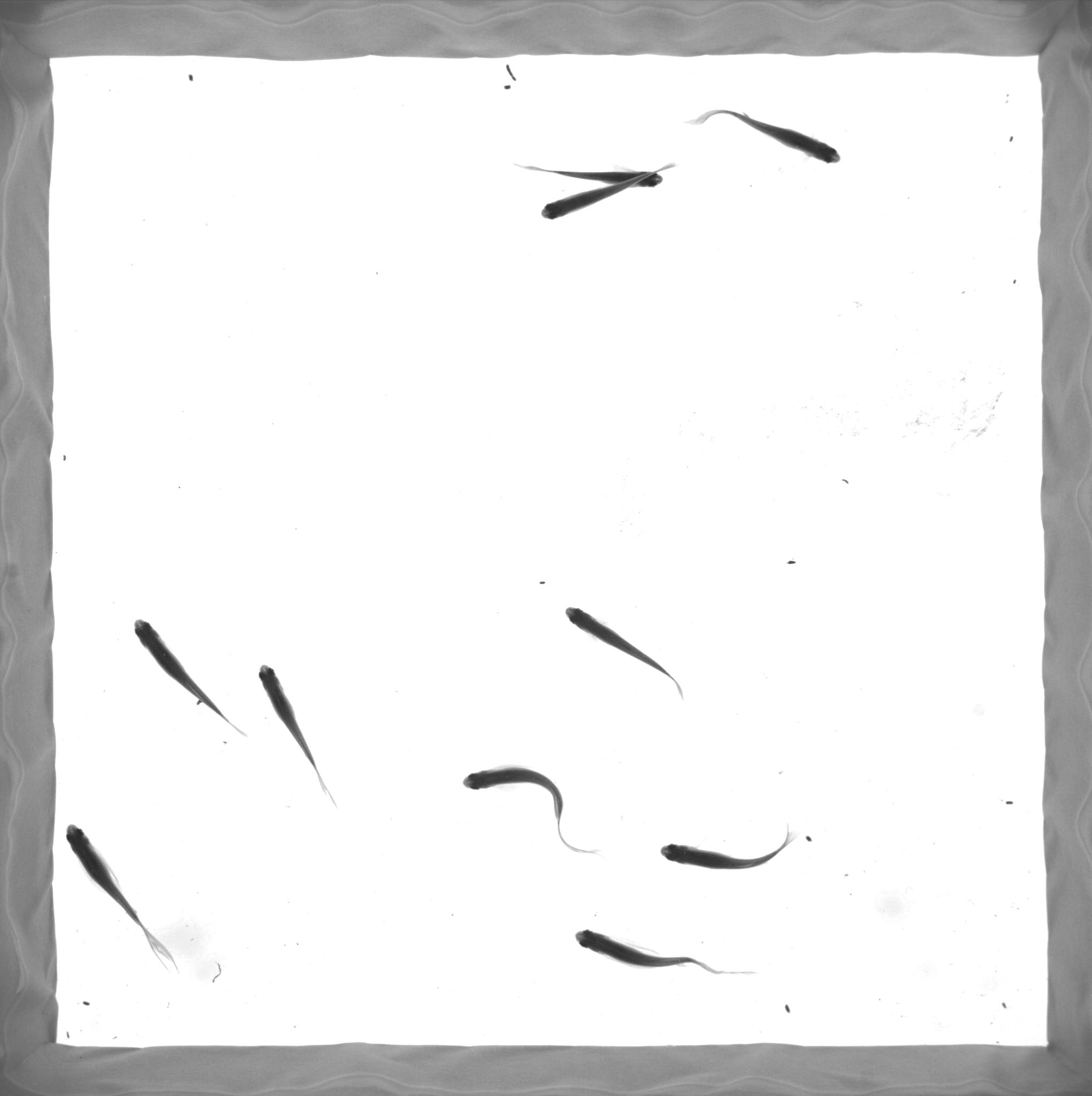

Supplement: S1 File — Source code of the proposed tracking system. (ZIP) [file pone.0154714.s002.zip › code_final/images/CoreView_275_Master_Camera_00013.jpg]

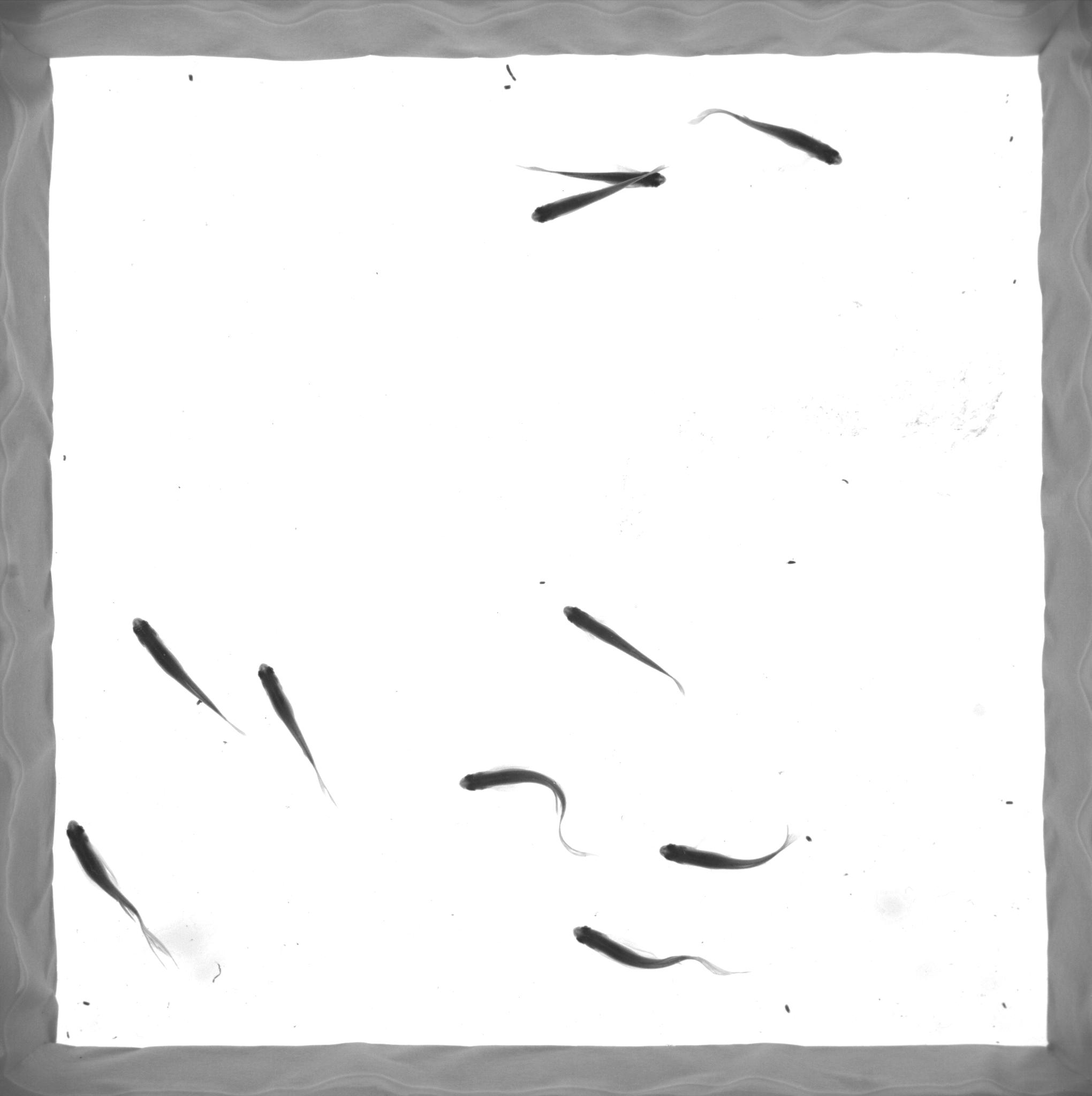

Supplement: S1 File — Source code of the proposed tracking system. (ZIP) [file pone.0154714.s002.zip › code_final/images/CoreView_275_Master_Camera_00014.jpg]

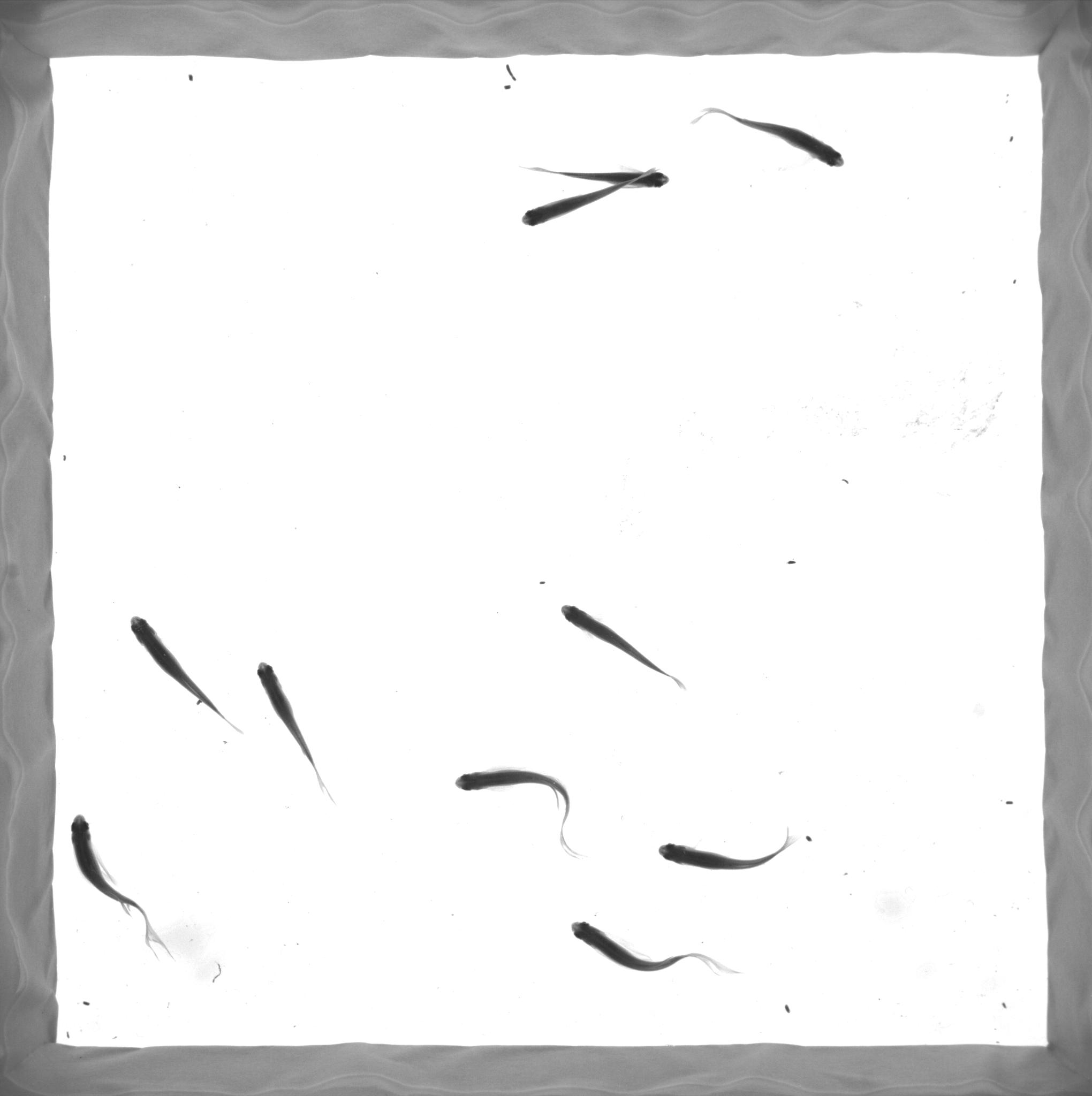

Supplement: S1 File — Source code of the proposed tracking system. (ZIP) [file pone.0154714.s002.zip › code_final/images/CoreView_275_Master_Camera_00015.jpg]

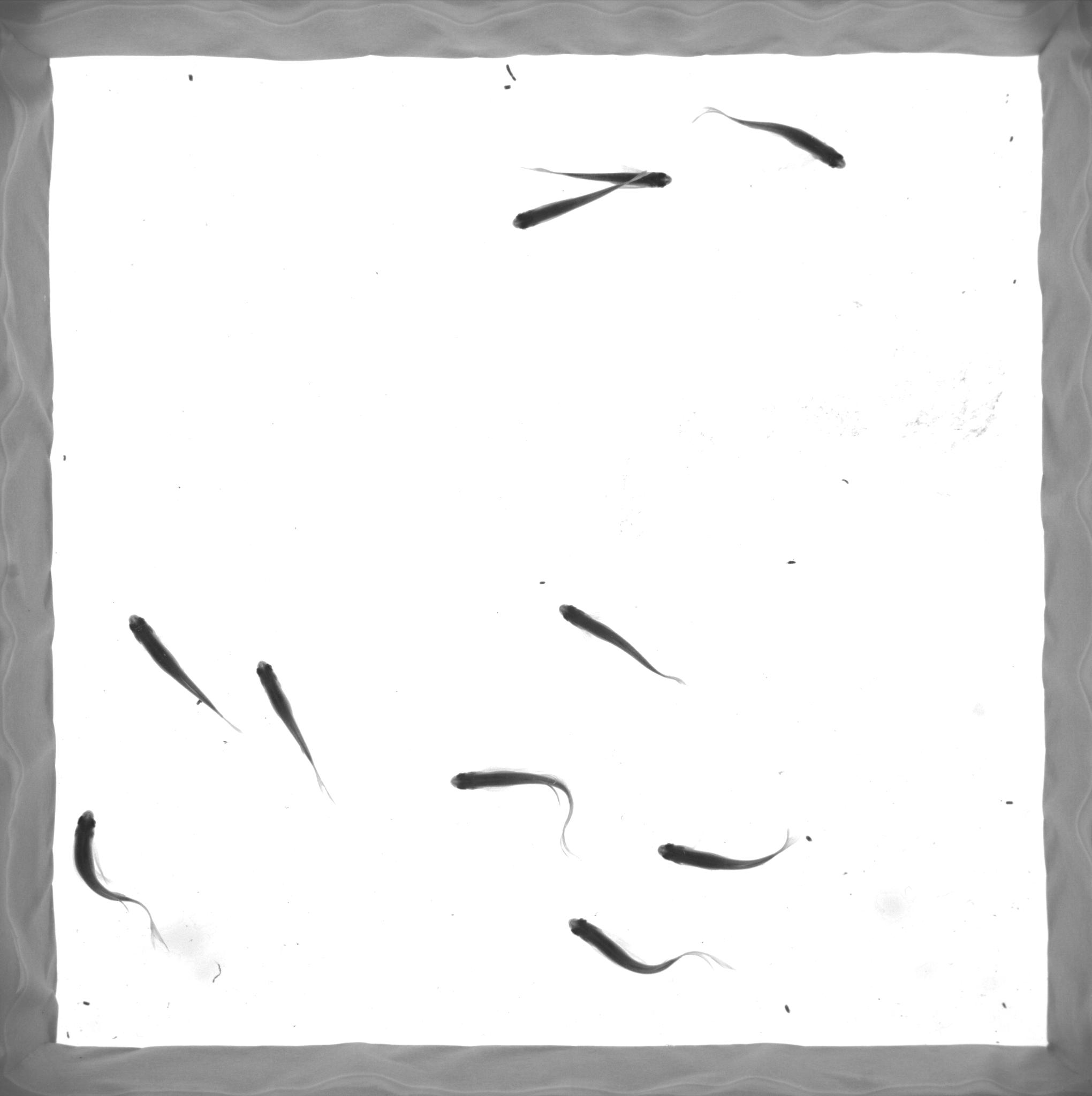

Supplement: S1 File — Source code of the proposed tracking system. (ZIP) [file pone.0154714.s002.zip › code_final/images/CoreView_275_Master_Camera_00016.jpg]

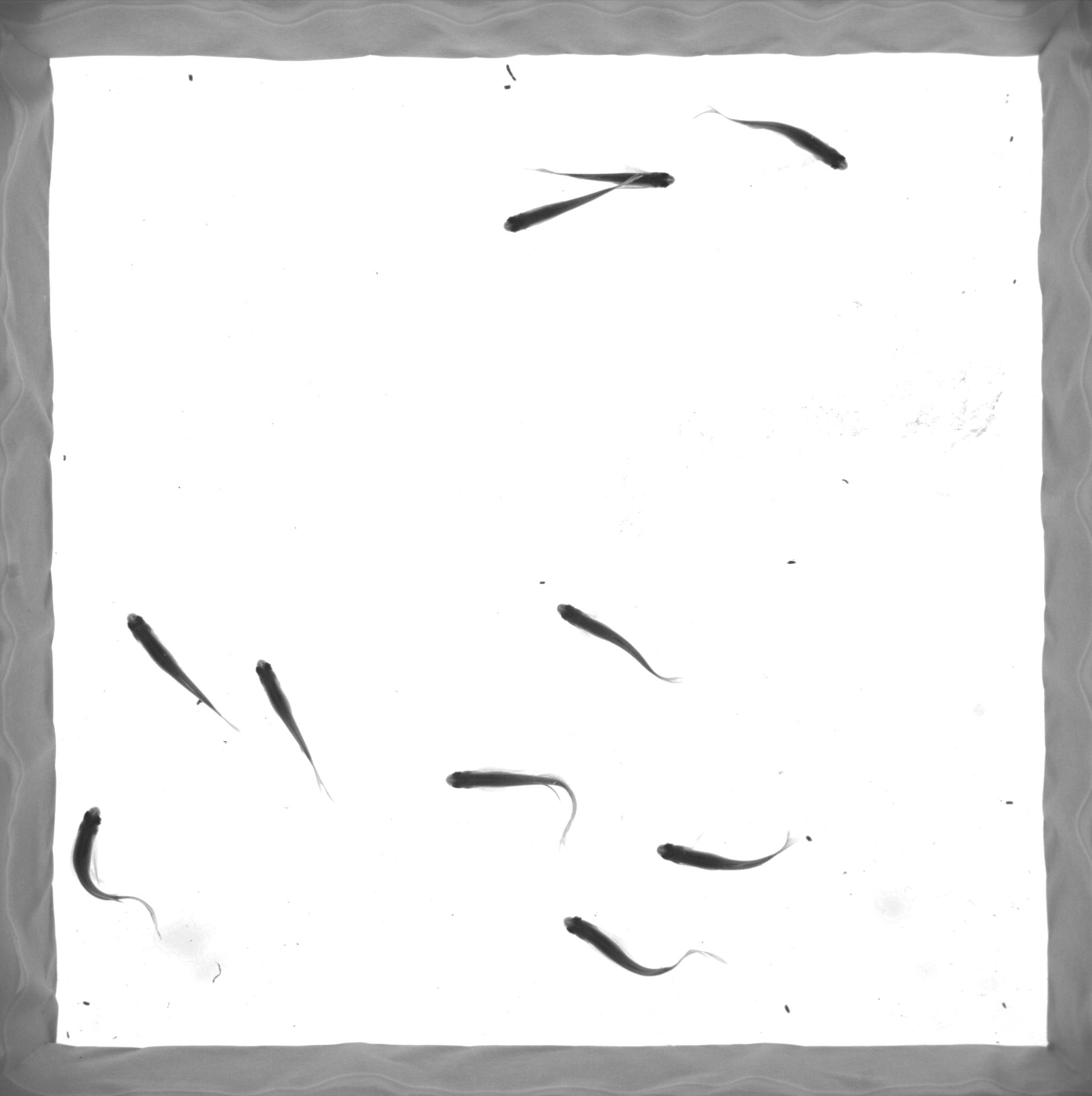

Supplement: S1 File — Source code of the proposed tracking system. (ZIP) [file pone.0154714.s002.zip › code_final/images/CoreView_275_Master_Camera_00017.jpg]

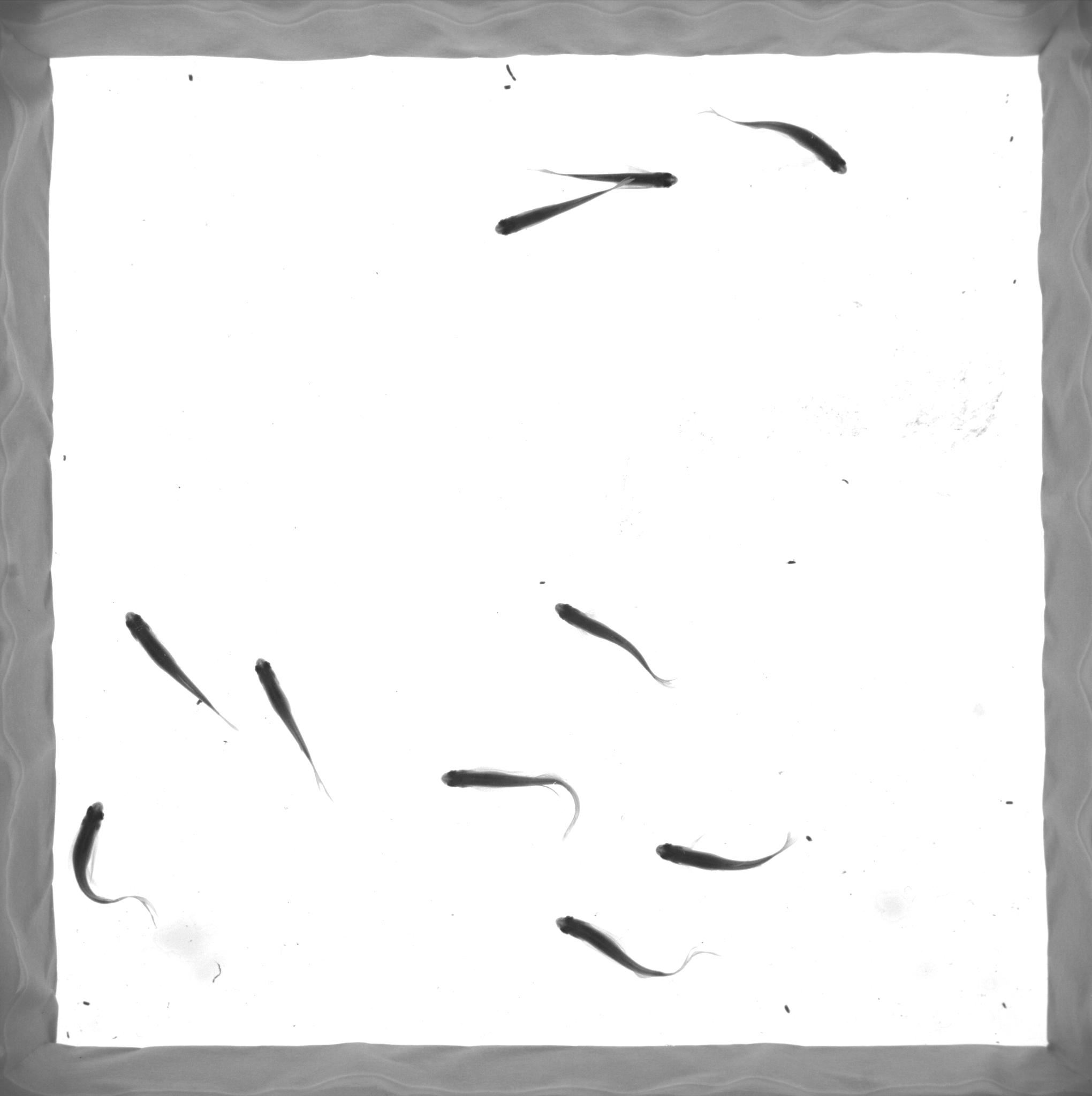

Supplement: S1 File — Source code of the proposed tracking system. (ZIP) [file pone.0154714.s002.zip › code_final/images/CoreView_275_Master_Camera_00018.jpg]

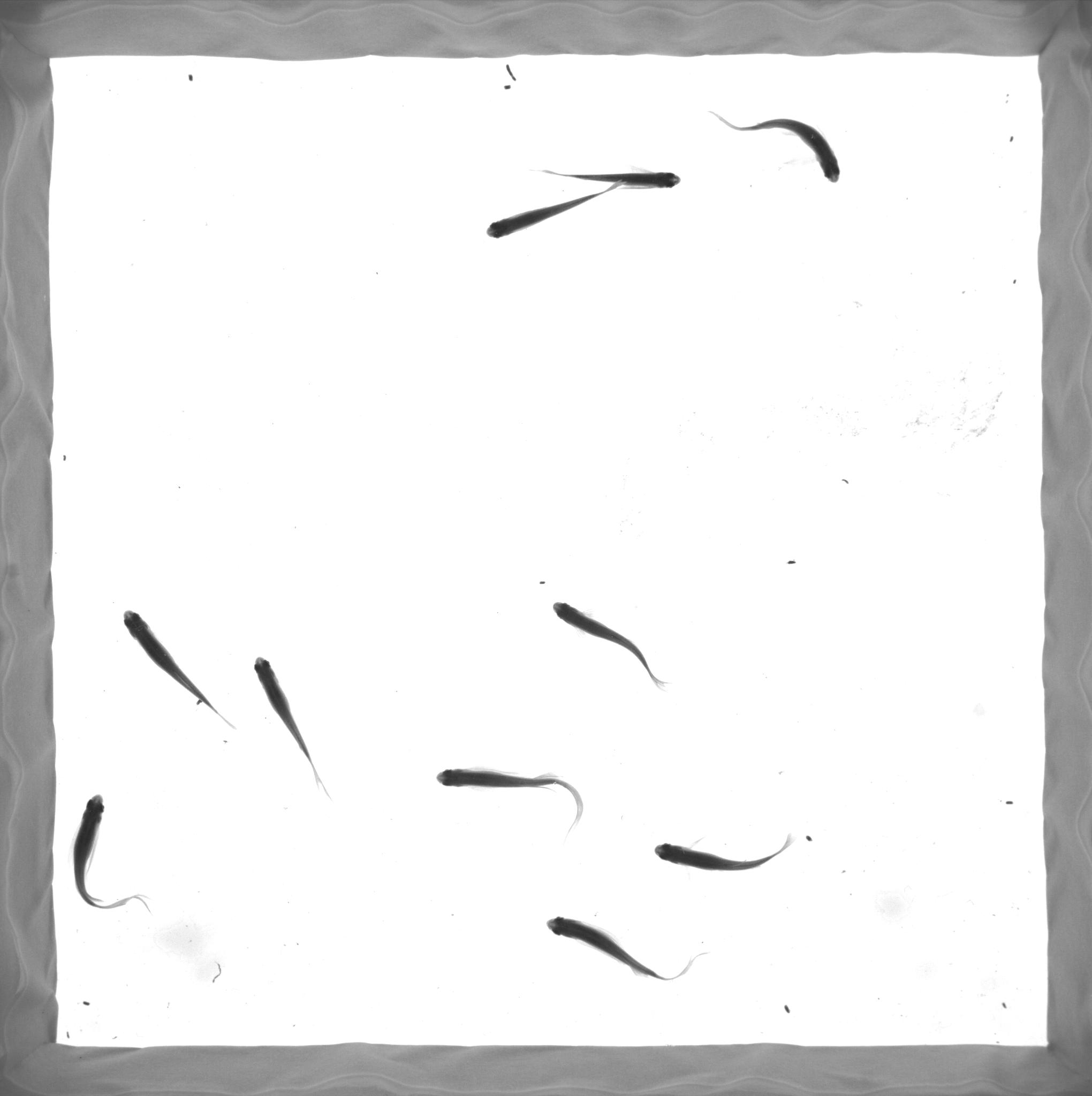

Supplement: S1 File — Source code of the proposed tracking system. (ZIP) [file pone.0154714.s002.zip › code_final/images/CoreView_275_Master_Camera_00019.jpg]

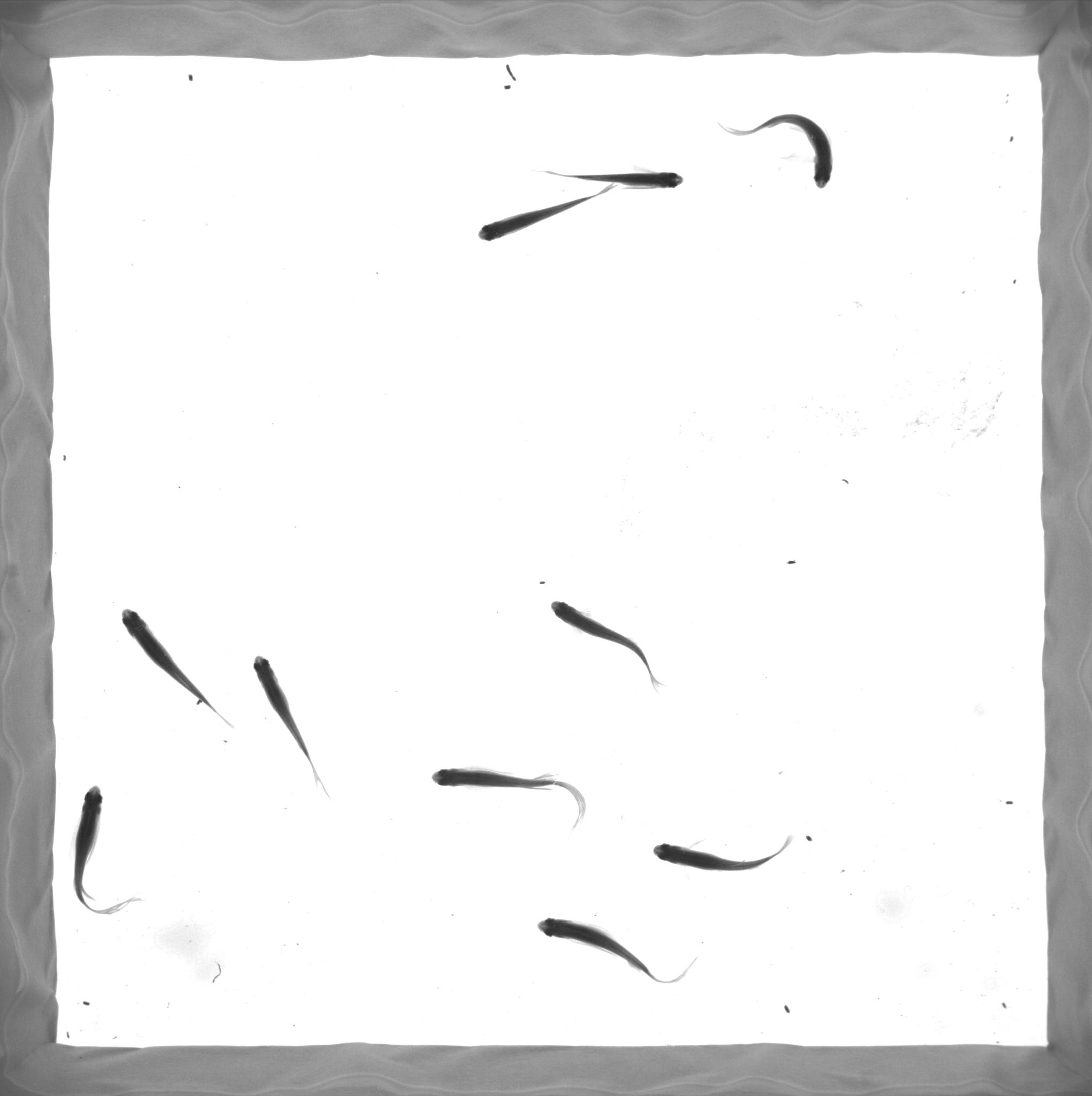

Supplement: S1 File — Source code of the proposed tracking system. (ZIP) [file pone.0154714.s002.zip › code_final/images/CoreView_275_Master_Camera_00020.jpg]

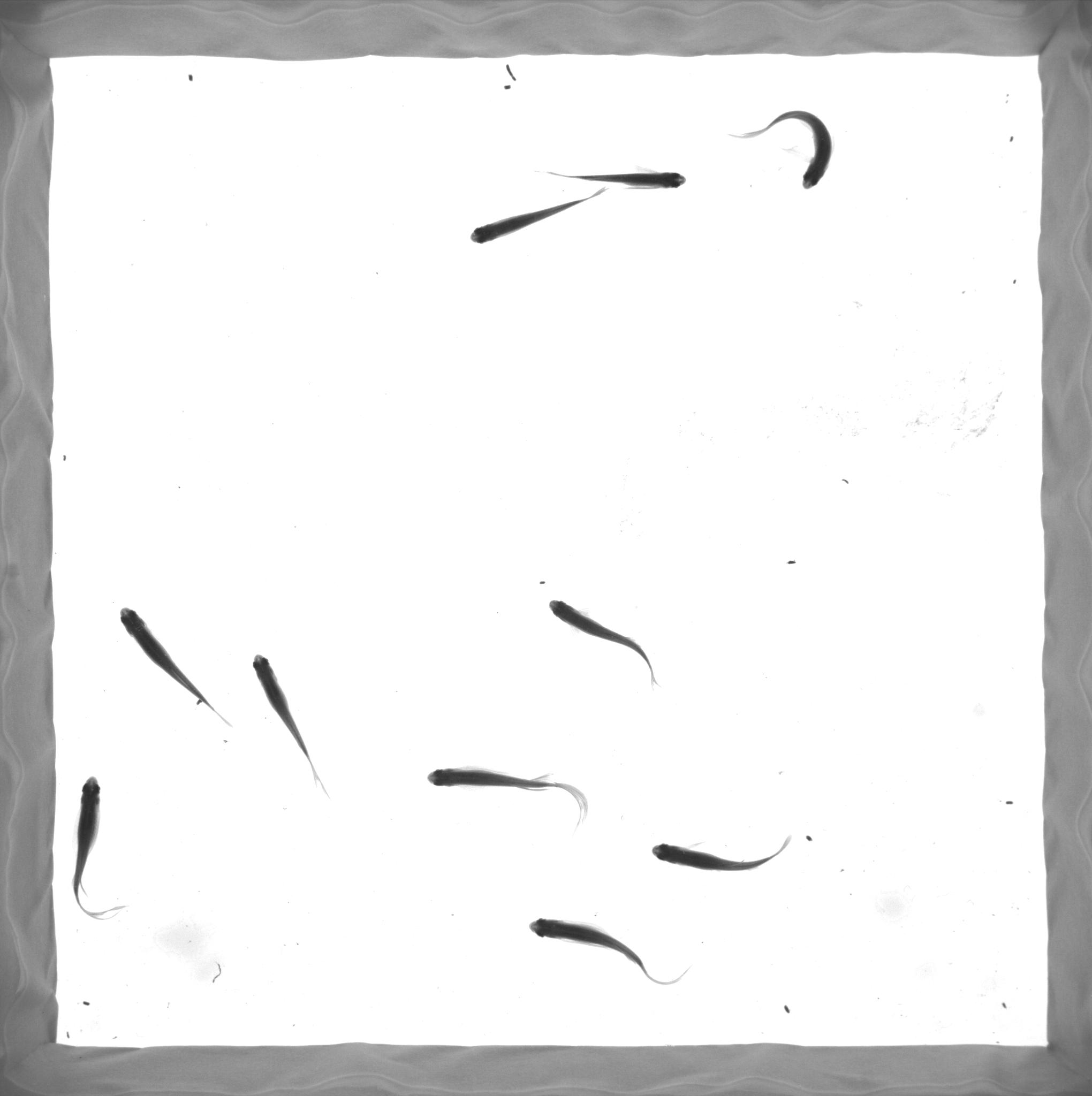

Supplement: S1 File — Source code of the proposed tracking system. (ZIP) [file pone.0154714.s002.zip › code_final/images/CoreView_275_Master_Camera_00021.jpg]

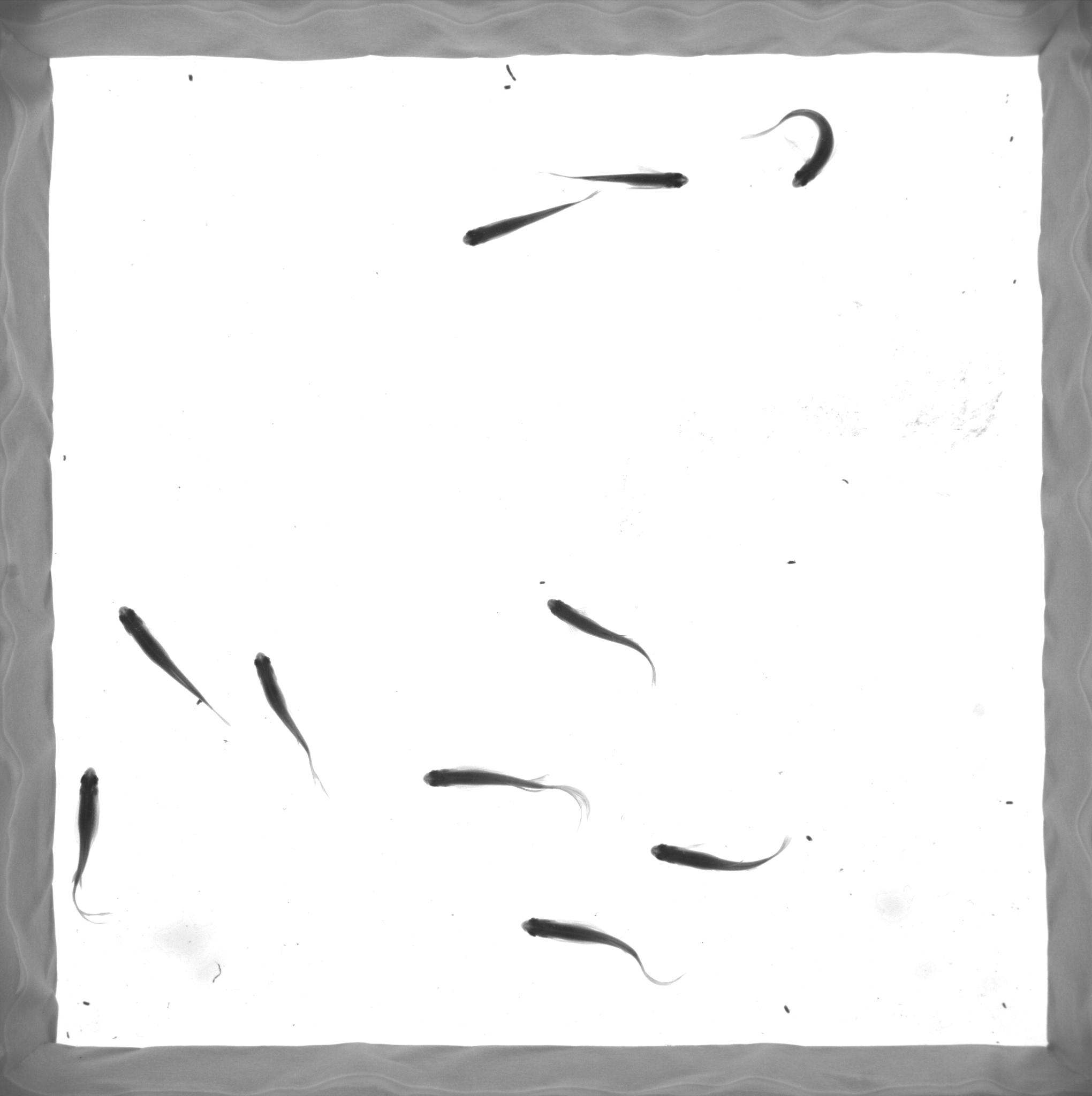

Supplement: S1 File — Source code of the proposed tracking system. (ZIP) [file pone.0154714.s002.zip › code_final/images/CoreView_275_Master_Camera_00022.jpg]

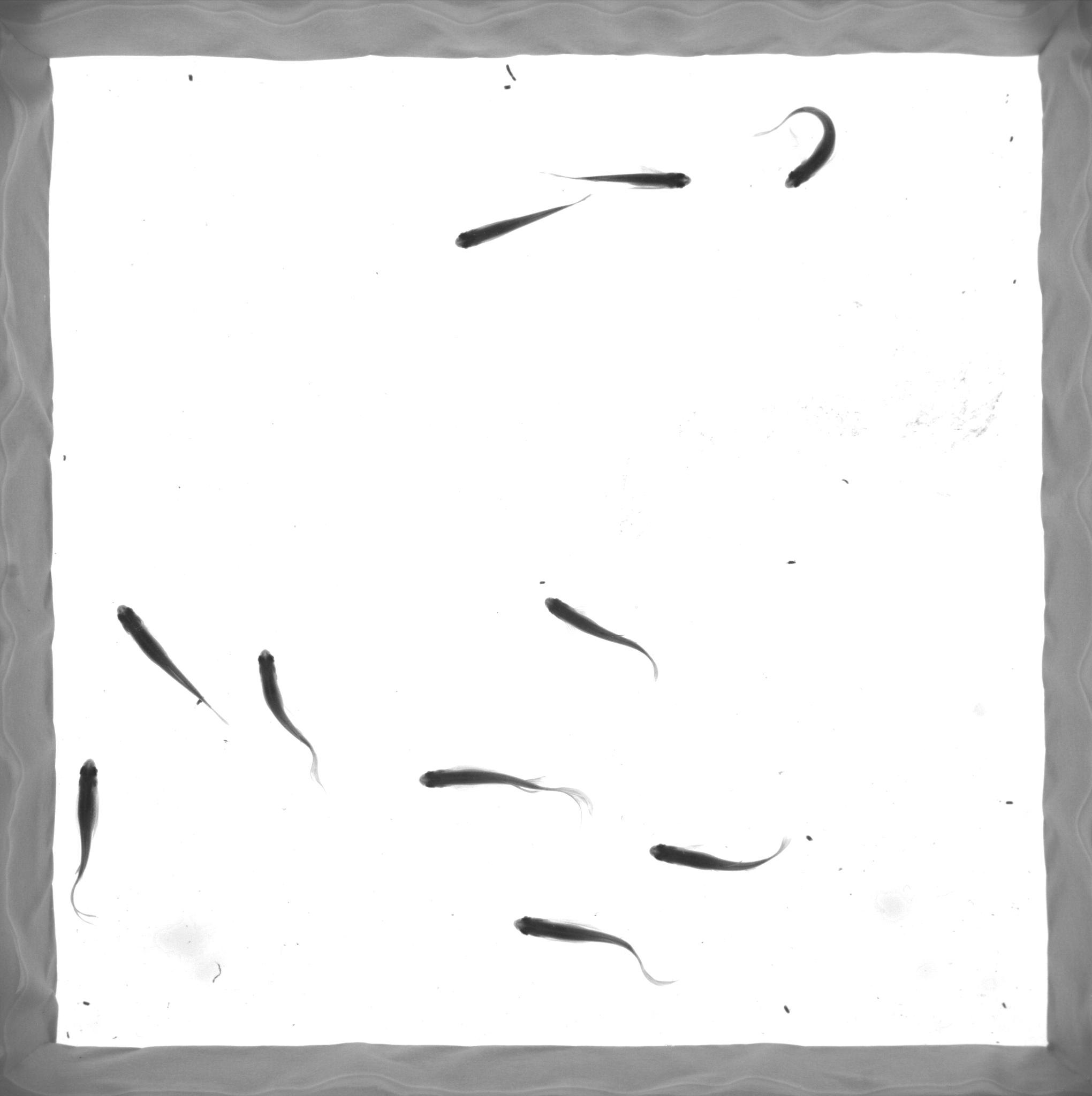

Supplement: S1 File — Source code of the proposed tracking system. (ZIP) [file pone.0154714.s002.zip › code_final/images/CoreView_275_Master_Camera_00023.jpg]

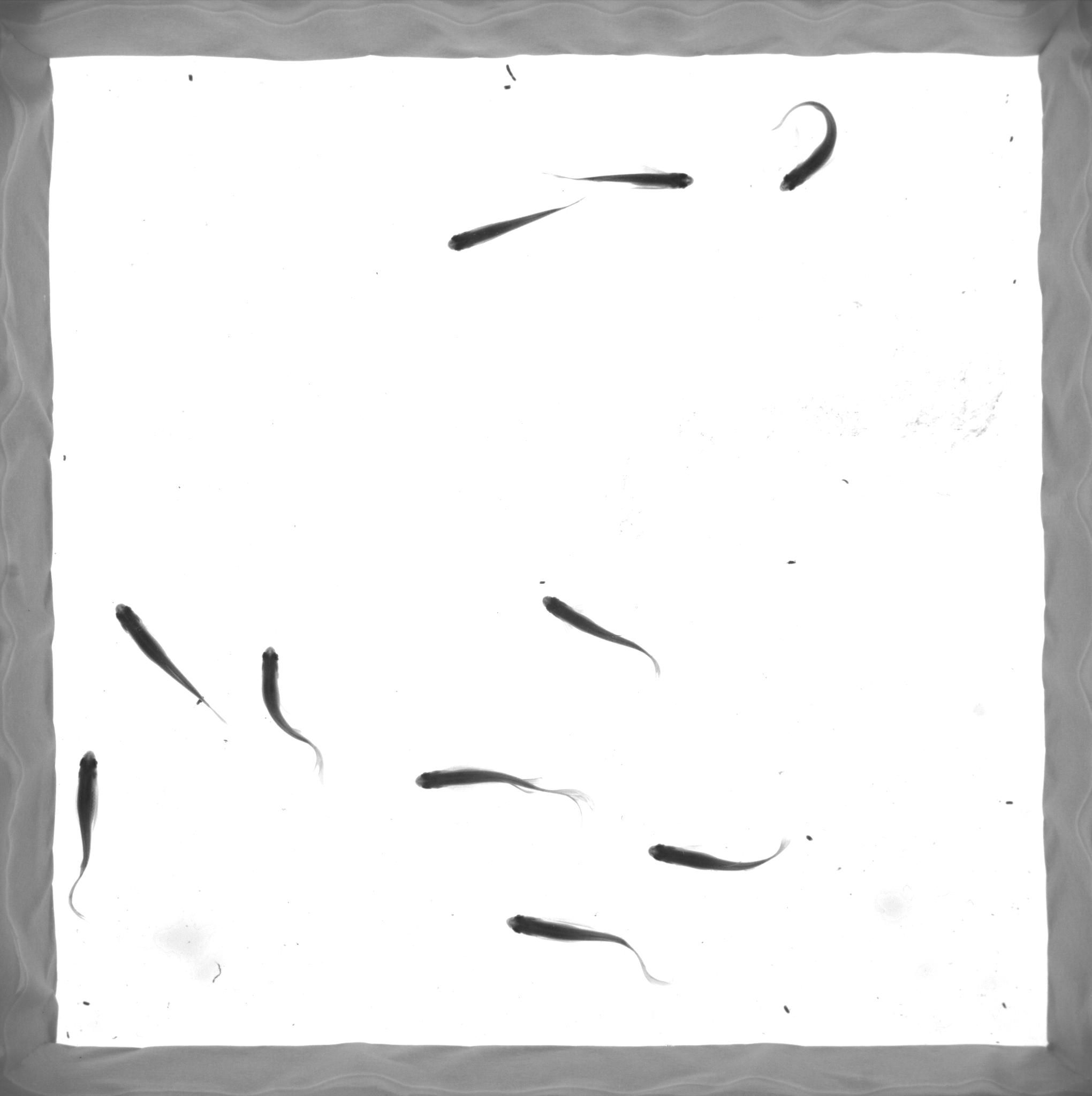

Supplement: S1 File — Source code of the proposed tracking system. (ZIP) [file pone.0154714.s002.zip › code_final/images/CoreView_275_Master_Camera_00024.jpg]

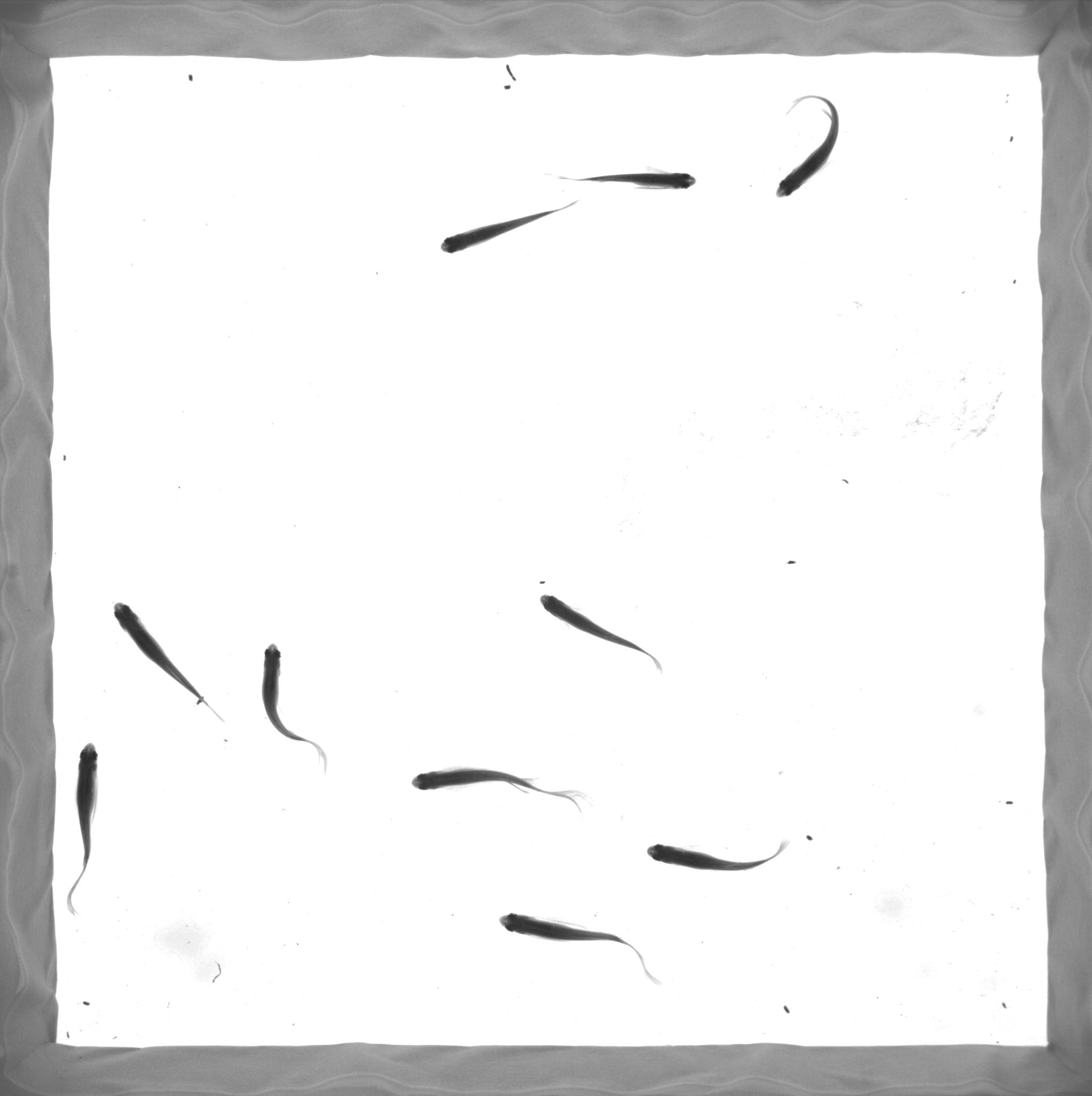

Supplement: S1 File — Source code of the proposed tracking system. (ZIP) [file pone.0154714.s002.zip › code_final/images/CoreView_275_Master_Camera_00025.jpg]

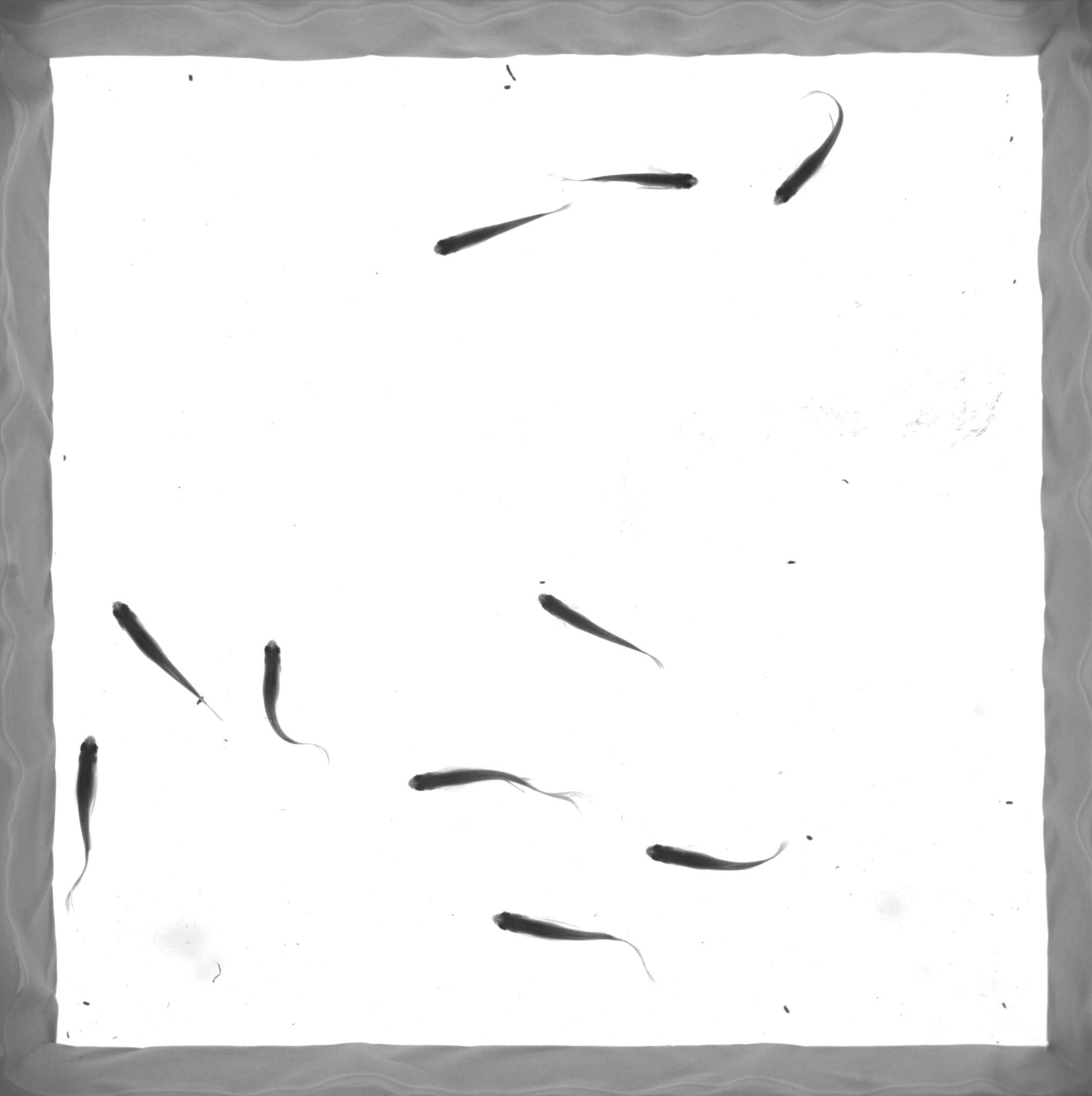

Supplement: S1 File — Source code of the proposed tracking system. (ZIP) [file pone.0154714.s002.zip › code_final/images/CoreView_275_Master_Camera_00026.jpg]

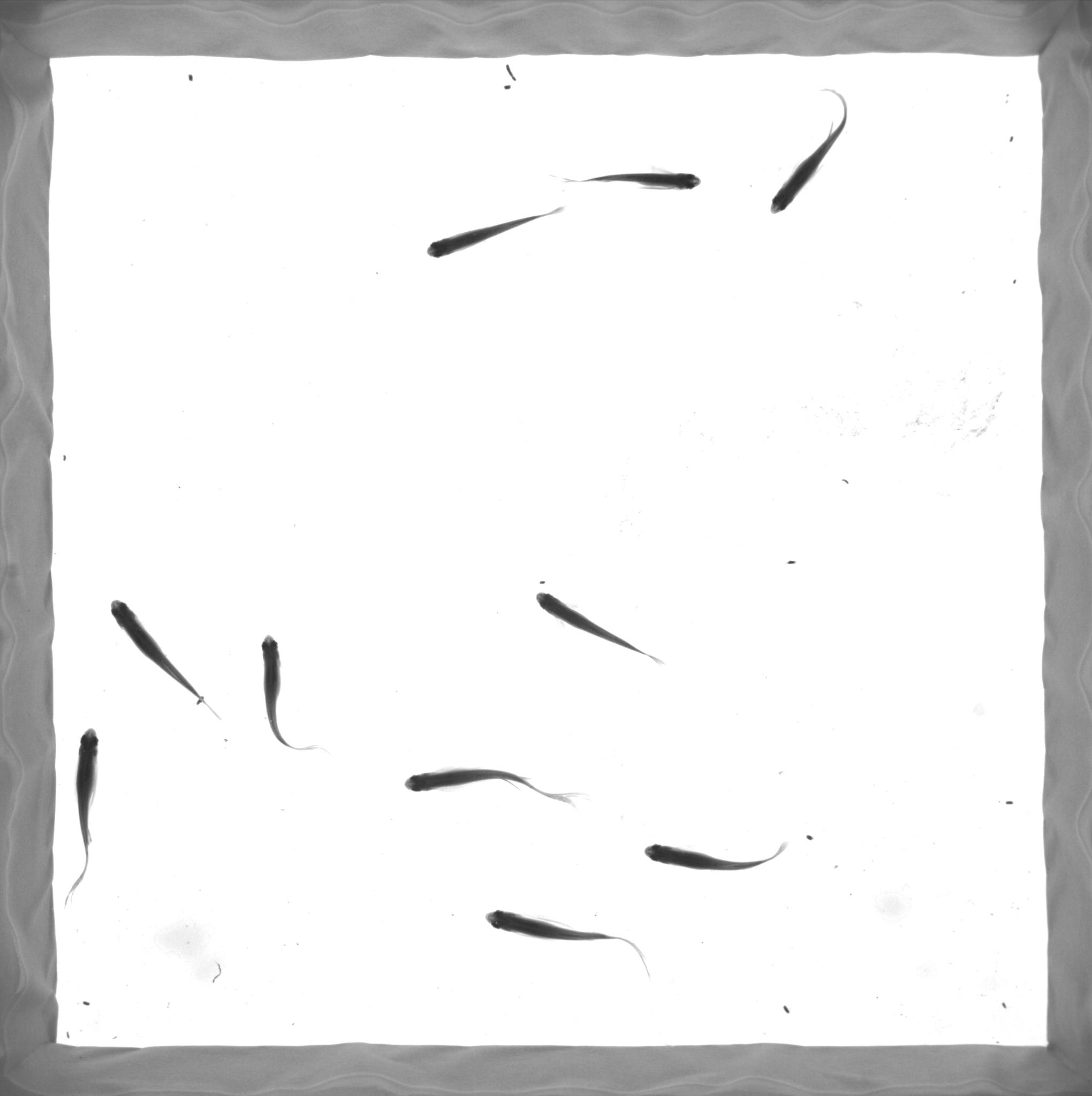

Supplement: S1 File — Source code of the proposed tracking system. (ZIP) [file pone.0154714.s002.zip › code_final/images/CoreView_275_Master_Camera_00027.jpg]

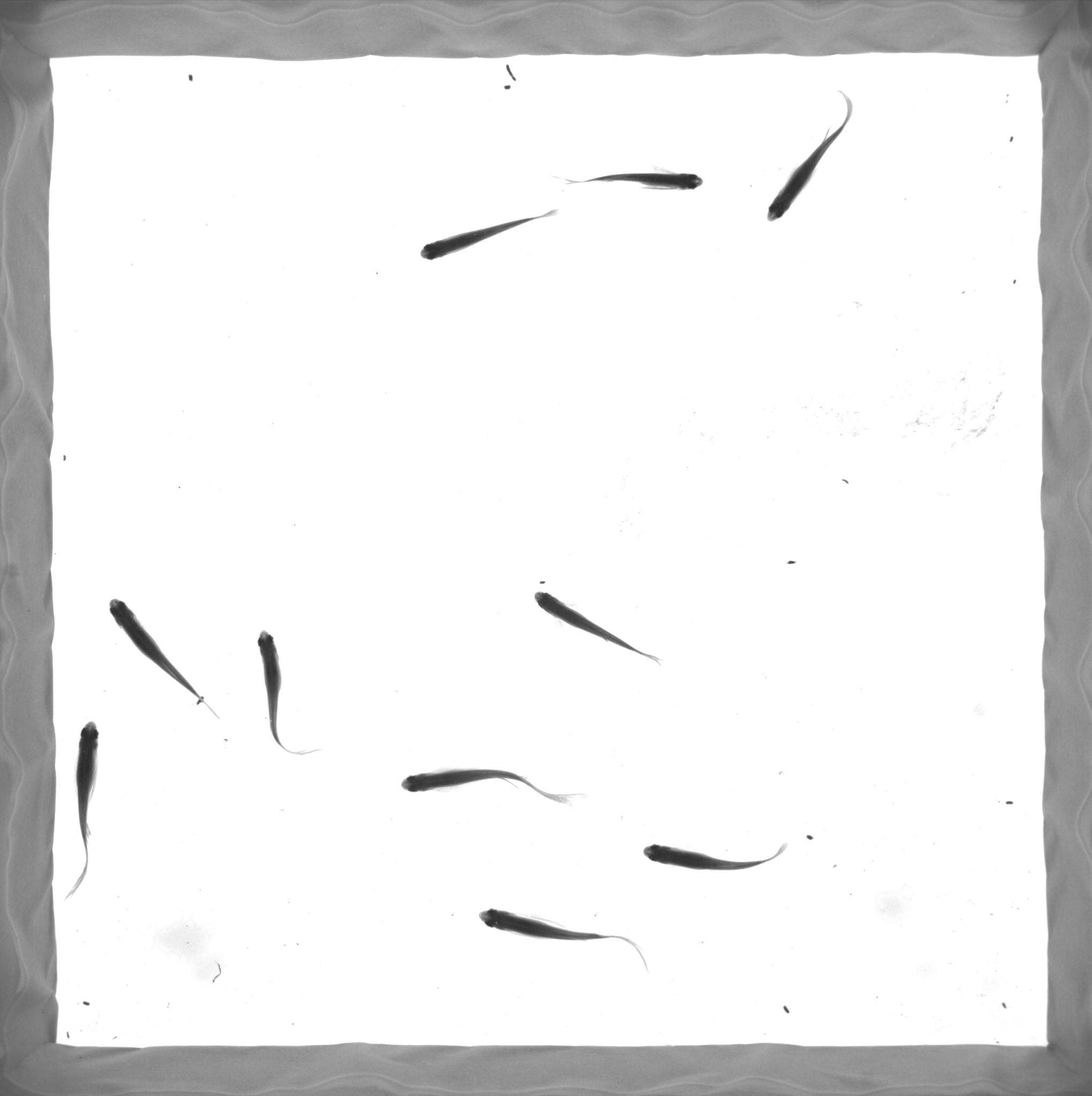

Supplement: S1 File — Source code of the proposed tracking system. (ZIP) [file pone.0154714.s002.zip › code_final/images/CoreView_275_Master_Camera_00028.jpg]

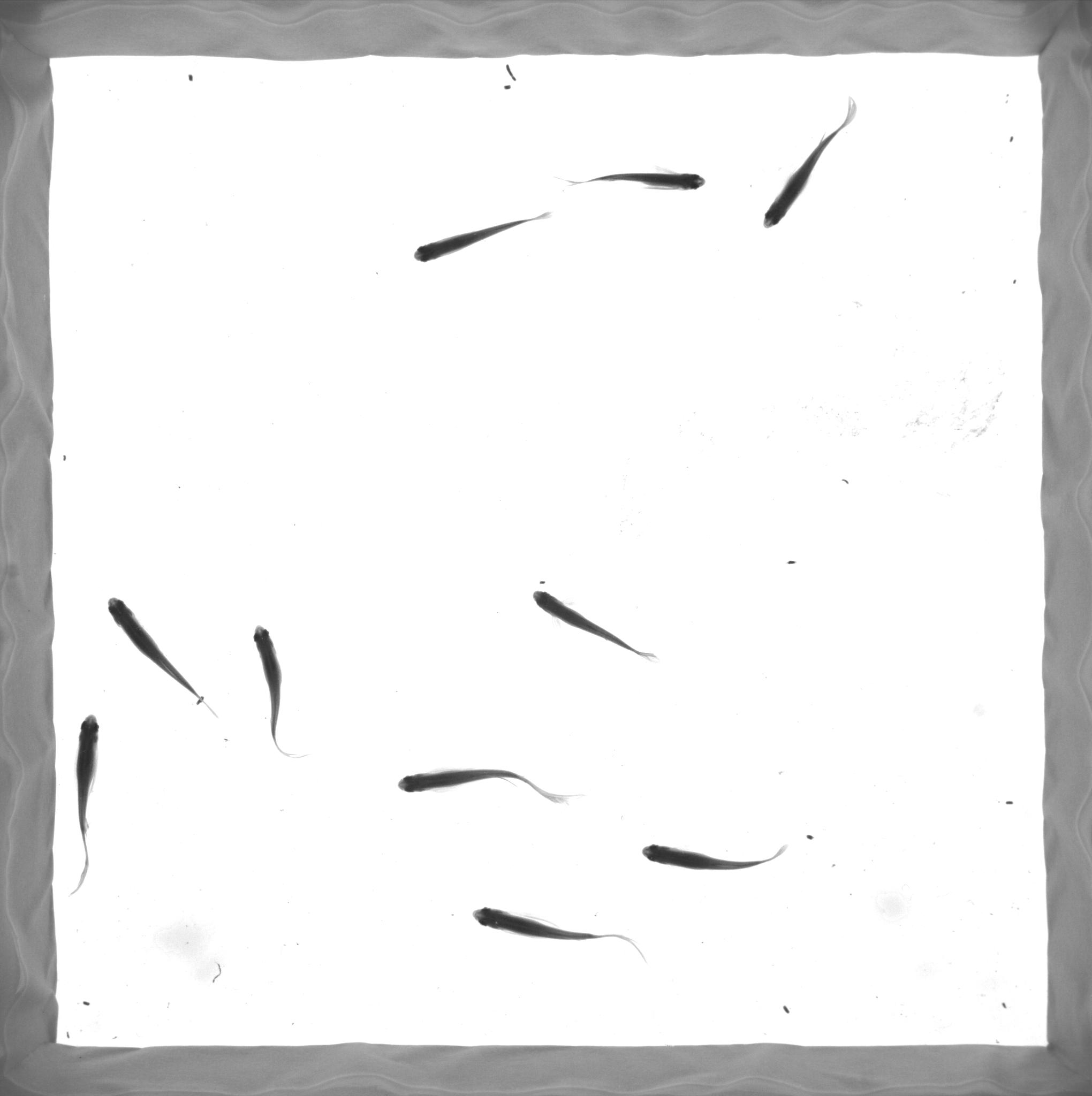

Supplement: S1 File — Source code of the proposed tracking system. (ZIP) [file pone.0154714.s002.zip › code_final/images/CoreView_275_Master_Camera_00029.jpg]

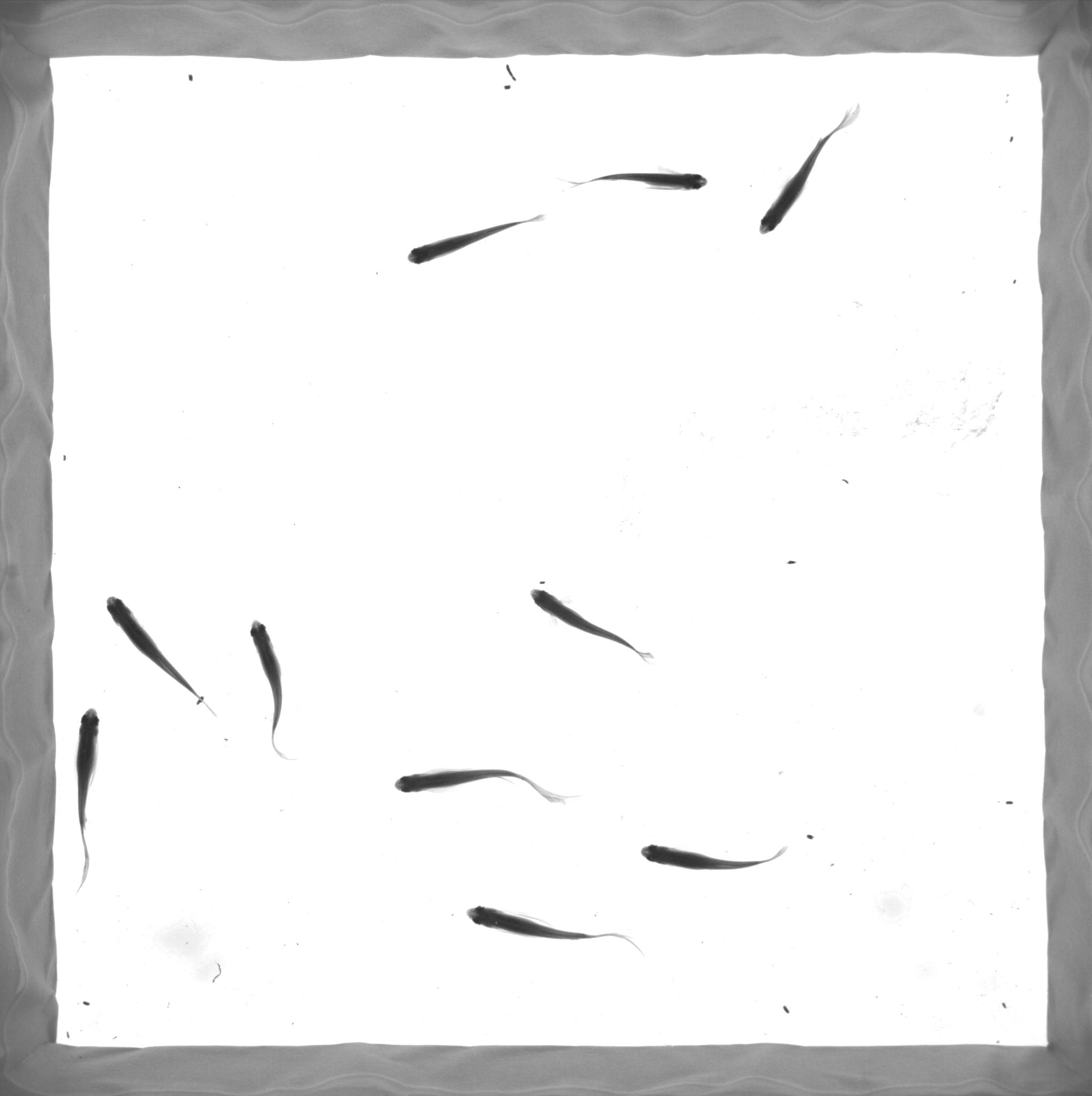

Supplement: S1 File — Source code of the proposed tracking system. (ZIP) [file pone.0154714.s002.zip › code_final/images/CoreView_275_Master_Camera_00030.jpg]

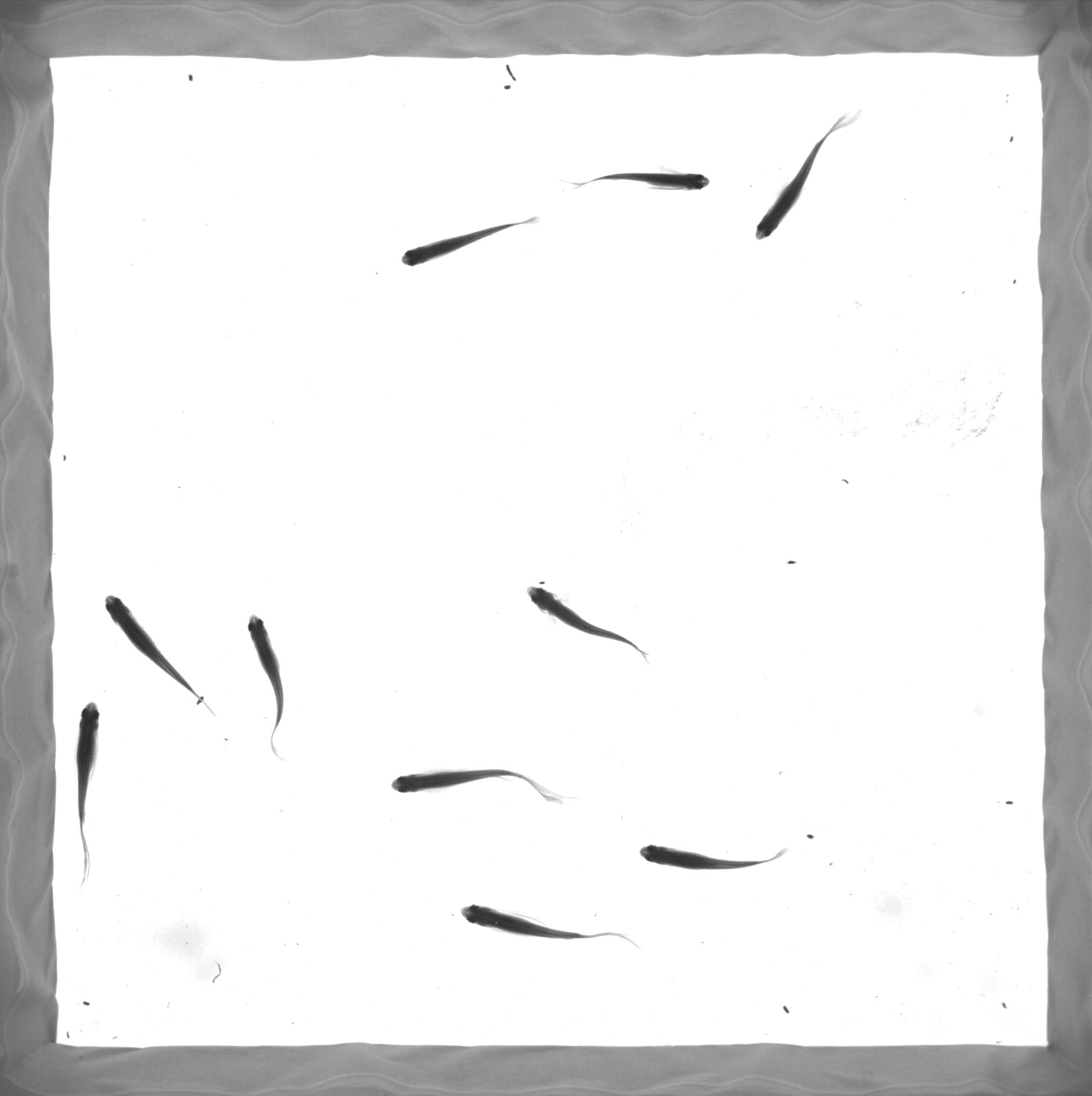

Supplement: S1 File — Source code of the proposed tracking system. (ZIP) [file pone.0154714.s002.zip › code_final/images/CoreView_275_Master_Camera_00031.jpg]

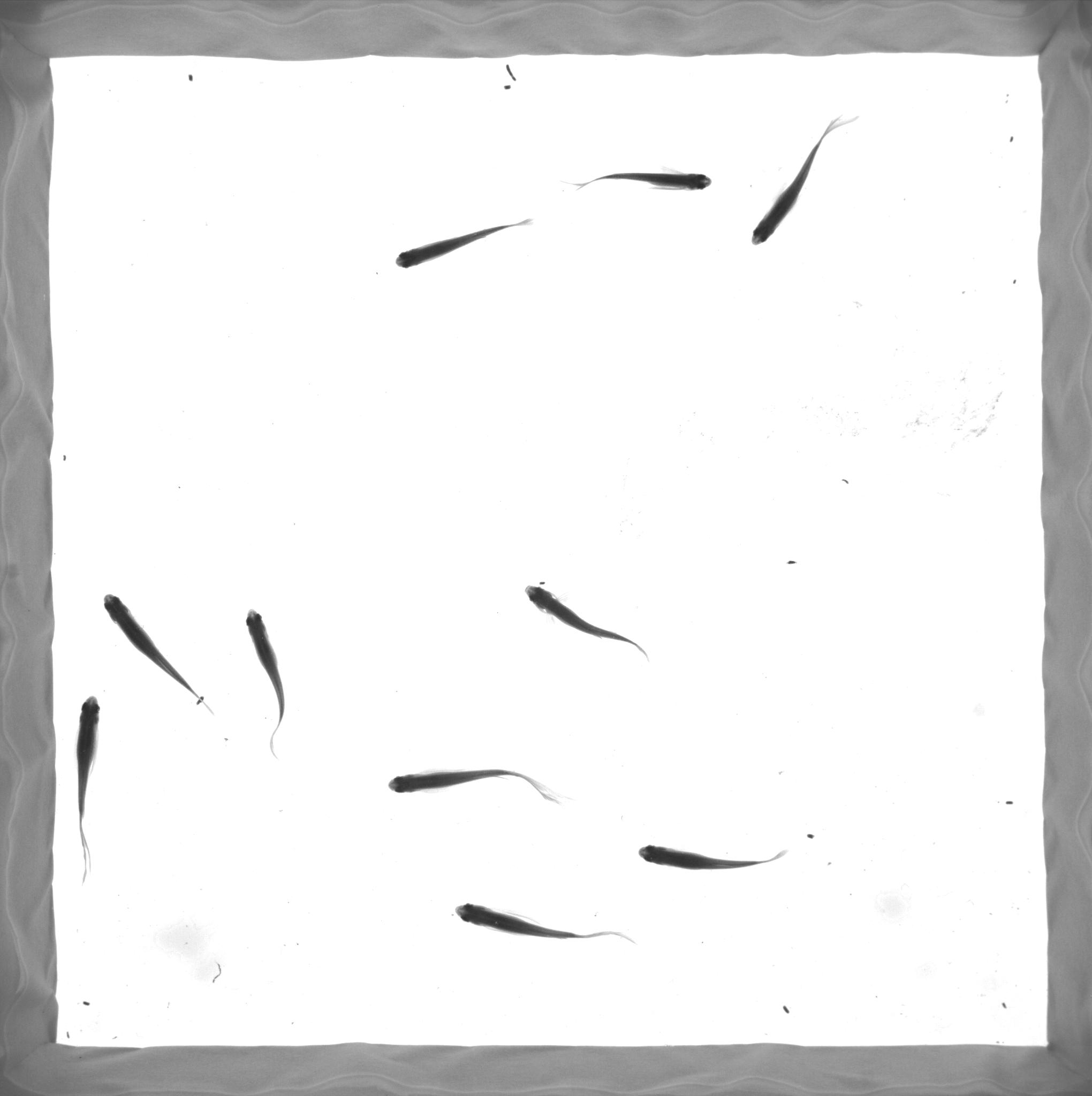

Supplement: S1 File — Source code of the proposed tracking system. (ZIP) [file pone.0154714.s002.zip › code_final/images/CoreView_275_Master_Camera_00032.jpg]

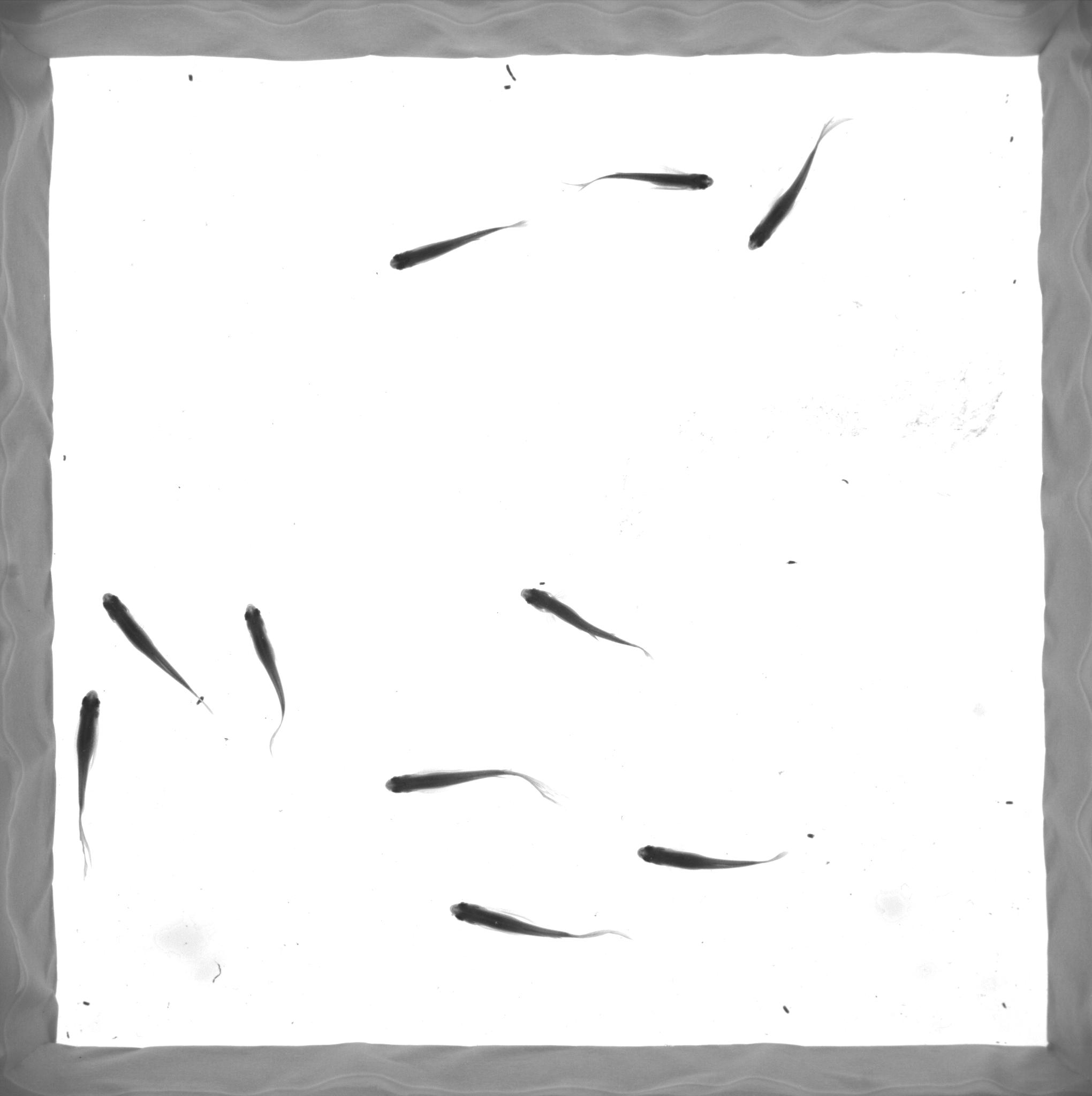

Supplement: S1 File — Source code of the proposed tracking system. (ZIP) [file pone.0154714.s002.zip › code_final/images/CoreView_275_Master_Camera_00033.jpg]

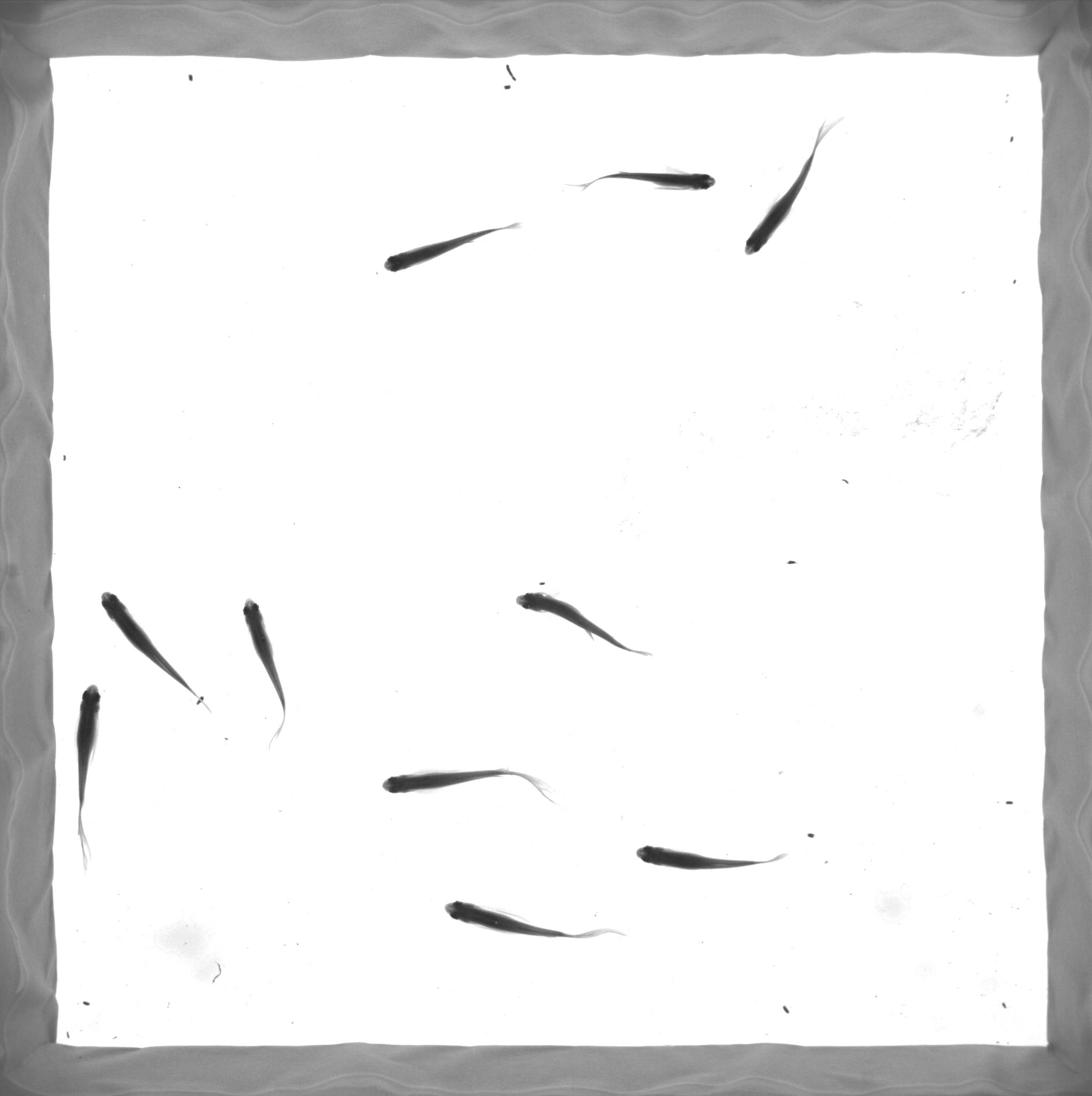

Supplement: S1 File — Source code of the proposed tracking system. (ZIP) [file pone.0154714.s002.zip › code_final/images/CoreView_275_Master_Camera_00034.jpg]

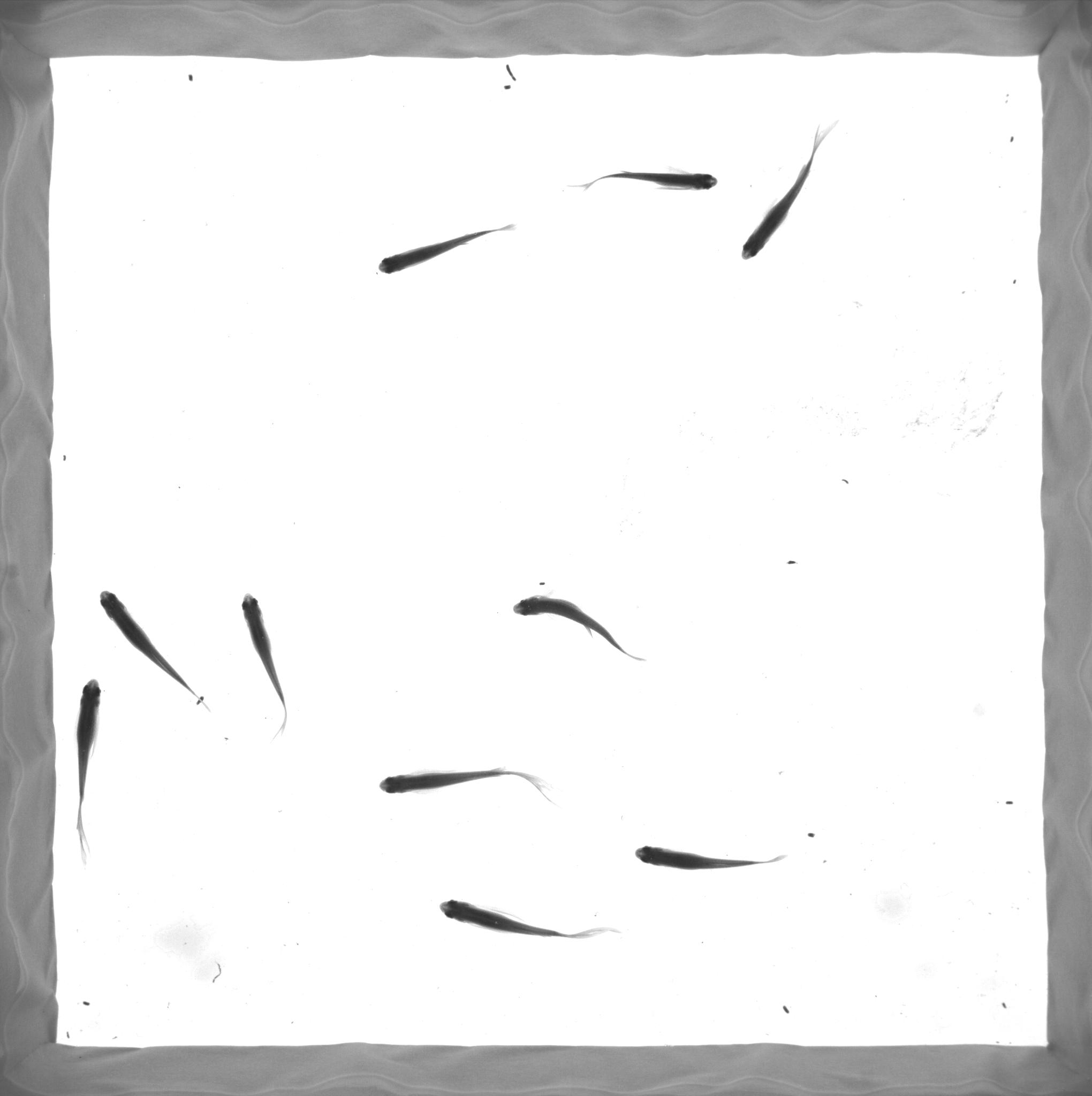

Supplement: S1 File — Source code of the proposed tracking system. (ZIP) [file pone.0154714.s002.zip › code_final/images/CoreView_275_Master_Camera_00035.jpg]

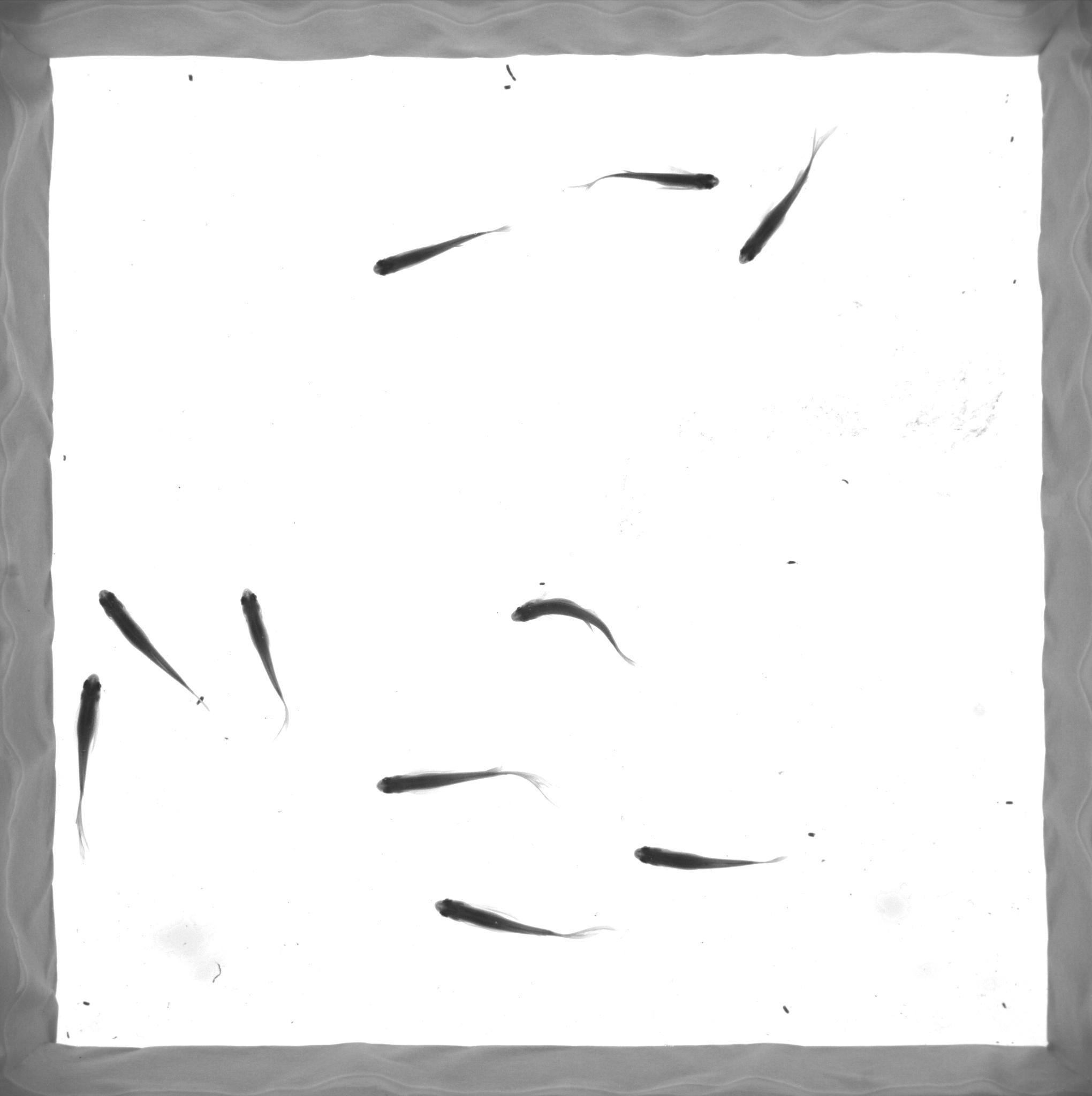

Supplement: S1 File — Source code of the proposed tracking system. (ZIP) [file pone.0154714.s002.zip › code_final/images/CoreView_275_Master_Camera_00036.jpg]

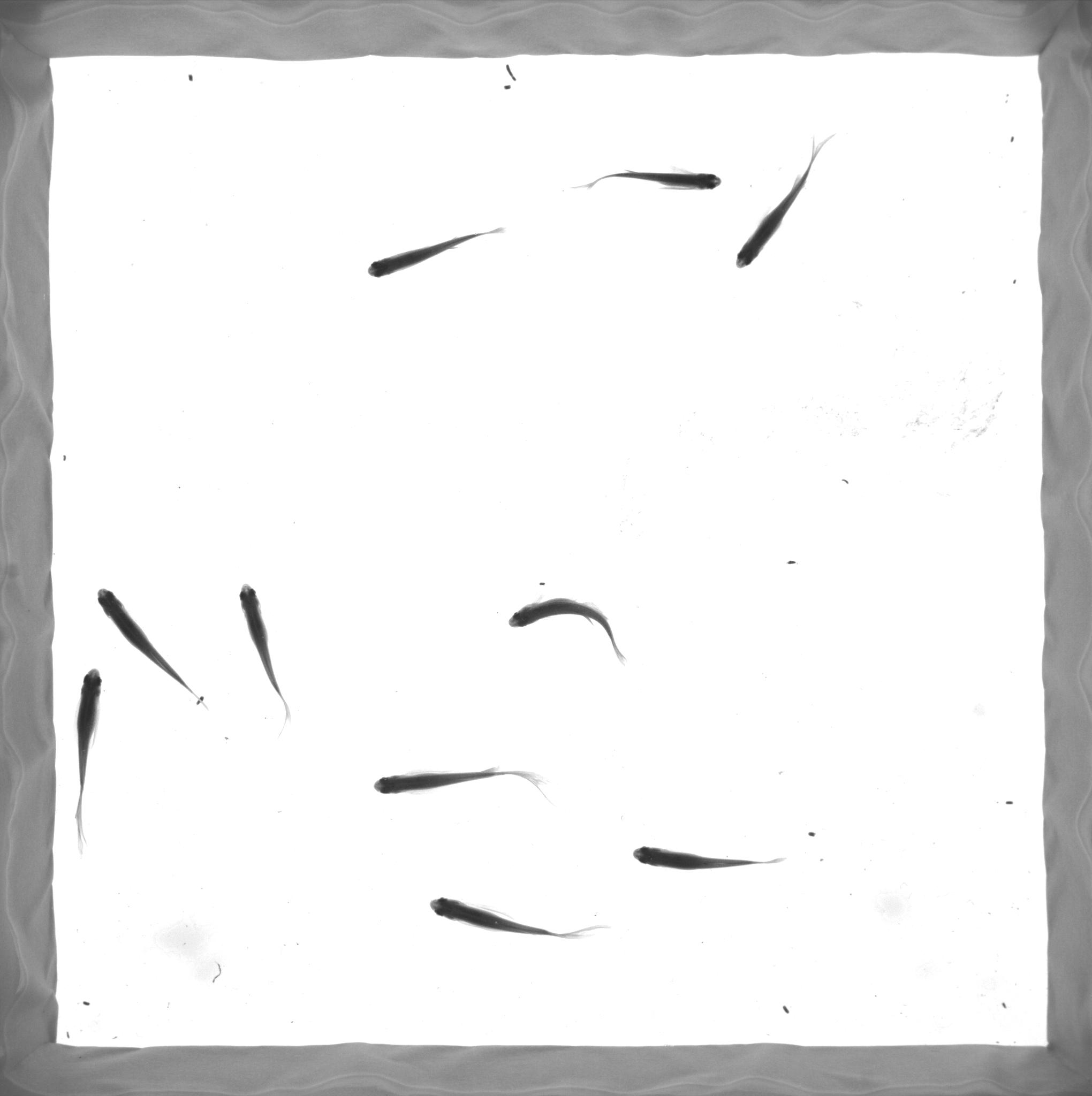

Supplement: S1 File — Source code of the proposed tracking system. (ZIP) [file pone.0154714.s002.zip › code_final/images/CoreView_275_Master_Camera_00037.jpg]

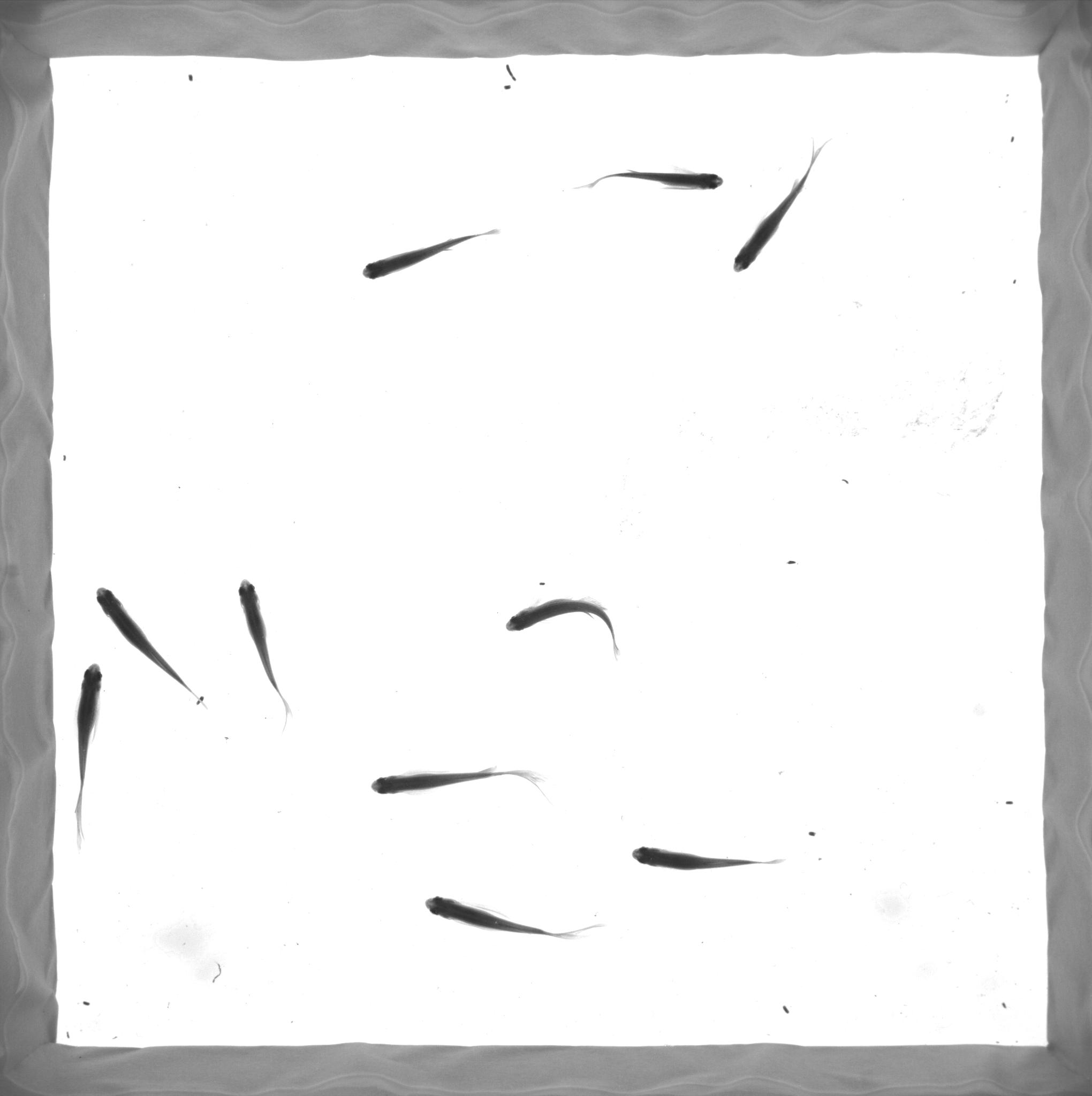

Supplement: S1 File — Source code of the proposed tracking system. (ZIP) [file pone.0154714.s002.zip › code_final/images/CoreView_275_Master_Camera_00038.jpg]

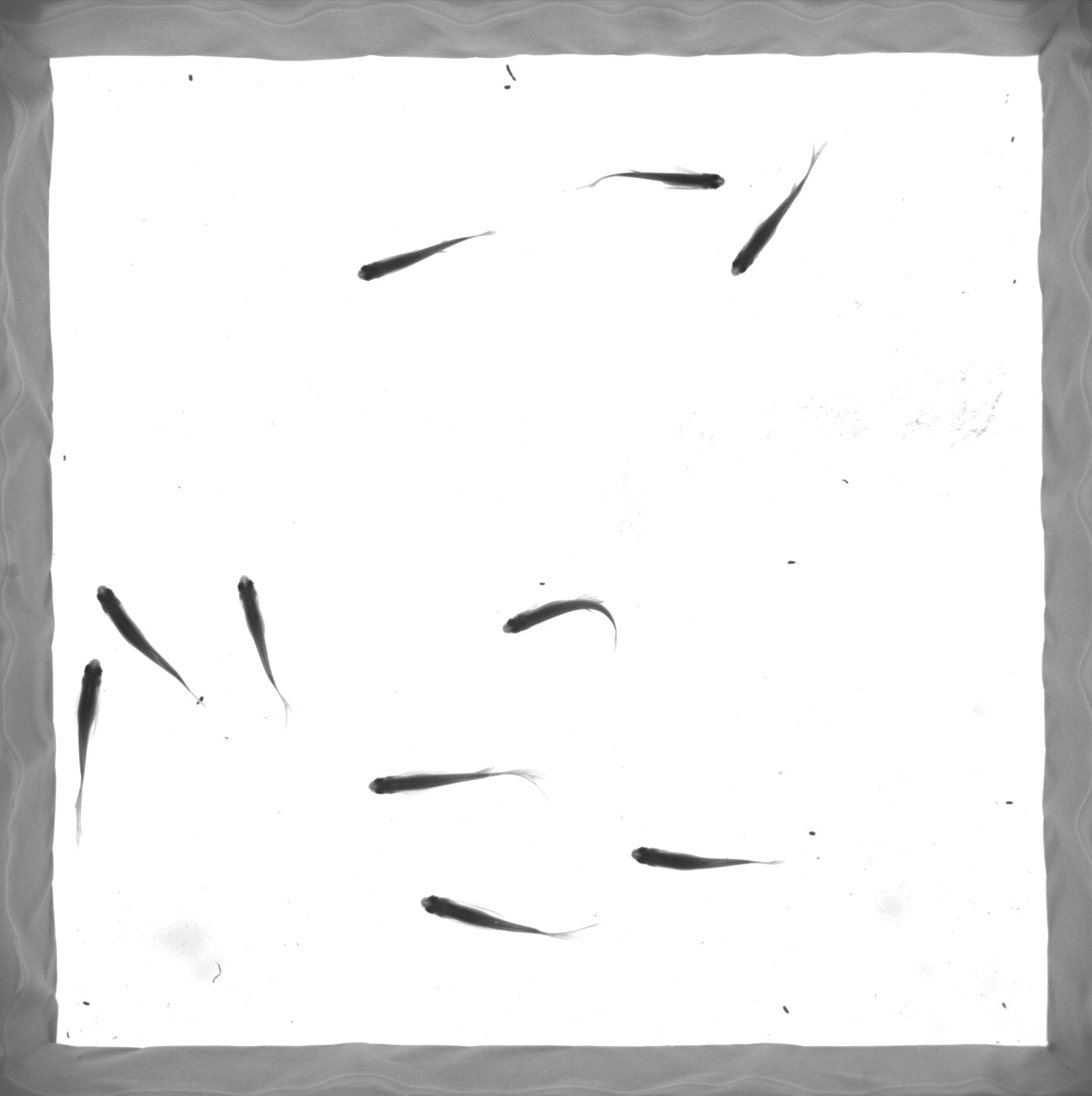

Supplement: S1 File — Source code of the proposed tracking system. (ZIP) [file pone.0154714.s002.zip › code_final/images/CoreView_275_Master_Camera_00039.jpg]

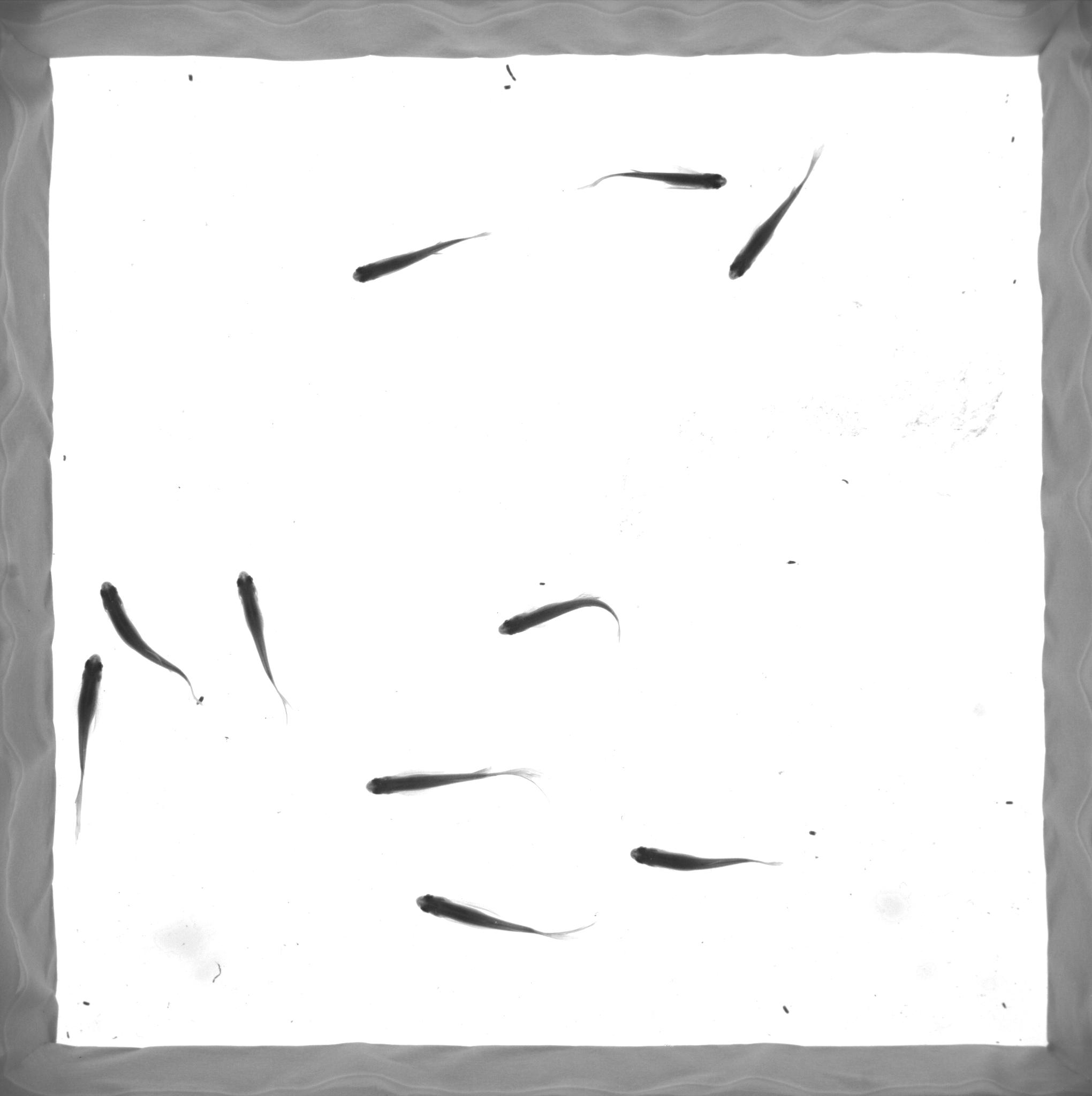

Supplement: S1 File — Source code of the proposed tracking system. (ZIP) [file pone.0154714.s002.zip › code_final/images/CoreView_275_Master_Camera_00040.jpg]

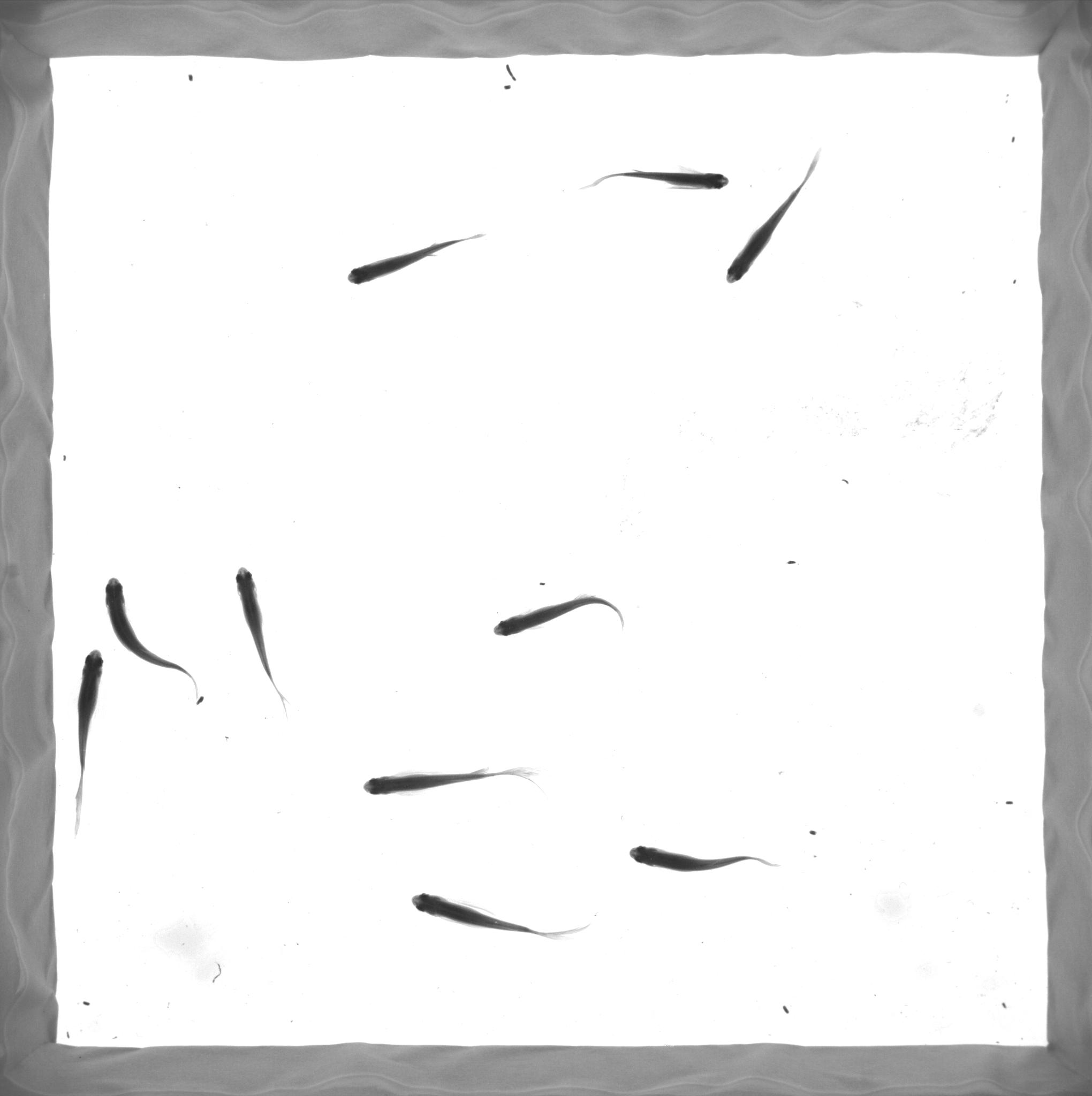

Supplement: S1 File — Source code of the proposed tracking system. (ZIP) [file pone.0154714.s002.zip › code_final/images/CoreView_275_Master_Camera_00041.jpg]

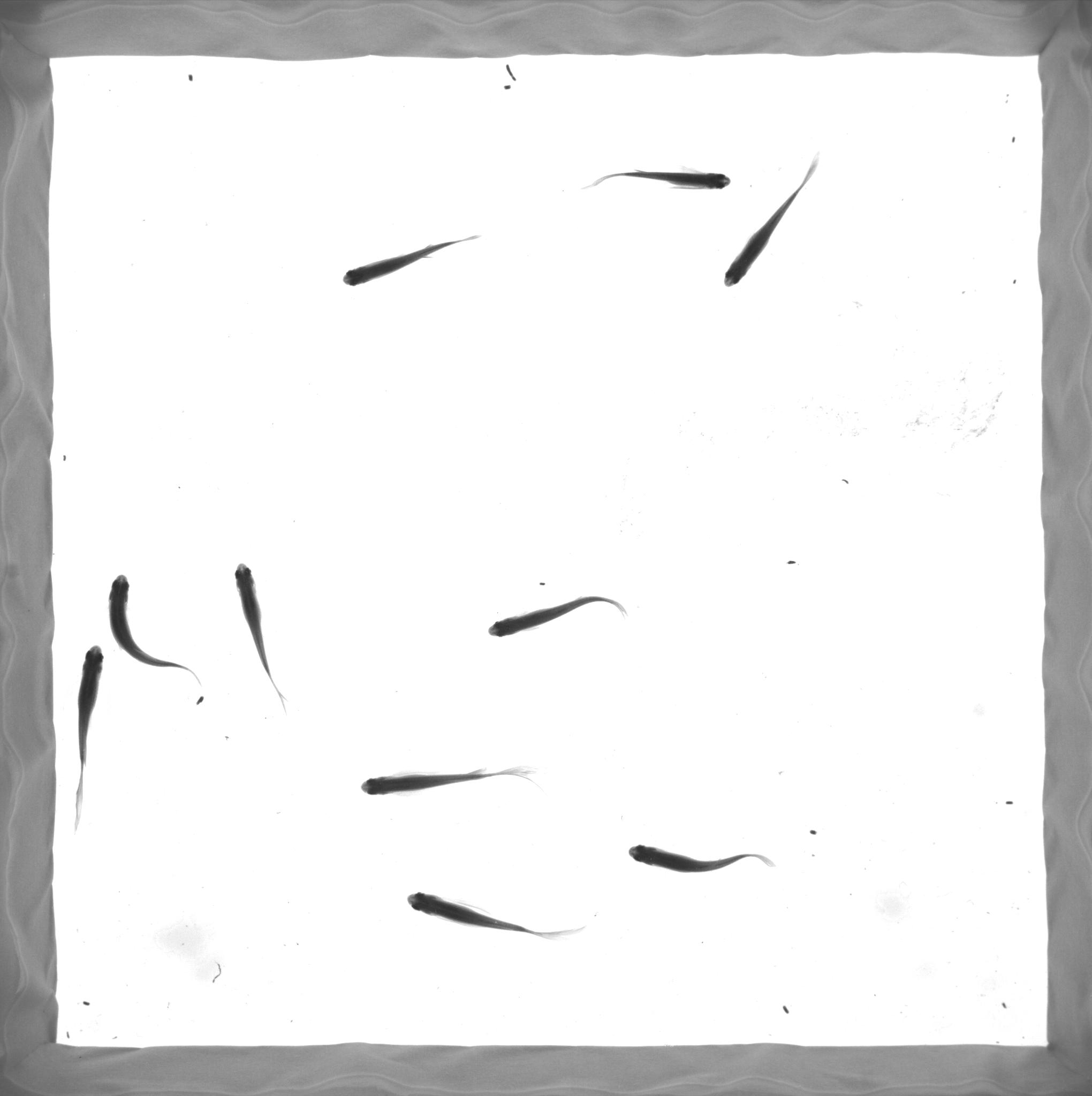

Supplement: S1 File — Source code of the proposed tracking system. (ZIP) [file pone.0154714.s002.zip › code_final/images/CoreView_275_Master_Camera_00042.jpg]

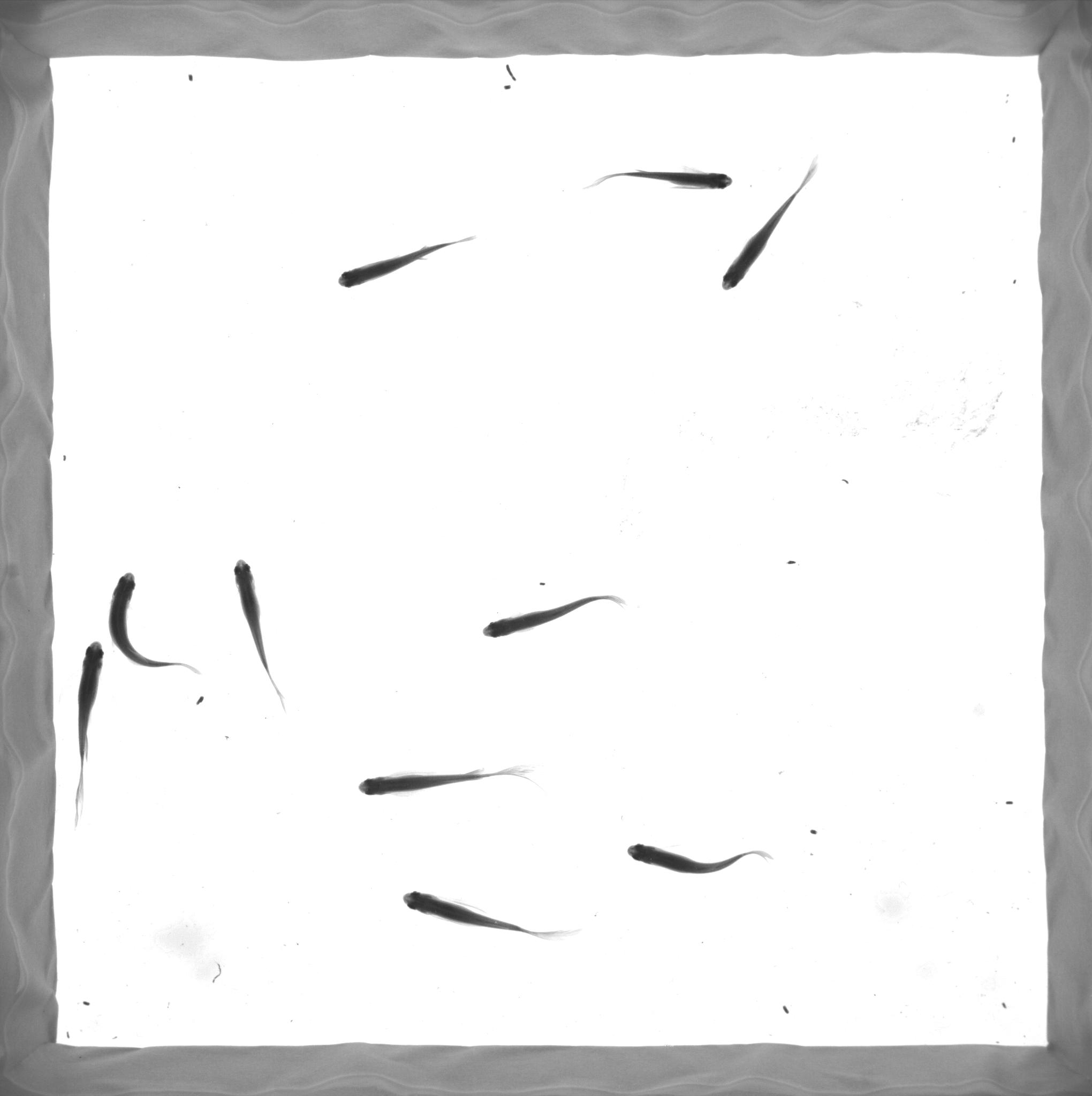

Supplement: S1 File — Source code of the proposed tracking system. (ZIP) [file pone.0154714.s002.zip › code_final/images/CoreView_275_Master_Camera_00043.jpg]

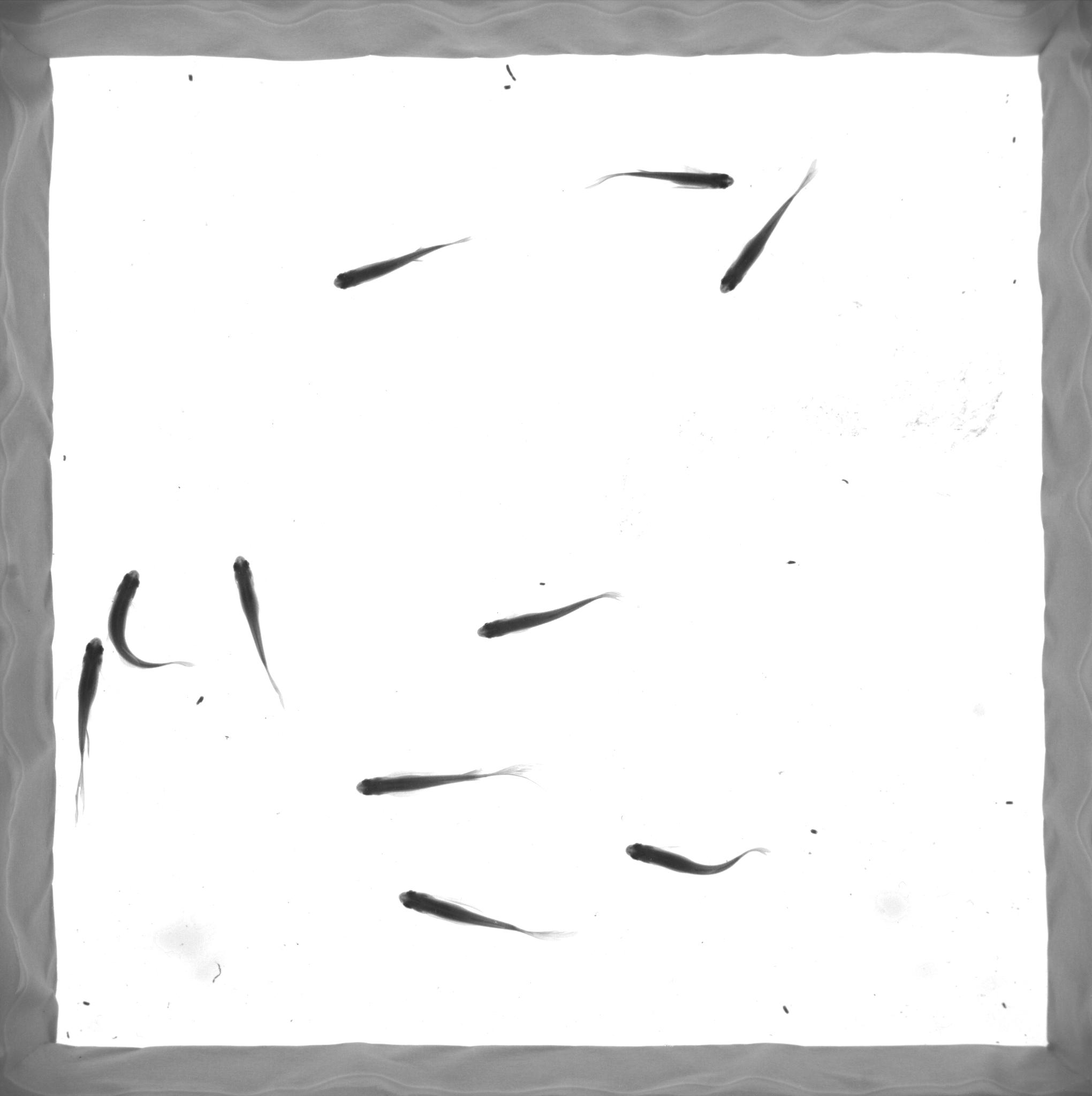

Supplement: S1 File — Source code of the proposed tracking system. (ZIP) [file pone.0154714.s002.zip › code_final/images/CoreView_275_Master_Camera_00044.jpg]

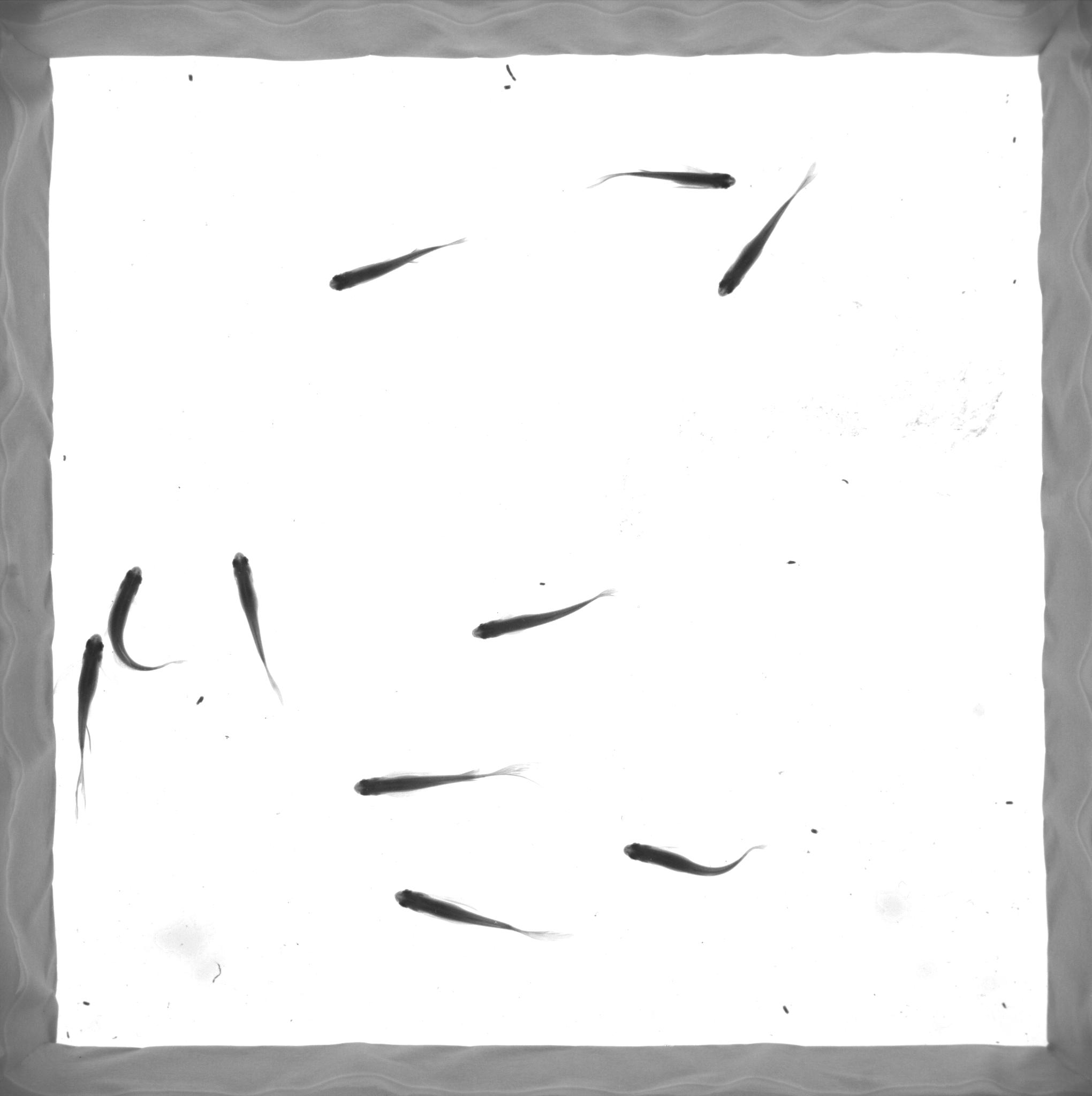

Supplement: S1 File — Source code of the proposed tracking system. (ZIP) [file pone.0154714.s002.zip › code_final/images/CoreView_275_Master_Camera_00045.jpg]

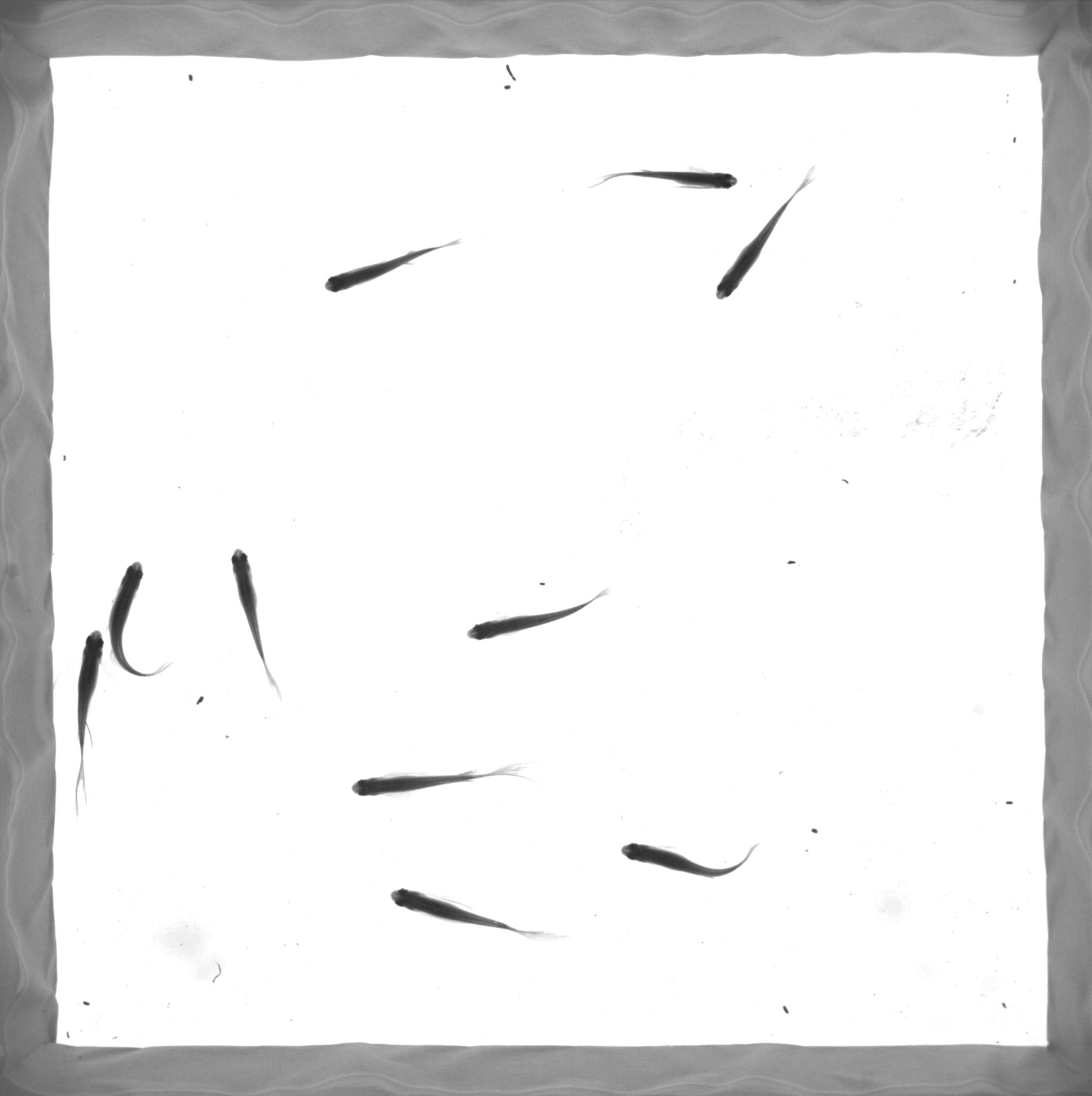

Supplement: S1 File — Source code of the proposed tracking system. (ZIP) [file pone.0154714.s002.zip › code_final/images/CoreView_275_Master_Camera_00046.jpg]

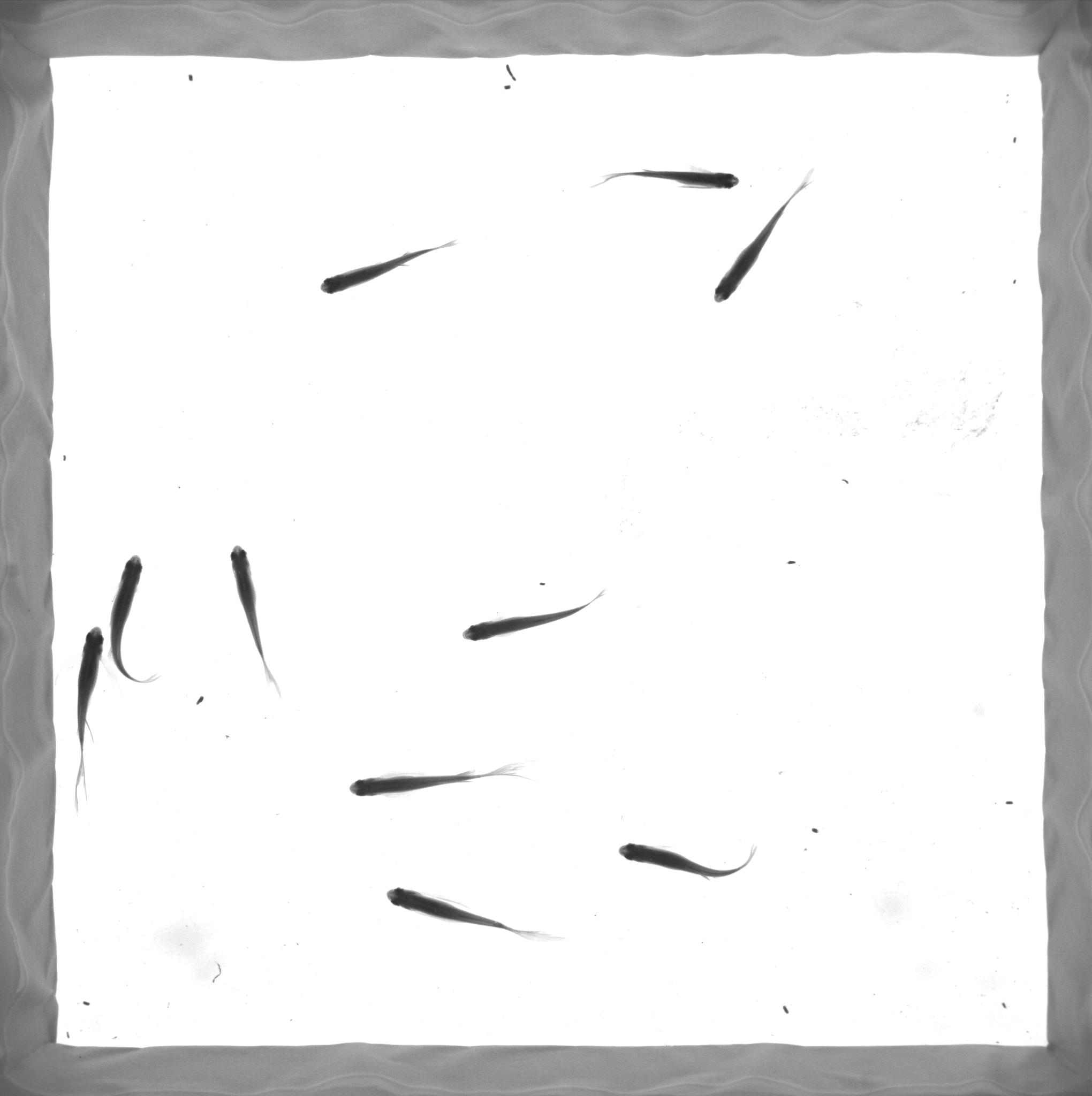

Supplement: S1 File — Source code of the proposed tracking system. (ZIP) [file pone.0154714.s002.zip › code_final/images/CoreView_275_Master_Camera_00047.jpg]

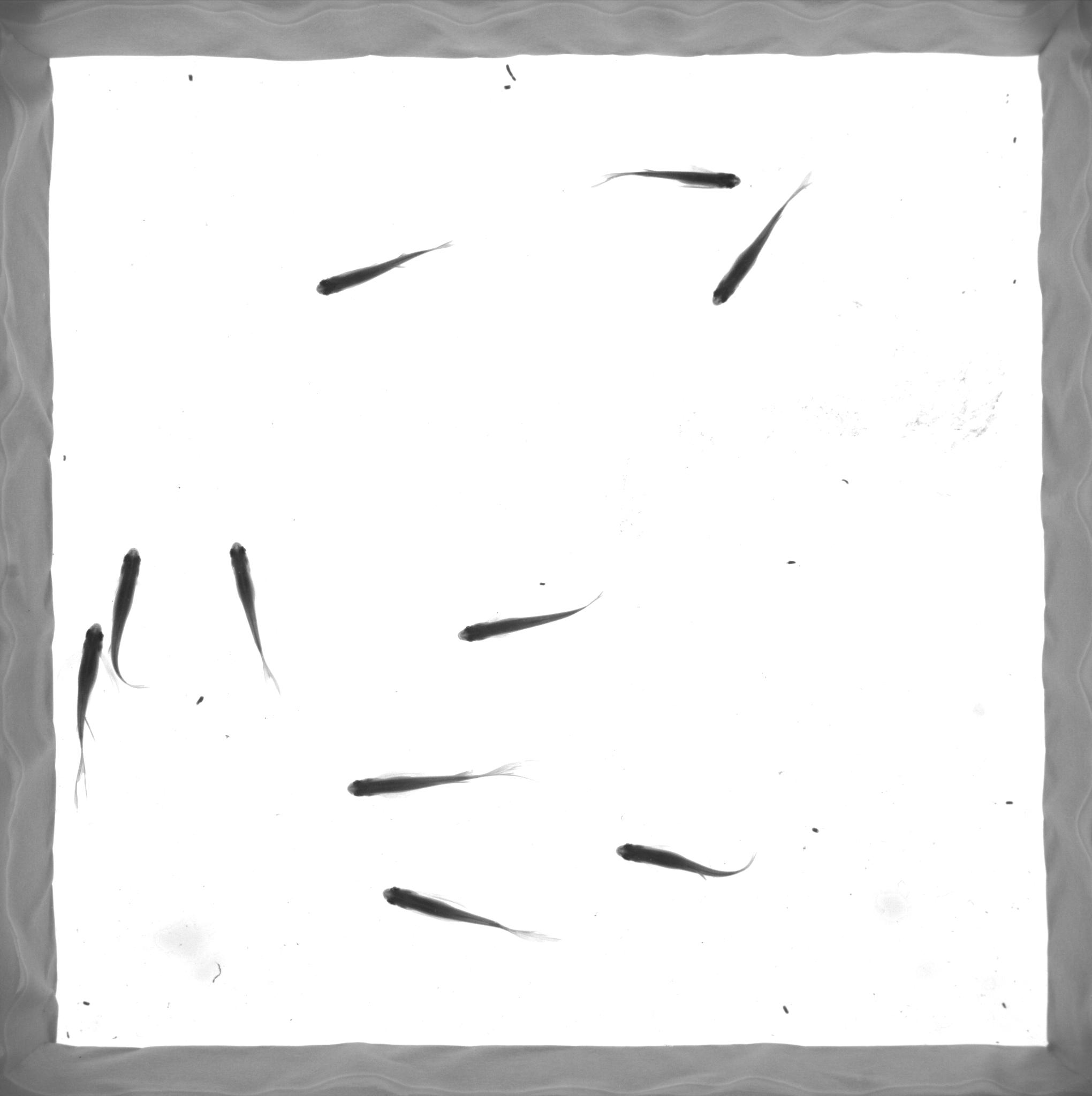

Supplement: S1 File — Source code of the proposed tracking system. (ZIP) [file pone.0154714.s002.zip › code_final/images/CoreView_275_Master_Camera_00048.jpg]

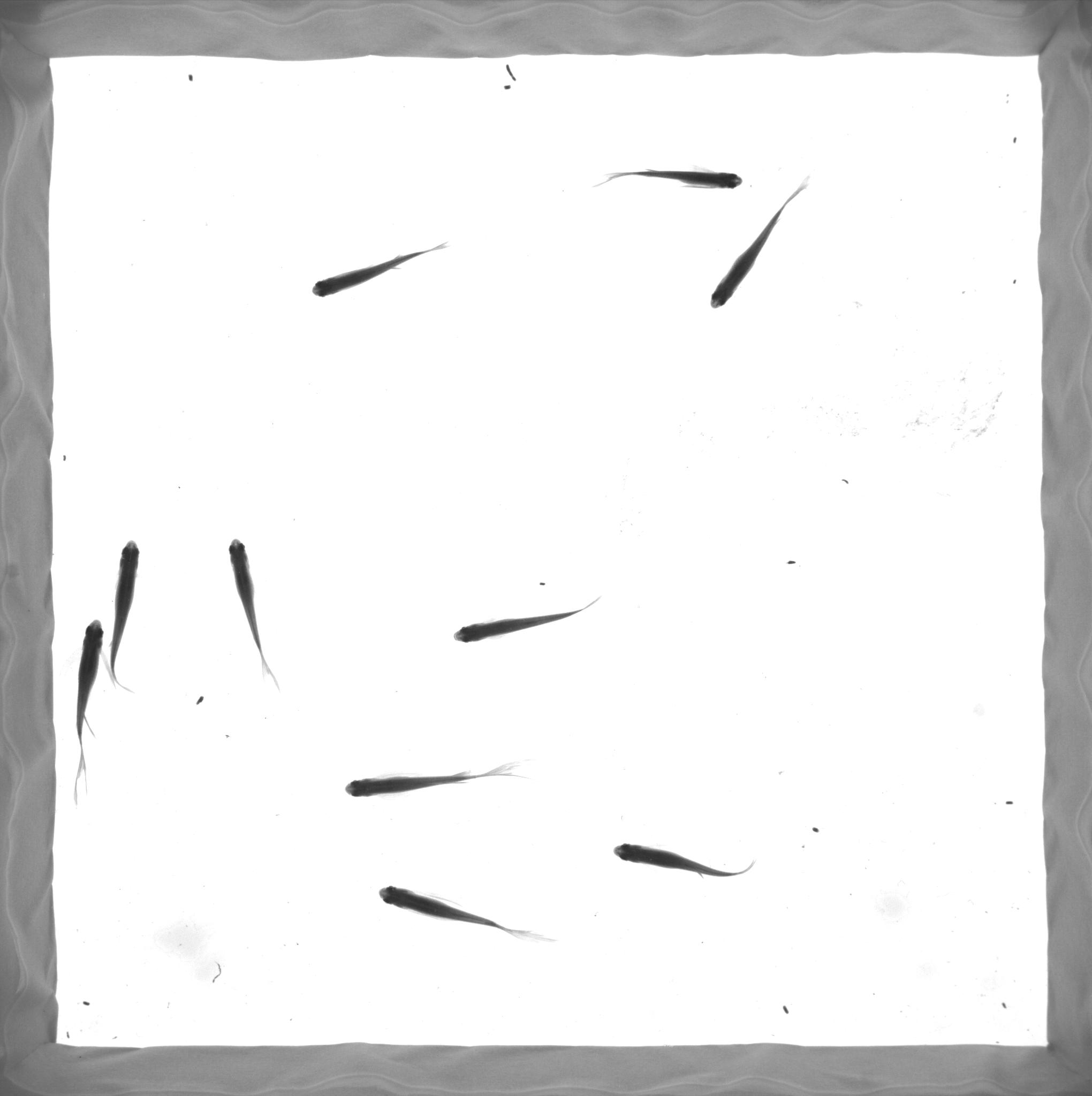

Supplement: S1 File — Source code of the proposed tracking system. (ZIP) [file pone.0154714.s002.zip › code_final/images/CoreView_275_Master_Camera_00049.jpg]

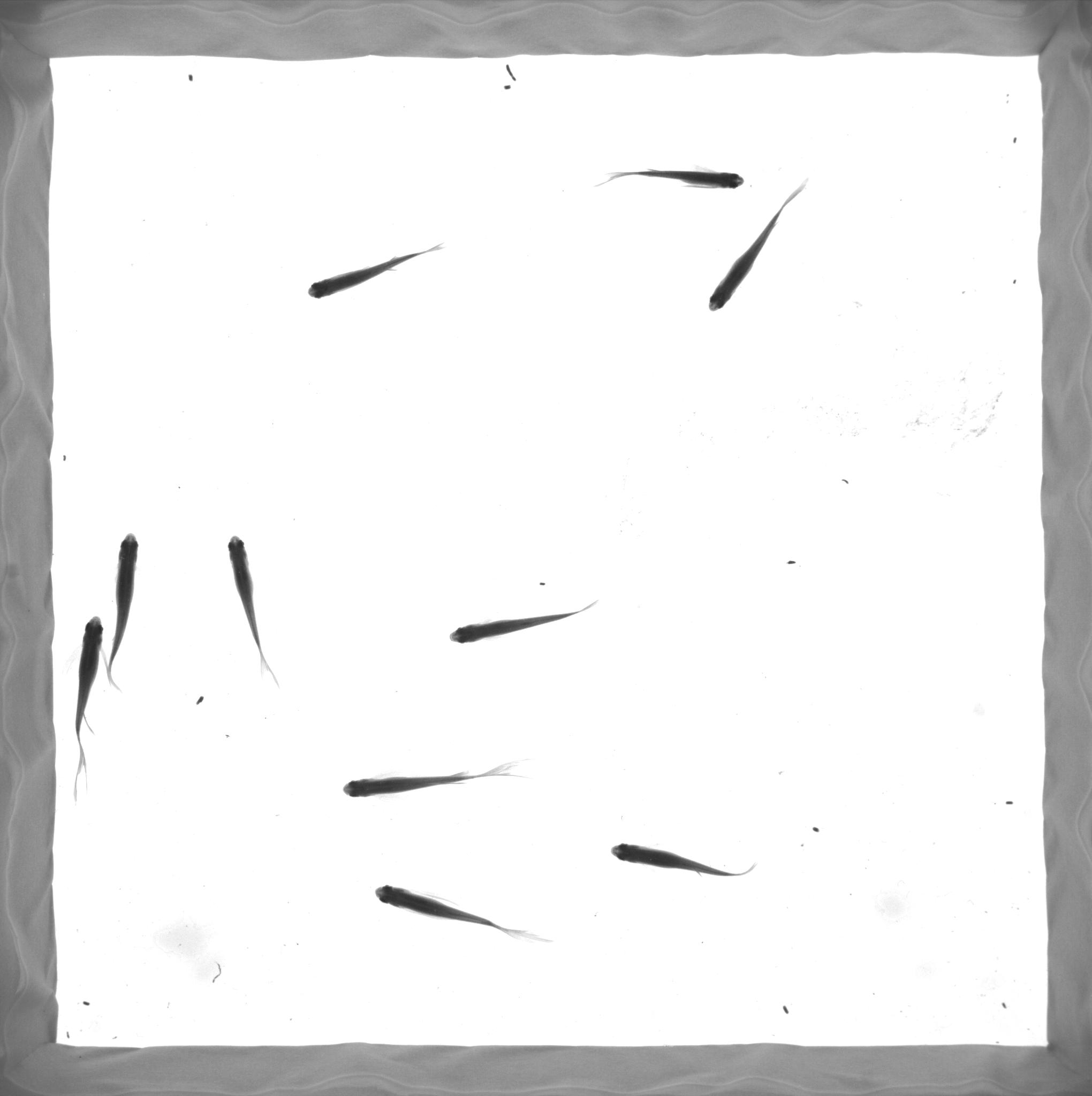

Supplement: S1 File — Source code of the proposed tracking system. (ZIP) [file pone.0154714.s002.zip › code_final/images/CoreView_275_Master_Camera_00050.jpg]

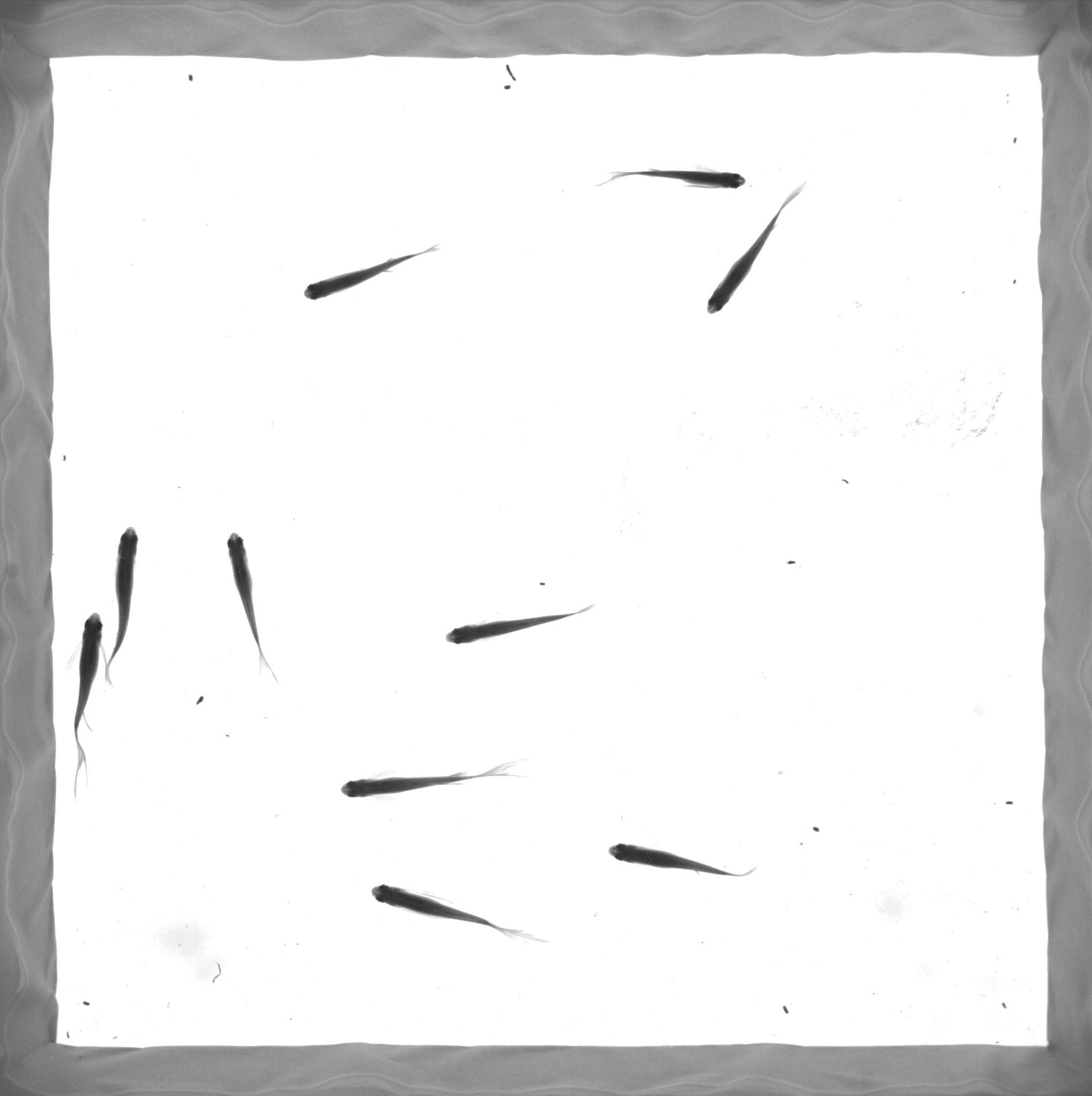

Supplement: S1 File — Source code of the proposed tracking system. (ZIP) [file pone.0154714.s002.zip › code_final/images/CoreView_275_Master_Camera_00051.jpg]

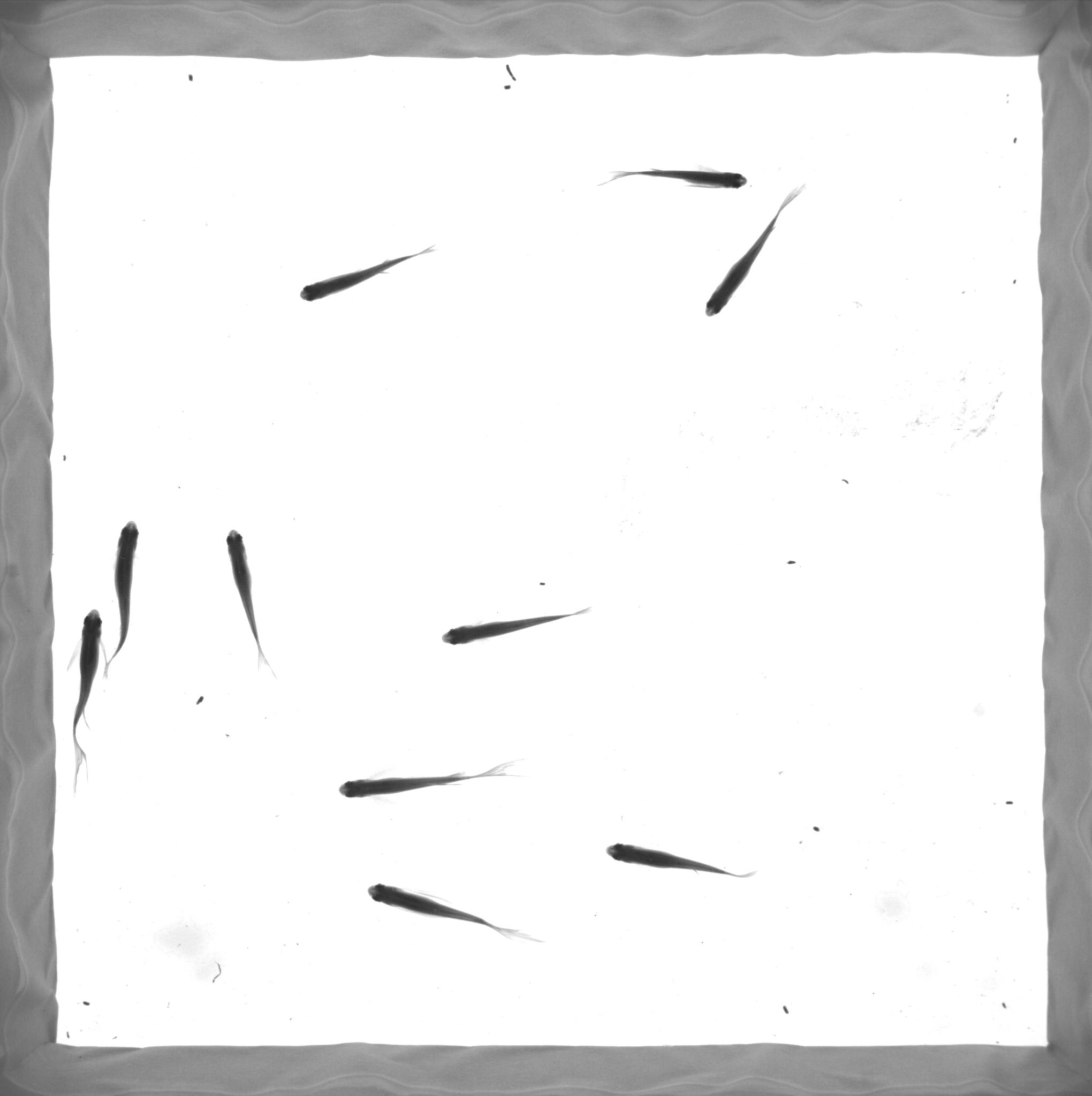

Supplement: S1 File — Source code of the proposed tracking system. (ZIP) [file pone.0154714.s002.zip › code_final/images/CoreView_275_Master_Camera_00052.jpg]

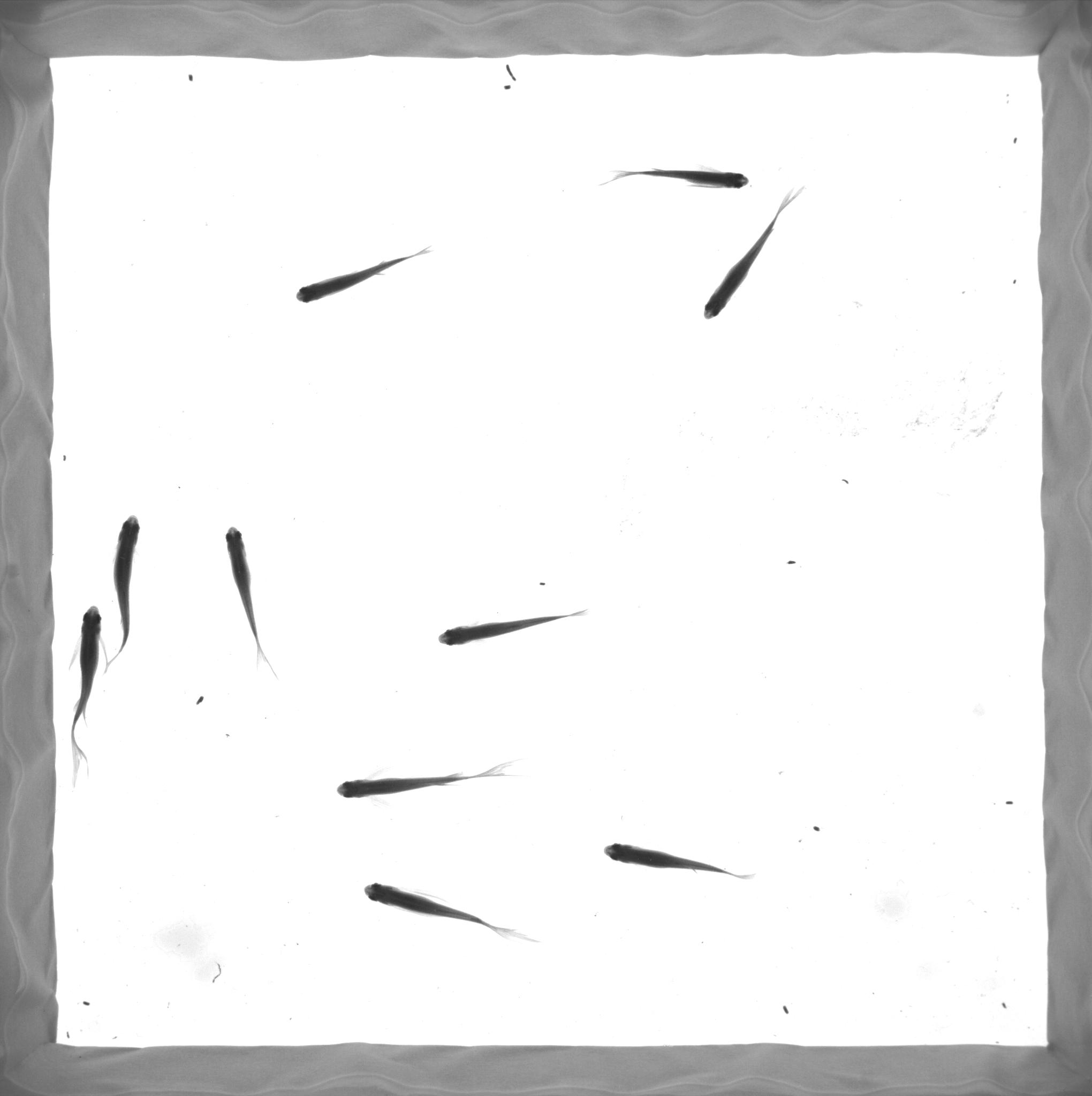

Supplement: S1 File — Source code of the proposed tracking system. (ZIP) [file pone.0154714.s002.zip › code_final/images/CoreView_275_Master_Camera_00053.jpg]

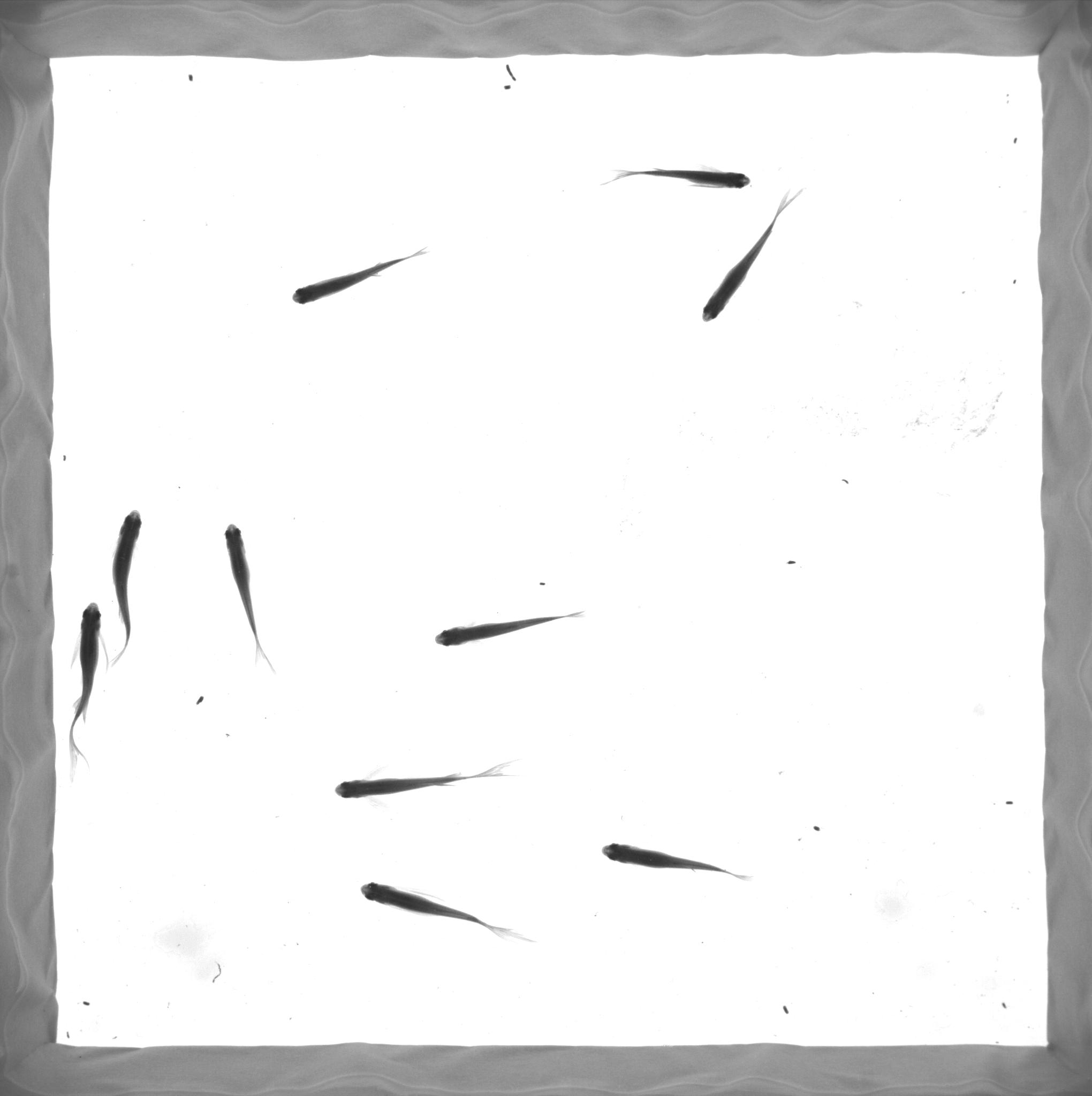

Supplement: S1 File — Source code of the proposed tracking system. (ZIP) [file pone.0154714.s002.zip › code_final/images/CoreView_275_Master_Camera_00054.jpg]

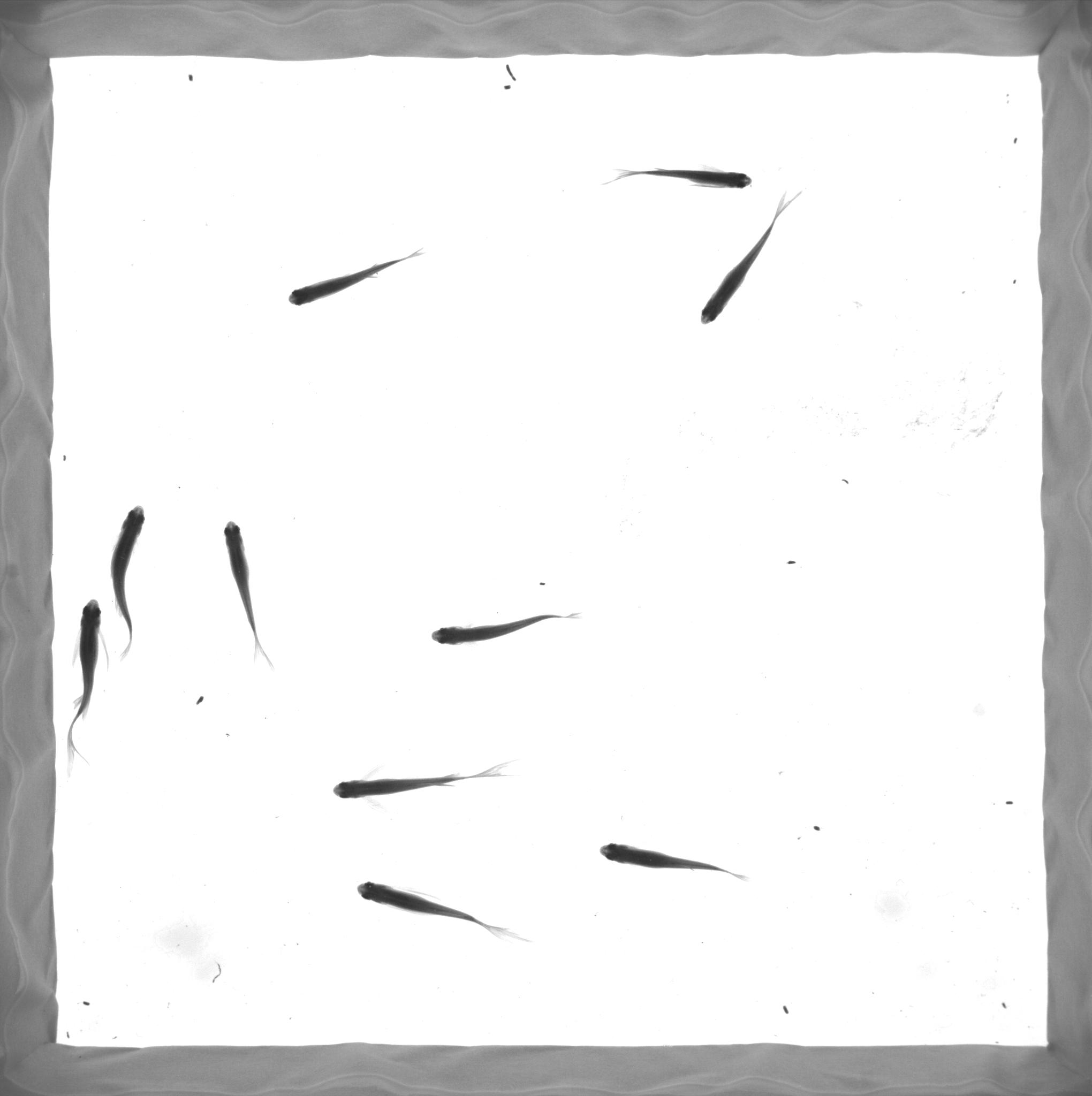

Supplement: S1 File — Source code of the proposed tracking system. (ZIP) [file pone.0154714.s002.zip › code_final/images/CoreView_275_Master_Camera_00055.jpg]

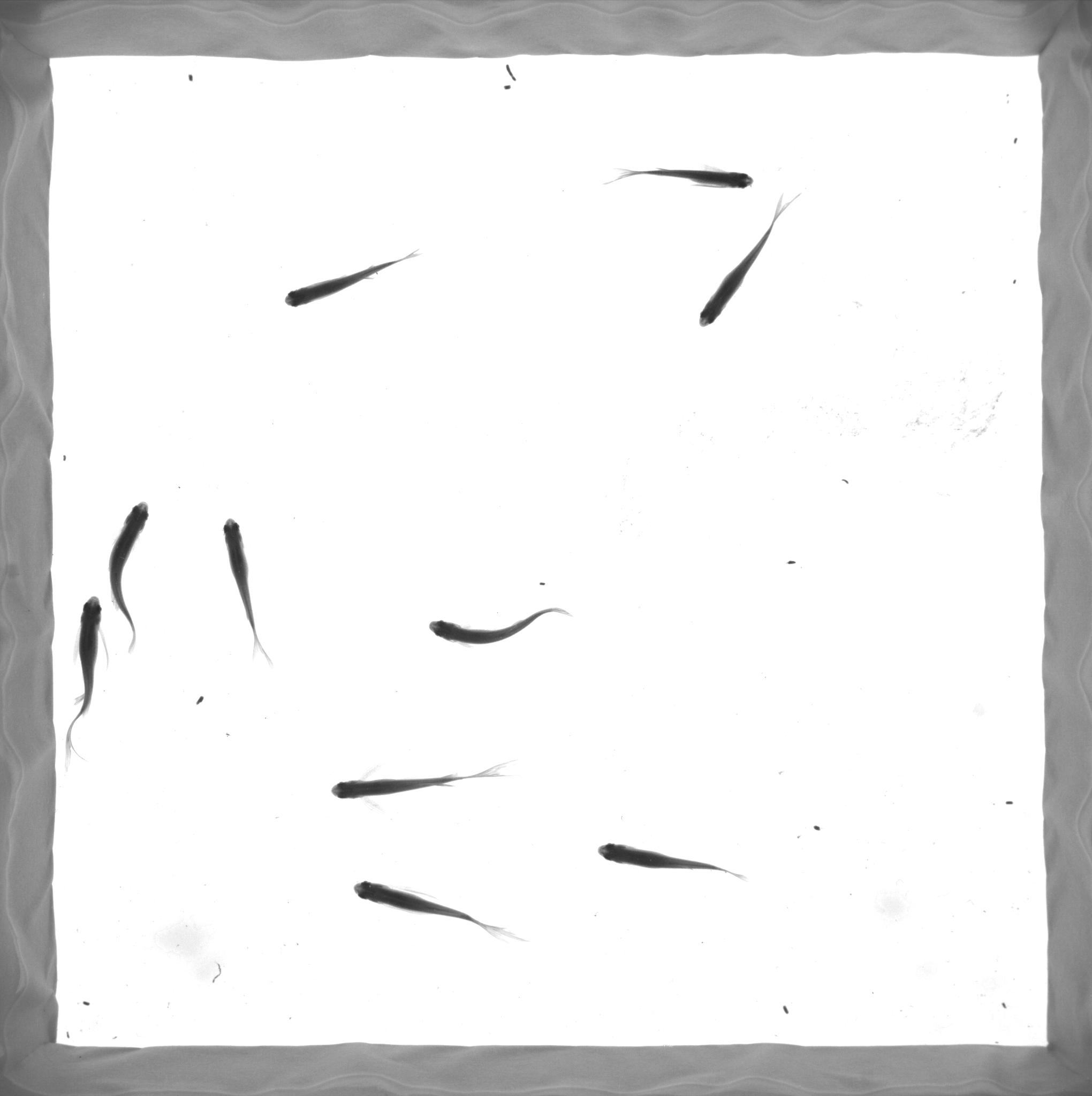

Supplement: S1 File — Source code of the proposed tracking system. (ZIP) [file pone.0154714.s002.zip › code_final/images/CoreView_275_Master_Camera_00056.jpg]

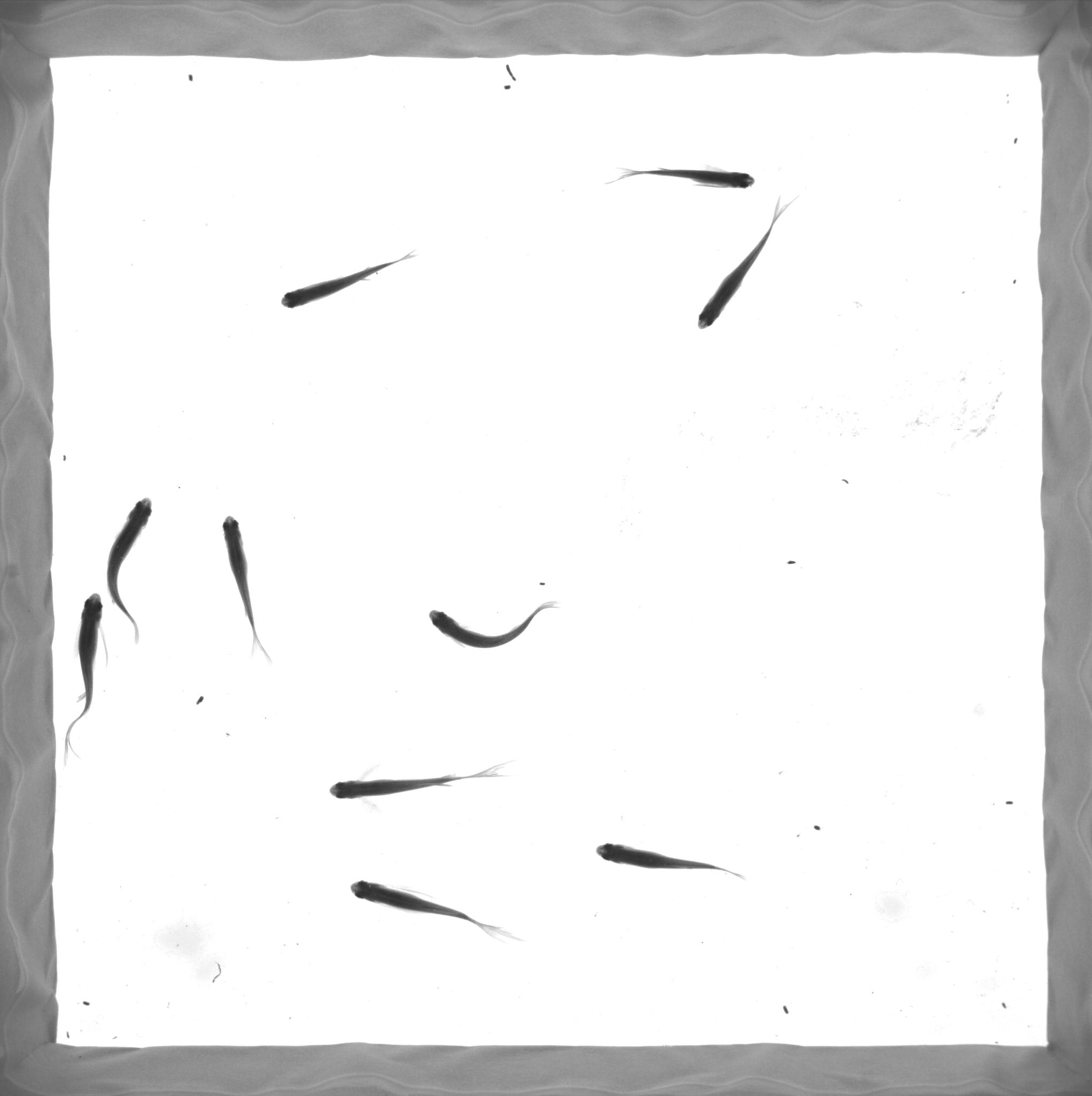

Supplement: S1 File — Source code of the proposed tracking system. (ZIP) [file pone.0154714.s002.zip › code_final/images/CoreView_275_Master_Camera_00057.jpg]

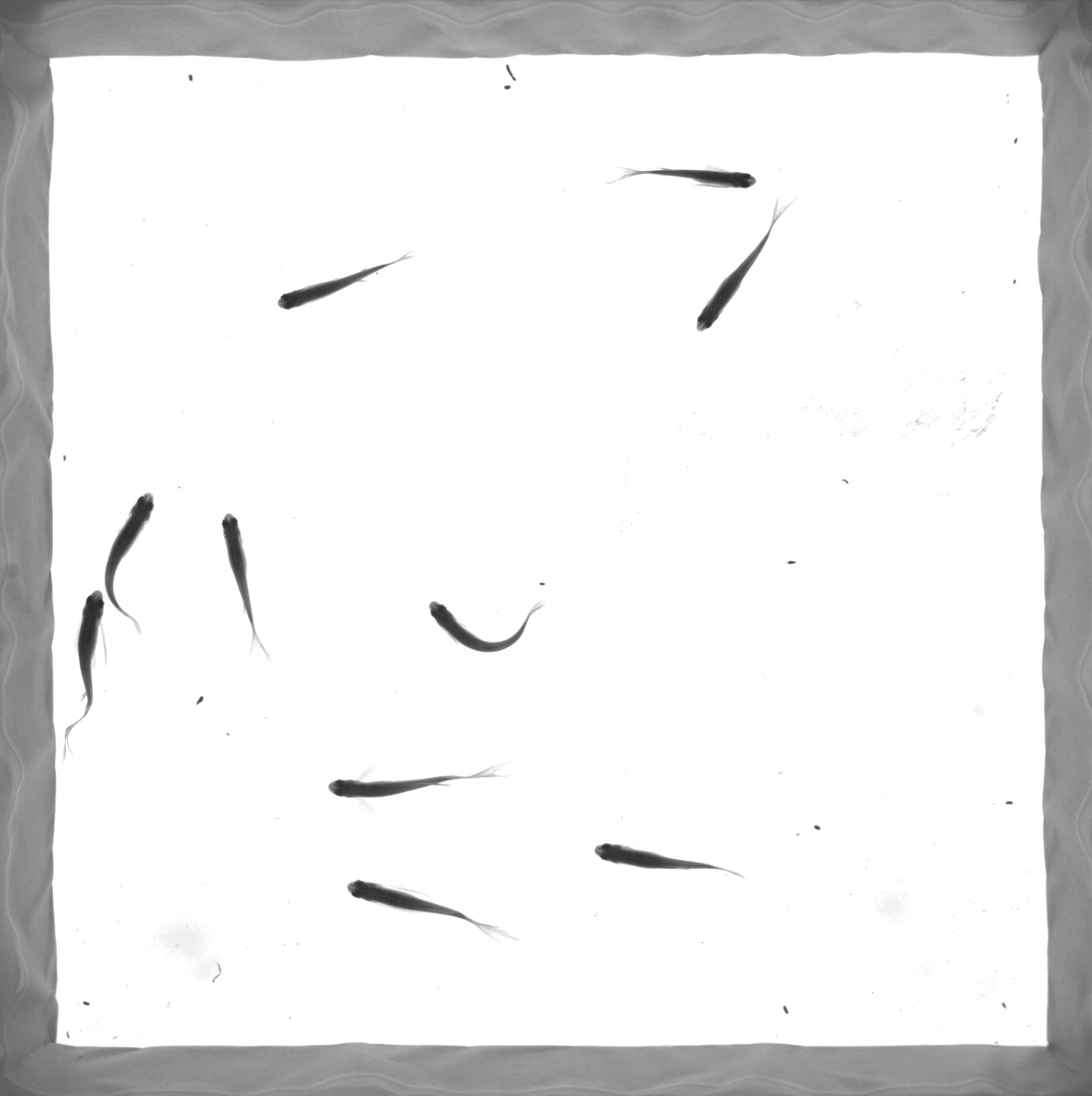

Supplement: S1 File — Source code of the proposed tracking system. (ZIP) [file pone.0154714.s002.zip › code_final/images/CoreView_275_Master_Camera_00058.jpg]

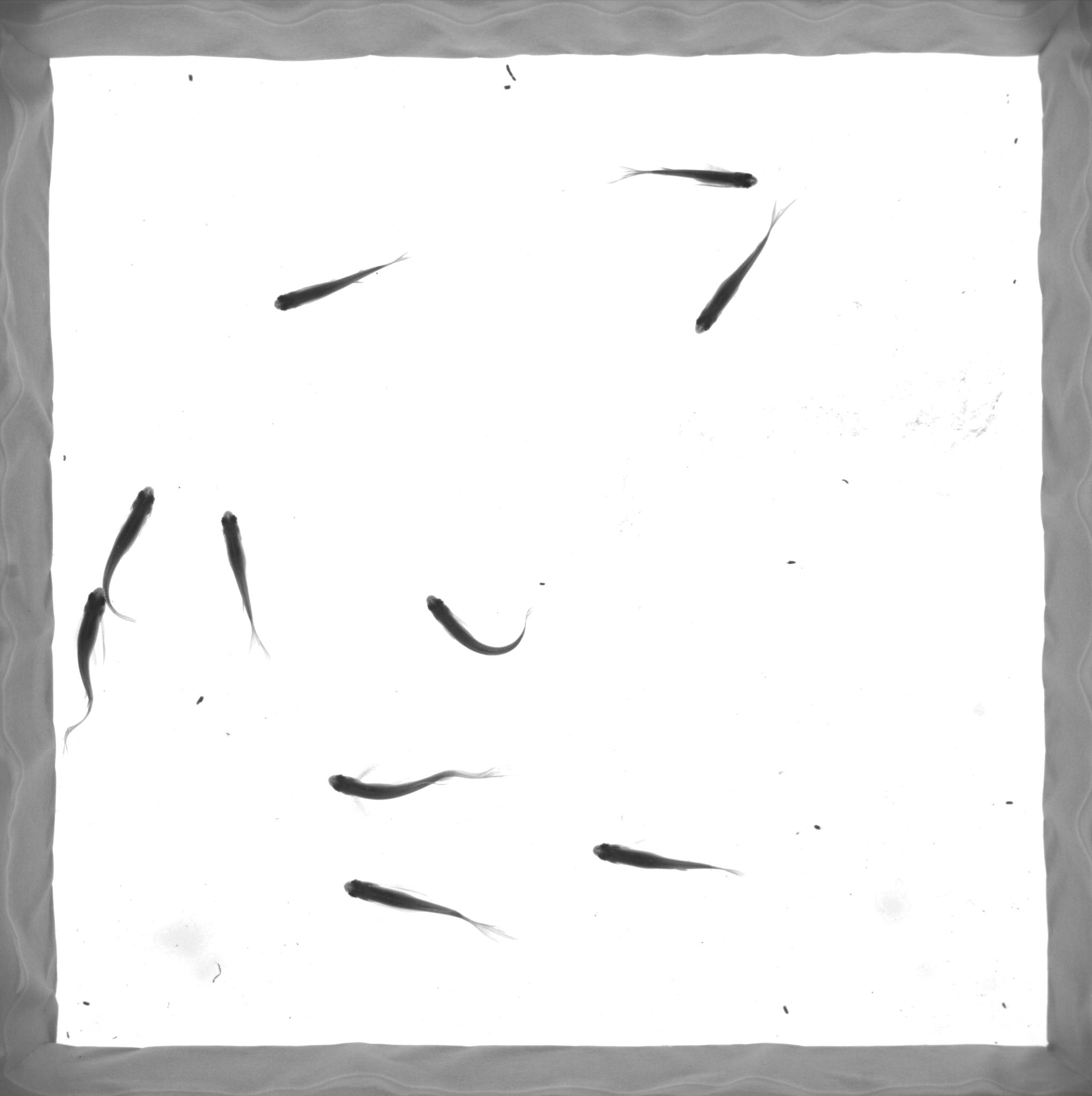

Supplement: S1 File — Source code of the proposed tracking system. (ZIP) [file pone.0154714.s002.zip › code_final/images/CoreView_275_Master_Camera_00059.jpg]

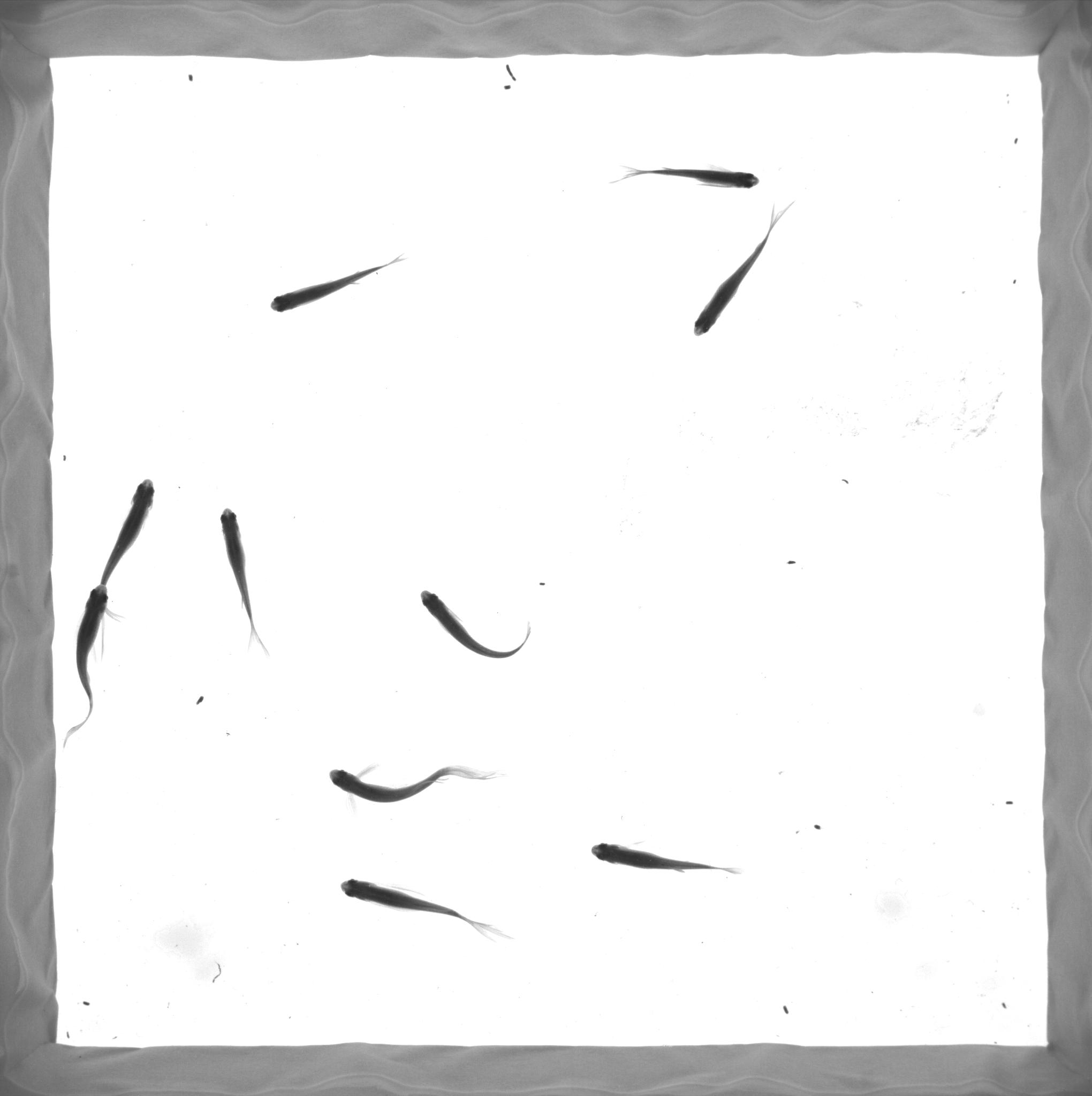

Supplement: S1 File — Source code of the proposed tracking system. (ZIP) [file pone.0154714.s002.zip › code_final/images/CoreView_275_Master_Camera_00060.jpg]

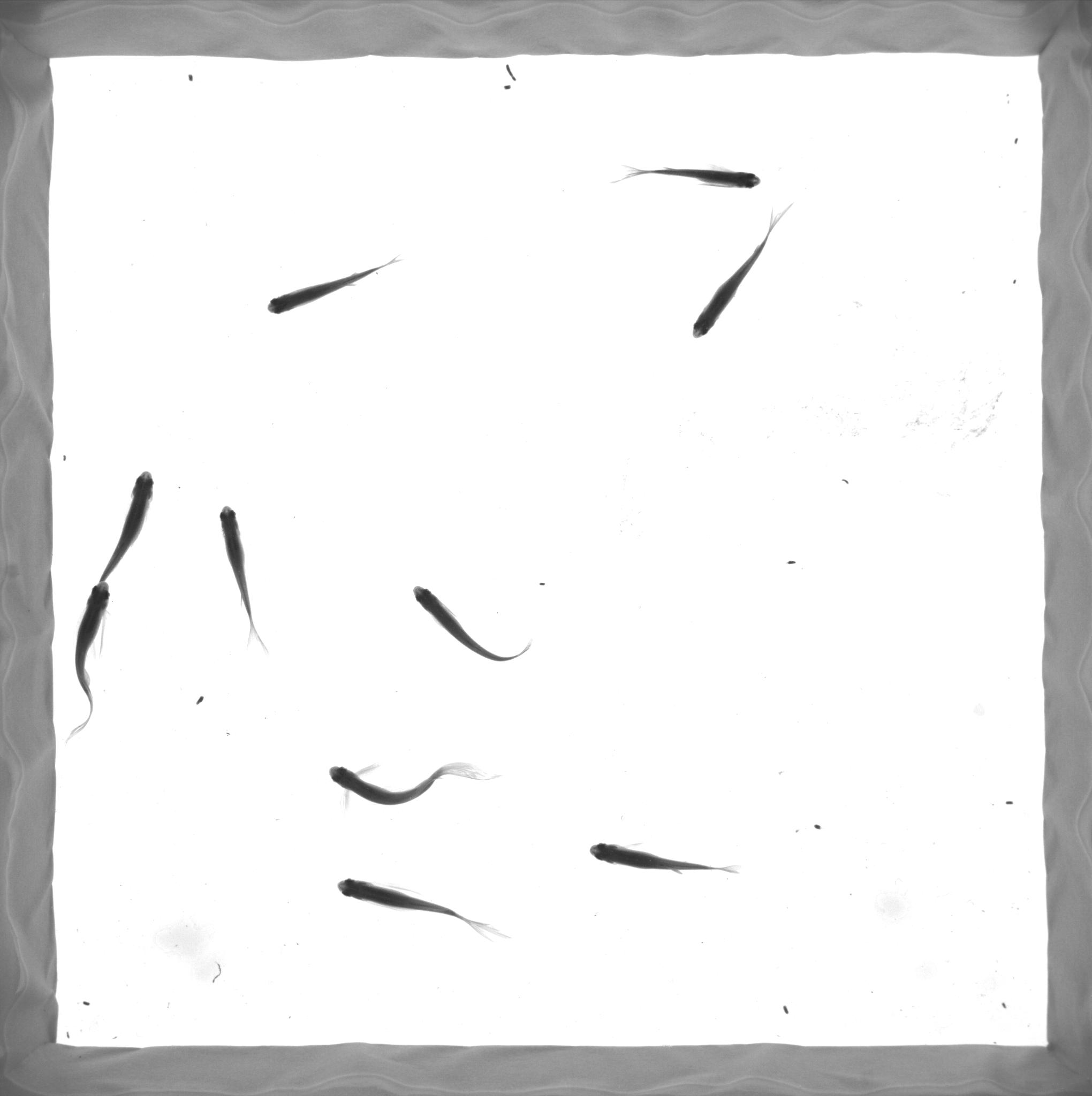

Supplement: S1 File — Source code of the proposed tracking system. (ZIP) [file pone.0154714.s002.zip › code_final/images/CoreView_275_Master_Camera_00061.jpg]

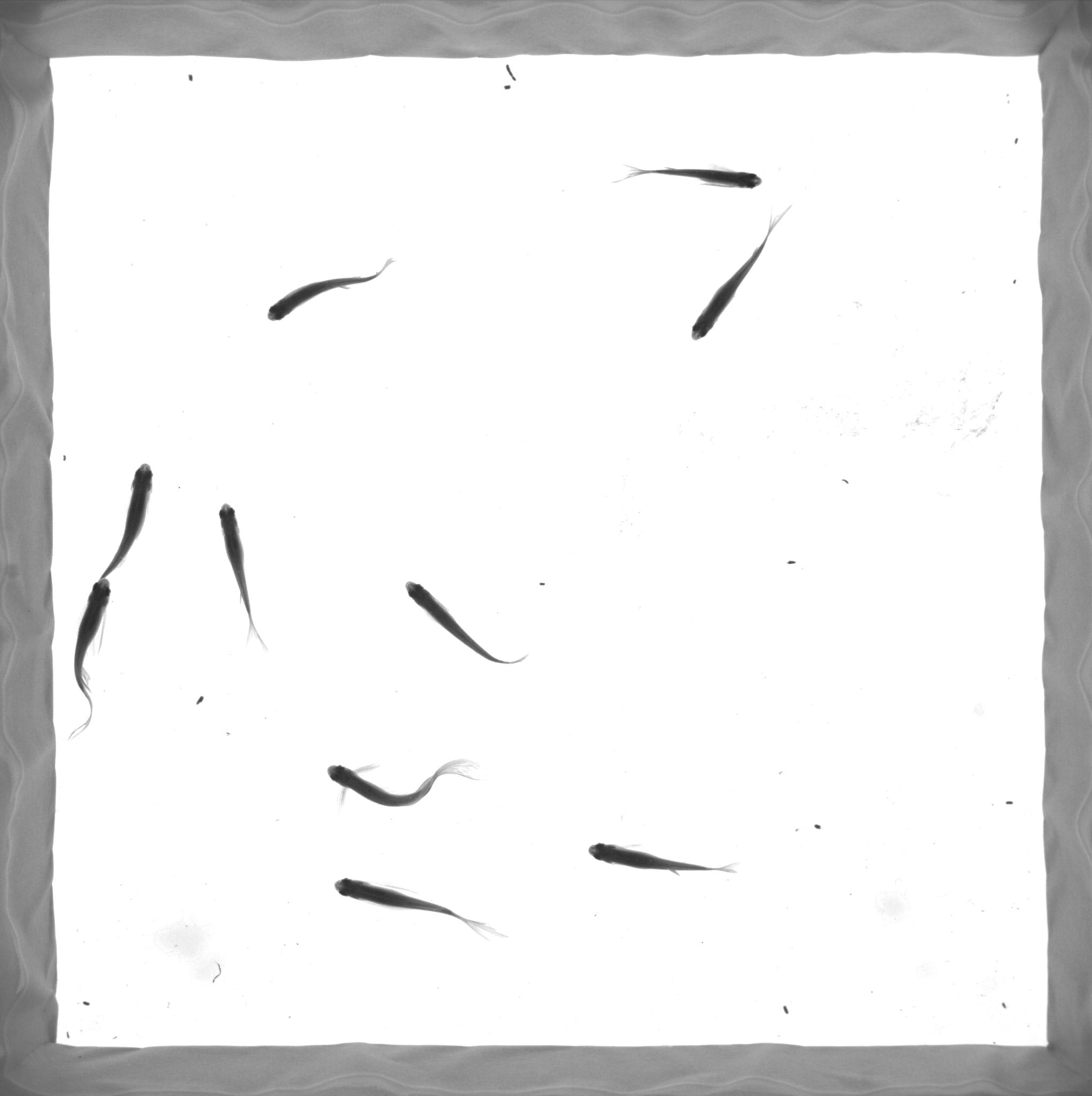

Supplement: S1 File — Source code of the proposed tracking system. (ZIP) [file pone.0154714.s002.zip › code_final/images/CoreView_275_Master_Camera_00062.jpg]

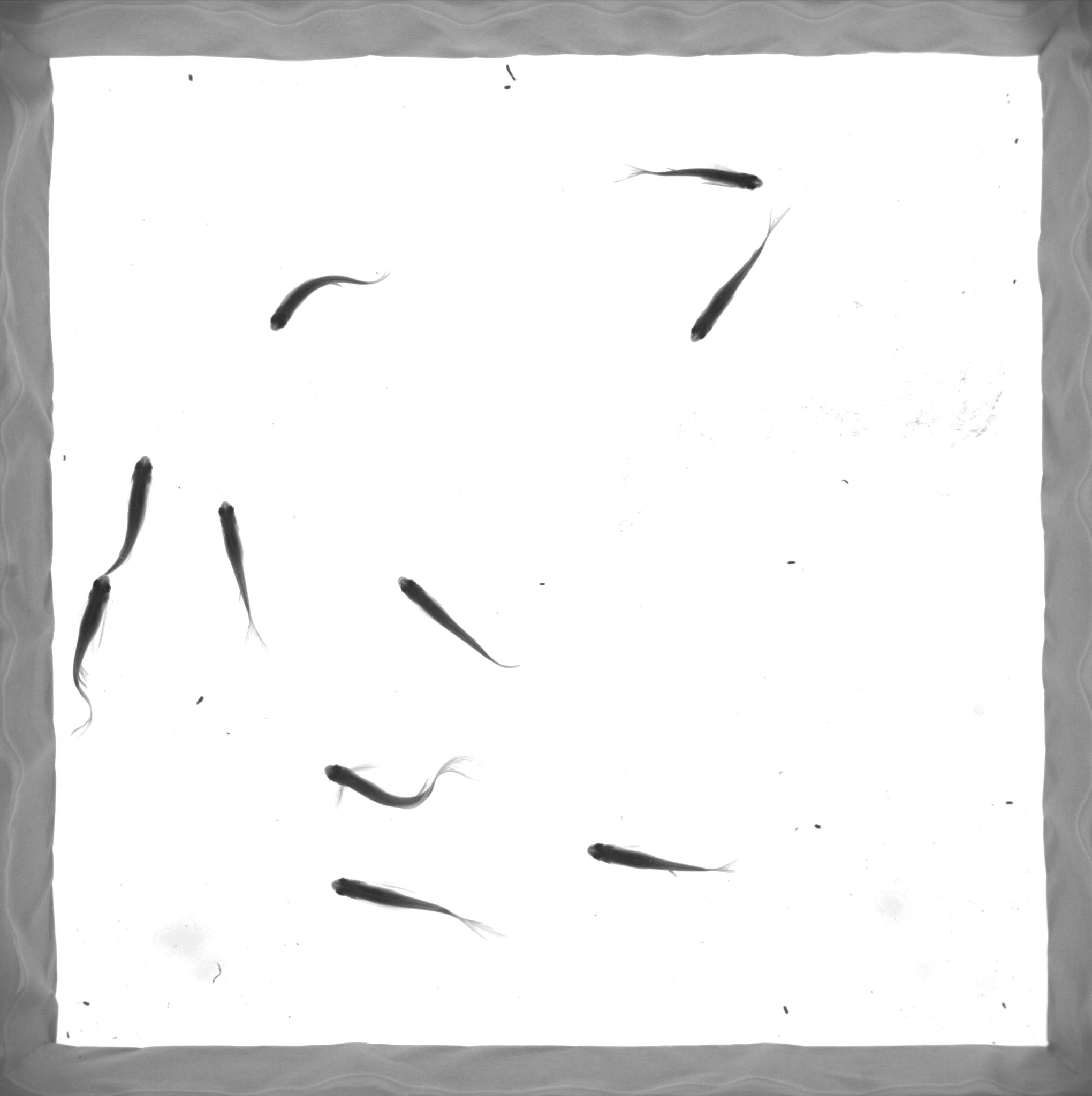

Supplement: S1 File — Source code of the proposed tracking system. (ZIP) [file pone.0154714.s002.zip › code_final/images/CoreView_275_Master_Camera_00063.jpg]

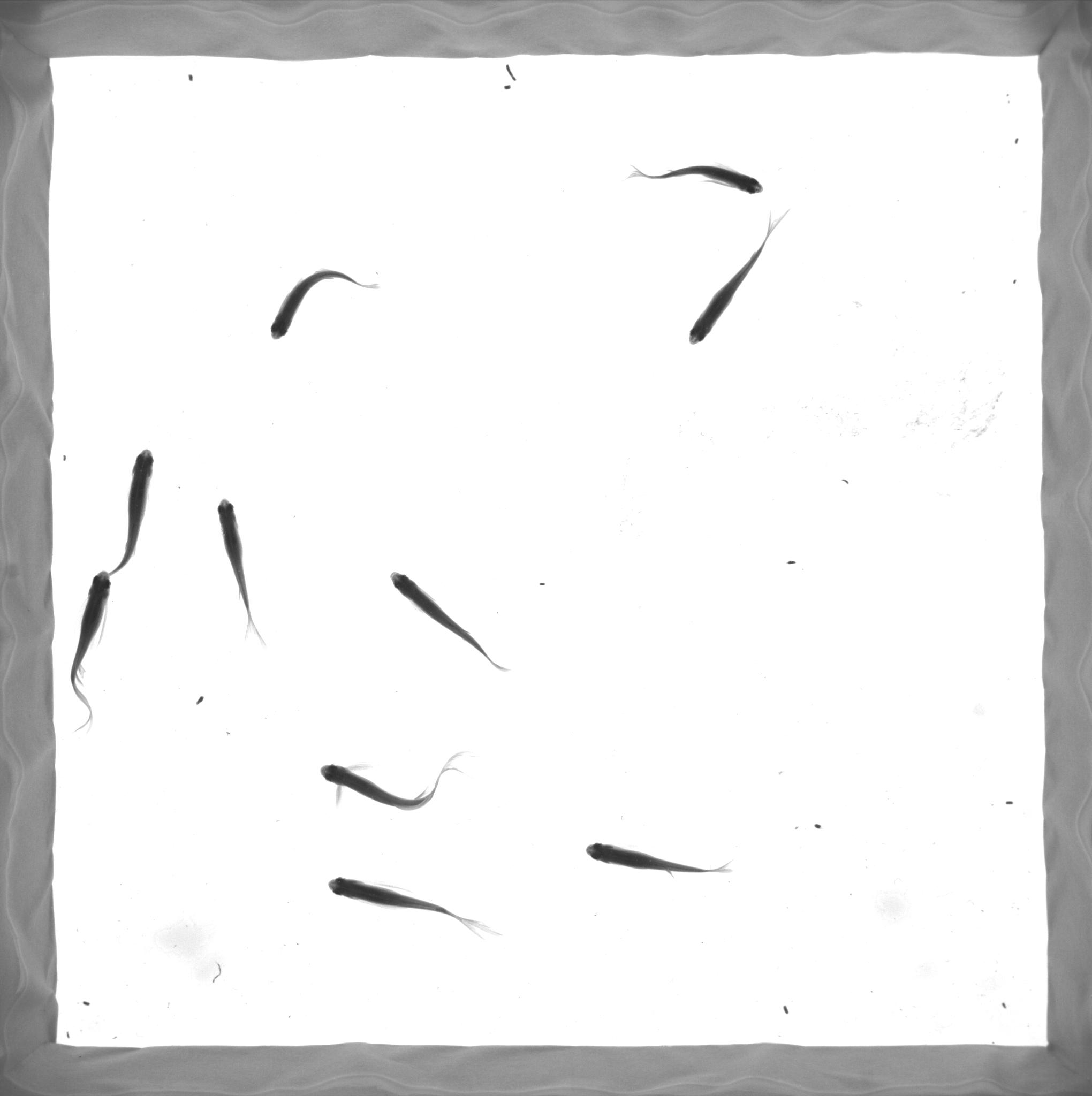

Supplement: S1 File — Source code of the proposed tracking system. (ZIP) [file pone.0154714.s002.zip › code_final/images/CoreView_275_Master_Camera_00064.jpg]

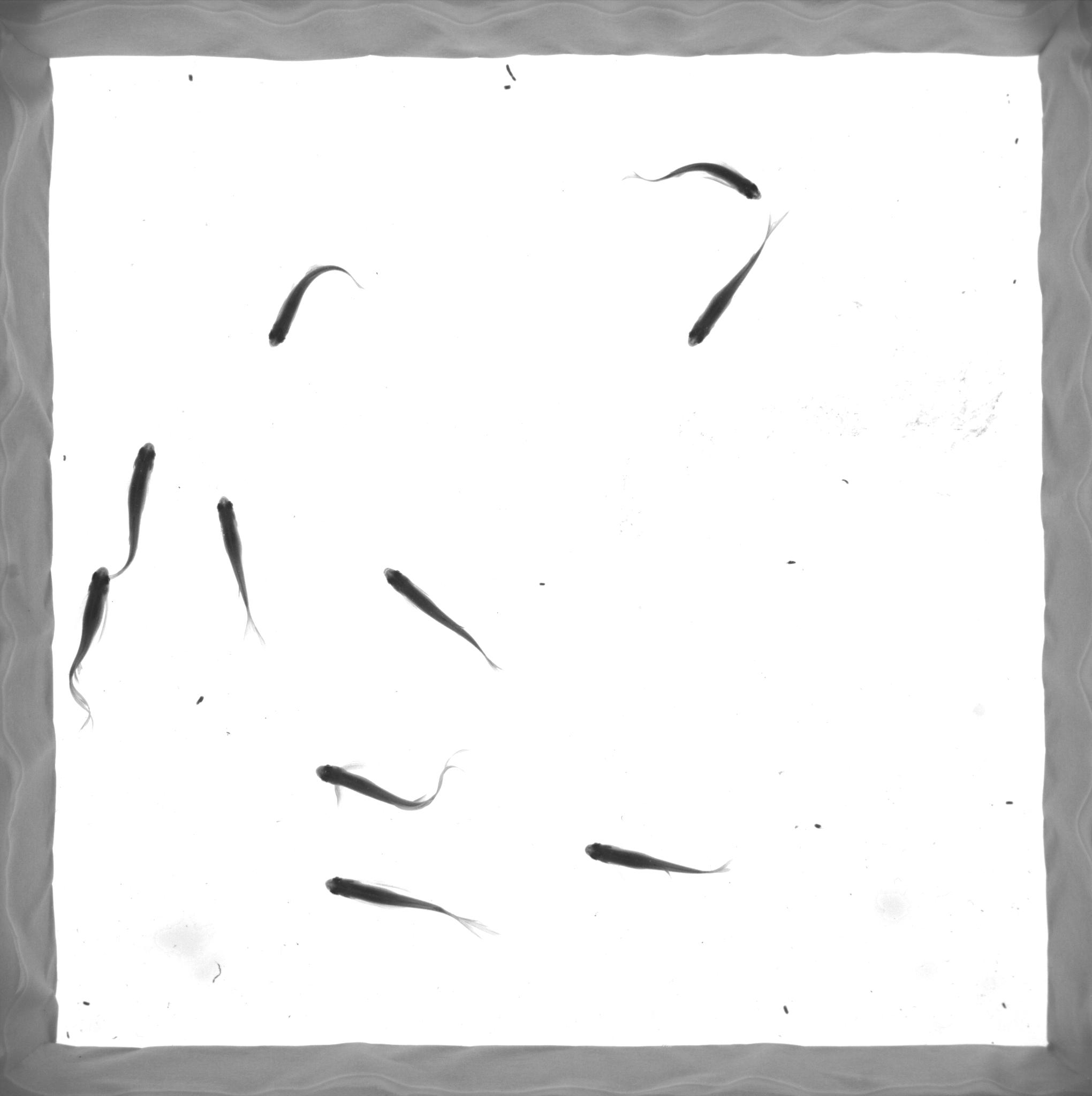

Supplement: S1 File — Source code of the proposed tracking system. (ZIP) [file pone.0154714.s002.zip › code_final/images/CoreView_275_Master_Camera_00065.jpg]

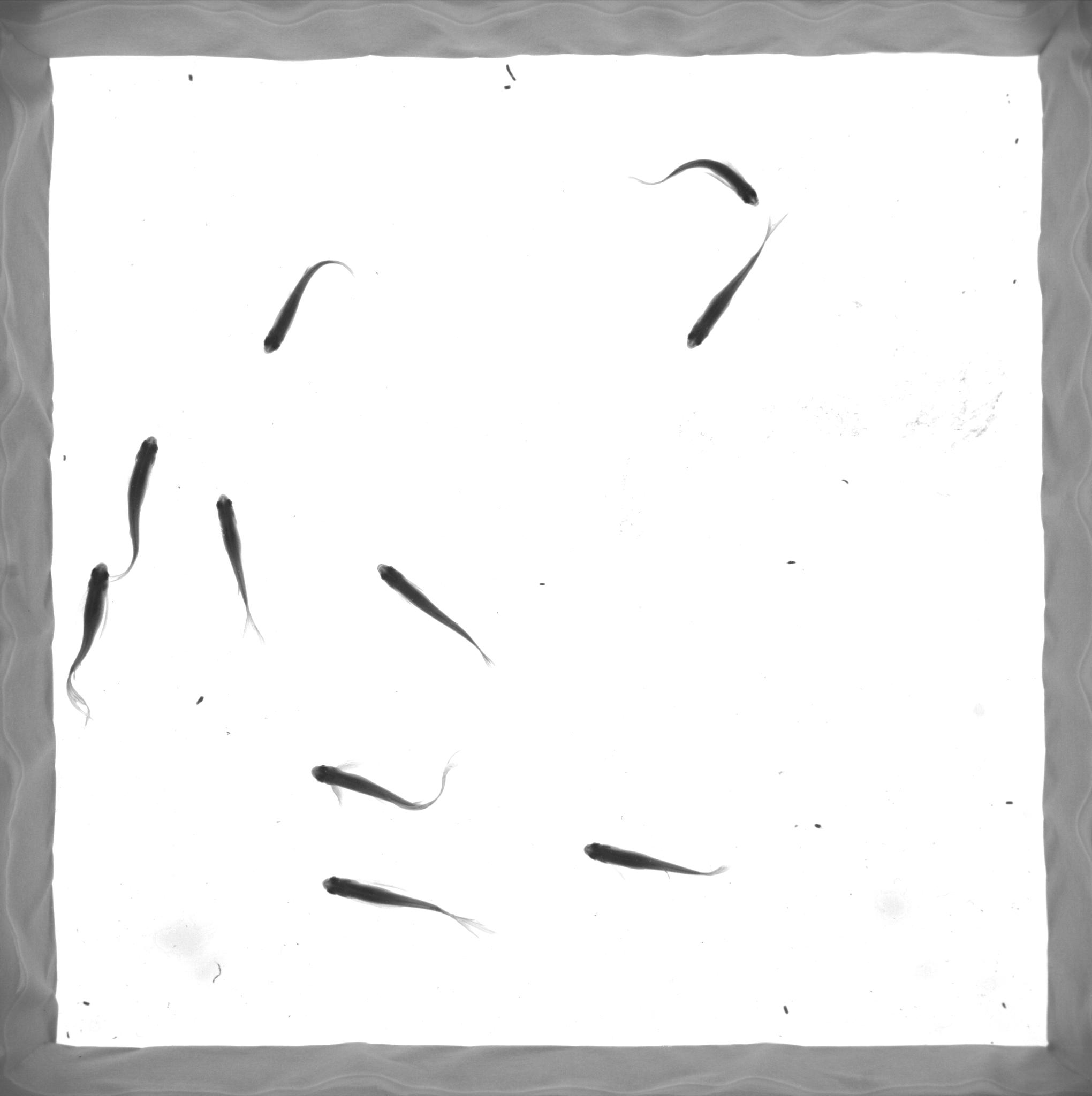

Supplement: S1 File — Source code of the proposed tracking system. (ZIP) [file pone.0154714.s002.zip › code_final/images/CoreView_275_Master_Camera_00066.jpg]

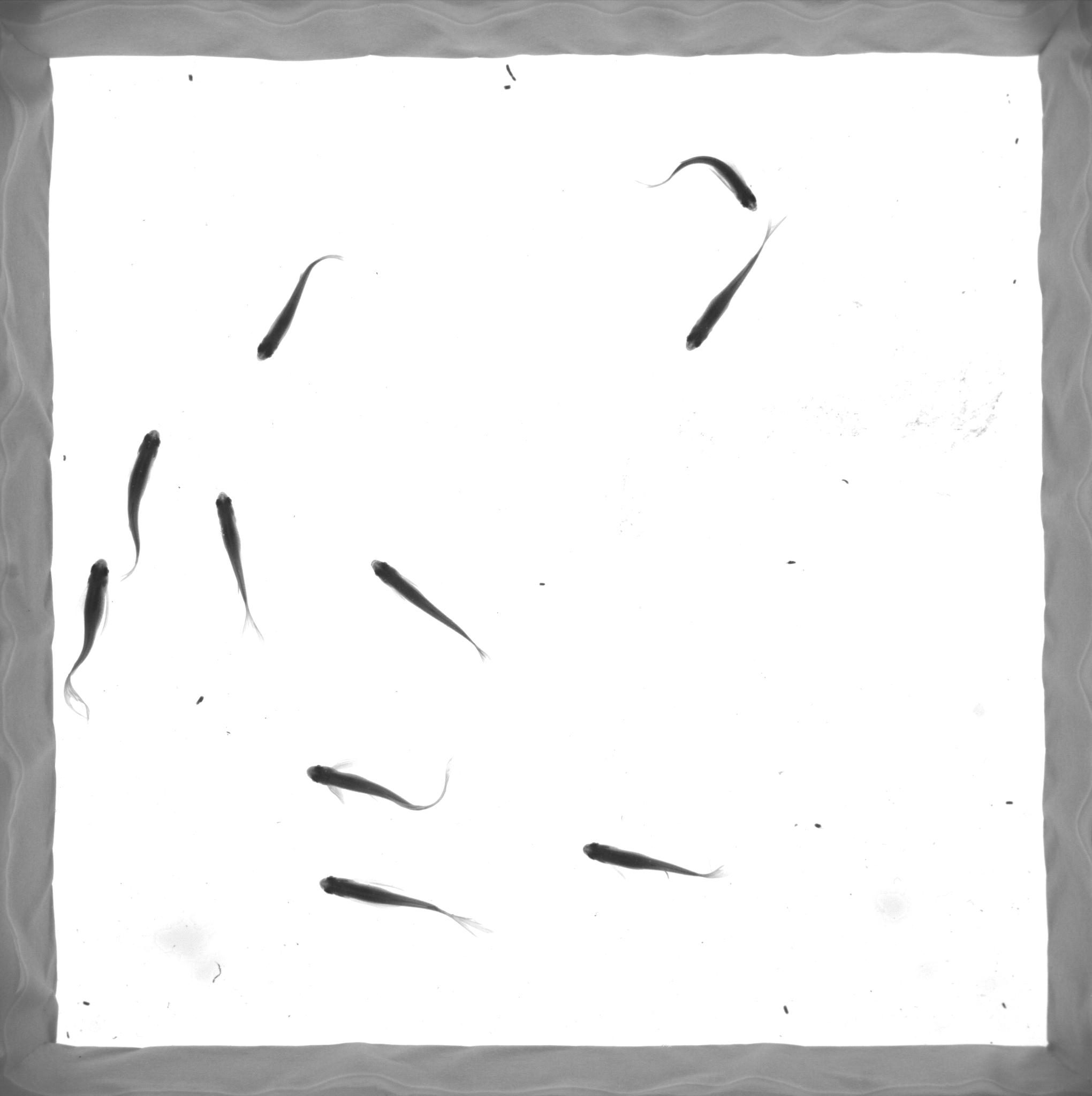

Supplement: S1 File — Source code of the proposed tracking system. (ZIP) [file pone.0154714.s002.zip › code_final/images/CoreView_275_Master_Camera_00067.jpg]

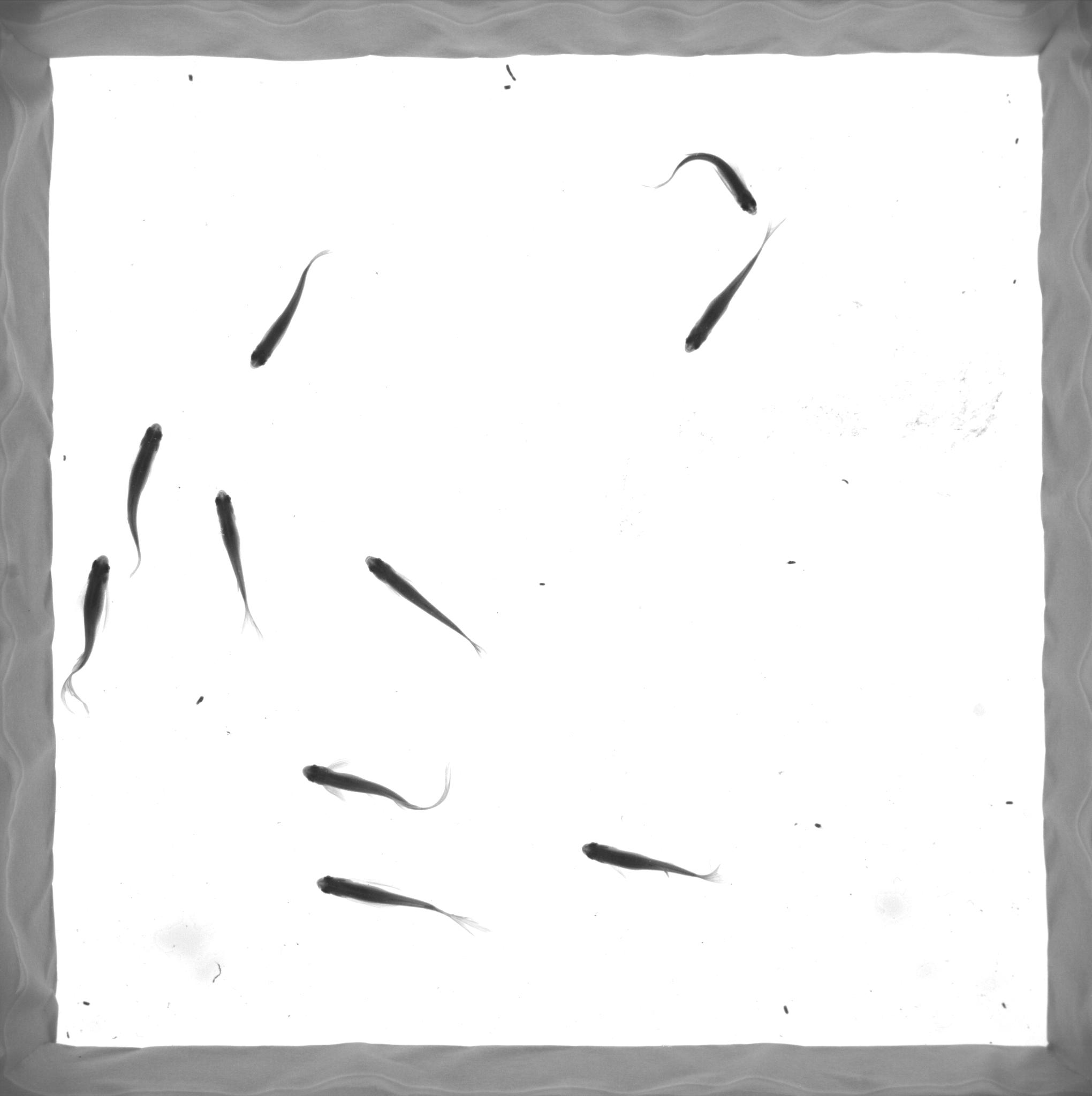

Supplement: S1 File — Source code of the proposed tracking system. (ZIP) [file pone.0154714.s002.zip › code_final/images/CoreView_275_Master_Camera_00068.jpg]

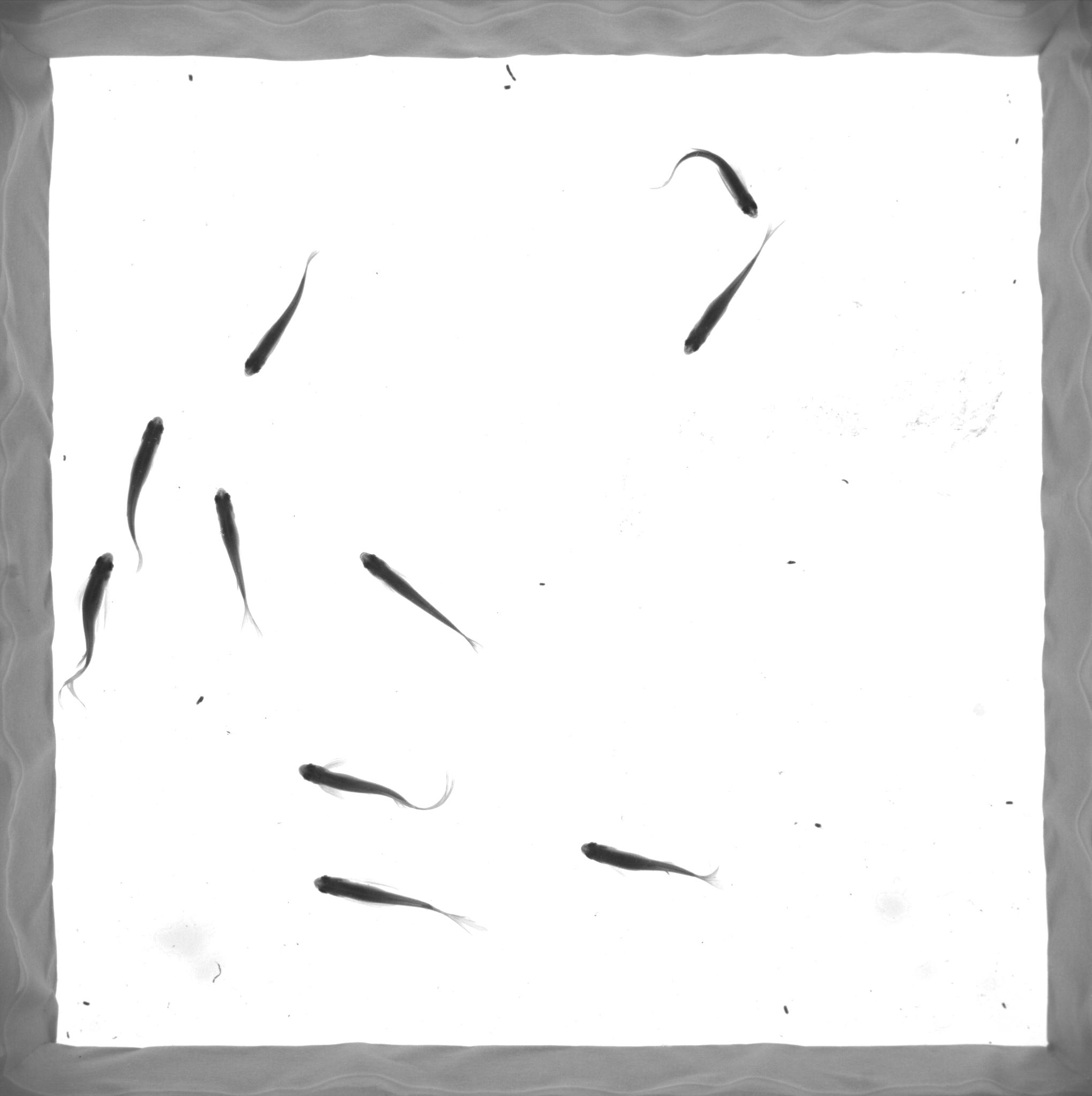

Supplement: S1 File — Source code of the proposed tracking system. (ZIP) [file pone.0154714.s002.zip › code_final/images/CoreView_275_Master_Camera_00069.jpg]

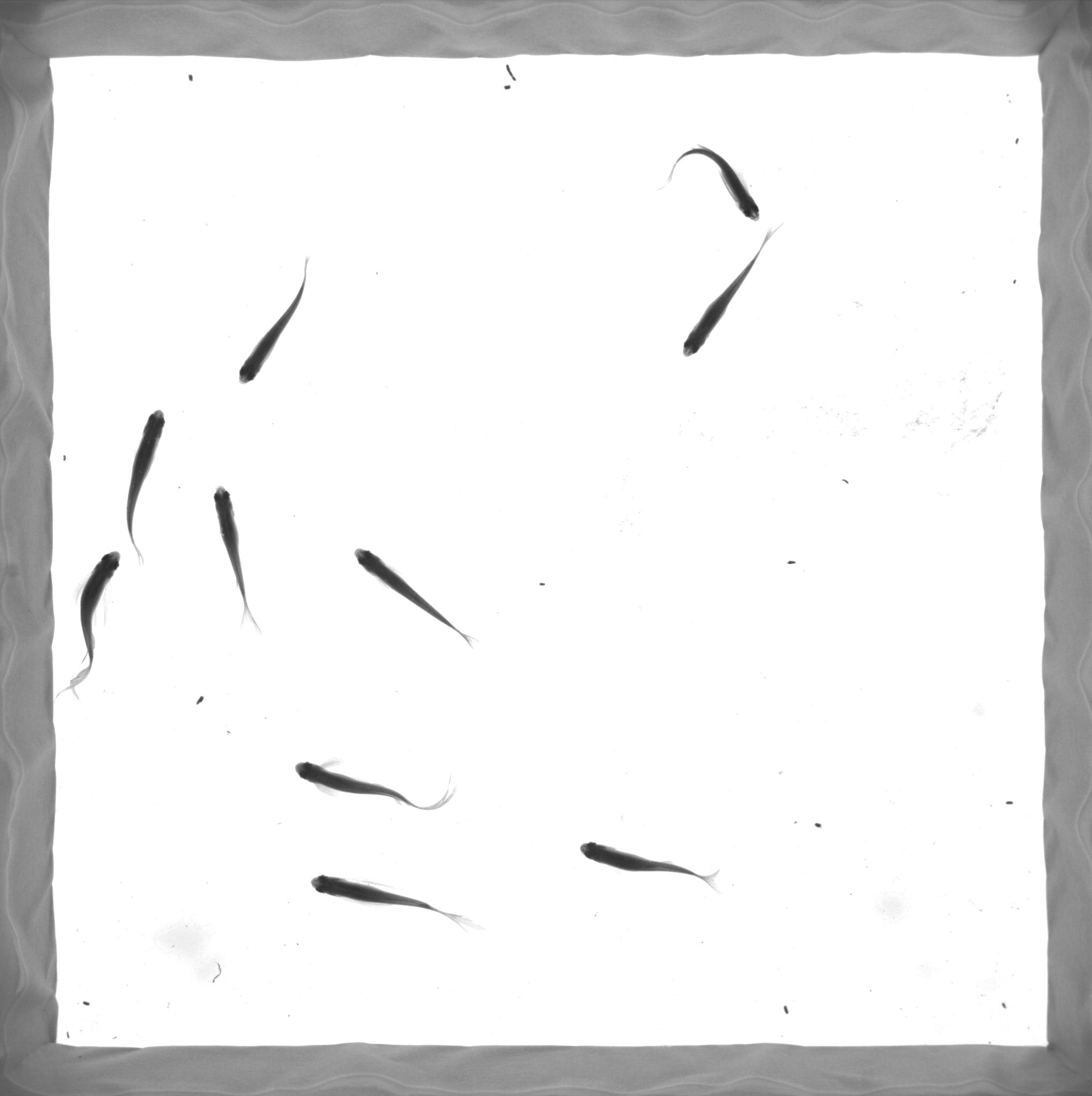

Supplement: S1 File — Source code of the proposed tracking system. (ZIP) [file pone.0154714.s002.zip › code_final/images/CoreView_275_Master_Camera_00070.jpg]

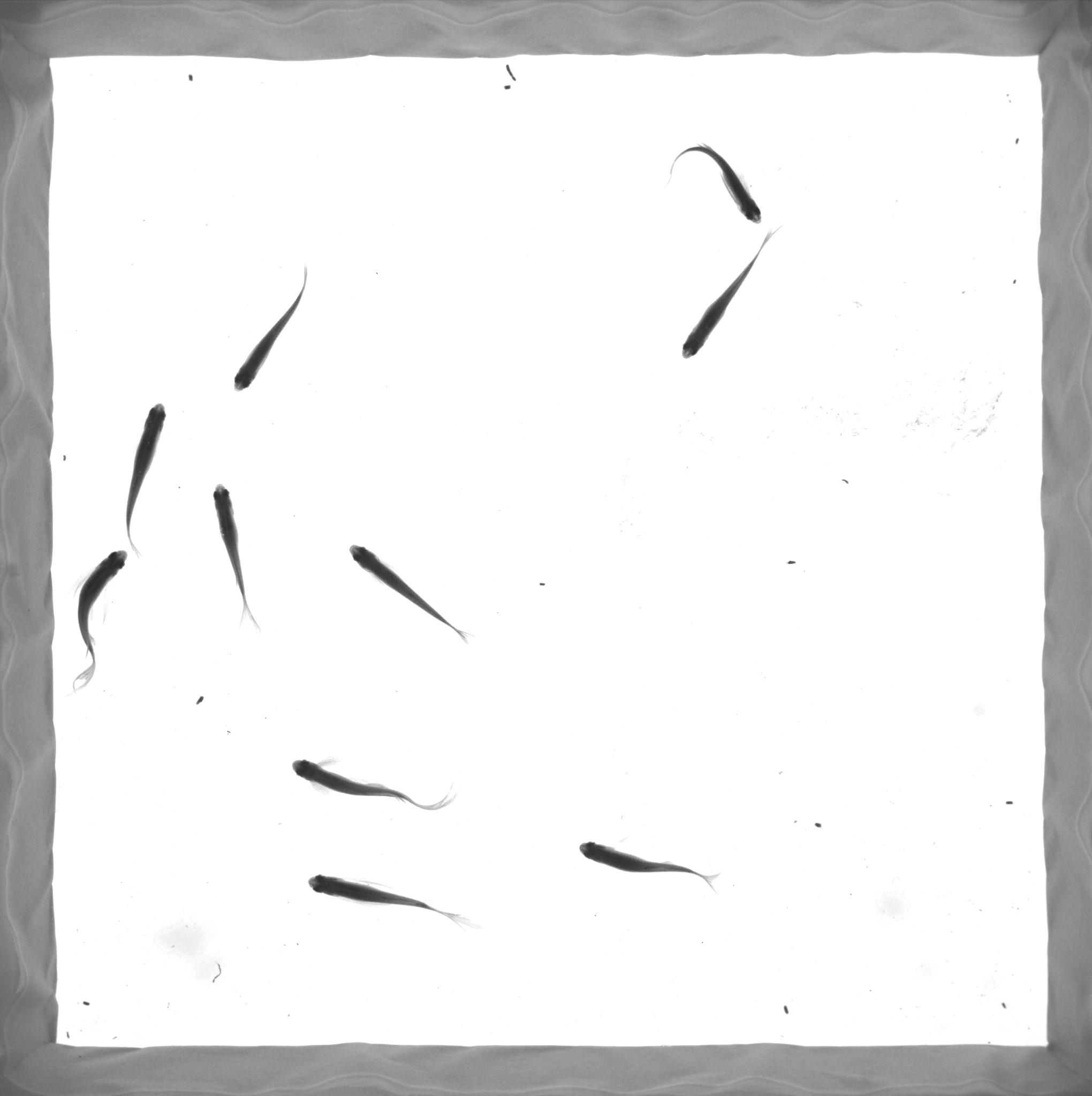

Supplement: S1 File — Source code of the proposed tracking system. (ZIP) [file pone.0154714.s002.zip › code_final/images/CoreView_275_Master_Camera_00071.jpg]

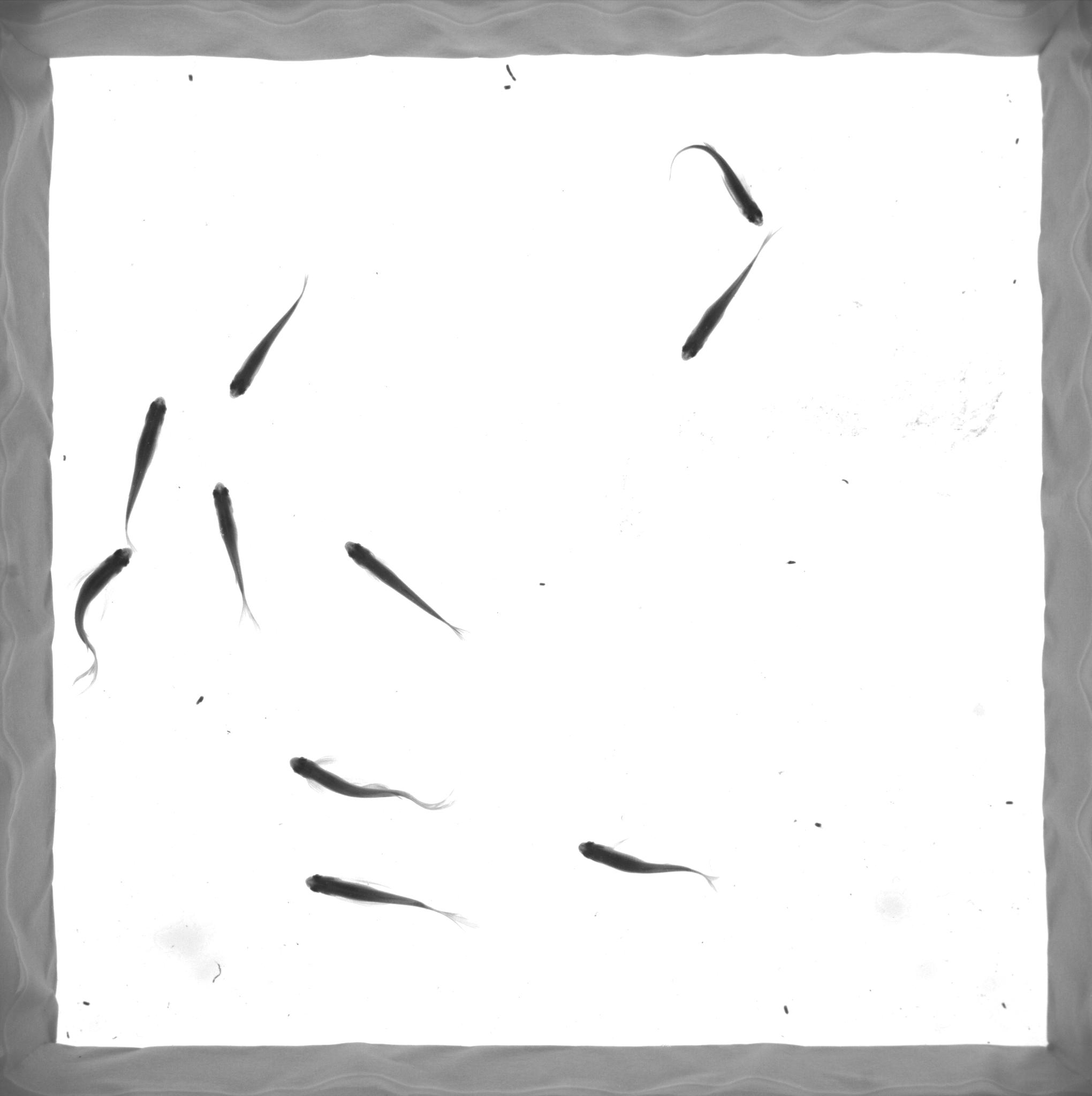

Supplement: S1 File — Source code of the proposed tracking system. (ZIP) [file pone.0154714.s002.zip › code_final/images/CoreView_275_Master_Camera_00072.jpg]

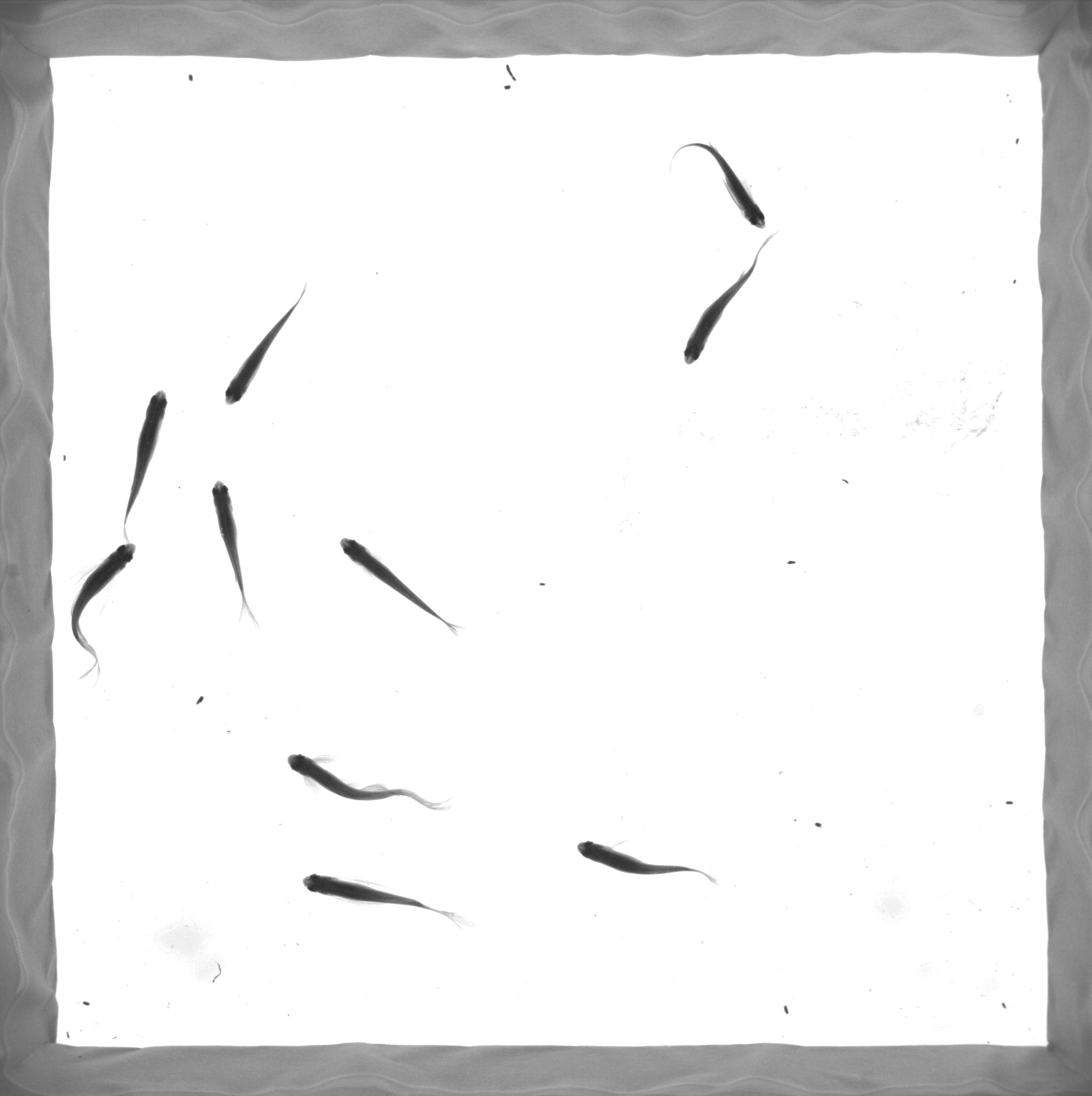

Supplement: S1 File — Source code of the proposed tracking system. (ZIP) [file pone.0154714.s002.zip › code_final/images/CoreView_275_Master_Camera_00073.jpg]

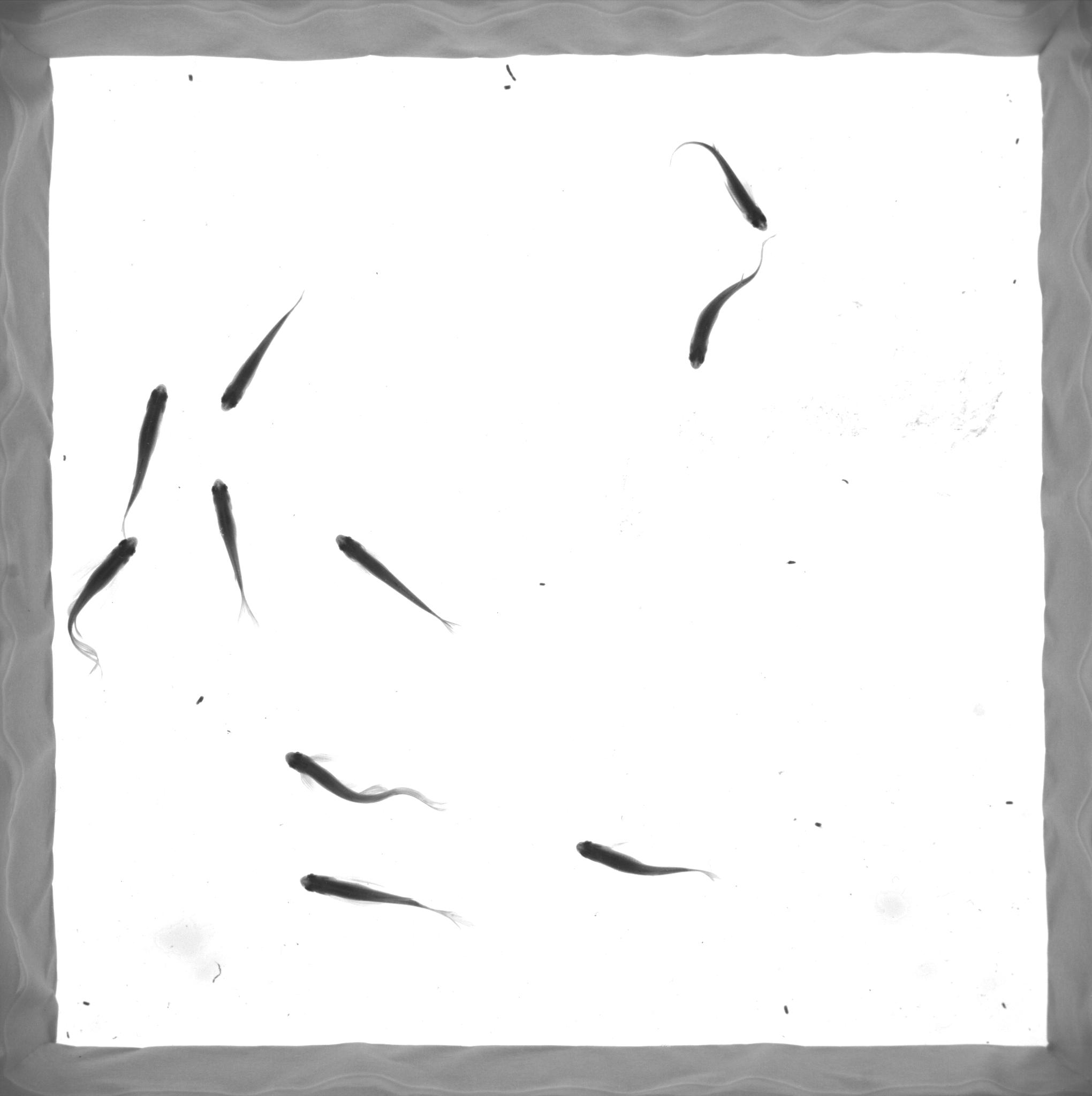

Supplement: S1 File — Source code of the proposed tracking system. (ZIP) [file pone.0154714.s002.zip › code_final/images/CoreView_275_Master_Camera_00074.jpg]

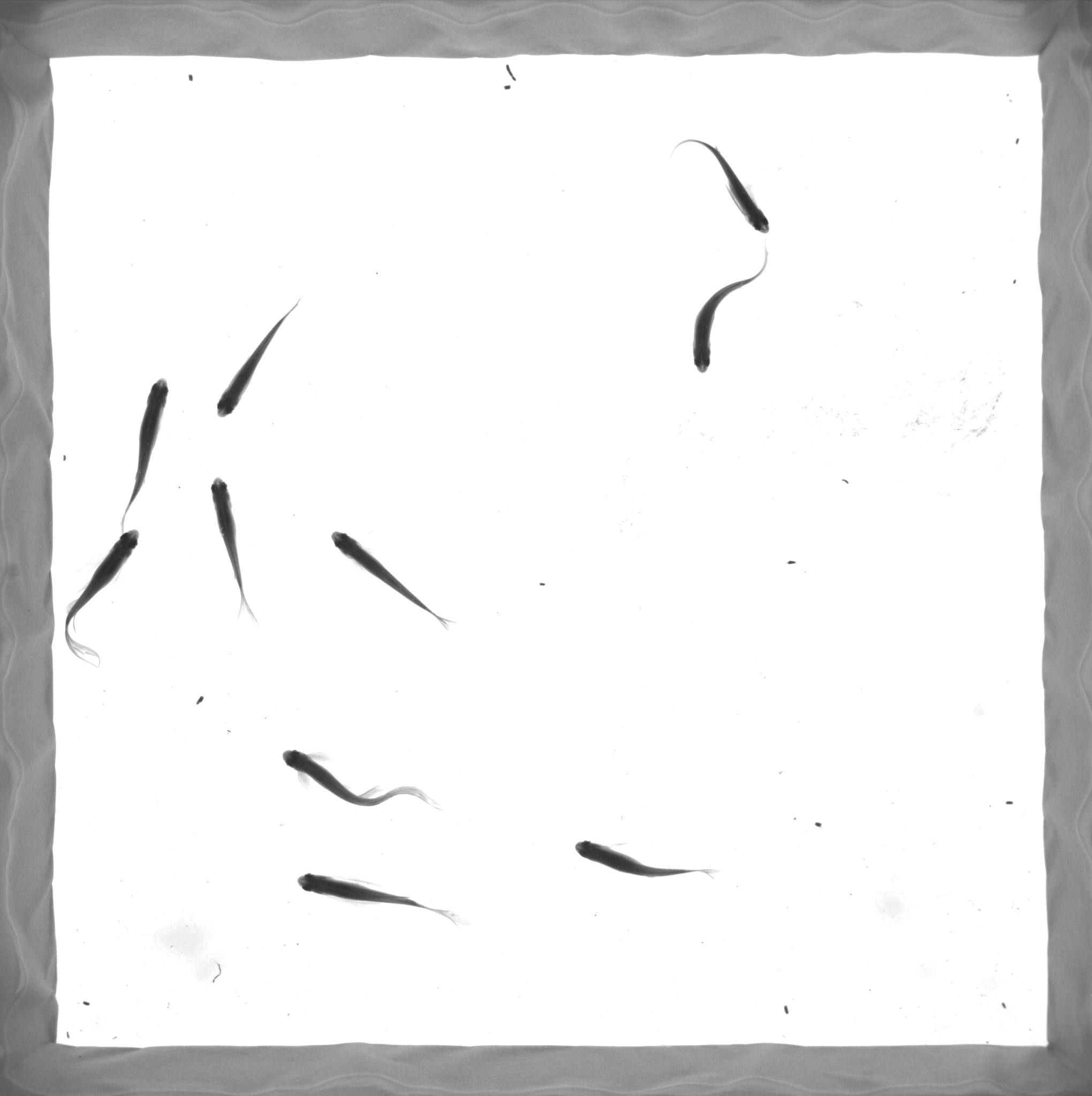

Supplement: S1 File — Source code of the proposed tracking system. (ZIP) [file pone.0154714.s002.zip › code_final/images/CoreView_275_Master_Camera_00075.jpg]

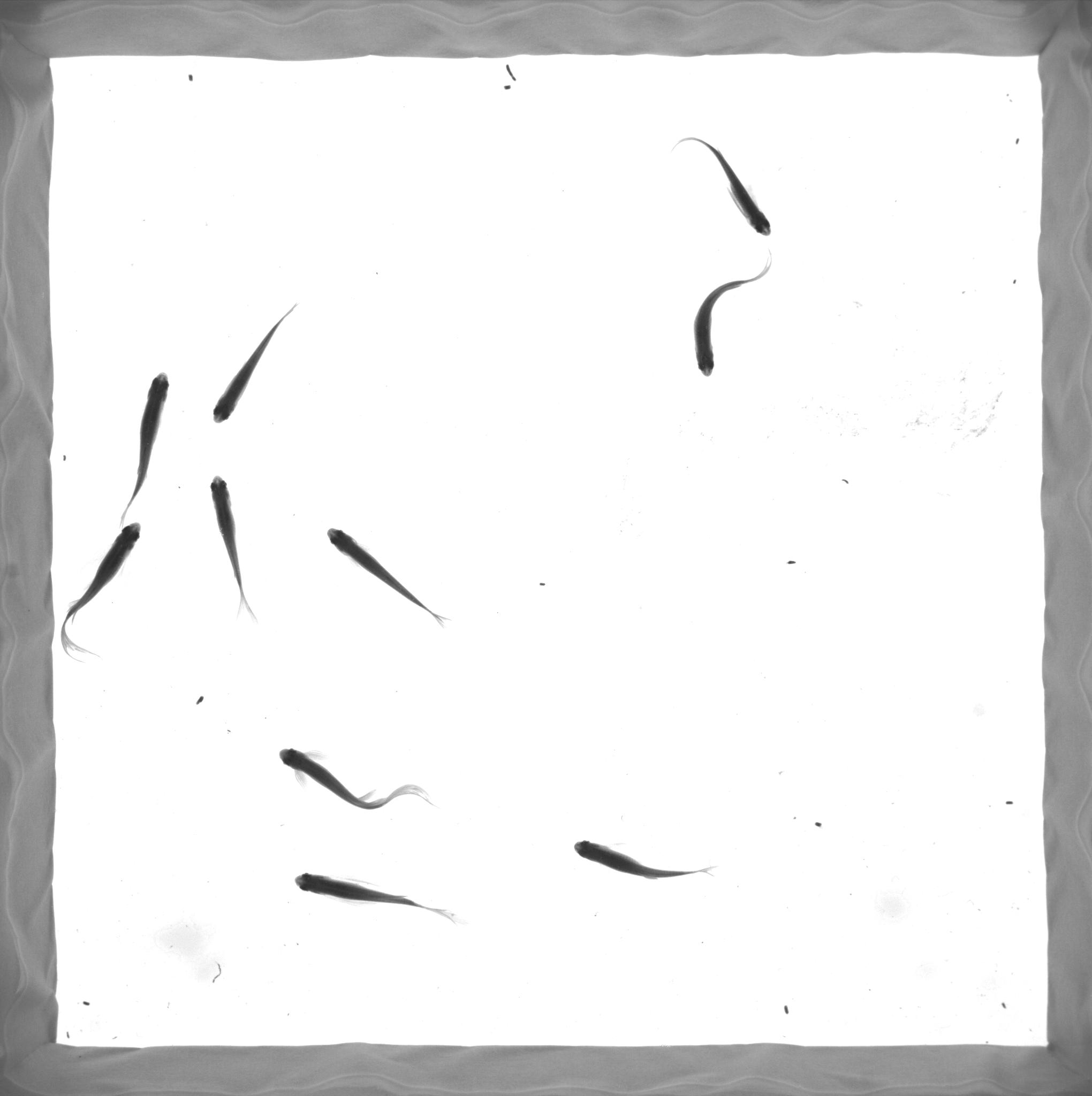

Supplement: S1 File — Source code of the proposed tracking system. (ZIP) [file pone.0154714.s002.zip › code_final/images/CoreView_275_Master_Camera_00076.jpg]

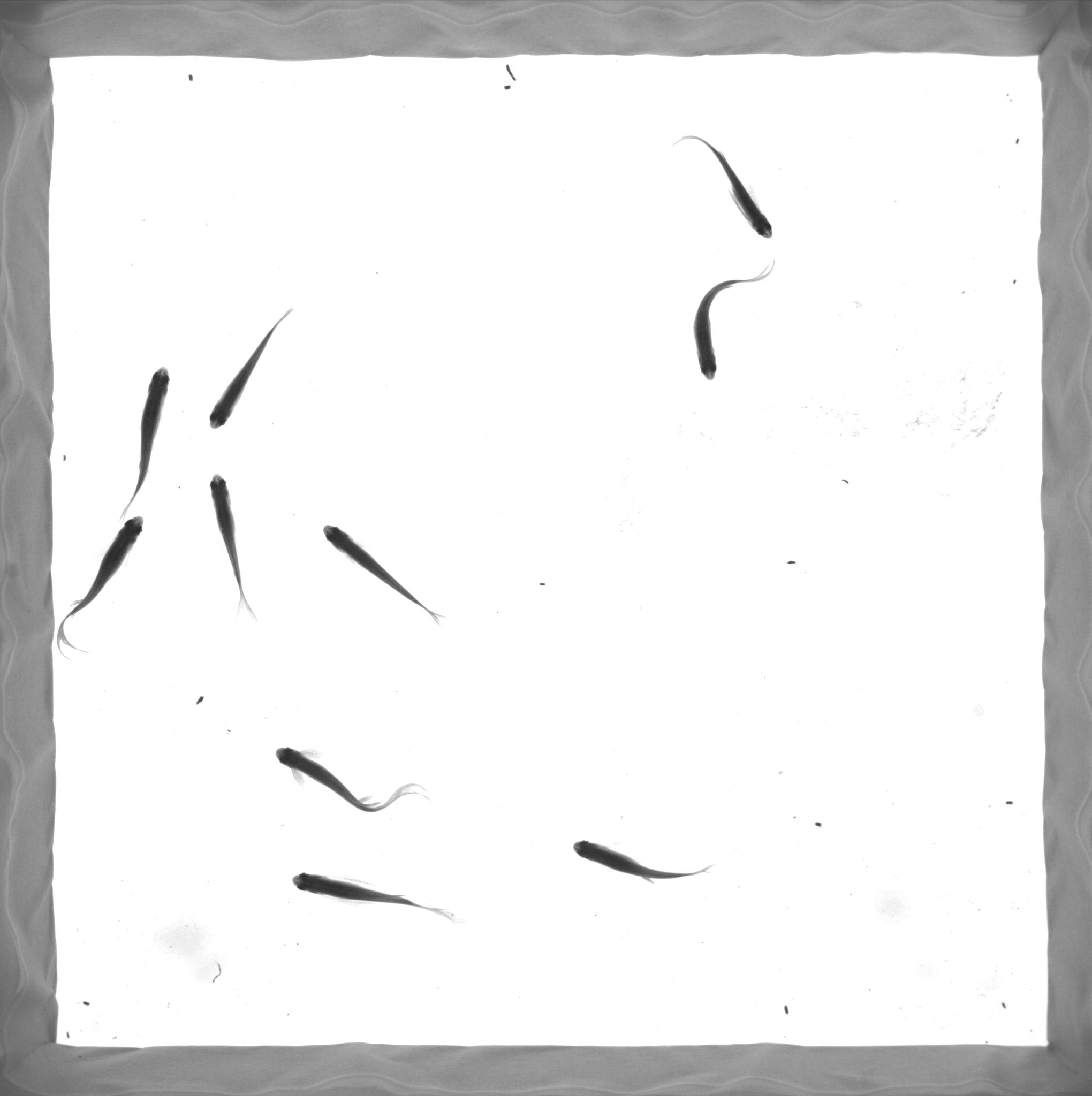

Supplement: S1 File — Source code of the proposed tracking system. (ZIP) [file pone.0154714.s002.zip › code_final/images/CoreView_275_Master_Camera_00077.jpg]

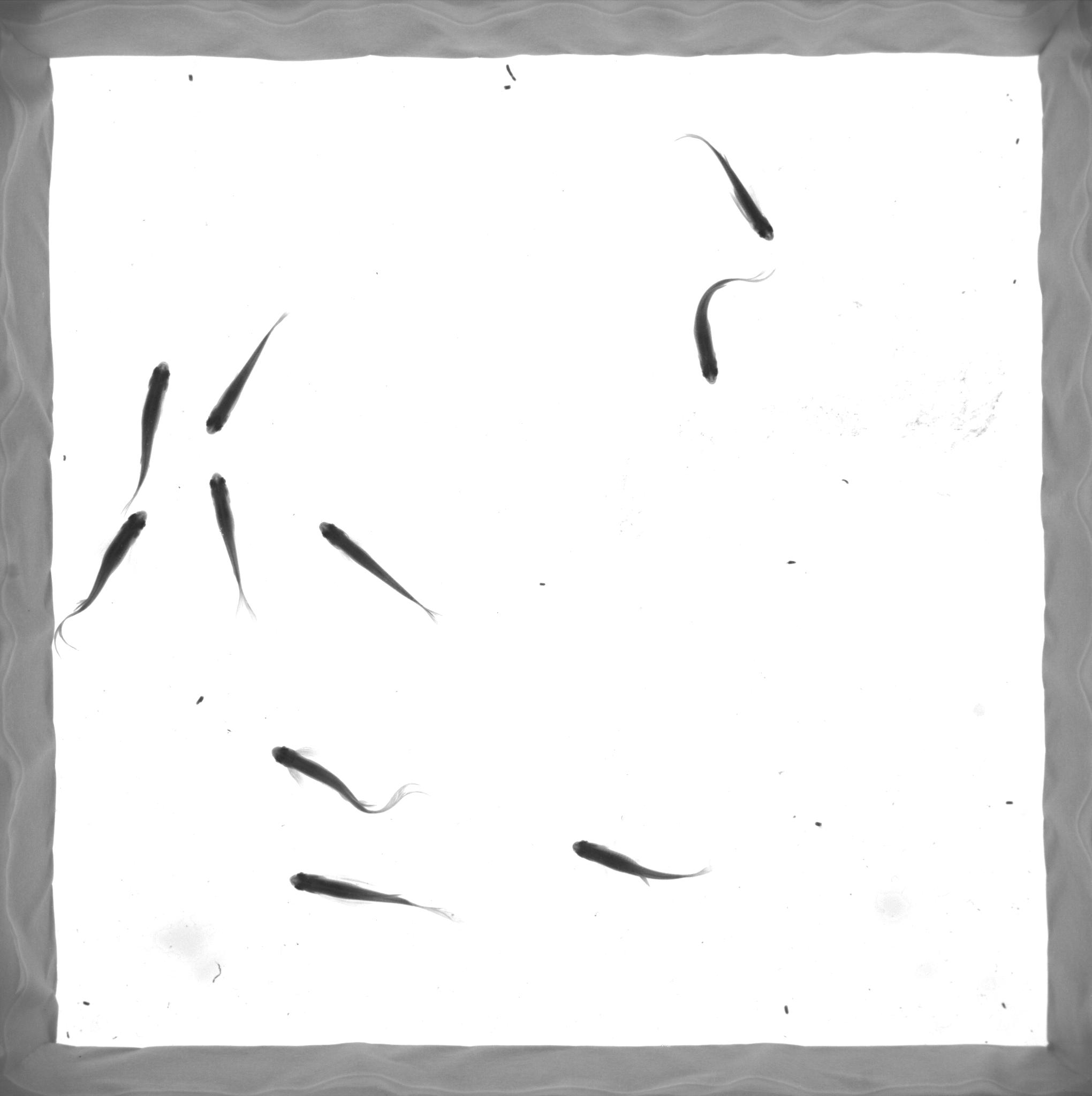

Supplement: S1 File — Source code of the proposed tracking system. (ZIP) [file pone.0154714.s002.zip › code_final/images/CoreView_275_Master_Camera_00078.jpg]

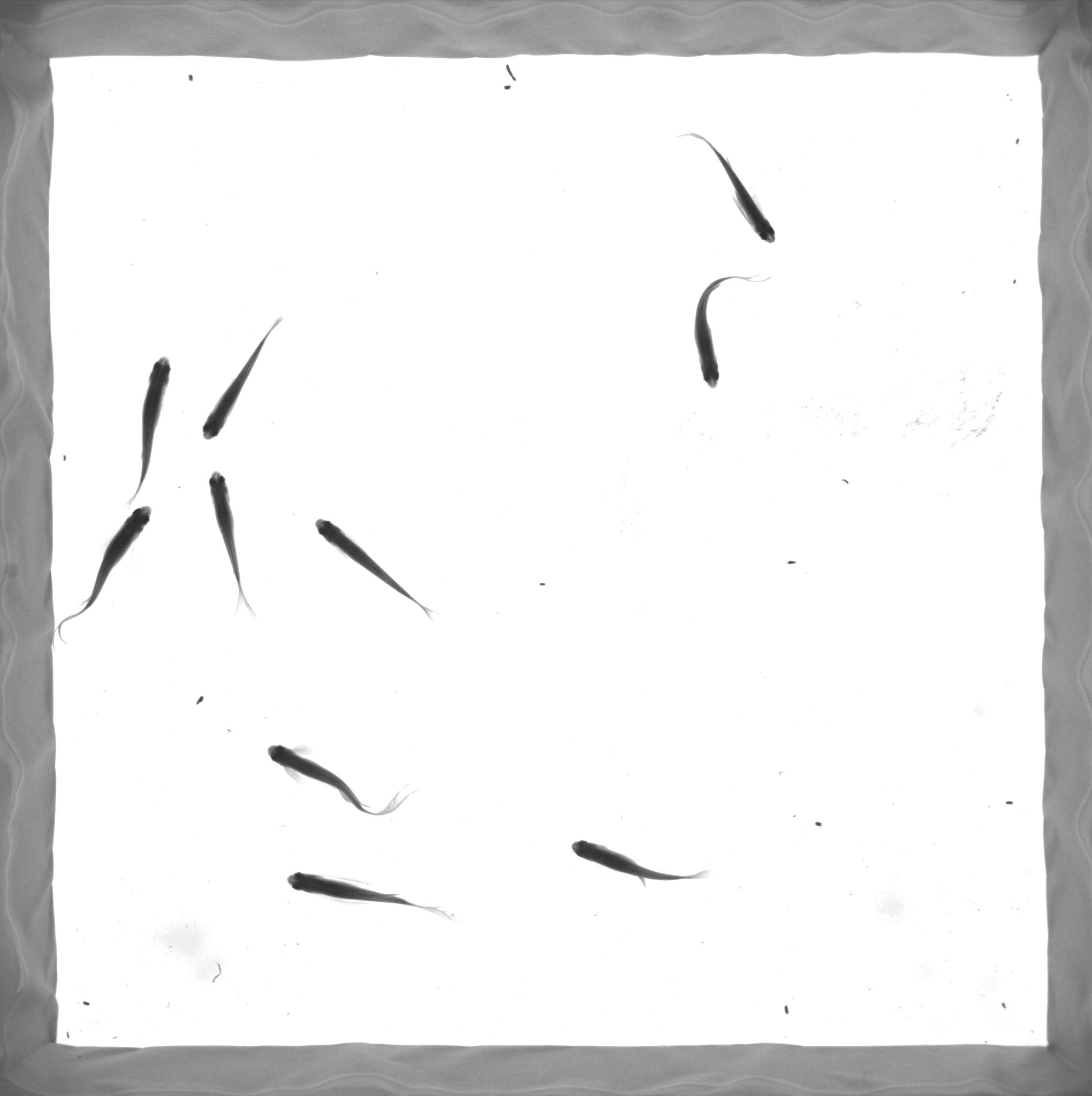

Supplement: S1 File — Source code of the proposed tracking system. (ZIP) [file pone.0154714.s002.zip › code_final/images/CoreView_275_Master_Camera_00079.jpg]

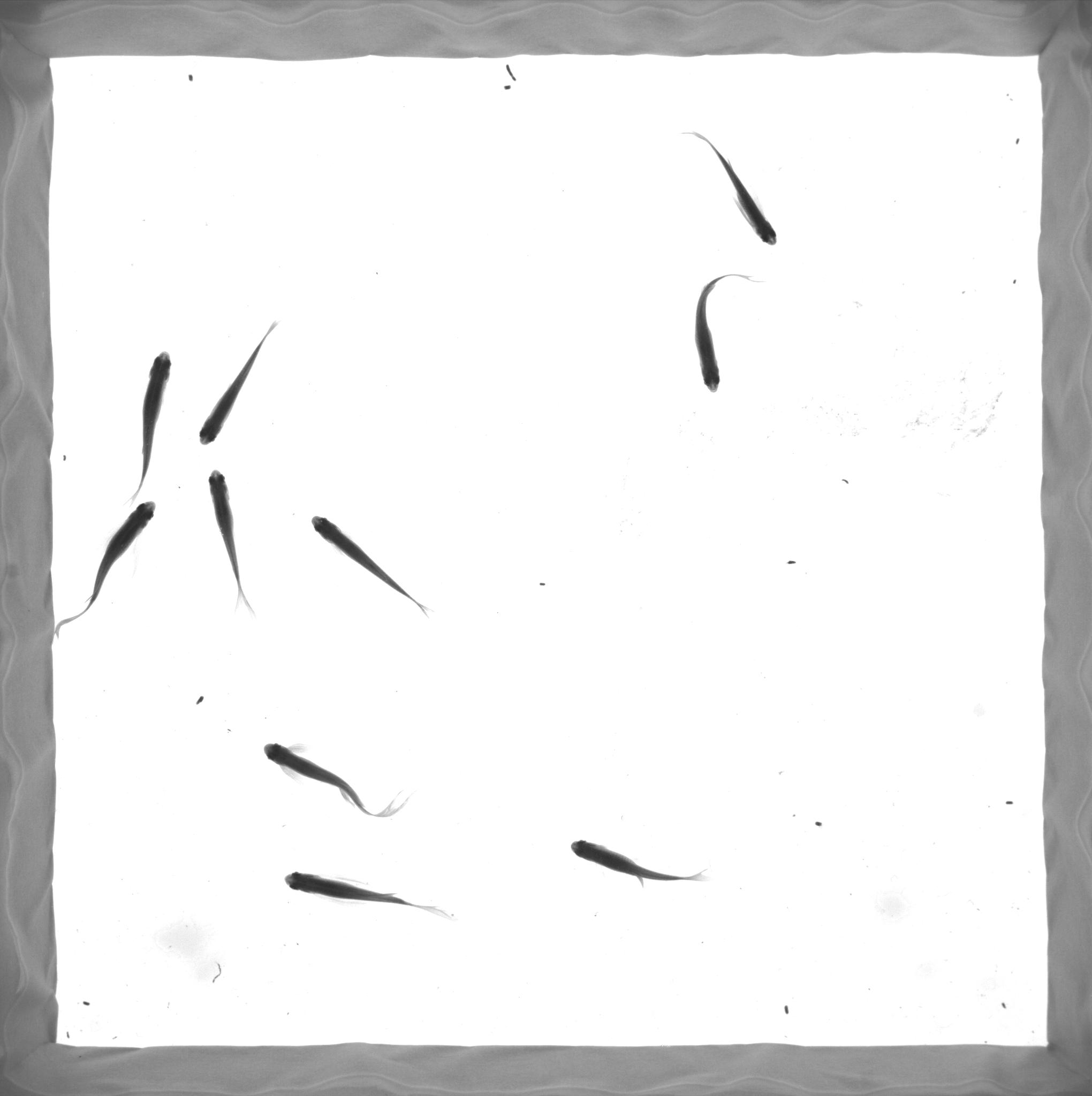

Supplement: S1 File — Source code of the proposed tracking system. (ZIP) [file pone.0154714.s002.zip › code_final/images/CoreView_275_Master_Camera_00080.jpg]

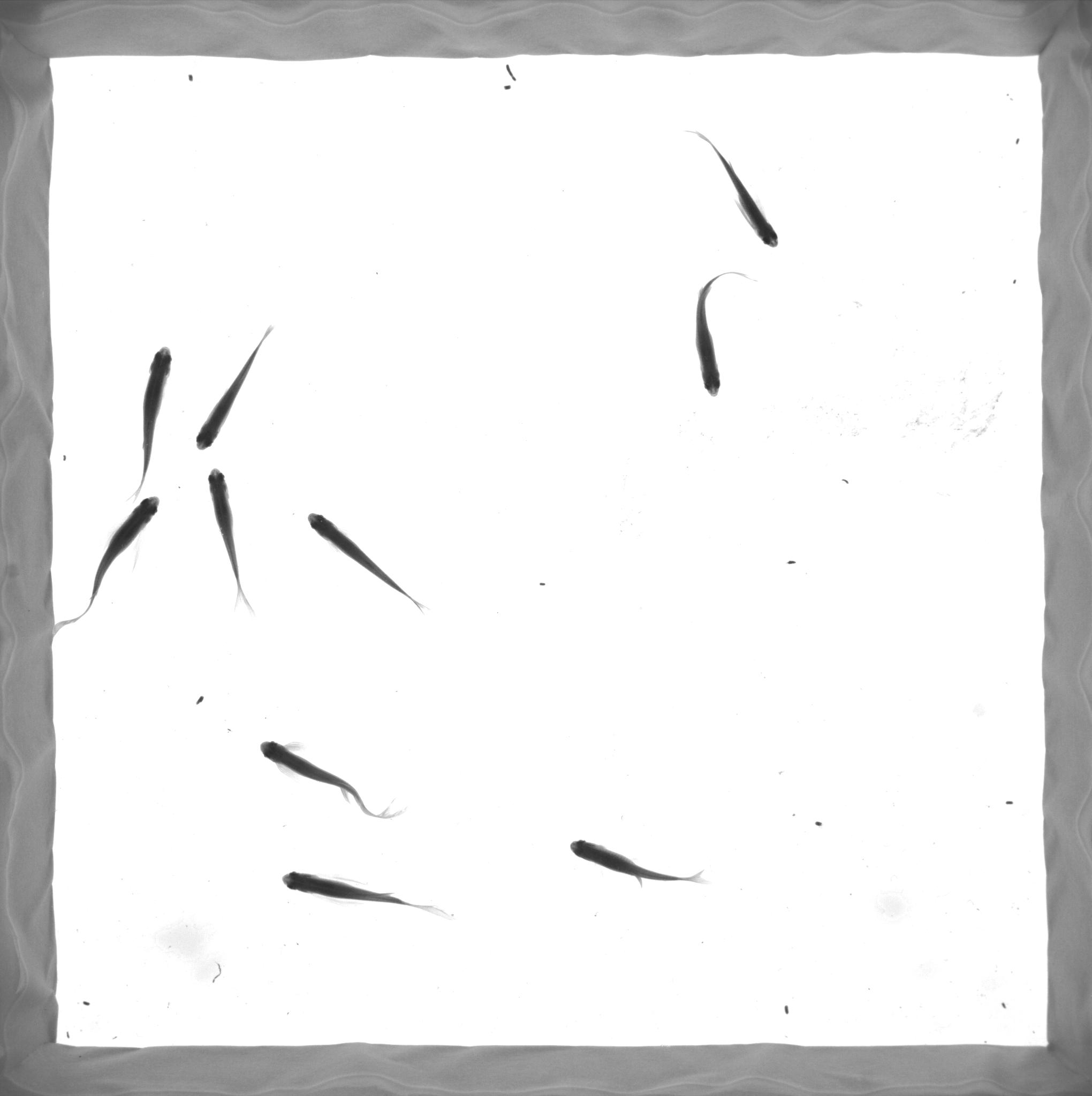

Supplement: S1 File — Source code of the proposed tracking system. (ZIP) [file pone.0154714.s002.zip › code_final/images/CoreView_275_Master_Camera_00081.jpg]

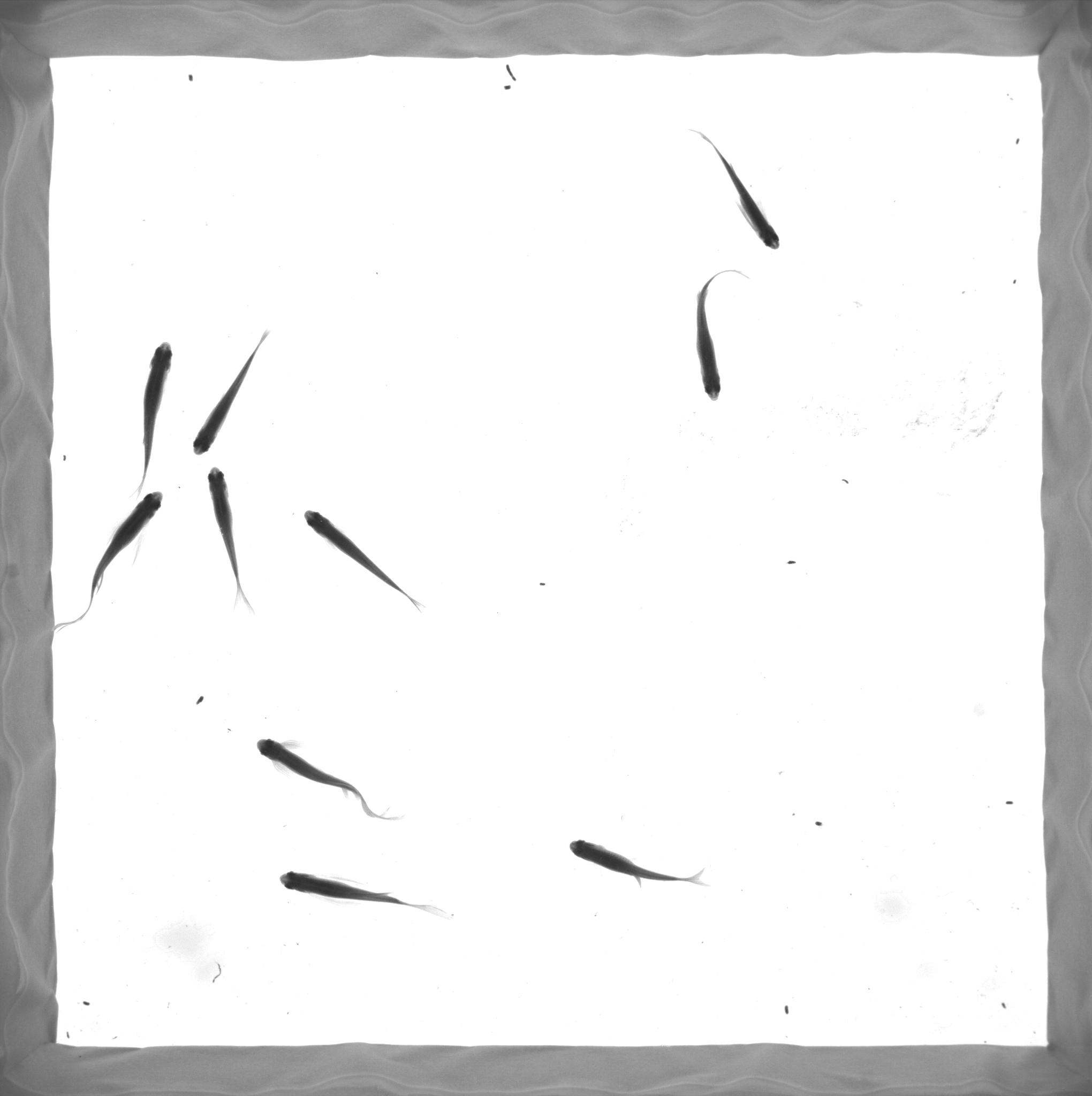

Supplement: S1 File — Source code of the proposed tracking system. (ZIP) [file pone.0154714.s002.zip › code_final/images/CoreView_275_Master_Camera_00082.jpg]

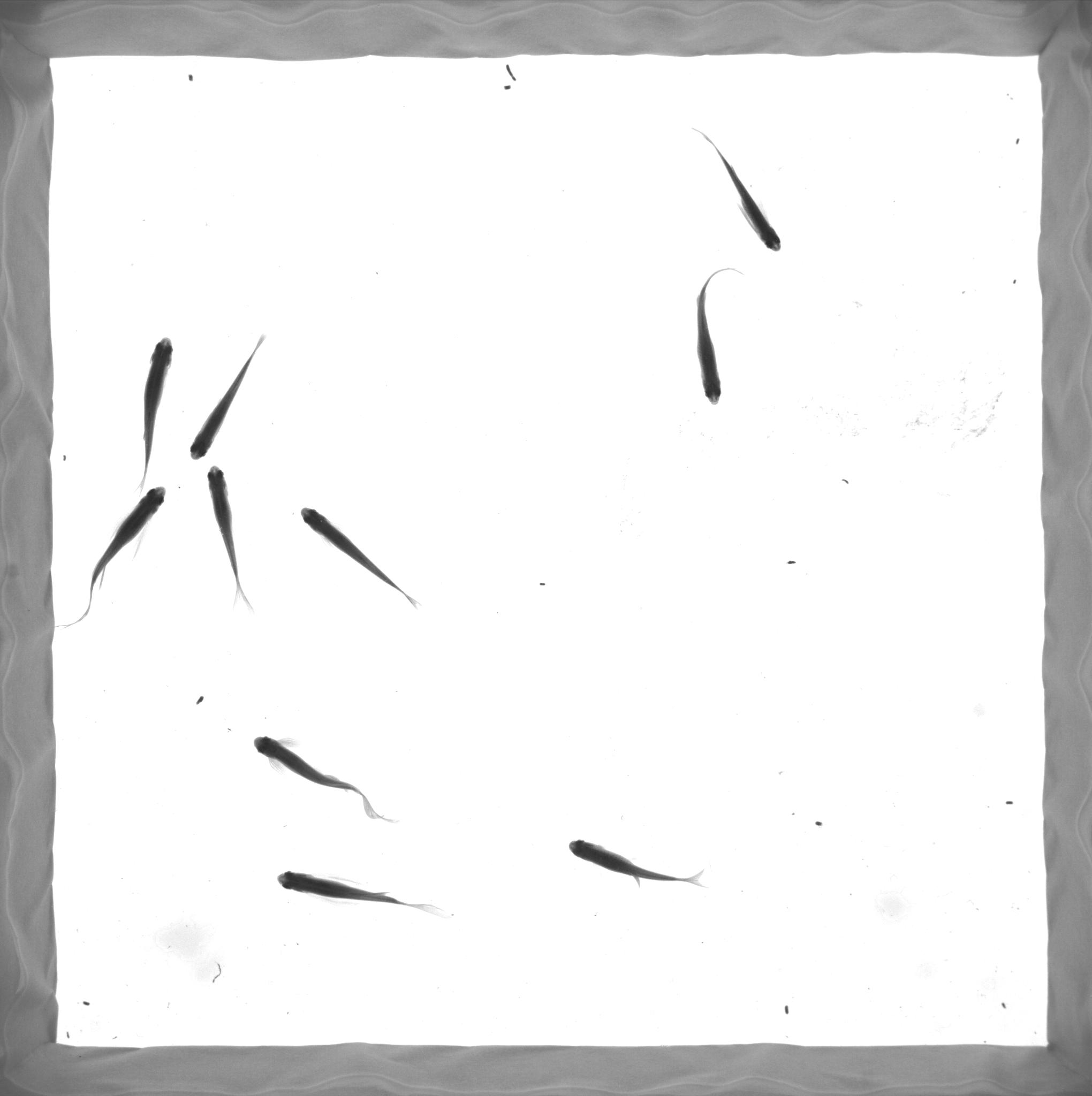

Supplement: S1 File — Source code of the proposed tracking system. (ZIP) [file pone.0154714.s002.zip › code_final/images/CoreView_275_Master_Camera_00083.jpg]

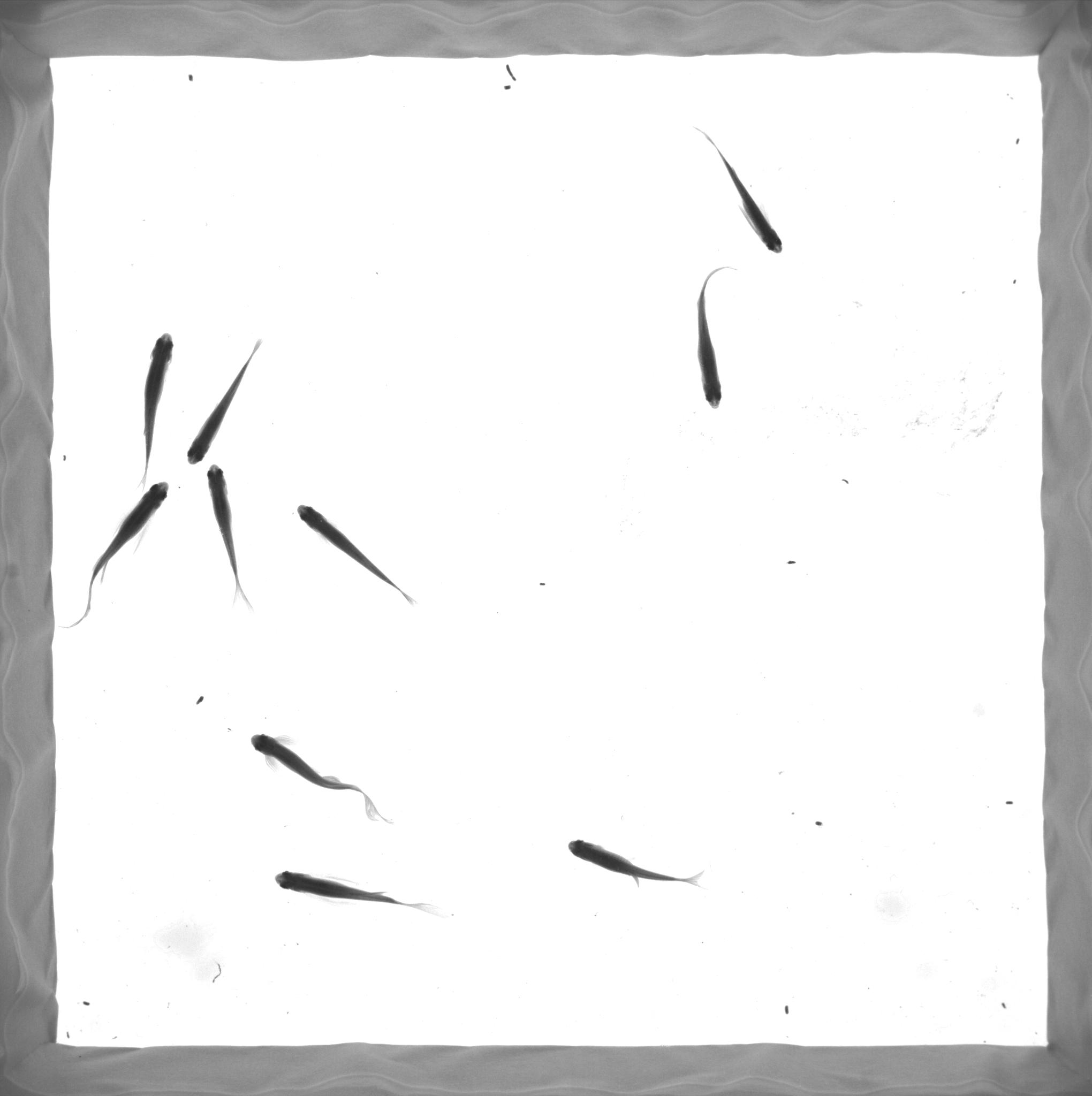

Supplement: S1 File — Source code of the proposed tracking system. (ZIP) [file pone.0154714.s002.zip › code_final/images/CoreView_275_Master_Camera_00084.jpg]

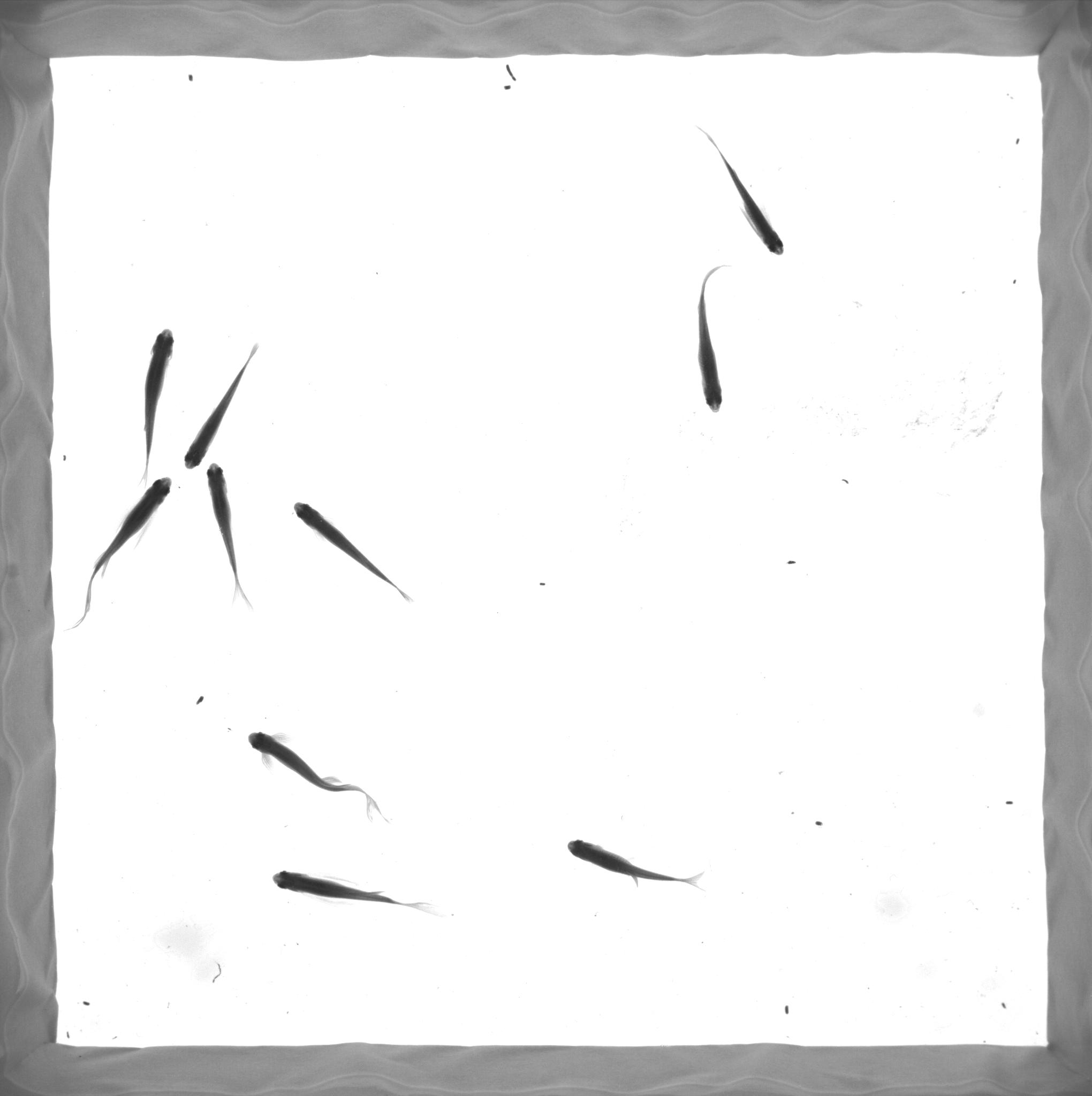

Supplement: S1 File — Source code of the proposed tracking system. (ZIP) [file pone.0154714.s002.zip › code_final/images/CoreView_275_Master_Camera_00085.jpg]

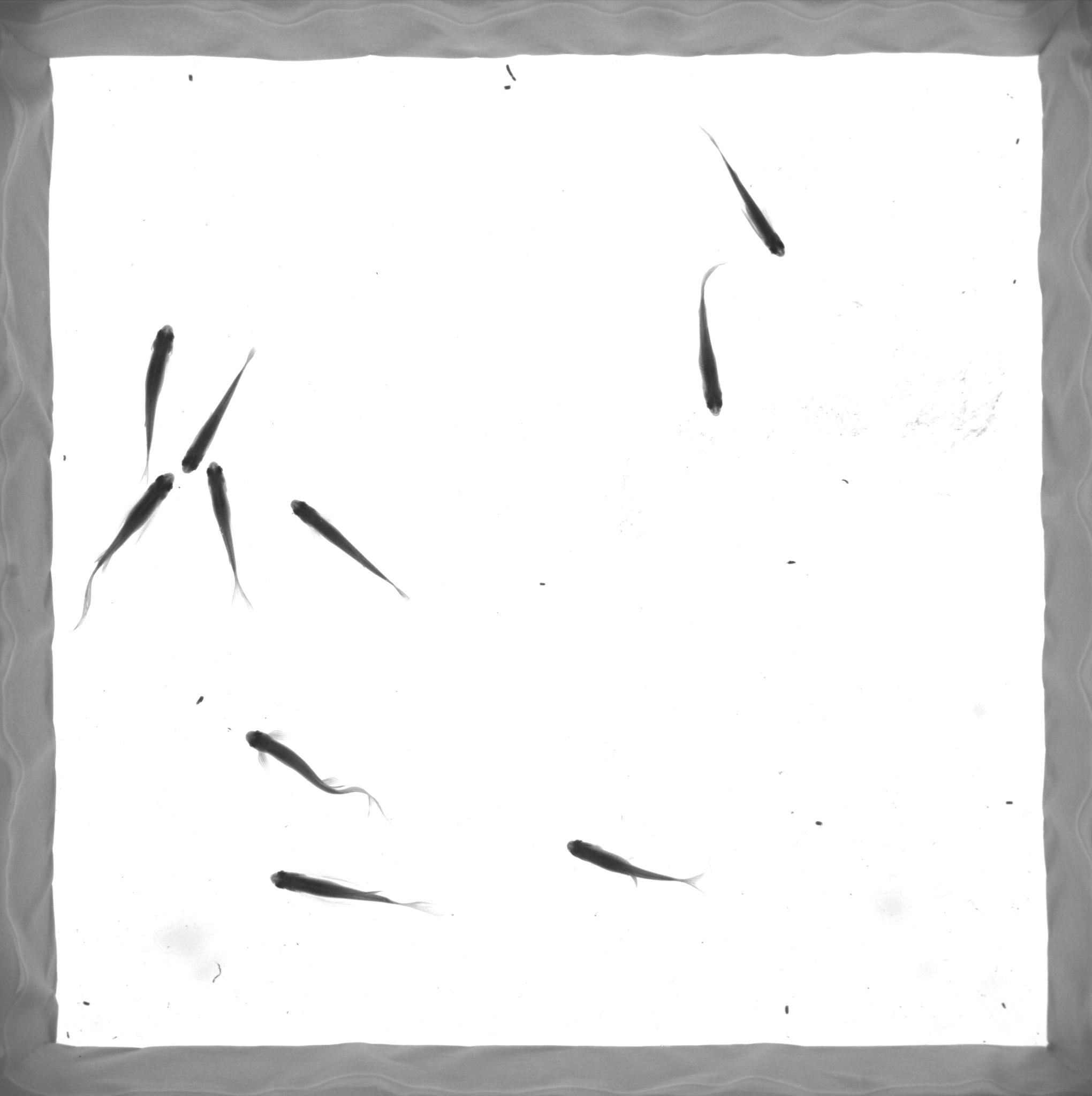

Supplement: S1 File — Source code of the proposed tracking system. (ZIP) [file pone.0154714.s002.zip › code_final/images/CoreView_275_Master_Camera_00086.jpg]

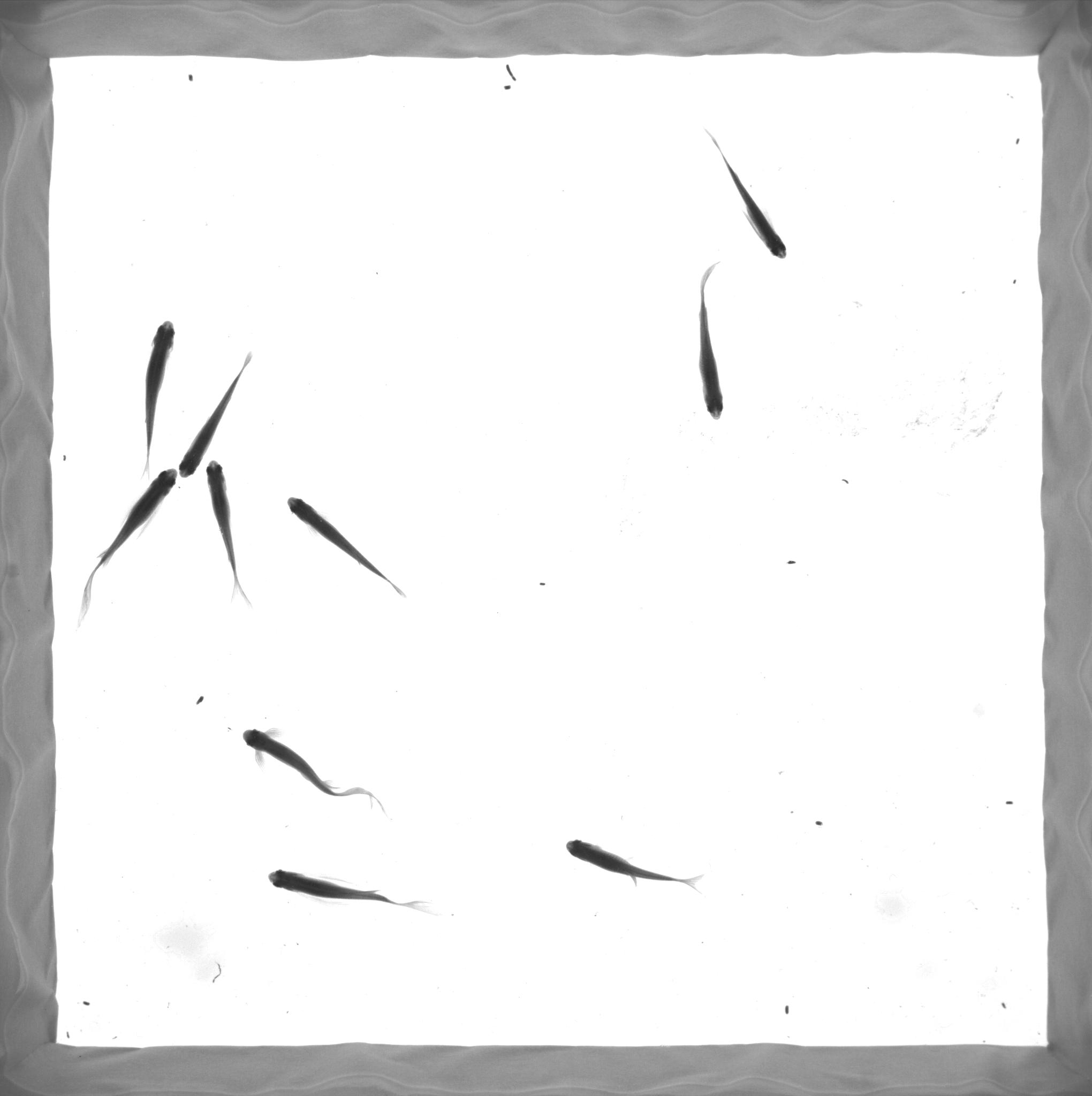

Supplement: S1 File — Source code of the proposed tracking system. (ZIP) [file pone.0154714.s002.zip › code_final/images/CoreView_275_Master_Camera_00087.jpg]

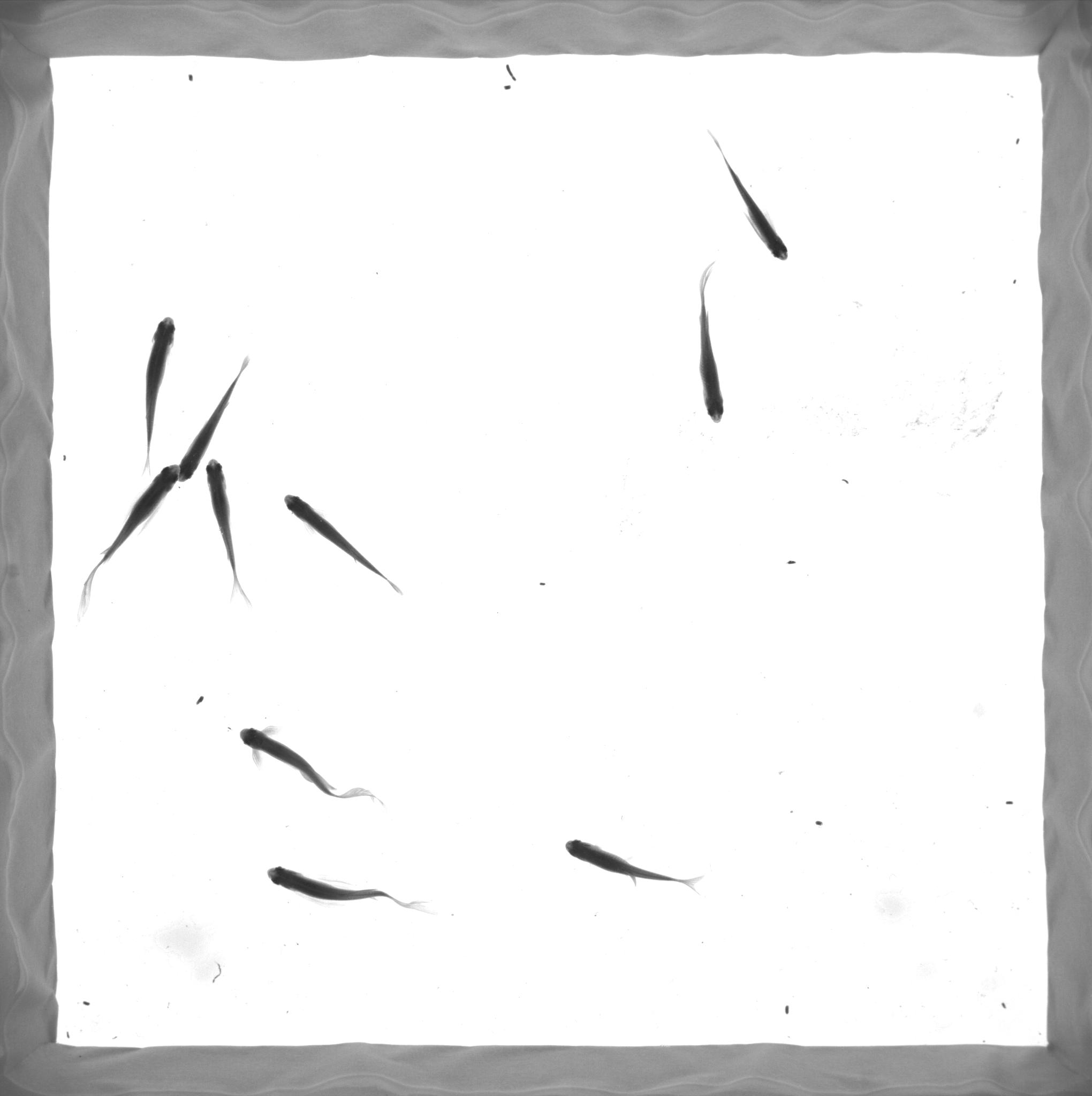

Supplement: S1 File — Source code of the proposed tracking system. (ZIP) [file pone.0154714.s002.zip › code_final/images/CoreView_275_Master_Camera_00088.jpg]

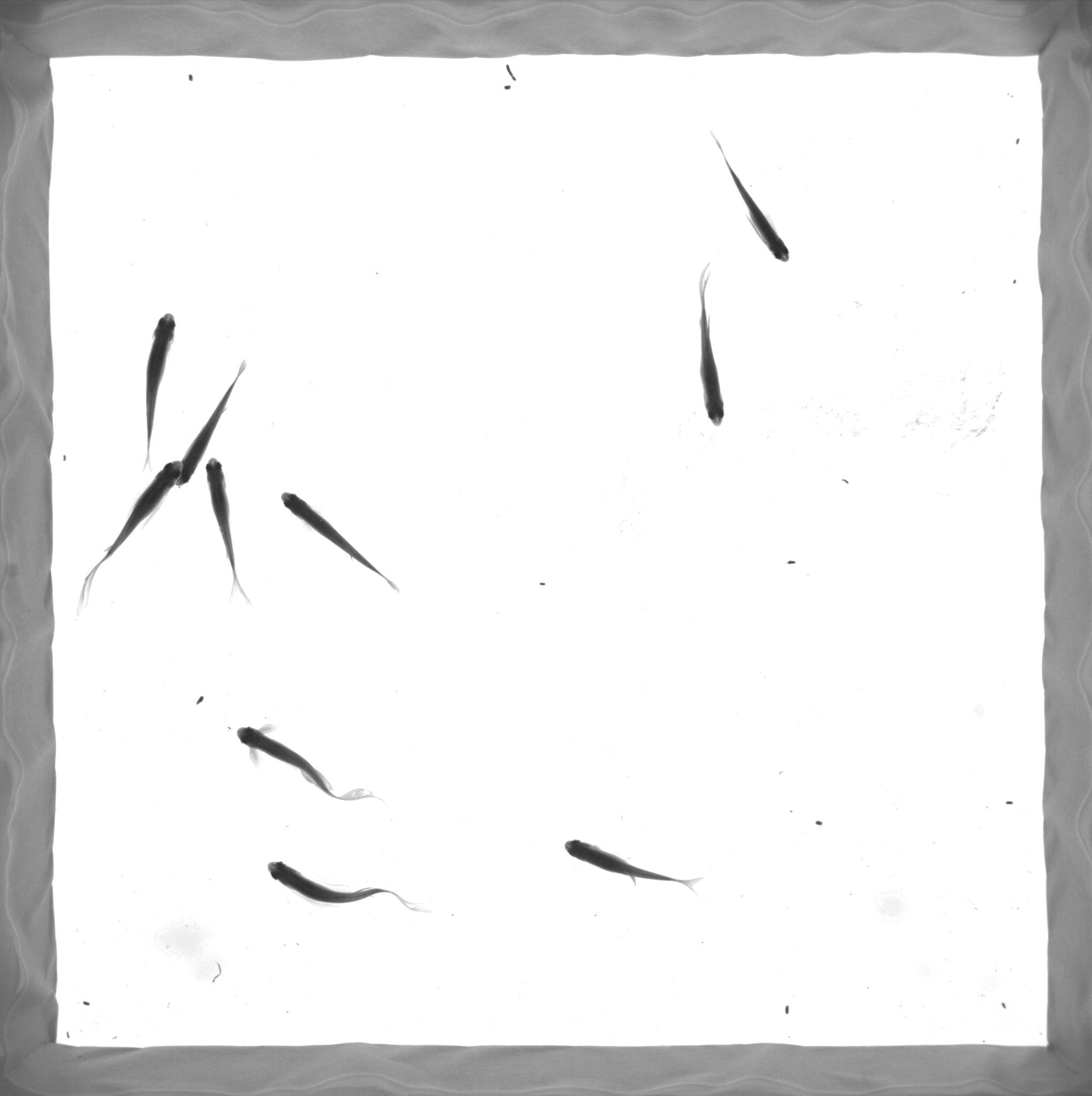

Supplement: S1 File — Source code of the proposed tracking system. (ZIP) [file pone.0154714.s002.zip › code_final/images/CoreView_275_Master_Camera_00089.jpg]

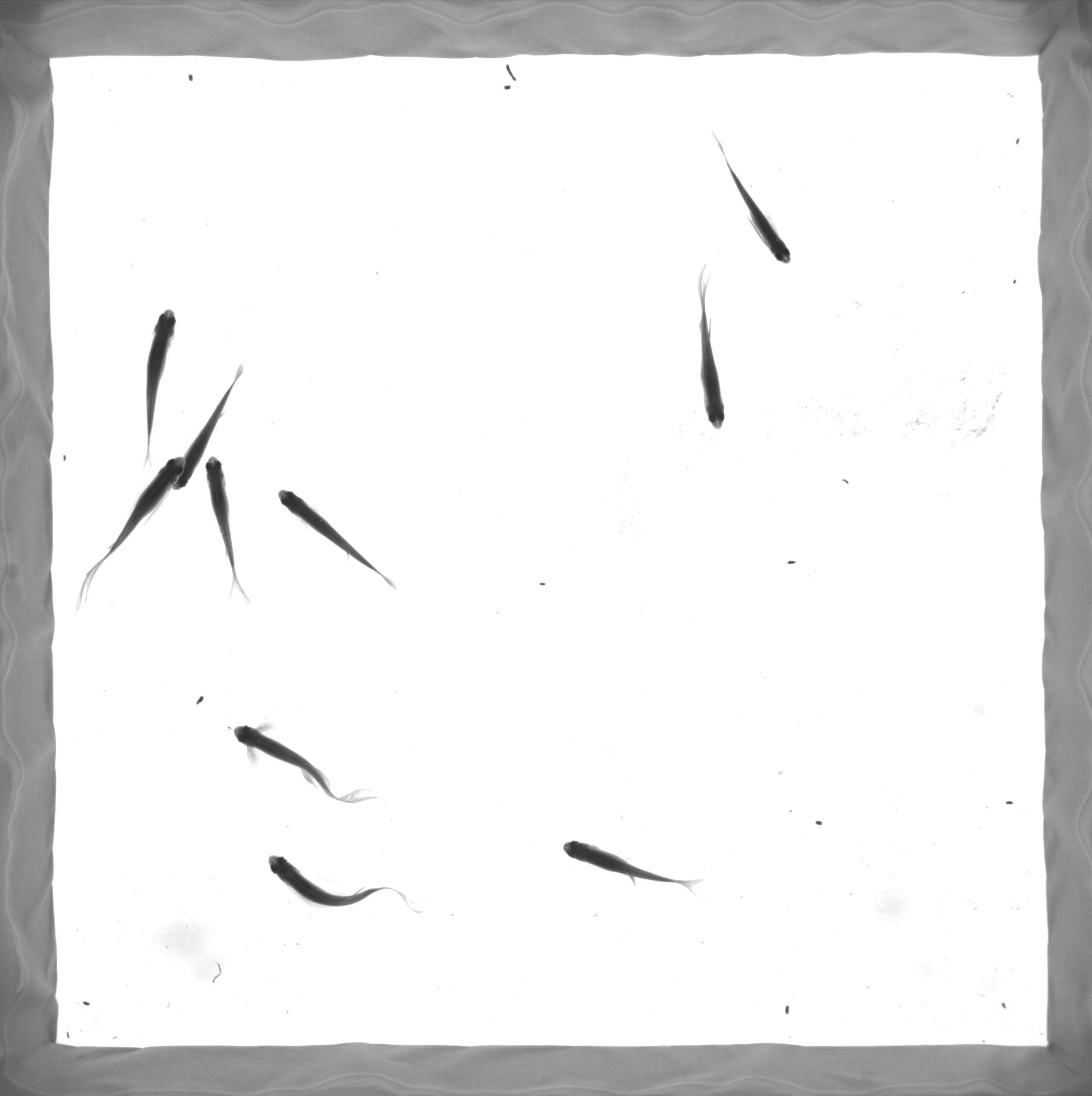

Supplement: S1 File — Source code of the proposed tracking system. (ZIP) [file pone.0154714.s002.zip › code_final/images/CoreView_275_Master_Camera_00090.jpg]

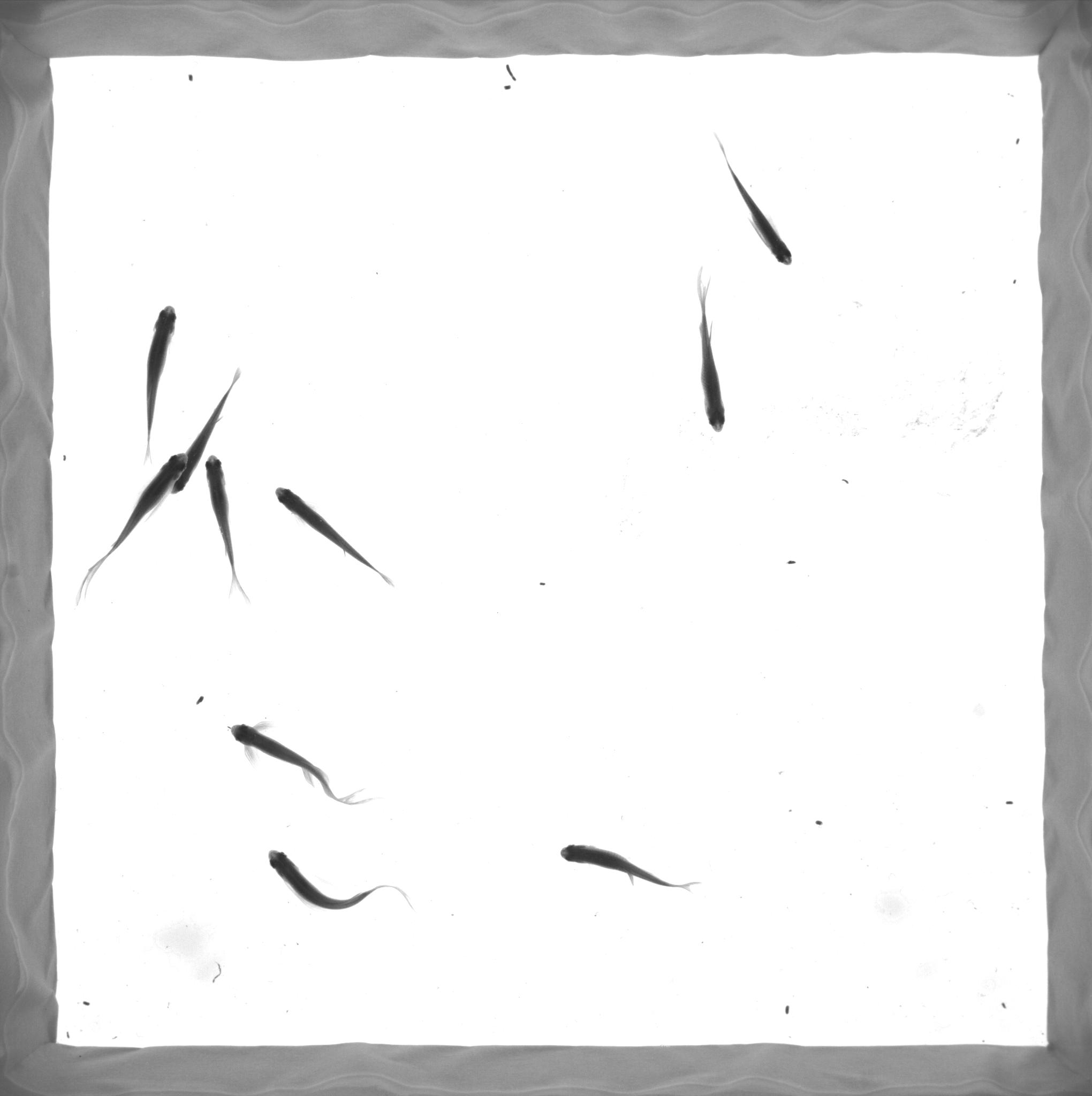

Supplement: S1 File — Source code of the proposed tracking system. (ZIP) [file pone.0154714.s002.zip › code_final/images/CoreView_275_Master_Camera_00091.jpg]

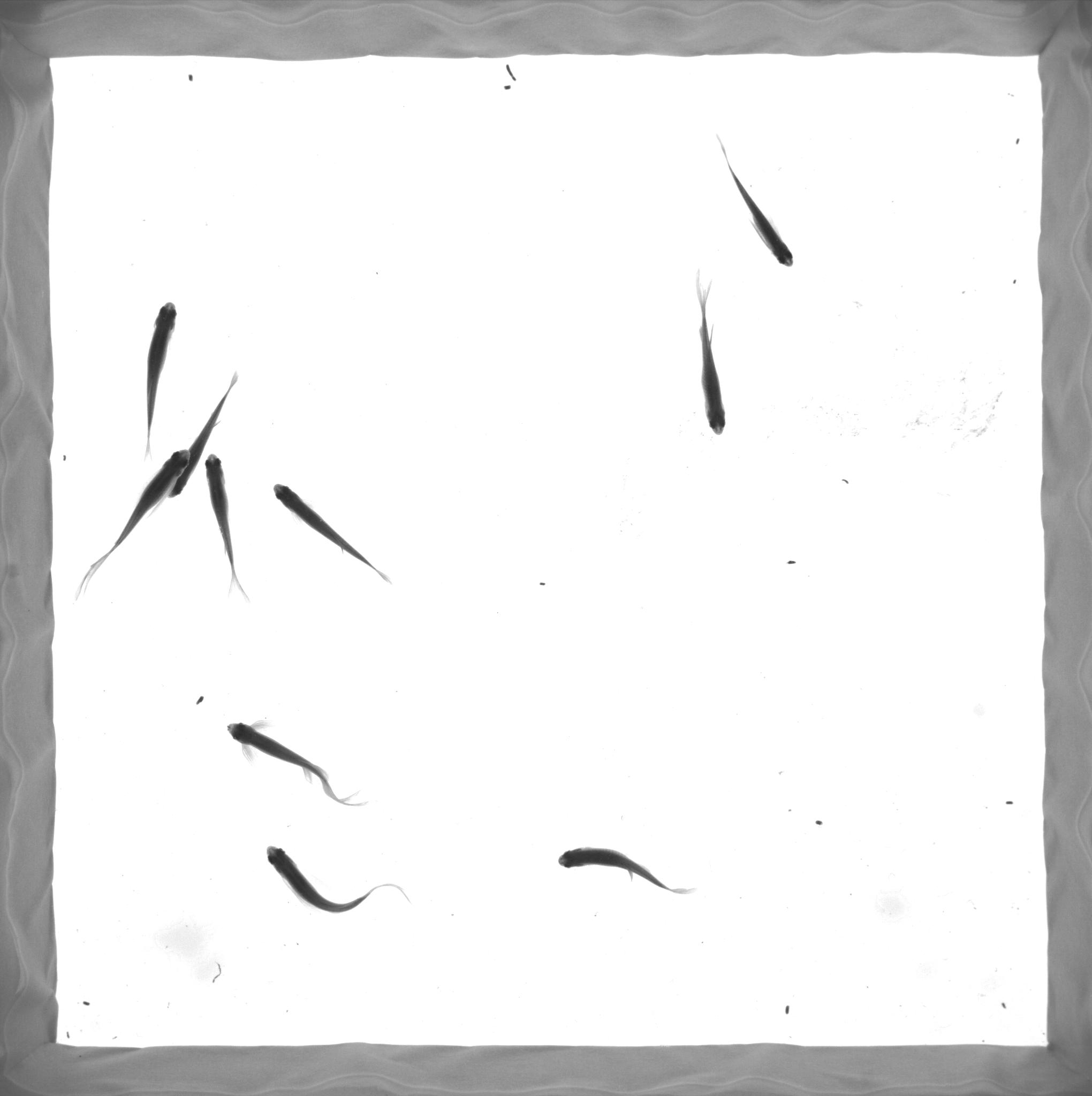

Supplement: S1 File — Source code of the proposed tracking system. (ZIP) [file pone.0154714.s002.zip › code_final/images/CoreView_275_Master_Camera_00092.jpg]

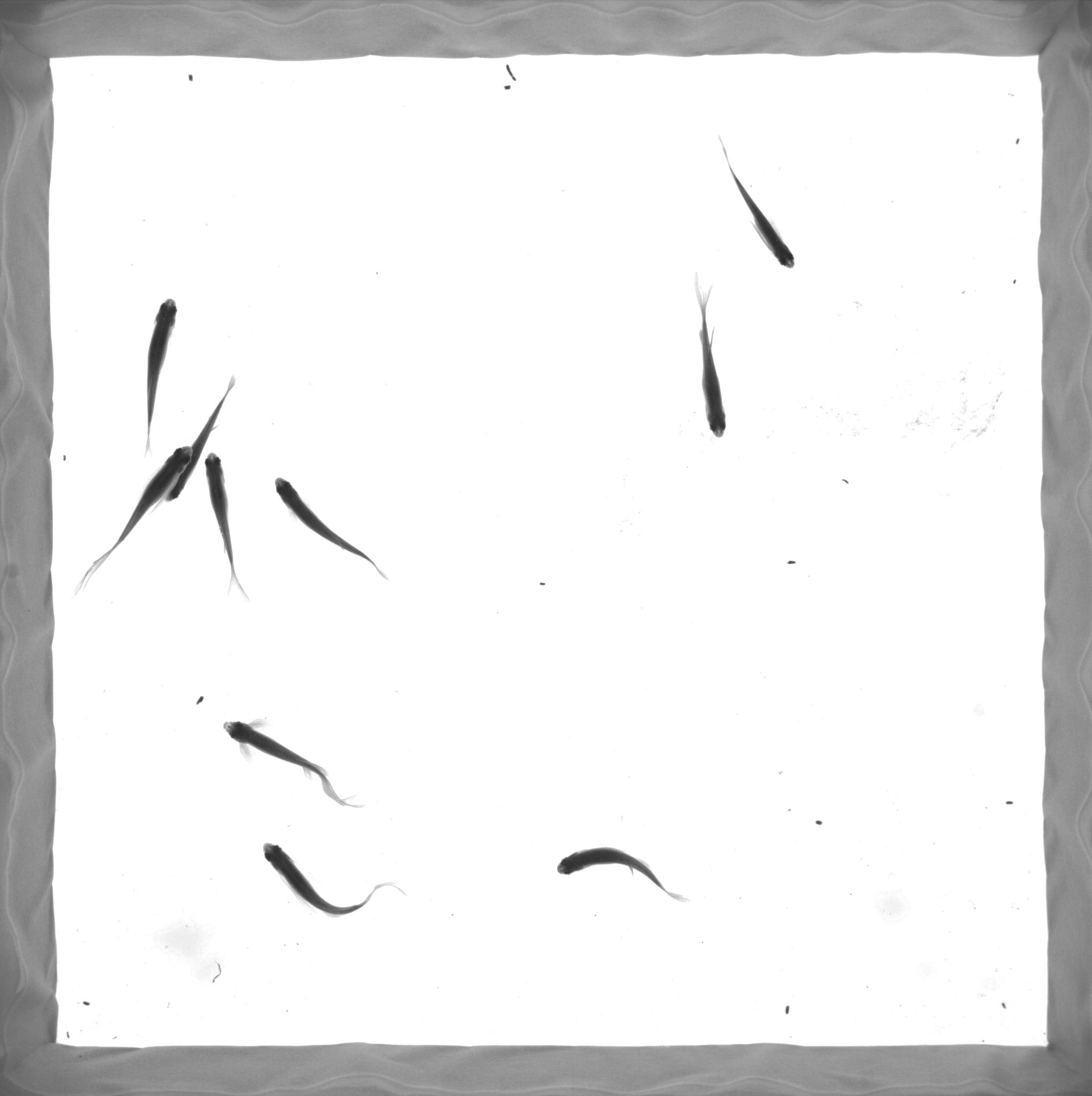

Supplement: S1 File — Source code of the proposed tracking system. (ZIP) [file pone.0154714.s002.zip › code_final/images/CoreView_275_Master_Camera_00093.jpg]

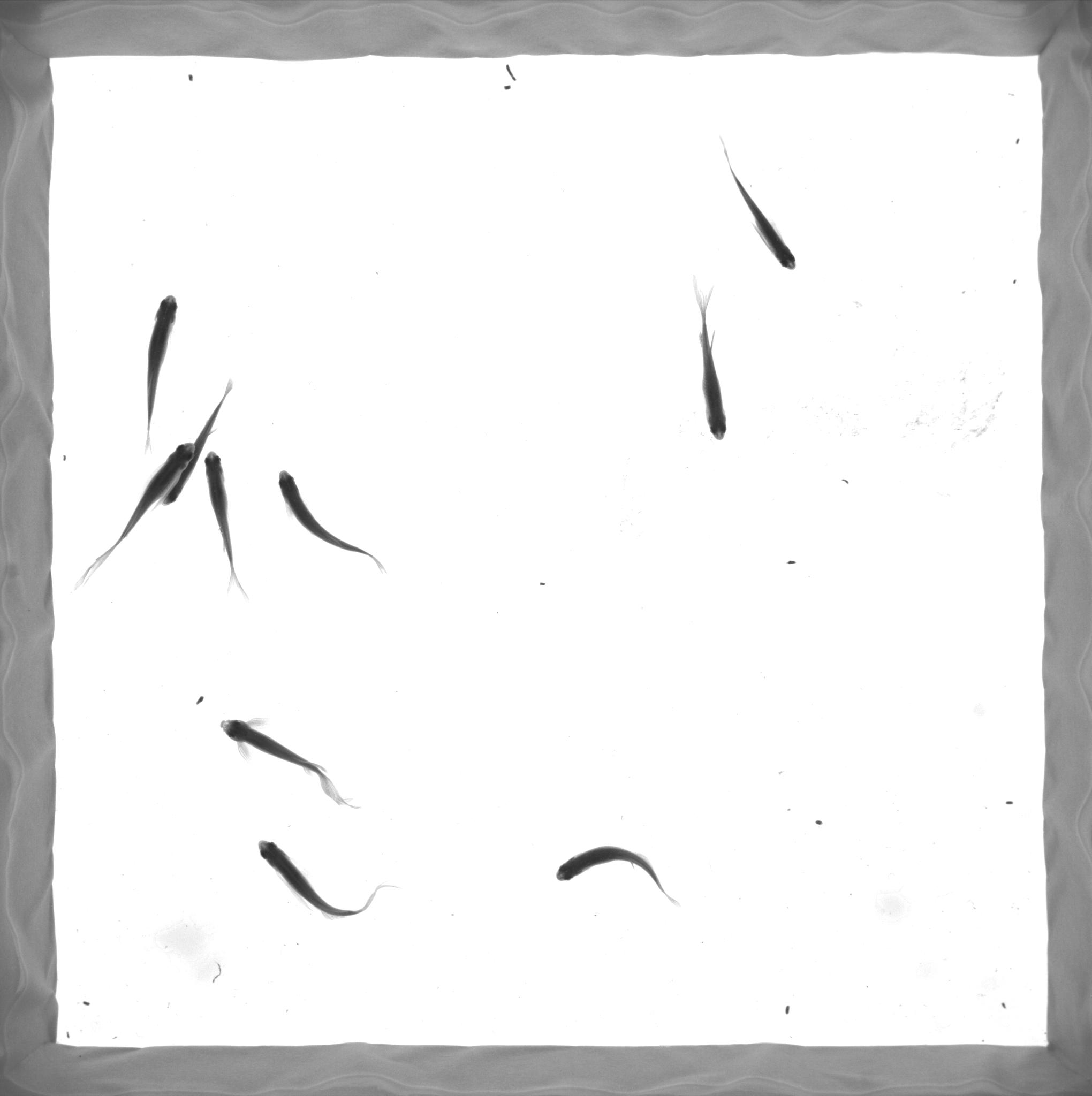

Supplement: S1 File — Source code of the proposed tracking system. (ZIP) [file pone.0154714.s002.zip › code_final/images/CoreView_275_Master_Camera_00094.jpg]

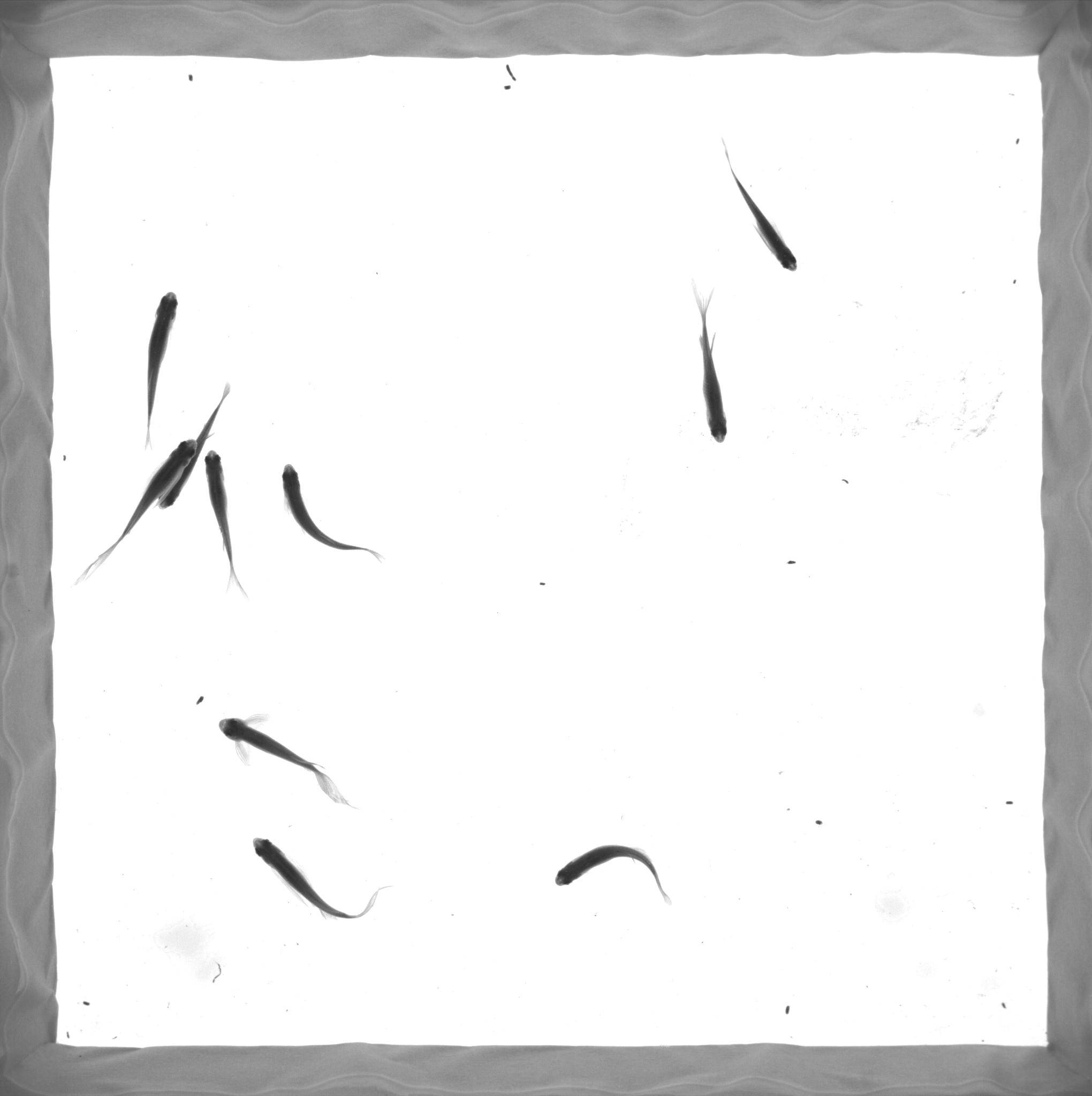

Supplement: S1 File — Source code of the proposed tracking system. (ZIP) [file pone.0154714.s002.zip › code_final/images/CoreView_275_Master_Camera_00095.jpg]

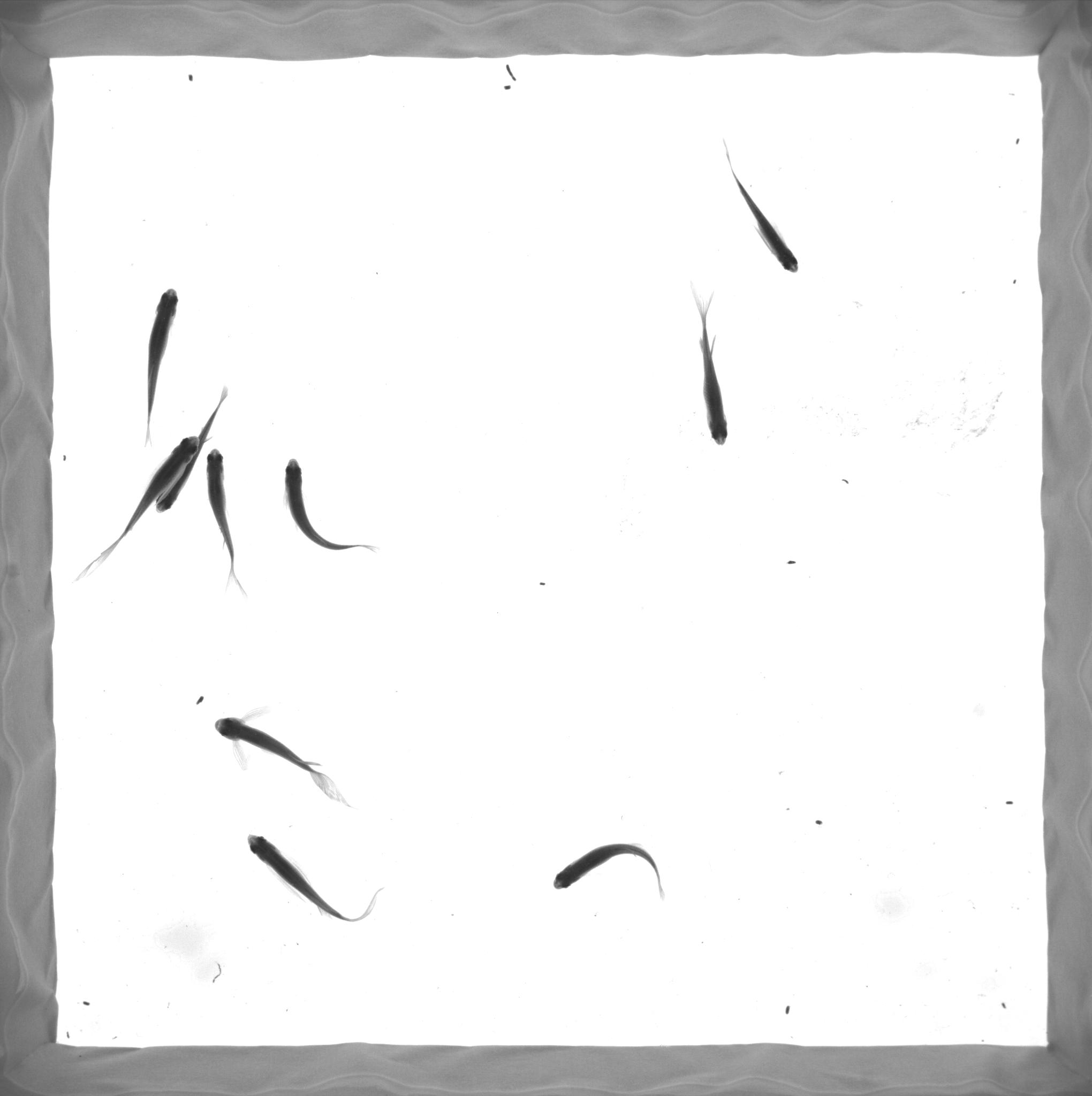

Supplement: S1 File — Source code of the proposed tracking system. (ZIP) [file pone.0154714.s002.zip › code_final/images/CoreView_275_Master_Camera_00096.jpg]

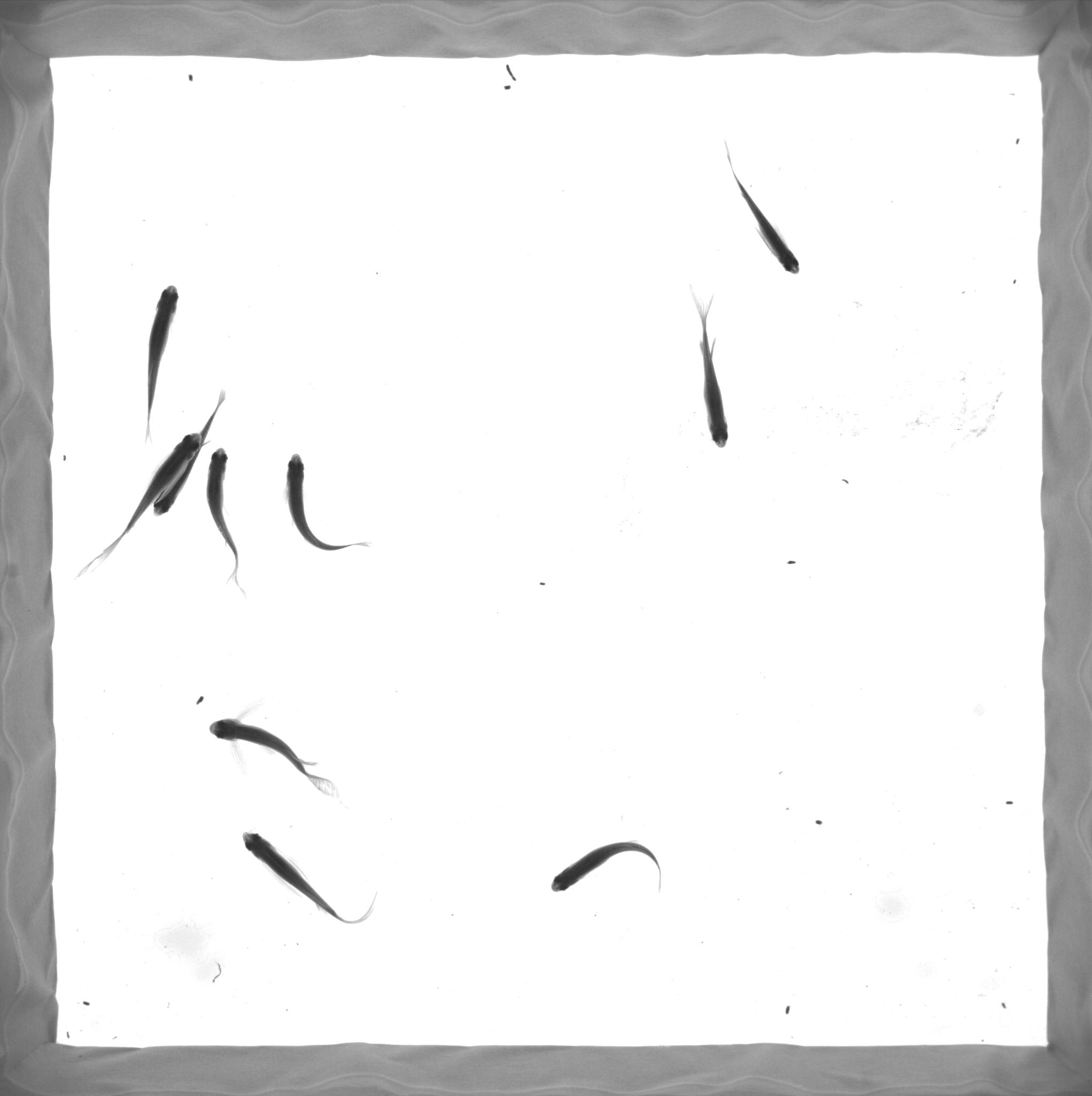

Supplement: S1 File — Source code of the proposed tracking system. (ZIP) [file pone.0154714.s002.zip › code_final/images/CoreView_275_Master_Camera_00097.jpg]

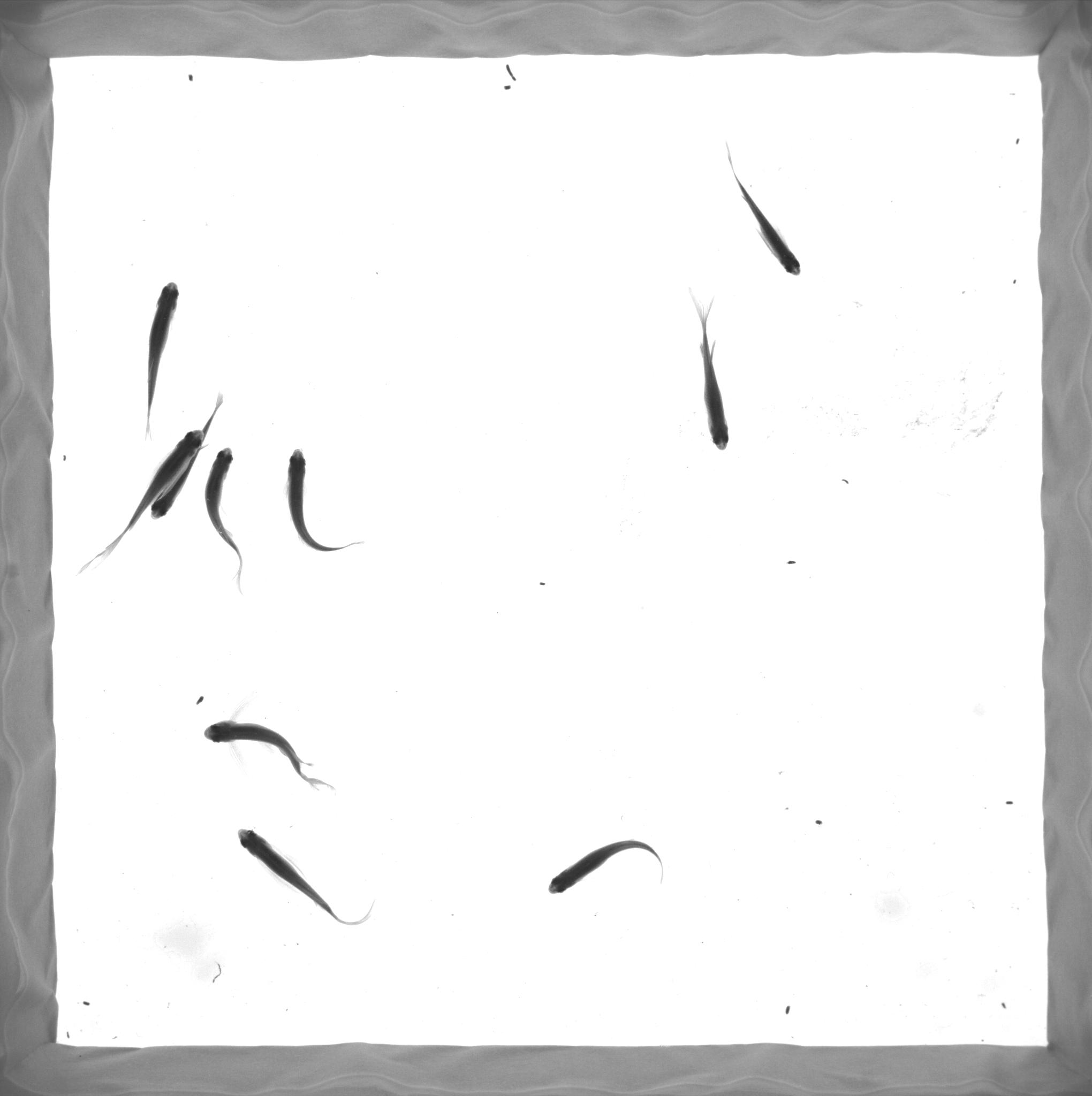

Supplement: S1 File — Source code of the proposed tracking system. (ZIP) [file pone.0154714.s002.zip › code_final/images/CoreView_275_Master_Camera_00098.jpg]
